# Iron-Catalyzed Dealkenylative Borylation of Alkenes via Ozonolysis

**DOI:** 10.1021/jacs.6c10840

**Published:** 2026-08-05

**Authors:** Kevin C. Wang, Emanuele Pinosa, Laura J. McCormick McPherson, Simon J. Coles, Jasper L. Tyler, Varinder K. Aggarwal

**Affiliations:** † School of Chemistry, 1980 University of Bristol, Cantock’s Close, Bristol BS8 1TS, U.K.; ‡ UK National Crystallography Service, Department of Chemistry, 7423 University of Southampton, Highfield Campus, Southampton SO17 1BJ, U.K.

## Abstract

Given the exceptional versatility of boronic esters as synthetic linchpins, strategies that directly transform naturally abundant motifs such as alkenes into boron-containing intermediates offer substantial value. Herein, we report the development of a dealkenylative borylation transformation that converts C­(sp^3^)–C­(sp^2^) bonds into C­(sp^3^)–B bonds via ozonolysis and iron catalysis. This protocol proceeds efficiently while tolerating a broad range of functional groups. Mechanistic studies indicate that the borylation reagent bis­(catecholato)­diborane (B_2_cat_2_) serves a dual purpose as both the boron source and the stoichiometric reductant. We demonstrate synthetic utility by applying this dealkenylative borylation reaction directly to naturally occurring steroids, terpenes, and terpenoids, as well as to the synthesis of natural products. Finally, the potential of our alkene-to-boron transformation to rapidly access structurally diverse analogues from feedstock compounds is highlighted through the divergent functionalization of the alkene-containing natural product betulinic acid.

## Graphical abstract

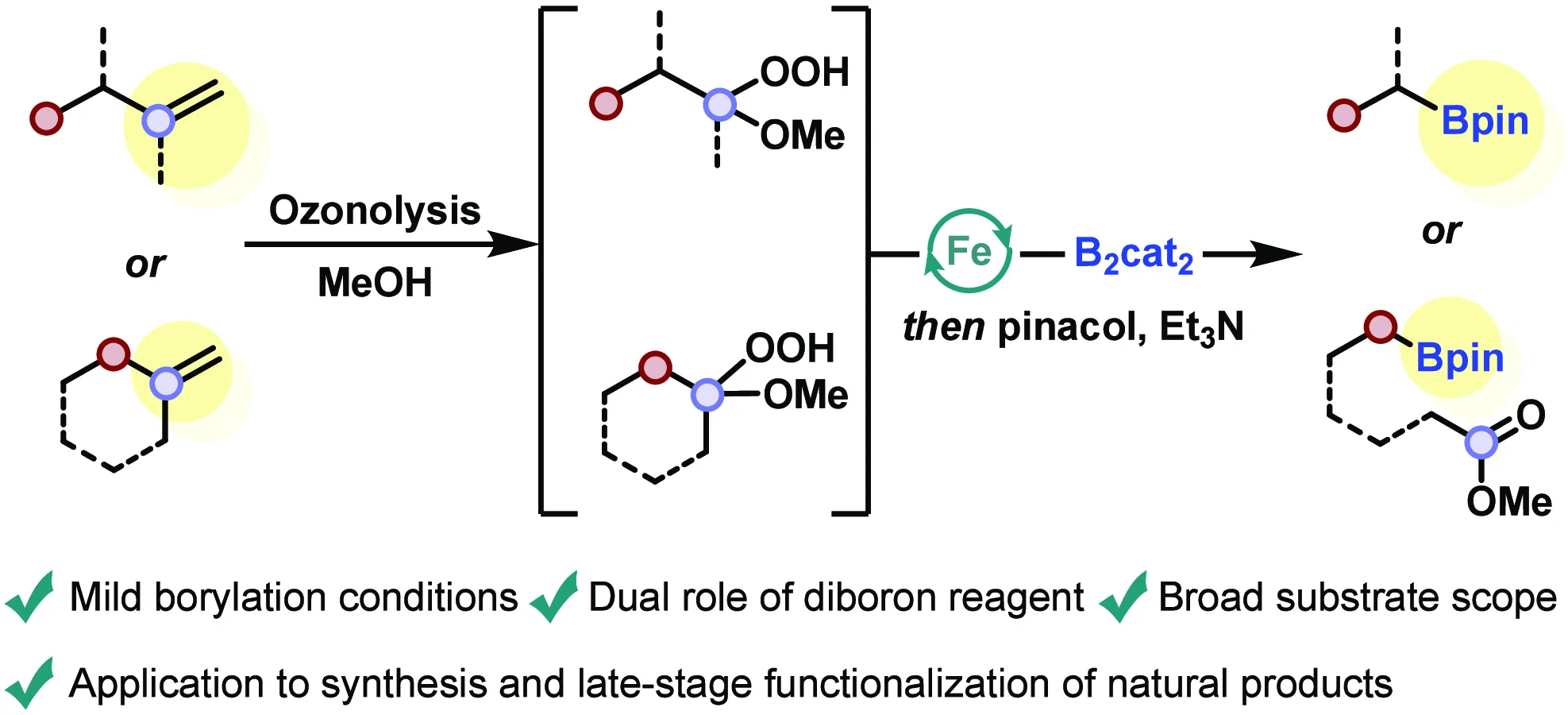

Methodologies that convert abundant functional groups present in structurally complex natural products into versatile synthetic handles are highly enabling and of considerable contemporary interest. Among such handles, boronic esters are particularly valuable because they can be transformed, often stereospecifically, into a broad range of functional groups including alcohols, amines, halides, alkenes, alkynes, and (hetero)­aromatics.^1^ As alcohols represent the most abundant functional group in natural products,^2,3^ considerable effort has been directed toward developing deoxygenative functionalization methods,^4^ including borylation,^5^ that exploit their ubiquity. Alkenes represent the next most abundant functional group,^3^ particularly prevalent within terpene,^6^ steroid,^7^ and polyketide frameworks.^8^ While the installation of boronic esters through alkene functionalization methods such as hydroboration is well-established,^9^ the development of a general transformation to achieve dealkenylative borylation remains highly desirable, with only one prior report demonstrating this transformation on a limited substrate set (see the Supporting Information (SI) for method benchmarking).^10^

In recent years, Kwon and co-workers have introduced a powerful new advance in dealkenylative functionalization, which has facilitated the conversion of alkenes into a range of functional groups^11^ including alkanes,^12^ sulfides,^13^ ketones,^14^ alkenes,^15^ alkynes,^16^ amines^17^ and halides^18^ (Scheme 1a). In these transformations, ozonolysis of the alkene in the presence of MeOH generates a peroxy acetal intermediate that subsequently undergoes single-electron reduction, via an interaction with a Fe­(II) or Cu­(I) reagent, to induce C–C bond cleavage and generate an alkyl radical. This radical intermediate can then be intercepted by an appropriate radical trapping reagent to furnish the functionalized product. Given prior reports demonstrating efficient trapping of alkyl radicals by diboron reagents,^19^ we aimed to investigate whether radicals generated from peroxy acetal intermediates could also be intercepted in this manner, thereby enabling dealkenylative borylation (Scheme 1b). However, this transformation was anticipated to pose significant challenges, including the potential incompatibility of the peroxy acetal intermediate with oxidation-sensitive diboron reagents and the requirement for a reducing agent that could enable catalyst turnover without interfering with any components of this redox system. Herein, we report the successful development of a catalytic protocol for dealkenylative borylation (Scheme 1c). Mechanistic studies are consistent with the formation of a peroxy acetal intermediate, generated via ozonolysis, which undergoes iron–catalyzed alkyl radical formation and subsequent radical capture with bis­(catecholato)­diborane B_2_cat_2_. Importantly, the resulting boron radical adduct facilitates catalyst recycling, obviating the need for stoichiometric reductants and providing an efficient method for converting feedstock alkenes into valuable, linchpin-containing compounds.

**Scheme 1:**
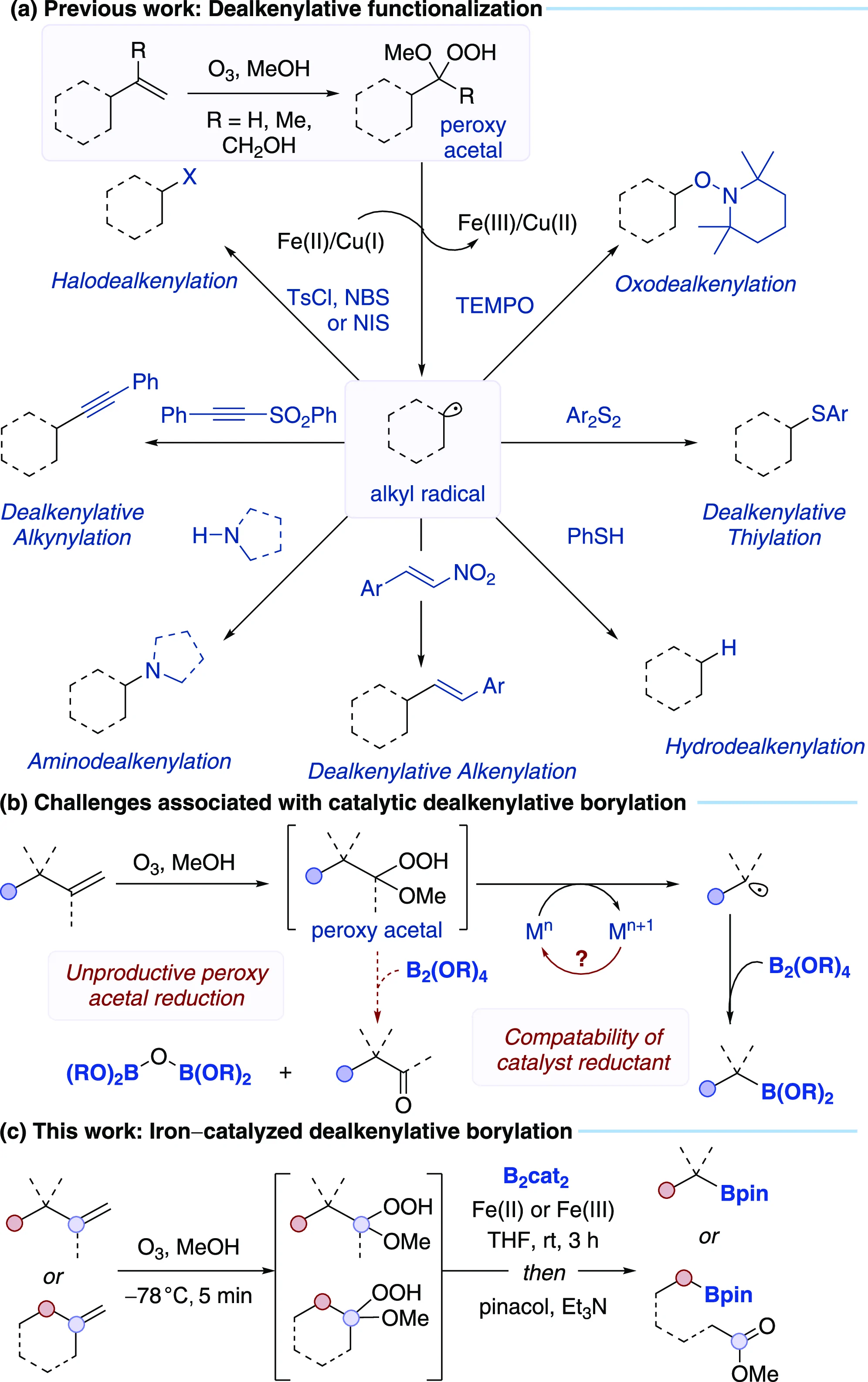
Dealkenylative Functionalization and Our Design for Dealkenylative Borylation

We began our investigation by employing 4-phenyl-1-butene (**1a**) as a model substrate and subjecting this compound to ozonolysis, followed by removal of methanol and the addition of a metal reductant, ligand, solvent, and B_2_cat_2_. After a systematic screen of conditions, it was determined that a mixture of FeCl_2_, dimethoxyethane (DME), and B_2_cat_2_ in THF for 3 h at room temperature represented the optimum conditions (Table 1, Entry 1). Although previous reports of dealkenylative functionalization reactions typically require either stoichiometric amounts of transition-metal complex^11–14^ or an external reductant,^15,17^ we observed that substoichiometric quantities of Fe­(II) catalyst were capable of delivering acceptable levels of the desired product. To facilitate analysis and subsequent purification, the catechol boronic ester was transformed into the pinacol boronic ester and isolated in 45% yield for this entire reaction sequence.

During the course of our optimization study, we identified a series of factors that were key to reaction efficiency. First, close analysis of the reaction mixtures showed the formation of ozonolysis-type ester and aldehyde side products. These species, which also observed by Kwon and co-workers,^12,16,17^ are proposed to arise from the intermediate alkoxy radical generated upon peroxy acetal reduction, and account for a significant proportion of the mass loss in these systems (see SI for details). It was also determined that prestirring of the metal catalyst, the ligand, and B_2_cat_2_ was critical to product formation, as the direct combination of all reagents at the outset resulted in substantially diminished yield (Table 1, Entry 2). This was rationalized by the increased solubility of the iron catalyst through ligand complexation upon prestirring. The efficiency of the catalyst turnover was highlighted by observations that stoichiometric amounts of FeCl_2_ gave similar results to 20 mol % loading (Table 1, Entry 3). Lower loadings of catalyst were investigated but provided a decrease in efficiency (Table 1, Entry 4). The reaction proceeds in diminished yield when omitting DME or reducing B_2_cat_2_ loading, indicating that 3 equivalent of diboron reagent is required to achieve maximum efficiency (Table 1, Entries 5–6). In addition, switching to alternative solvents or employing B_2_pin_2_ caused significant loss in yield (Table 1, Entries 7–8). Employing Cu­(I) as the catalyst was shown to severely affect reaction efficiency (Table 1, Entry 9), presumably because of its slower rate of peroxy acetal reduction (4 × 10^3^ M^–1^·s^–1^)^17^ compared to Fe­(II) (1.2 × 10^7^ M^–1^·s^–1^),^16^ enabling competing reduction with B_2_cat_2_ to generate the corresponding aldehyde (see SI Table S4, Entry 4). Interestingly, Fe­(acac)_3_ could be employed under identical conditions to provide **2a** in 43% yield (Table 1, Entry 11). Although our establishment of catalytic reactivity in this system evidences a Fe­(II)/Fe­(III) redox couple, this result clearly indicates the existence of a pathway for the *in situ* generation of the proposed catalytically active Fe­(II) species. Although Fe­(acac)_3_ was shown to be similarly effective as the FeCl_2_/DME system in the model reaction, the latter proved effective across a broader range of substrates. Nevertheless, the Fe­(III) conditions were taken forward as our alternative set of optimal conditions.

**Table 1:**
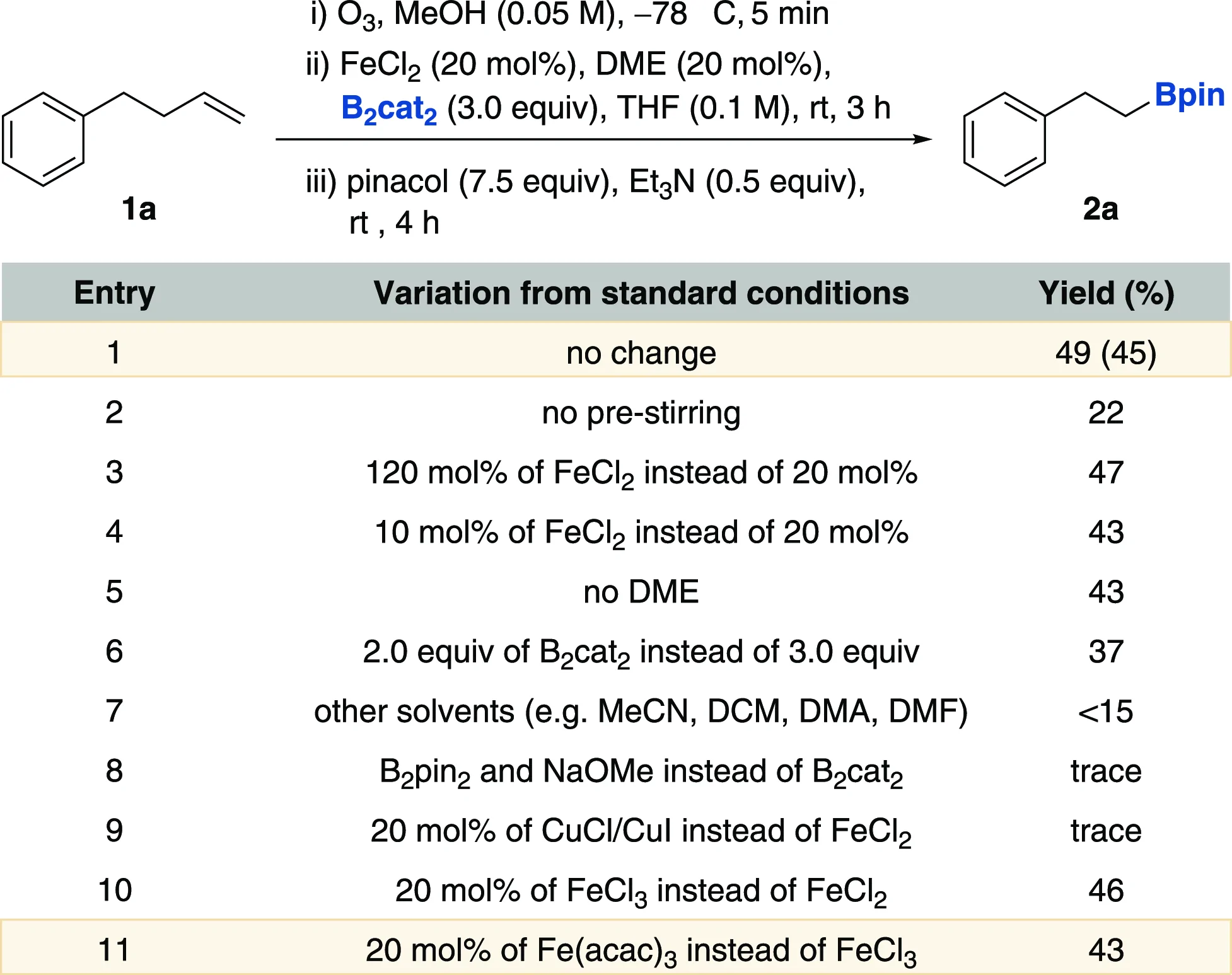
Optimization Studies for Fe−Catalyzed Dealkenylative Borylation^a^

a All reactions were performed on a 0.2 mmol scale. Yields were determined by quantitative GC-MS analysis.

With two sets of optimized conditions in hand, we explored the substrate scope of the reaction (Table 2). 1,1-Disubstituted alkene **1b**, bearing a methyl substituent at the α-position, afforded a significantly higher yield than the corresponding terminal alkene substrate **1a** (63% vs 45%). This yield enhancement arises due to the absence of an α-proton in the key peroxy acetal intermediate which suppresses a deleterious HAT pathway that accounts for a noticeable proportion of mass loss (see SI for discussions on side-product formation). The methodology proved general across a series of simple alkenes (**1c**–**1e**), and a diverse set of terpene substrates such as dihydrocarvone (**1f**) and nootkatone (**1k**), to afford the corresponding boronic esters in good yields. (−)-Isopulegol (**1g**) initially provided **2g** in only 19% yield,^20^ but after silyl ether protection **1h** gave the desired boronic ester **2h** in a much improved 67% yield. Interestingly, limonene oxide **1i** was shown to undergo dealkenylative borylation accompanied by a Meinwald rearrangement^21^ to deliver aldehyde **2i**. The protocol was further applicable to more complex, functionalized molecules. Specifically, phytol (**1l**), liquid-crystal precursor **1m**, and fludioxonil-derived alkene **1n**, furnished the corresponding products (**2l**–**2n**) in good yields. Steroid-derived alkene **1o**, as well as methyl and *tert*-butyl esters of the pentacyclic triterpenoid betulinic acid (**1p–1r**), also underwent borylation with excellent diastereoselectivity. Interestingly, most substrates performed better with the Fe­(II) system with few exceptions, although the origin of these observations was not obvious (see SI for details).

**Table 2:**
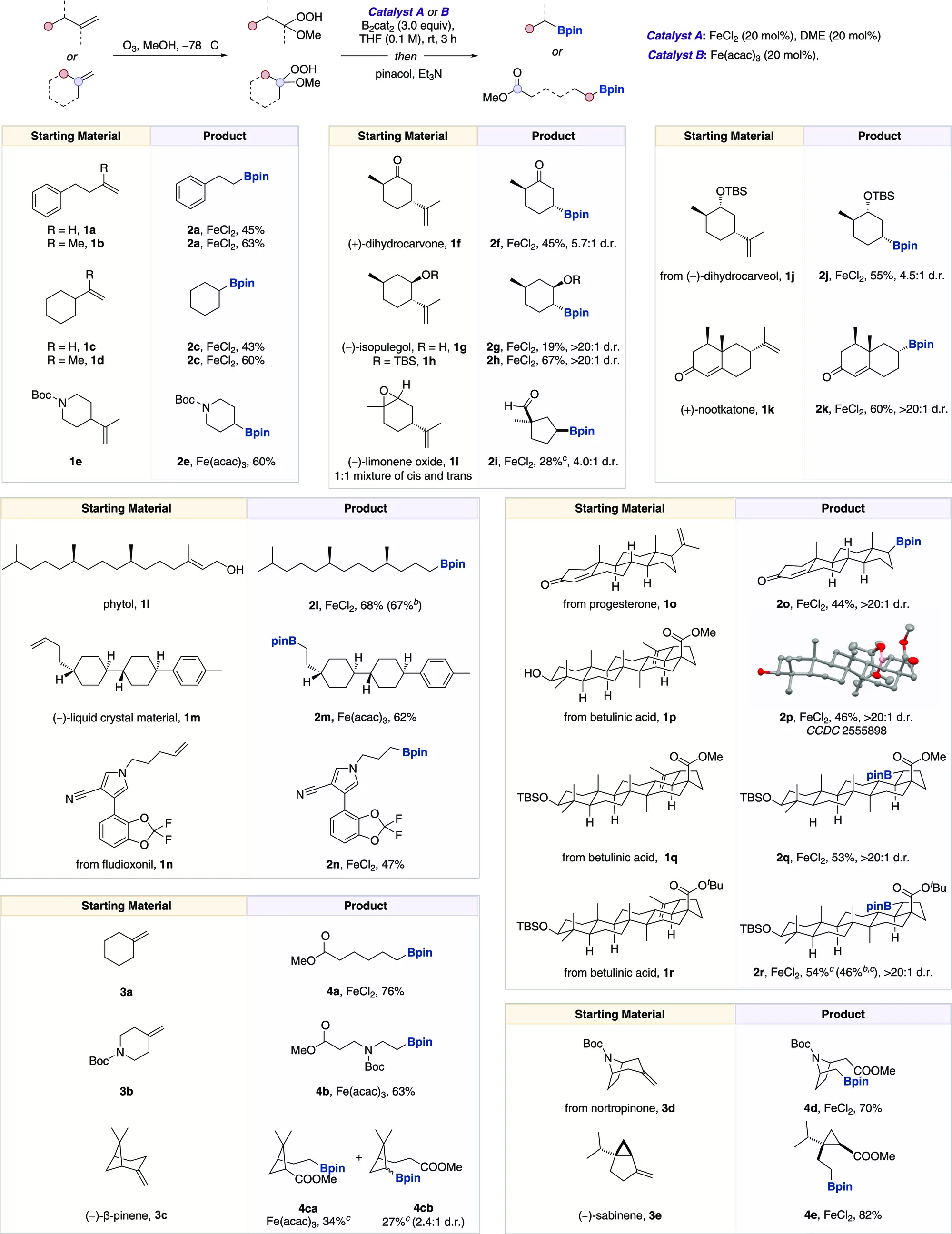
Substrate Scope^a^

a All reactions were performed on a 0.2 mmol scale. Yields are of isolated products after chromatographic purification. The d.r. was determined by quantitative GC-MS or ^1^H NMR analysis.

b The reaction was performed on a 2.0 mmol scale.

c The reaction was performed at 0.05 M concentration.

We next evaluated exocyclic alkenes, which were anticipated, upon β-scission-promoted ring-opening, to furnish acyclic products bearing methyl ester and boronic ester functional handles that are highly amenable to further derivatization. We successfully engaged methylene-cyclohexane (**3a**) and *tert*-butyl 4-methylenepiperidine-1-carboxylate (**3b**), as well as rigid natural product frameworks such as (−)-β-pinene (**3c**), nortropinone derived alkene (**3d**), and (±)-sabinene (**3e**). The high regioselectivity observed for (±)-sabinene is noteworthy and presumably originates from preferential β-scission to form a primary radical over the alternative cyclopropyl radical, an observation that has been ascribed to the formation of the C=O double bond being facilitated by overlap with the C–C bonding orbitals of the cyclopropane ring.^22^

Internal cyclic alkenes, e.g., (−)-α-pinene, did not furnish the desired boronic ester product, presumably because the aldehyde generated from ozonolysis can react with the diboron reagent^23^ (see SI for full details on failed substrates). Tertiary radical precursors failed to deliver the targeted borylated products, forming the corresponding ketone side product instead (see SI). Given that the formation of peroxy acetal intermediates from similarly substituted alkenes has been previously reported,^15,16^ and that β-scission is expected to be facile for these substrates, it is anticipated that the ketone side products arise from reduction of the peroxy acetal by B_2_cat_2_ in preference to SET reduction by Fe­(II).

We then looked to explore the main aspects of our reaction system in which the mechanism diverges from previously established dealkenylative functionalization reactions. Namely, we wished to gain insight into how the catalytic turnover from Fe­(III) to Fe­(II) proceeded under our reaction conditions. We were intrigued by the observation that Fe­(III) catalyst precursors were highly active in this system and that prestirring of the Fe­(acac)_3_ complex with B_2_cat_2_ was necessary to achieve efficient reactivity, indicating an initial catalyst activation process. To elucidate the nature of the active species formed during this prestirring, ^1^H and ^11^B NMR studies were performed (Figure 1a). ^1^H NMR analysis of the mixture of B_2_cat_2_ and Fe­(acac)_3_ in THF revealed the formation of a species consistent with formation of Bcat–acac and concomitant reduction of paramagnetic Fe­(III) to diamagnetic Fe­(II) by the diboron reagent (see SI for details). ^11^B NMR analysis of the same mixture provided spectral data that matched that of independently synthesized Bcat–acac (Figure 1b). Although this pathway does not account for catalyst turnover in the reaction mechanism, we hypothesize that this is likely to occur through the interaction of the alkyl radical–B_2_cat_2_ adduct with the Fe­(III) complex.^5a,5b,19a,19e,24^ This was evidenced by the detection of Bcat–OH, the expected byproduct from this interaction, highlighting the dual role of B_2_cat_2_ in acting as the boron source and facilitating catalyst turnover.

**Figure 1:**
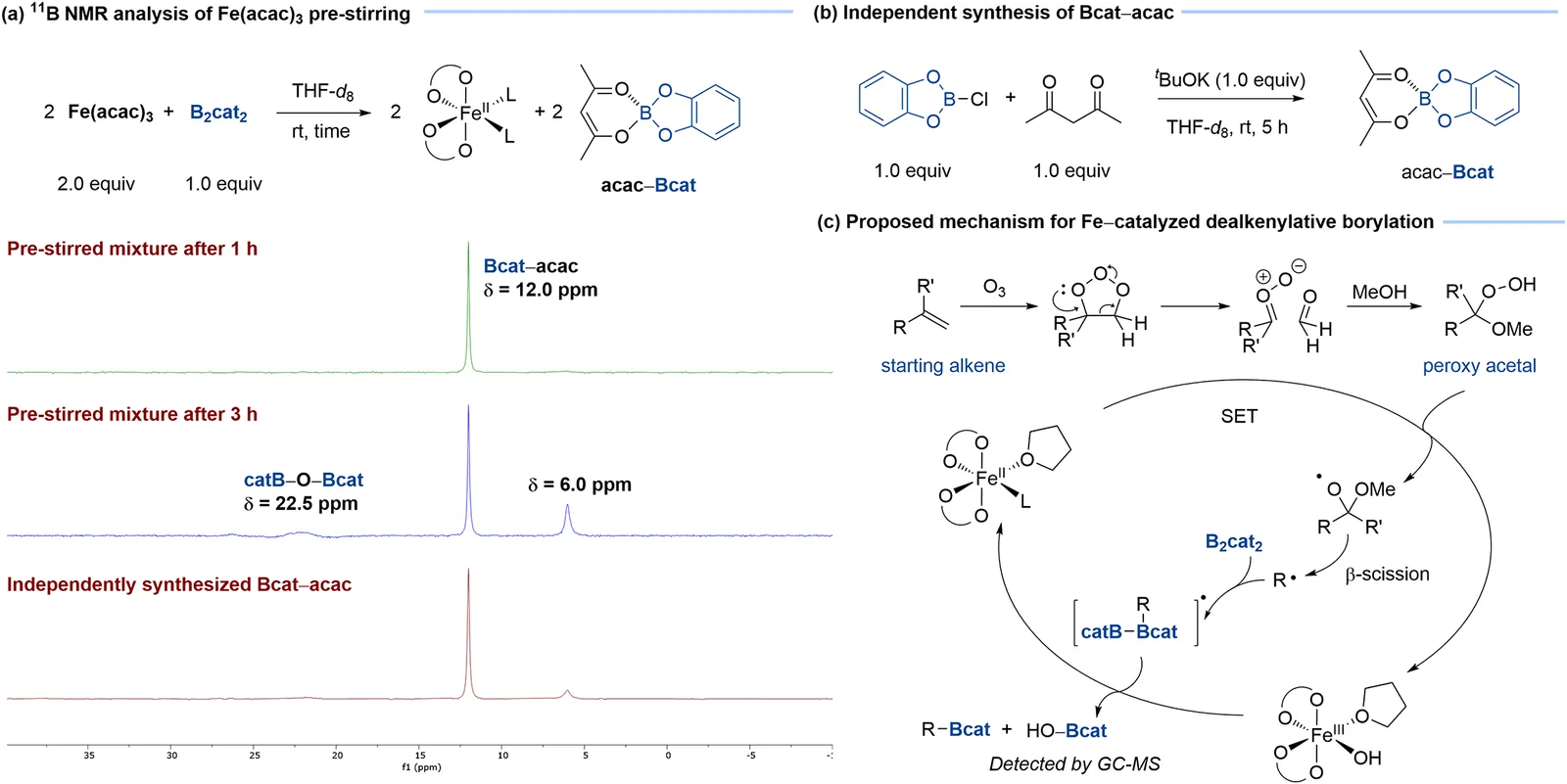
Mechanistic studies. (a) ^11^B NMR analysis of Fe­(acac)_3_ prestirring. (b) Independent synthesis of Bcat–acac. (c) Plausible mechanism for the Fe−catalyzed dealkenylative borylation reaction.

According to these observations, a plausible catalytic cycle is proposed (Figure 1c). The sequence begins with ozonolysis of the alkene substrate in methanol to generate a peroxy acetal intermediate. Single-electron transfer (SET) from the active Fe­(II) species then generates an alkoxy radical and Fe­(III).^25^ Although B_2_cat_2_ is capable of competing with this interaction through reduction of the peroxy acetal intermediate, we anticipate that the Fe­(II) SET pathway occurs at a higher rate (1.2 × 10^7^ M^–1^·s^–1^).^16^

This mechanistic proposal aligns with the observations made during mechanistic investigations of related dealkylative radical functionalization reactions.^11,14,15^ Subsequent β-scission of the alkoxy radical affords the corresponding alkyl radical, which can then be trapped by B_2_cat_2_ to form a radical adduct. This species is proposed to reduce Fe­(III) back to Fe­(II), generating Bcat–OH and thereby closing the catalytic cycle.

To further demonstrate the synthetic utility of dealkenylative borylation, we applied our protocol to the assembly of naturally occurring molecules via efficient access to advanced synthetic intermediates (Figure 2a). Accordingly, boronic ester **2l**, synthesized from phytol under standard conditions, was elaborated to rice moth pheromone **5** by employing our group’s previously reported lithiation-borylation-oxidation methodology (47% yield over 2 steps).^26^ Furthermore, Suzuki-Miyaura cross-coupling of **2l** with naphthoquinone triflate **9**, followed by oxidation, delivered solanoquinone **10**, found in cured tobacco leaves in 71% yield. Both syntheses showed an improvement in step economy from the previously reported methods.^27,28^

**Figure 2:**
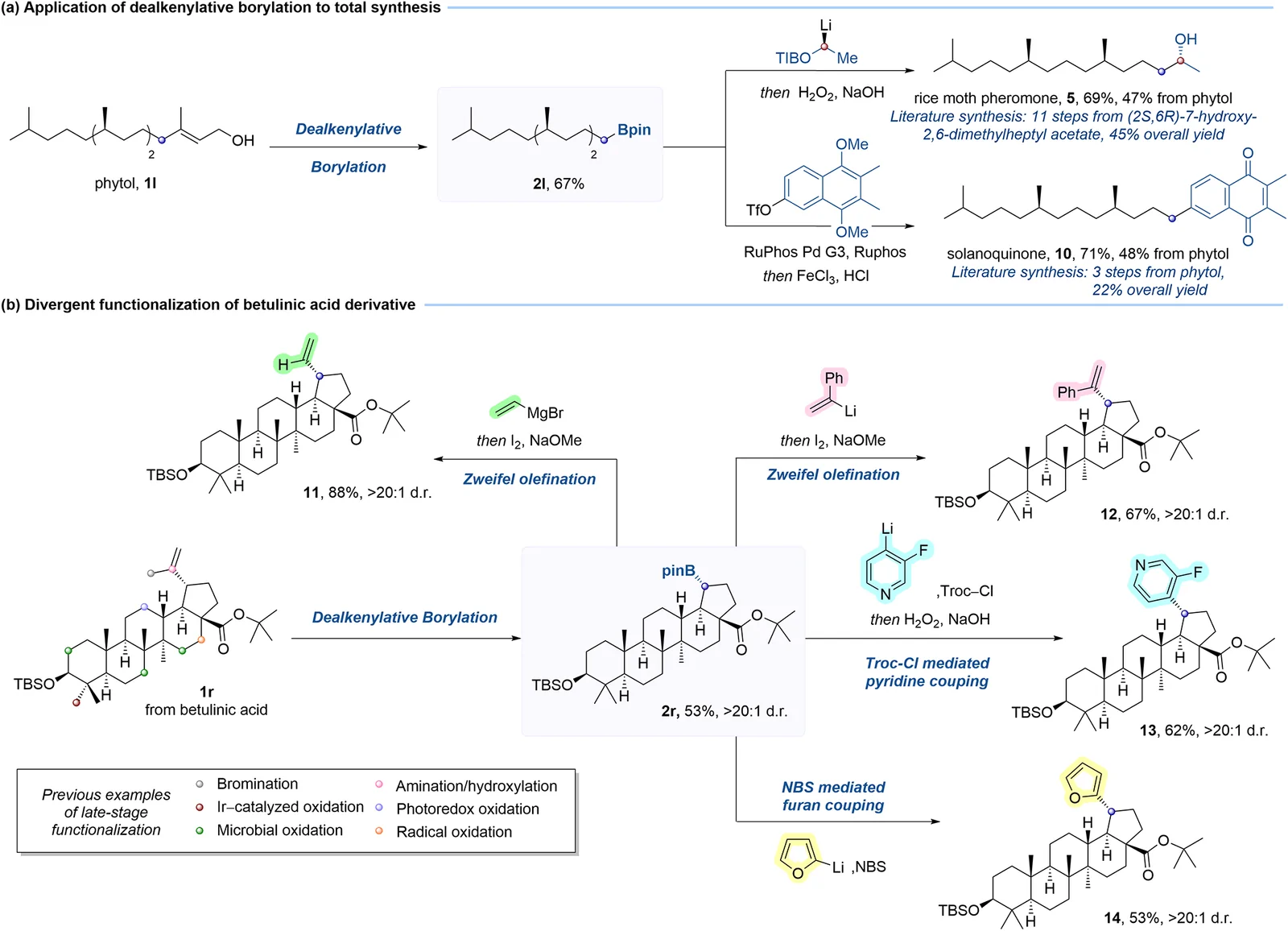
(a) Application of dealkenylative borylation to the synthesis of rice moth pheromone **5** and solanoquinone **10**, from phytol. (b) Diversification of betulinic acid derivative **2s** (see SI for full experimental details).

As well as streamlining the synthesis of known target compounds, this transformation was applied to the divergent late-stage functionalization of alkene-containing betulinic acid to access a range of natural product analogues (Figure 2b). Despite extensive late-stage functionalization studies, driven by its anti-HIV, antibacterial, and antimalarial activity,^29^ prior modifications have focused on the hydroxyl and carboxyl groups,^30^ with limited examples of modification to the carbon framework.^31,32^ Even fewer alkene derivatization reactions have been reported, via either allylic bromination and subsequent nucleophilic substitution,^33^ ozonolysis,^34^ or olefin hydration.^31^ Given this limited scope for functionalization at this position, we engaged borylated betulinic acid derivative **2r** in stereospecific coupling reactions under a variety of conditions with excellent diastereocontrol. For example, Zweifel-type olefination yielded formally demethylated betulinic acid derivative **11** and phenyl-substituted alkene **12** in good yields.^35^ Furthermore, stereospecific cross-coupling with 3-fluoropyridine and furan proceeded to give **13** and **14** in 62% and 53% yields, respectively.^36^ These overall transformations, which have not previously been achieved on this scaffold using established methods, all proceeded with excellent diastereocontrol and illustrate the capacity of the boronic ester handle to expand the chemical space available for biological evaluation of alkene-containing bioactive molecules.

In conclusion, we developed a catalytic dealkenylative borylation reaction for the synthesis of alkyl boronic esters directly from alkene-containing molecules. Key to this transformation is the role of the diboron reagent B_2_cat_2_, which serves as both a boron source and a reductant for the iron catalyst. This protocol employs inexpensive and readily available reagents and is applicable to both terminal and exomethylene alkenes, exhibiting broad functional group tolerance. The synthetic utility of the method has been demonstrated through efficient access to structurally complex, biologically active molecules and their novel derivatives. Given the versatility of downstream stereospecific transformations available for alkyl boronic esters, we anticipate that this strategy will find broad applications in both organic synthesis and medicinal chemistry.

## Supplementary Material



## Notes

Experimental procedures and characterization data for new compounds (PDF)

## References

[1] Armstrong R. J., Sandford C., García-Ruiz C., Aggarwal V. K. (2017). Conjunctive functionalization of vinyl boronate complexes
with electrophiles: a diastereoselective three-component coupling. Chem. Commun..

[2] Henkel T., Brunne R. M., Müller H., Reichel F. (1999). Statistical Investigation
into the Structural Complementarity of Natural Products and Synthetic
Compounds. Angew. Chem., Int. Ed..

[3] Ertl P., Schuhmann T. (2019). A Systematic
Cheminformatics Analysis of Functional
Groups Occurring in Natural Products. J. Nat.
Prod..

[4] Yuan Y., Liu L., Zhang F., Zhang Y.-N., Huo C. (2025). Recent advances in electrochemical deoxygenative functionalization
of alcohols and their derivatives. Tetrahedron
Chem..

[5] Friese F. W., Studer A. (2019). Deoxygenative Borylation of Secondary
and Tertiary Alcohols. Angew. Chem., Int. Ed..

[6] Hanssens J., Meneses D., Saya J. M., Orru R. V. A. (2025). Terpenes and
Terpenoids: How can we use them?. Eur. J. Org.
Chem..

[7] Hanson J. R. (2002). Steroids:
reactions and partial synthesis. Nat. Prod.
Rep..

[8] Paterson I., Lam N. Y. S. (2018). Challenges and
discoveries in the total synthesis of
complex polyketide natural products. J. Antibiot..

[9] Geier S. J., Vogels C. M., Melanson J. A., Westcott S. A. (2022). The transition
metal-catalysed
hydroboration reaction. Chem. Soc. Rev..

[10] Chen S.-C., Zhu Q., Chen H., Chen Z., Luo T. (2023). Pro-aromaticity Enabled
Dealkenylative Functionalizations via Photo-Excitation and Oxidation. Chem. Eur. J..

[11] Dworkin J. H., Dehnert B. W., Kwon O. (2023). When all C–C
breaks LO–Ose. Trends Chem..

[12] Smaligo A. J., Swain M., Quintana J. C., Tan M. F., Kim D. A., Kwon O. (2019). Hydrodealkenylative C­(sp3)–C­(sp2) bond fragmentation. Science.

[13] Smaligo A. J., Kwon O. (2019). Dealkenylative Thiylation of C­(sp3)–C­(sp2) Bonds. Org. Lett..

[14] Smaligo A. J., Wu J., Burton N. R., Hacker A. S., Shaikh A. C., Quintana J. C., Wang R., Xie C., Kwon O. (2020). Oxodealkenylative Cleavage
of Alkene C­(sp3)–C­(sp2) Bonds: A Practical Method for Introducing
Carbonyls into Chiral Pool Materials. Angew.
Chem., Int. Ed..

[15] Swain M., Sadykhov G., Wang R., Kwon O. (2020). Dealkenylative Alkenylation:
Formal σ-Bond Metathesis of Olefins. Angew.
Chem., Int. Ed..

[16] Swain M., Bunnell T. B., Kim J., Kwon O. (2022). Dealkenylative Alkynylation
Using Catalytic Fe^II^ and Vitamin C. J. Am. Chem. Soc..

[17] He Z., Moreno J. A., Swain M., Wu J., Kwon O. (2023). Aminodealkenylation:
Ozonolysis and copper catalysis convert C­(sp3)–C­(sp2) bonds
to C­(sp3)–N bonds. Science.

[18] Dehnert B. W., Yin Y., Kwon O. (2024). Halodealkenylation:
Ozonolysis and Catalytic Fe^II^ with Vitamin C Convert C­(sp3)–C­(sp2)
Bonds to C­(sp3)–Halide
Bonds. Org. Lett..

[19] Li C., Wang J., Barton L. M., Yu S., Tian M., Peters D. S., Kumar M., Yu A. W., Johnson K. A., Chatterjee A. K., Yan M., Baran P. S. (2017). Decarboxylative
borylation. Science.

[20] LaPorte A. J., Shi Y., Hein J. E., Burke M. D. (2022). Stereospecific Csp^3^ Suzuki–Miyaura
Cross-Coupling That Evades β-Oxygen Elimination. ACS Catal..

[21] Zhang Y., Hu B., Chen Y., Wang Z. (2024). Review on Catalytic Meinwald Rearrangement
of Epoxides. Chem. Eur. J..

[22] Bietti M., Gente G., Salamone M. (2005). Structural
Effects on the β-Scission
Reaction of Tertiary Arylcarbinyloxyl Radicals. The Role of α-Cyclopropyl
and α-Cyclobutyl Groups. J. Org. Chem..

[23] Li J., Wang H., Qiu Z., Huang C.-Y., Li C.-J. (2020). Metal-Free
Direct Deoxygenative Borylation of Aldehydes and Ketones. J. Am. Chem. Soc..

[24] Zhong P.-F., Tu J.-L., Zhao Y., Zhong N., Yang C., Guo L., Xia W. (2023). Photoelectrochemical oxidative C­(sp3)–H borylation
of unactivated hydrocarbons. Nat. Commun..

[25] Schreiber S.
L. (1980). Fragmentation reactions
of. alpha.-alkoxy
hydroperoxides and application to the synthesis of the macrolide (.+-.)-recifeiolide. J. Am. Chem. Soc..

[26] Burns M., Essafi S., Bame J. R., Bull S. P., Webster M. P., Balieu S., Dale J. W., Butts C. P., Harvey J. N., Aggarwal V. K. (2014). Assembly-line synthesis
of organic molecules with tailored
shapes. Nature.

[27] Nakamura Y., Mori K. (2000). New Syntheses of the Rice Moth and Stink Bug Pheromones by Employing
(2*R*, 6*S*)-7-Acetoxy-2,6-dimethyl-1-heptanol
as a Building Block. Biosci. Biotechnol. Biochem..

[28] Ferguson R. N., Whidby J. F., Sanders E. B., Levins R. J., Katz T., DeBardeleben J. F., Einolf W. N. (1978). Isolation and identification
of 2,3-dimethyl-6-(4,8,12-trimethyltridecyl)-1,4-naphthoquinone from
tobacco and smoke. Tetrahedron Lett..

[29] Fujioka T., Kashiwada Y., Kilkuskie R. E., Cosentino L. M., Ballas L. M., Jiang J. B., Janzen W. P., Chen I.-S., Lee K.-H. (1994). Anti-AIDS Agents,
11. Betulinic Acid and Platanic Acid as Anti-HIV Principles from Syzigium
claviflorum, and the Anti-HIV Activity of Structurally Related Triterpenoids. J. Nat. Prod..

[30] Santos R. C., Salvador J. A. R., Marín S., Cascante M., Moreira J. N., Dinis T. C. P. (2010). Synthesis and
structure–activity relationship study of novel cytotoxic carbamate
and N-acylheterocyclic bearing derivatives of betulin and betulinic
acid. Biorg. Med. Chem..

[31] Michaudel Q., Journot G., Regueiro-Ren A., Goswami A., Guo Z., Tully T. P., Zou L., Ramabhadran R. O., Houk K. N., Baran P. S. (2014). Improving Physical Properties via
C–H Oxidation: Chemical and Enzymatic Approaches. Angew. Chem., Int. Ed..

[32] Castellino N. J., Montgomery A. P., Danon J. J., Kassiou M. (2023). Late-stage Functionalization
for Improving Drug-like Molecular Properties. Chem. Rev..

[33] Sidova V., Zoufaly P., Pokorny J., Dzubak P., Hajduch M., Popa I., Urban M. (2017). Cytotoxic
conjugates of betulinic acid and substituted triazoles prepared by
Huisgen Cycloaddition from 30-azidoderivatives. PLoS One.

[34] Kahnt M., Heller L., Al-Harrasi A., Schäfer R., Kluge R., Wagner C., Otgonbayar C., Csuk R. (2018). Platanic acid-derived methyl 20-amino-30-norlupan-28-oates are potent
cytotoxic agents acting by apoptosis. Med. Chem.
Res..

[35] Armstrong R. J., Niwetmarin W., Aggarwal V. K. (2017). Synthesis of Functionalized Alkenes
by a Transition-Metal-Free Zweifel Coupling. Org. Lett..

[36] Bonet A., Odachowski M., Leonori D., Essafi S., Aggarwal V. K. (2014). Enantiospecific
sp2–sp3 coupling of secondary and tertiary boronic esters. Nat. Chem..

