# Supplementary material for: Iron-Catalyzed Dealkenylative Borylation of Alkenes via Ozonolysis
Source: J Am Chem Soc. 2026 Aug 5;148(32):34066–72. doi: 10.1021/jacs.6c10840 (PMC13495747; doi:10.1021/jacs.6c10840)
Supplement: Supplementary file 1 [file ja6c10840_si_001.pdf]

SUPPORTING INFORMATION

Iron–Catalyzed Dealkenylative Borylation of Alkenes via Ozonolysis

Kevin C. Wang[a], Emanuele Pinosa[a], Laura J. McCormick McPherson[b], Simon J. Coles[b], Jasper L. Tyler[a], Varinder K. Aggarwal[a]*

[a]*School of Chemistry, University of Bristol, Cantock's Close, Bristol BS8 1TS, United Kingdom*

*e-mail: v.aggarwal@bristol.ac.uk

[b]*School of Chemistry, University of Bristol, Cantock's Close, Bristol BS8 1TS, United Kingdom*

TABLE OF CONTENTS

LIST OF SUPPLEMENTARY SCHEMES, FIGURES AND TABLES 3

LIST OF CHARACTERISED PRODUCTS 3

1. MATERIALS AND GENERAL METHODS 5

1.1. Glassware, Solvents and Reagents 5

1.2. Chromatography and Instrumentation 5

1.3. Naming of Compounds 6

2. EXPERIMENTAL DATA 7

2.1. General Procedures 7

2.1.1. General Procedure A: Synthesis of alkene substrates for dealkenylative borylation 7

2.1.2. General Procedure B: Dealkenylative Borylation of Alkenes with Fe(II) 7

2.1.3. General Procedure C: Dealkenylative Borylation of Alkenes with Fe(III) 8

2.2. Reaction Optimization 8

2.2.1. Stoichiometric metal–mediated dealkenylative borylation: metal complex screening 8

2.2.2. Fe(II)–catalyzed dealkenylative borylation: catalyst loading and ligand screening 10

2.2.3. Fe(II)–catalyzed dealkenylative borylation: solvent screening 11

2.2.4. Fe(II)–catalyzed dealkenylative borylation: control experiments 11

2.2.5. Fe(III)–catalyzed dealkenylative borylation: Fe source, ligand, and additive screening 12

2.3. Synthesis of Alkene substrates 14

2.4. Substrate Scope 23

2.5 Comparison of Fe(II)/Fe(III) conditions with selected substrates 40

2.6. Failed Substrates 41

2.7. Benchmarking analysis against previous report of dealkenylative borylation 43

2.8. Synthesis of Rice Moth Pheromone 44

2.8.1 Literature synthesis of rice moth pheromone[18] 45

2.9. Synthesis of Solanoquinone 45

2.9.1 Literature synthesis of solanoquinone[22] 50

2.10. Synthetic diversifications on betulinic acid derivatives 51

3. MECHANISTIC STUDIES 56

3.1. Proposed mechanism for the Fe–catalyzed dealkenylative borylation 56

3.2. Proposed mechanism for the formation of side products 57

3.3. Radical trapping experiment with TEMPO 58

3.4. Fe(III) catalyst activation studies 58

3.4.1. ^{1}H NMR of the pre-stirring mixture using either $Fe(acac)_3$ or $FeCl_2$ 59

3.4.2. Independent synthesis of Bcat–acac 60

4. X-RAY CRYSTALLOGRAPHIC ANALYSIS 61

4.1. Compound 2p (CCDC 2555898) 61

5. SPECTROSCOPIC DATA 63

6. REFERENCES 111

LIST OF SUPPLEMENTARY SCHEMES, FIGURES AND TABLES

Table S1 Optimization of the stoichiometric metal-mediated dealkenylative borylation of 1a. 9

Figure S1. Representative GC-MS spectrum of the crude reaction mixture. 9

Figure S2. Attribution of each peak of the GC-MS spectrum in Figure S1... 10

Table S2. Optimization of metal catalyst loading and ligand for the Fe(II)–catalyzed dealkenylative borylation of 1a. 10

Table S3. Optimization of solvent and concentration for the Fe(II)–catalyzed dealkenylative borylation of 1a. 11

Table S4. Control experiments for the Fe(II)–catalyzed dealkenylative borylation of 1a. 12

Table S5. Optimization for Fe(III)–catalyzed dealkenylative borylation with substrate 1a. 12

Table S6. Optimization for Fe(III)–catalyzed dealkenylative borylation with substrate 1c. 13

Figure S3. Proposed catalytic cycle for the Fe–catalyzed dealkenylative borylation. 56

Figure S4. Proposed mechanism for the formation of ester/aldehyde side products. 57

Figure S5. Proposed mechanism for the formation of aldehyde/ketone side products. 57

Figure S6. Pre-stirring of the reaction mixture 58

Figure S7. ^{1}H NMR of the reaction mixture after pre-stirring 59

Figure S8. Independent synthesis of Bcat–acac and comparison of ^{11}B NMR 60

Figure S9. Compound 2p (CCDC 2555898) 61

Table S7 Crystal Data and structure refinement for compound 2p (CCDC 2555898) 62

LIST OF CHARACTERISED PRODUCTS

(3-methylbut-3-en-1-yl)benzene (1b) 14

prop-1-en-2-ylcyclohexane (1d) 14

tert-butyl 4-(prop-1-en-2-yl)piperidine-1-carboxylate (1e) 15

tert-butyldimethyl(((1*R*,2*S*,5*R*)-5-methyl-2-(prop-1-en-2-yl)cyclohexyl)oxy)silane (1h) 15

tert-butyldimethyl(((1*R*,2*R*,5*R*)-2-methyl-5-(prop-1-en-2-yl)cyclohexyl)oxy)silane (1j) 16

4-(2,2-difluorobenzo[*d*][1,3]dioxol-4-yl)-1-(pent-4-en-1-yl)-1*H*-pyrrole-3-carbonitrile (1n) 17

(8*S*,9*S*,10*R*,13*S*,14*S*,17*R*)-10,13-dimethyl-17-(prop-1-en-2-yl)-1,2,6,7,8,9,10,11,12,13,14,15,16,17-tetradecahydro-3*H*-cyclopenta[*a*]phenanthren-3-one (1o) 17

methyl (1*R*,3a*S*,5a*R*,5b*R*,7a*R*,9*S*,11a*R*,11b*R*,13a*R*,13b*R*)-9-hydroxy-5a,5b,8,8,11a-pentamethyl-1-(prop-1-en-2-yl)icosahydro-3a*H*-cyclopenta[*a*]chrysene-3a-carboxylate (1p) 19

methyl (1*R*,3a*S*,5a*R*,5b*R*,7a*R*,9*S*,11a*R*,11b*R*,13a*R*,13b*R*)-9-((tert-butyldimethylsilyl)oxy)-5a,5b,8,8,11a-pentamethyl-1-(prop-1-en-2-yl)icosahydro-3a*H*-cyclopenta[*a*]chrysene-3a-carboxylate (1q) 19

tert-butyl (1*R*,3a*S*,5a*R*,5b*R*,7a*R*,9*S*,11a*R*,11b*R*,13a*R*,13b*R*)-9-hydroxy-5a,5b,8,8,11a-pentamethyl-1-(prop-1-en-2-yl)icosahydro-3a*H*-cyclopenta[*a*]chrysene-3a-carboxylate (SI-4) 20

tert-butyl (1*R*,3a*S*,5a*R*,5b*R*,7a*R*,9*S*,11a*R*,11b*R*,13a*R*,13b*R*)-9-((tert-butyldimethylsilyl)oxy)-5a,5b,8,8,11a-pentamethyl-1-(prop-1-en-2-yl)icosahydro-3a*H*-cyclopenta[*a*]chrysene-3a-carboxylate (1r) 21

tert-butyl 4-methylenepiperidine-1-carboxylate (3b) 22

tert-butyl (1*R*,5*S*)-3-methylene-8-azabicyclo[3.2.1]octane-8-carboxylate (3d) 23

4,4,5,5-tetramethyl-2-phenethyl-1,3,2-dioxaborolane (2a) 23

2-cyclohexyl-4,4,5,5-tetramethyl-1,3,2-dioxaborolane (2c) 24

tert-butyl 4-(4,4,5,5-tetramethyl-1,3,2-dioxaborolan-2-yl)piperidine-1-carboxylate (2e) 25

(2*R*)-2-methyl-5-(4,4,5,5-tetramethyl-1,3,2-dioxaborolan-2-yl)cyclohexan-1-one (2f) 25

(1*R*,2*R*,5*R*)-5-methyl-2-(4,4,5,5-tetramethyl-1,3,2-dioxaborolan-2-yl)cyclohexan-1-ol (2g) 26

tert-butyldimethyl(((1*R*,2*R*,5*R*)-5-methyl-2-(4,4,5,5-tetramethyl-1,3,2-dioxaborolan-2-yl)cyclohexyl)oxy)silane (2h) 26

(1*S*)-1-methyl-3-(4,4,5,5-tetramethyl-1,3,2-dioxaborolan-2-yl)cyclopentane-1-carbaldehyde (2i) 27

tert-butyldimethyl(((1*R*,2*R*,5*R*)-2-methyl-5-(4,4,5,5-tetramethyl-1,3,2-dioxaborolan-2-yl)cyclohexyl)oxy)silane (2j) 28

(4*R*,4a*S*,6*R*)-4,4a-dimethyl-6-(4,4,5,5-tetramethyl-1,3,2-dioxaborolan-2-yl)-4,4a,5,6,7,8-hexahydronaphthalen-2(3*H*)-one (2k) 29

4,4,5,5-tetramethyl-2-((4*S*,8*R*)-4,8,12-trimethyltridecyl)-1,3,2-dioxaborolane (2l) 29

4,4,5,5-tetramethyl-2-(2-((1*r*,1'*r*,4*R*,4'*R*)-4'-(*p*-tolyl)-[1,1'-bi(cyclohexan)]-4-yl)ethyl)-1,3,2-dioxaborolane (2m) 30

4-(2,2-difluorobenzo[*d*][1,3]dioxol-4-yl)-1-(3-(4,4,5,5-tetramethyl-1,3,2-dioxaborolan-2-yl)propyl)-1*H*-pyrrole-3-carbonitrile (2n) 31

(8*S*,9*S*,10*R*,13*R*,14*S*,17*S*)-10,13-dimethyl-17-(4,4,5,5-tetramethyl-1,3,2-dioxaborolan-2-yl)-1,2,6,7,8,9,10,11,12,13,14,15,16,17-tetradecahydro-3*H*-cyclopenta[*a*]phenanthren-3-one (2o) 32

methyl (1*R*,3a*S*,5a*R*,5b*R*,7a*R*,9*S*,11a*R*,11b*R*,13a*R*,13b*R*)-9-hydroxy-5a,5b,8,8,11a-pentamethyl-1-(4,4,5,5-tetramethyl-1,3,2-dioxaborolan-2-yl)icosahydro-3a*H*-cyclopenta[*a*]chrysene-3a-carboxylate (2p) 33

methyl (1*R*,3a*S*,5a*R*,5b*R*,7a*R*,9*S*,11a*R*,11b*R*,13a*R*,13b*R*)-9-((*tert*-butyldimethylsilyl)oxy)-5a,5b,8,8,11a-pentamethyl-1-(4,4,5,5-tetramethyl-1,3,2-dioxaborolan-2-yl)icosahydro-3a*H*-cyclopenta[*a*]chrysene-3a-carboxylate (2q) 34

tert-butyl (1*R*,3a*S*,5a*R*,5b*R*,7a*R*,9*S*,11a*R*,11b*R*,13a*R*,13b*R*)-9-((*tert*-butyldimethylsilyl)oxy)-5a,5b,8,8,11a-pentamethyl-1-(4,4,5,5-tetramethyl-1,3,2-dioxaborolan-2-yl)icosahydro-3a*H*-cyclopenta[*a*]chrysene-3a-carboxylate (2r) 35

methyl 6-(4,4,5,5-tetramethyl-1,3,2-dioxaborolan-2-yl)hexanoate (4a) 36

methyl 3-((*tert*-butoxycarbonyl)(2-(4,4,5,5-tetramethyl-1,3,2-dioxaborolan-2-yl)ethyl)amino)propanoate (4b). 36

methyl (1*R*,3*S*)-2,2-dimethyl-3-(2-(4,4,5,5-tetramethyl-1,3,2-dioxaborolan-2-yl)ethyl)cyclobutane-1-carboxylate (4ca) and methyl 3-((1*S*,3*R*)-2,2-dimethyl-3-(4,4,5,5-tetramethyl-1,3,2-dioxaborolan-2-yl)cyclobutyl)propanoate (4cb) 37

tert-butyl 2-(2-methoxy-2-oxoethyl)-5-((4,4,5,5-tetramethyl-1,3,2-dioxaborolan-2-yl)methyl)pyrrolidine-1-carboxylate (4d) 38

methyl (1*R*,2*S*)-2-isopropyl-2-(2-(4,4,5,5-tetramethyl-1,3,2-dioxaborolan-2-yl)ethyl)cyclopropane-1-carboxylate (4e) 38

1,5-bis(4,4,5,5-tetramethyl-1,3,2-dioxaborolan-2-yl)pentan-1-ol (4f) 41

(2*R*,6*R*,10*R*)-6,10,14-trimethylpentadecan-2-ol (5) 44

2,3-dimethylnaphthalene-1,4-dione (6) 45

1,4-dimethoxy-2,3-dimethylnaphthalene (7) 46

5,8-dimethoxy-6,7-dimethylnaphthalen-2-ol (8) 47

5,8-dimethoxy-6,7-dimethylnaphthalen-2-yl trifluoromethanesulfonate (9) 48

2,3-dimethyl-6-((4*R*,8*R*)-4,8,12-trimethyltridecyl)naphthalene-1,4-dione (10) 48

tert-butyl (1*S*,3a*S*,5a*R*,5b*R*,7a*R*,9*S*,11a*R*,11b*R*,13a*R*,13b*R*)-9-((tert-butyldimethylsilyl)oxy)-5a,5b,8,8,11a-pentamethyl-1-vinylicosahydro-3a*H*-cyclopenta[*a*]chrysene-3a-carboxylate (11) 51

tert-butyl (1*R*,3a*S*,5a*R*,5b*R*,7a*R*,9*S*,11a*R*,11b*R*,13a*R*,13b*R*)-9-((*tert*-butyldimethylsilyl)oxy)-5a,5b,8,8,11a-pentamethyl-1-(1-phenylvinyl)icosahydro-3a*H*-cyclopenta[*a*]chrysene-3a-carboxylate (12) 52

tert-butyl (1*R*,3a*S*,5a*R*,5b*R*,7a*R*,9*S*,11a*R*,11b*R*,13a*R*,13b*S*)-9-((*tert*-butyldimethylsilyl)oxy)-1-(3-fluoropyridin-4-yl)-5a,5b,8,8,11a-pentamethylicosahydro-3a*H*-cyclopenta[*a*]chrysene-3a-carboxylate (13) 53

tert-butyl (1*R*,3a*S*,5a*R*,5b*R*,7a*R*,9*S*,11a*R*,11b*R*,13a*R*,13b*S*)-9-((*tert*-butyldimethylsilyl)oxy)-1-(furan-2-yl)-5a,5b,8,8,11a-pentamethylicosahydro-3a*H*-cyclopenta[*a*]chrysene-3a-carboxylate (14) 54

1. MATERIALS AND GENERAL METHODS

1.1. Glassware, Solvents and Reagents

All manipulations were performed with oven-dried (130 °C for a minimum of 12 h) or flame-dried glassware using standard Schlenk techniques under an atmosphere of nitrogen, unless otherwise stated. Air and moisture sensitive liquids and solutions were transferred via syringe into the reaction vessels through a rubber septum. Temperatures described below –10 °C were achieved using Thermo Scientific EK-90 or Huber TC100E cryostats or appropriate cooling baths.

All anhydrous solvents were commercially supplied or dried using an Anhydrous Engineering alumina column drying system (THF, PhMe, Et_2O, CH_2Cl_2). Reagents were purchased from commercial sources and used as received. Exceptions: *N*,*N*,*N′*,*N′*-tetramethylethylenediamine (TMEDA) was distilled over CaH_2 under an inert atmosphere at standard pressure. All organolithium reagents were titrated against *N*-benzylbenzamide.[1]

1.2. Chromatography and Instrumentation

Thin layer chromatography (TLC) was performed using Merck Kieselgel 60 F254 fluorescent treated silica, which was visualized under UV light, or by staining with either aqueous basic potassium permanganate ($KMnO_4$), *p*-anisaldehyde solution, Hanessian's stain (CAM), or an ethanolic solution of phosphomolybdic acid (PMA) followed by heating.

Preparative thin layer chromatography was performed using Merck Z513032- 1PAK TLC plates (silica gel) and visualized under UV light.

Flash column chromatography (FCC) was carried out using silica gel (60 Å, 230–400 mesh, 40–63 μm) purchased from VWR or a Biotage Isolera™ flash purification system. Boric acid treated silica was synthesized according to a literature method.[2]

NMR spectra were recorded at various field strengths, as indicated, using Bruker 400 MHz, Varian VNMR 400 MHz, Bruker Prodigy 500 MHz, or Bruker Cryo 500 MHz for ^{1}H, ^{11}B, ^{13}C and ^{19}F acquisitions. All NMR spectra were recorder at 25 °C unless otherwise stated. Chemical shifts (δ) are reported in parts per million (ppm) and referenced to $CDCl_3$ (^{1}H: 7.26 ppm; ^{13}C: 77.16 ppm). Coupling constants (*J*) are given in Hertz (Hz) and refer to apparent multiplicities (s = singlet, d = doublet, t = triplet, q = quartet, quin = quintet, hex = sextet, h = heptet, m = multiplet, br = broad signal, dd = doublet of doublets, etc.). The ^{1}H NMR spectra are reported as follows: chemical shift (multiplicity, coupling constants, number of protons).

NMR yields: Following work-up, 1,2-dibromomethane or 1,3,5-trimethylbenzene (1.0 equiv relative to limiting reagent) was added to the crude residue. The resultant mixture was dissolved in $CDCl_3$ (1.0 mL), and a 0.5 mL sample of the solution was taken for ^{1}H NMR analysis. Yields were calculated based on the integrals of known product resonances relative to the internal standard.

High-resolution mass spectrometry (HRMS) was recorded on a Bruker Daltonics MicrOTOF II by Electrospray Ionisation (ESI); a Thermo Scientific QExactive by Electron Ionisation (EI); a Thermo Scientific Orbitrap Elite by ESI or Atmospheric Pressure Chemical Ionisation (APCI); or a Bruker UltrafleXtreme by Matrix-assisted Laser Desorption/Ionisation (MALDI).

IR spectra were recorded neat as a thin film on a Perkin Elmer Spectrum One FT-IR. Selected absorption maxima (ν_{max}) are reported in wavenumbers (cm^{-1}).

Ozone generator: Ozonolysis experiments were conducted using a AirTree Europe C-Lasky C-L010DT ozone generator (10 g/h @ oxygen fed 6 L/min), with 70% of output level and 1 L/min oxygen flow rate for 5 min or as indicated by a blue color in the reaction mixture as stated in the experimental.

Note: Ozone is potentially harmful to the human body (8-h TWA limit of 1 ppm and 15-min STEL of 0.3 ppm) and is an oxidant that can react with certain compounds to form explosive and shock-sensitive products. Although we have not encountered any ozone-related safety issues in our lab, reactions with ozone should be performed only by properly trained individuals in a well-ventilated fume hood (use of a blast-shield is also recommended, especially for reactions performed on larger scales).

1.3. Naming of Compounds

Compound names are those generated by ChemDraw Professional 20.0 software (PerkinElmer), following the IUPAC nomenclature.

2. EXPERIMENTAL DATA

2.1. General Procedures

2.1.1. General Procedure A: Synthesis of alkene substrates for dealkenylative borylation

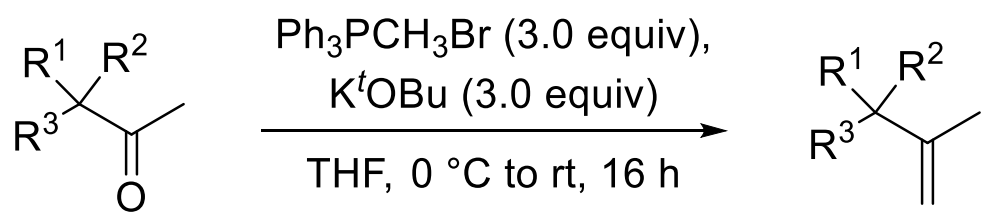

A flame-dried 50 mL RBF was charged with a magnetic stirrer bar and methyltriphenylphosphonium bromide (5.5 g, 15 mmol, 3.0 equiv) followed by the addition of THF (25 mL, 0.2 M). The mixture was then cooled to 0 °C followed by addition of potassium *tert*-butoxide (1.7 g, 15 mmol, 3.0 equiv). The yellow suspension was allowed to warm up to room temperature and stirred for 1 h before cooling down to 0 °C again followed by dropwise addition of the carbonyl substrate (5.0 mmol, 1.0 equiv). The reaction mixture was then allowed to stir at room temperature overnight. After completion, as indicated by TLC analysis, the mixture was quenched with H_2O (25 mL) and extracted with Et_2O (3 × 25 mL). The combined organic layers were dried over anhydrous $MgSO_4$ and concentrated under reduced pressure followed by silica gel flash column chromatography with pentane/EtOAc to afford the desired alkene substrate.

2.1.2. General Procedure B: Dealkenylative Borylation of Alkenes with Fe(II)

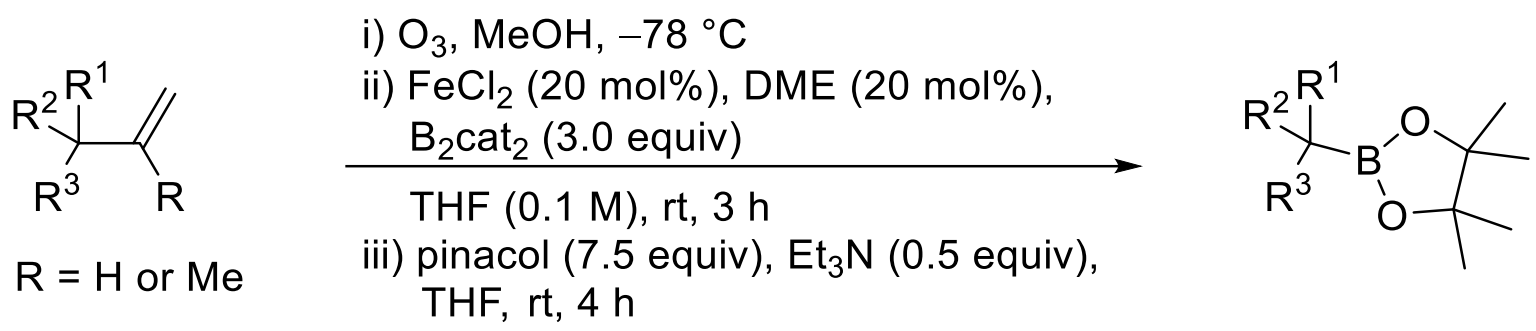

A flame-dried 8 mL Biotage® microwave reaction vial equipped with a magnetic stirrer bar was charged with $FeCl_2$ (5.1 mg, 40 μmol, 0.20 equiv) and B_2cat_2 (143 mg, 0.600 mmol, 3.00 equiv) inside an argon-filled glovebox. The vial was sealed with a cap with a septum, taken outside of the glovebox, followed by addition of anhydrous THF (1 mL) and anhydrous dimethoxyethane (4.2 μL, 40 μmol, 0.2 equiv). The mixture was allowed to stir for 1 h at room temperature to give a *Suspension A*. Another flame-dried 25 mL RBF was charged with the alkene substrate (0.20 mmol, 1.0 equiv) and MeOH (4 mL) and cooled to –78 °C in a dry ice/acetone bath. Ozone was bubbled through the solution until complete consumption of the starting material (as indicated by a blue color in the reaction mixture). The solution was sparged with N_2 for 5 min to expel excess ozone and the reaction mixture was warmed up to room temperature before MeOH was removed under reduced pressure. The reaction mixture was redissolved in anhydrous THF (4 mL), and the solvent was removed under reduced pressure again to ensure removal of MeOH. Note: Any remaining MeOH will react with B_2cat_2 and reduce the efficiency of the desired reaction. The residue was redissolved in anhydrous THF (0.8 mL) and transferred into *Suspension A* via a 1-mL syringe, with an additional rinse of THF (0.2 mL) used to wash the remaining residue into *Suspension A*. The reaction vial was allowed to stir for 3 h at room temperature followed by the addition of a solution of pinacol (177 mg, 1.50 mmol, 7.50 equiv) and Et_3N (14 μL, 0.10 mmol, 0.50 equiv) in THF (2 mL). The reaction was allowed to stir for 4 h at room temperature and the crude mixture was filtered through a short pad of silica,

washed with Et_2O (10 mL) and purified via silica gel flash column chromatography with pentane/EtOAc to afford the desired pinacol boronic ester.

2.1.3. General Procedure C: Dealkenylative Borylation of Alkenes with Fe(III)

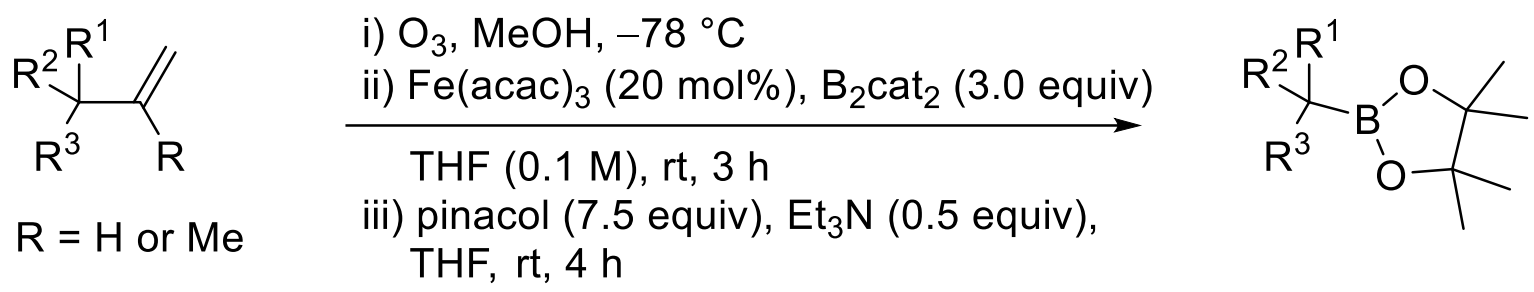

A flame-dried 8 mL Biotage® microwave reaction vial equipped with a magnetic stirrer bar was charged with $Fe(acac)_3$ (14 mg, 40 μmol, 0.20 equiv) and B_2cat_2 (143 mg, 0.600 mmol, 3.00 equiv) under ambient atmosphere. The vial was sealed with a cap with a septum, back filled with N_2 three times, followed by addition of anhydrous THF (1 mL). The mixture was allowed to stir for 20 min at room temperature to give a *Suspension B*. (Note: a color change from red brown to yellow would be observed within 10 min). Another flame-dried 25 mL RBF was charged with the alkene (0.20 mmol, 1.0 equiv) and MeOH (4 mL) and cooled to –78 °C in a dry ice/acetone bath. Ozone was bubbled through the solution until complete consumption of the starting material (as indicated by a blue color in the reaction mixture). The solution was sparged with N_2 for 5 min to expel excess ozone and the reaction mixture was warmed up to room temperature before MeOH was removed under reduced pressure. The reaction mixture was redissolved in anhydrous THF (4 mL), and the solvent was removed under reduced pressure again to ensure removal of MeOH. Note: Any remaining MeOH will react with B_2cat_2 and reduce the efficiency of the desired reaction. The residue was redissolved in anhydrous THF (0.8 mL) and transferred into *Suspension B* via a 1-mL syringe, with an additional rinse of THF (0.2 mL) used to wash the remaining residue into *Suspension B*. The reaction vial was allowed to stir for 3 h at room temperature followed by the addition of a solution of pinacol (177 mg, 1.50 mmol, 7.50 equiv) and Et_3N (14 μL, 0.10 mmol, 0.50 equiv) in THF (2 mL). The reaction was allowed to stir for 4 h at room temperature and the crude mixture was filtered through a short pad of silica, washed with Et_2O (10 mL) and purified via silica gel flash column chromatography with pentane/EtOAc to afford the desired pinacol boronic ester.

2.2. Reaction Optimization

2.2.1. Stoichiometric metal–mediated dealkenylative borylation: metal complex screening

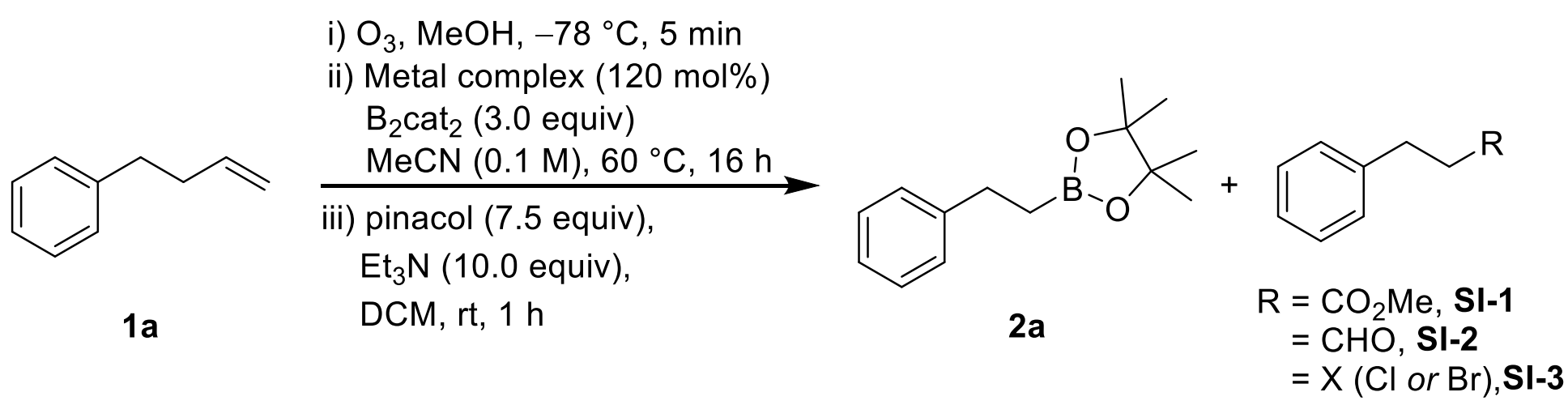

Entry[a,b]	Metal complex	% yield of **2a**	% yield of **SI-1**	% yield of **SI-2**	% yield of **SI-3**	Mass Balance
1	$Fe(SO_4)•7H_2O$	4	0	19	/	23
2	$Fe(OAc)_2$	24	16	0	/	39
3	$FeCl_2•4H_2O$	26	13	0	10	49
4	$FeCl_2$	26	12	0	10	48
5	$FeBr_2$	4	12	0	36	52
6	$(NH_4)_2Fe(SO_4)_2•6H_2O$	5	1	0	/	6
7	$Fe(BF_4)•6H_2O$	9	3	0	/	12
8	$Fe(acac)_2$	23	20	0	/	43
9	CuCl	8	14	0	10	32
10	CuBr	2	21	0	16	39
11	CuI	2	11	3	3	19
12	CuOAc	8	15	4	/	27
13	CuTc	6	9	0	/	15

Table S1 Optimization of the stoichiometric metal-mediated dealkenylative borylation of 1a.

[a] Reactions performed on 0.2 mmol scale. [b] Yields were determined by quantitative GC-MS analysis using 1,3,5-trimethylbenzene as the internal standard.

Initial optimization studies were performed with stoichiometric amounts of metal catalyst. Under these conditions, the desired product could only be obtained in low yield due to the competing formation of side products. From GC-MS and ^{1}H NMR analysis of the crude mixture, three main side products were identified, namely, methyl ester **SI-1**, aldehyde **SI-2** and alkyl halide **SI-3**. A typical GC-MS spectrum of the crude mixture is shown below in **Figure S1**, with the structures of the various components shown in **Figure S2**.

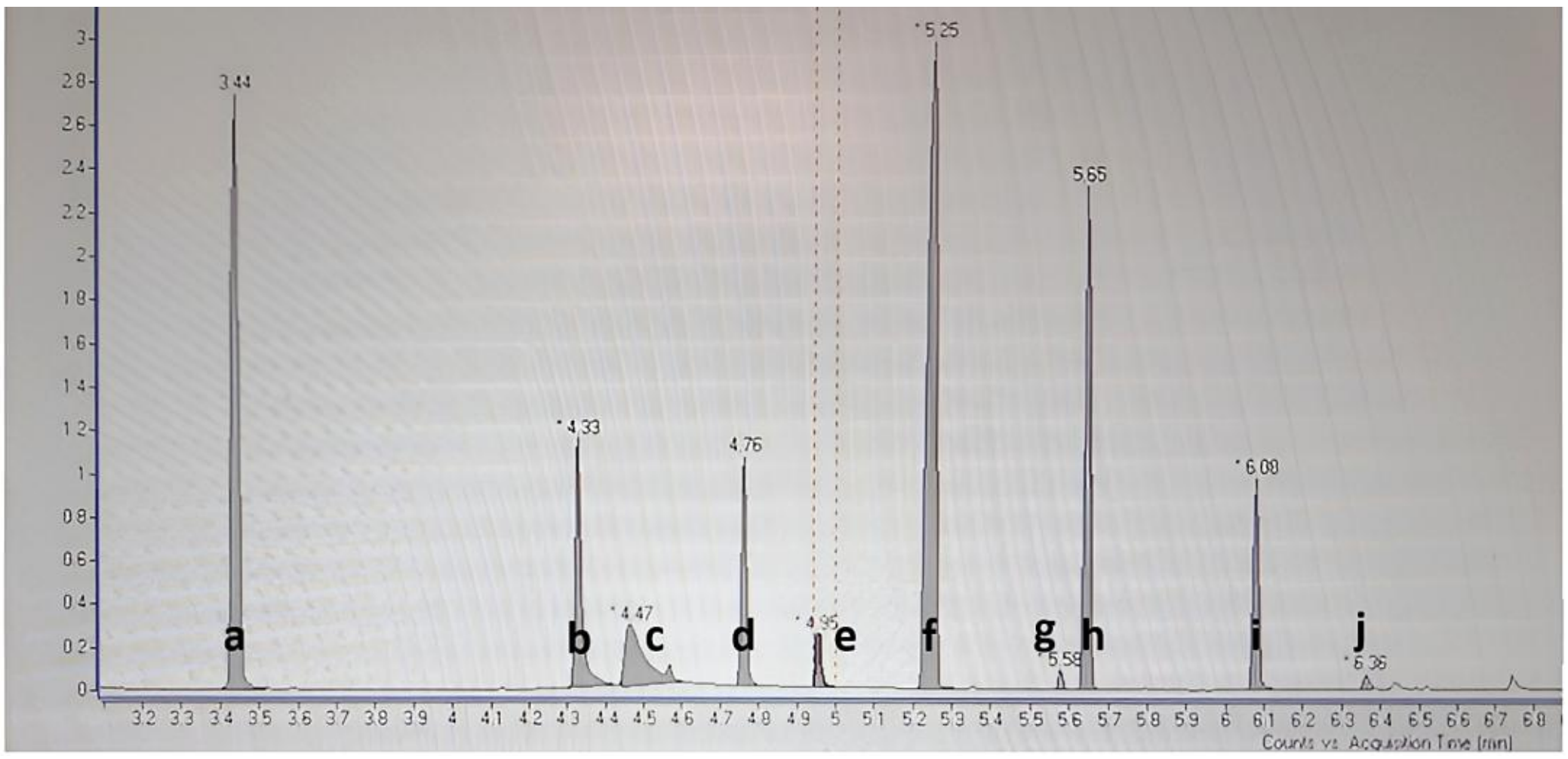

Figure S1. Representative GC-MS spectrum of the crude reaction mixture.

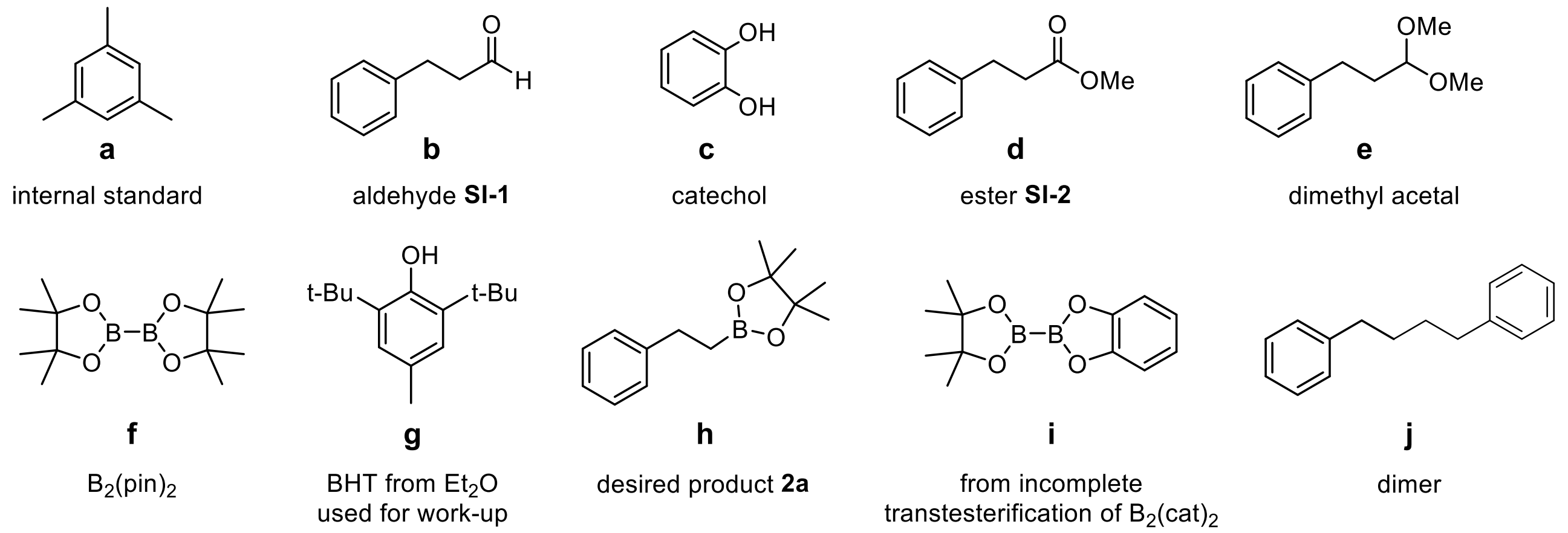

Figure S2. Attribution of each peak of the GC-MS spectrum in Figure S1.

2.2.2. Fe(II)–catalyzed dealkenylative borylation: catalyst loading and ligand screening

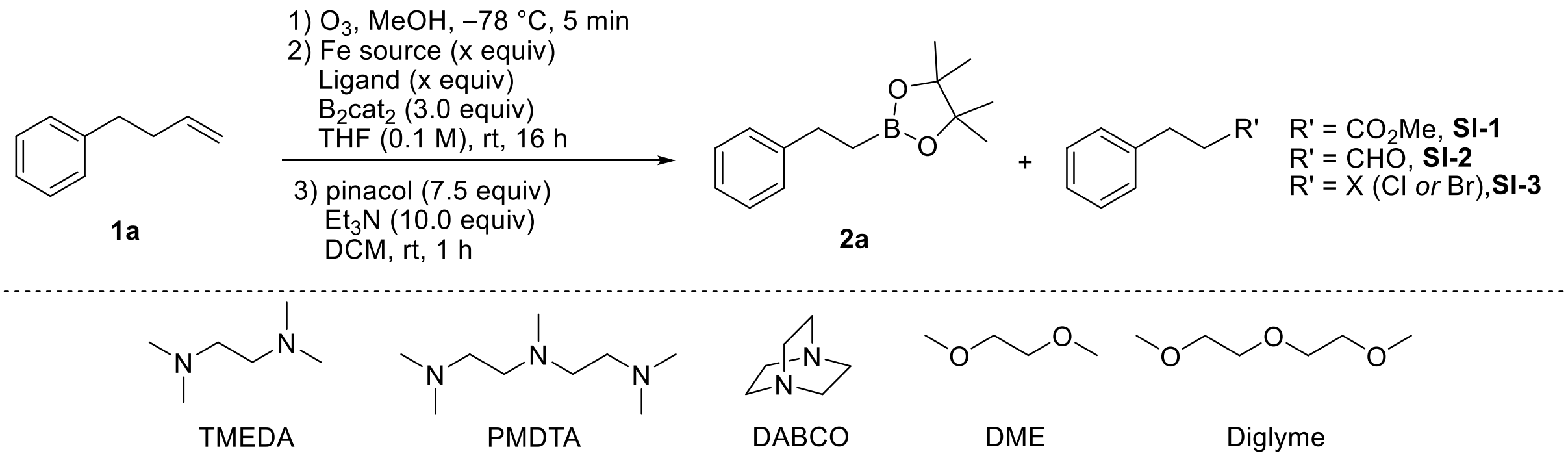

Entry[a,b]	Fe source (equiv)	Ligand (equiv)	% yield of **2a**	% yield of **SI-1**	% yield of **SI-2**	Mass Balance[c]
1[d]	$FeCl_2$ (0.2)	TMEDA (0.2)	34	19	12	65
2	$FeCl_2$ (0.2)	TMEDA (0.2)	41	19	29	89
3	$FeCl_2$ (0.2)	TMEDA (0.4)	27	12	trace	39
4	$FeCl_2$ (0.2)	PMDTA (0.2)	38	17	19	74
5	$FeCl_2$ (0.2)	pyridine (0.4)	22	11	57	90
6	$FeCl_2$ (0.2)	DABCO (0.2)	23	11	trace	34
7	$FeCl_2$ (0.2)	DME (0.2)	49 (45)	20	14	83
8[e]	$FeCl_2$ (0.2)	DME (0.2)	48	19	10	77
9[f]	$FeCl_2$ (0.2)	DME (0.2)	46	18	21	85
10	$FeCl_2$ (0.2)	Diglyme (0.2)	51	25	19	95
11	$FeCl_2$ (0.1)	DME (0.1)	43	20	21	84
12	$FeCl_2$ (1.2)	DME (1.2)	47	28	7	82
13	$FeCl_3$ (0.2)	DME (0.2)	41	16	14	71

Table S2. Optimization of metal catalyst loading and ligand for the Fe(II)–catalyzed dealkenylative borylation of 1a.

[a] Reactions performed on 0.2 mmol scale. [b] Yields were determined by quantitative GC-MS analysis using 1,3,5-trimethylbenzene as the internal standard. Isolated yields were reported in parentheses. [c] Trace amount of halide **SI-3** was detected in all the reactions performed. [d] Azeotropic removal of MeOH with THF was not

performed before the addition of the peroxy intermediate to the Fe(II)/B_2cat_2/ligand solution. [e] Reaction performed at 40 °C. [f] 3 h reaction time for the borylation step.

Using a catalytic amount of $FeCl_2$ in the presence of DME or diglyme as the ligand, the product can be obtained in higher yields with better mass balance. DME was chosen as optimal since anhydrous DME is commercially available while diglyme requires distillation prior to use. Thorough removal of methanol through azeotropic mixture with anhydrous THF improved both the yield and the mass balance since methanol is known to react with B_2cat_2. The reaction goes to completion within 3 h. However, during the optimization studies process a reaction time of 16 h was used for convenience.

2.2.3. Fe(II)–catalyzed dealkenylative borylation: solvent screening

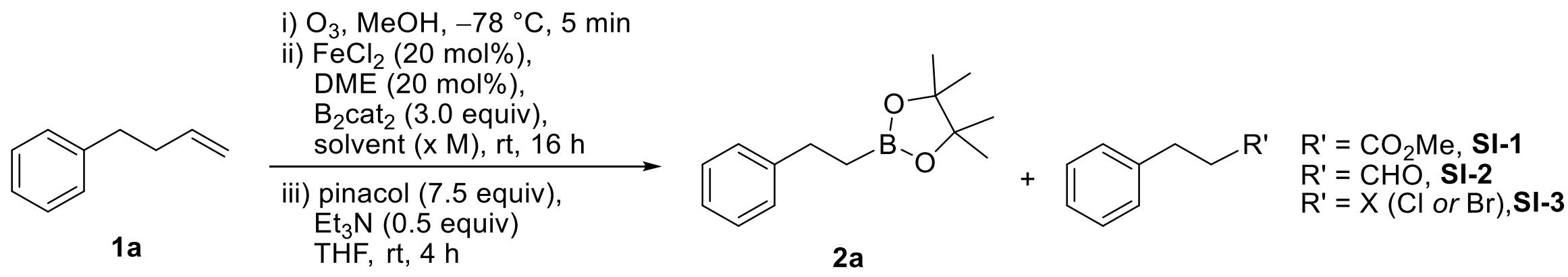

Entry[a,b]	Sovlent (x M)	% yield of **2a**	% yield of **SI-1**	% yield of **SI-2**	Mass Balance[c]
1	THF (0.1 M)	49 (45)	20	14	83
2	THF (0.2 M)	36	17	17	70
3	THF (0.05 M)	42	17	16	75
4	DME (0.1 M)	37	18	23	78
5	DMSO (0.1 M)	0	35	56	91
6	DMA (0.1 M)	9	3	57	69
7	MeCN (0.1 M)	14	5	40	59
8	DMF (0.1 M)	7	6	72	85
9	PhMe (0.1 M)	9	4	44	57
10	DCM (0.1 M)	27	14	trace	41
11	TBME (0.1 M)	19	9	64	92
12	2-MeTHF (0.2 M)	29	17	49	95

Table S3. Optimization of solvent and concentration for the Fe(II)–catalyzed dealkenylative borylation of 1a.

[a] Reactions performed on 0.2 mmol scale. [b] Yields were determined by quantitative GC-MS analysis using 1,3,5-trimethylbenzene as the internal standard. Isolated yields were reported in parentheses. [c] Trace amount of **SI-3** was detected in all the reactions performed.

2.2.4. Fe(II)–catalyzed dealkenylative borylation: control experiments

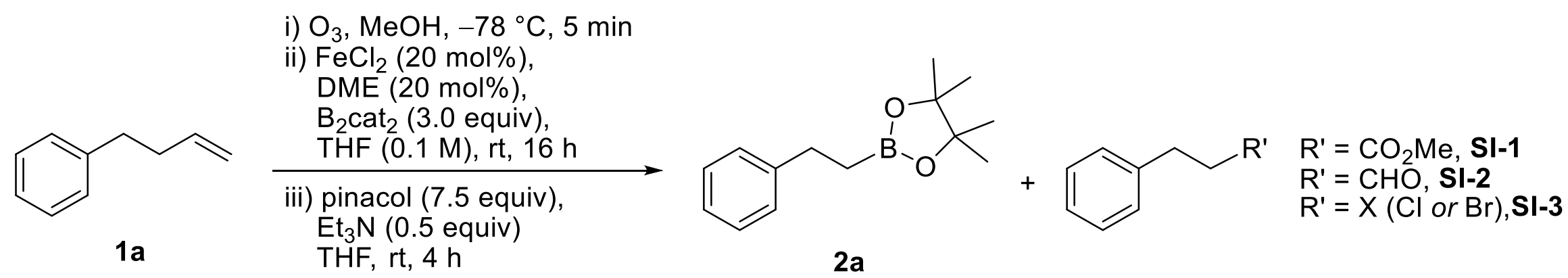

Entry[a,b]	Deviation from standard conditions	% yield of **2a**	% yield of **SI-1**	% yield of **SI-2**	Mass Balance[c]
1	None	49 (45)	20	14	83
2	No $FeCl_2$	12	traces	14	26
3	No DME	43	19	14	76
4	No $FeCl_2$ and DME	7	5	40	52
5	No pre-stirring	22	15	17	54
6	Pre-stirring without B_2cat_2	46	17	17	80
7	B_2pin_2 with NaOMe instead of B_2cat_2	9	7	0	16

Table S4. Control experiments for the Fe(II)–catalyzed dealkenylative borylation of 1a.

[a] Reactions performed on 0.2 mmol scale. [b] Yields were determined by quantitative GC-MS analysis using 1,3,5-trimethylbenzene as the internal standard. Isolated yields were reported in parentheses. [c] Trace amount of **SI-3** was detected in all reactions performed.

Note: Pre-stirring proved to be essential in achieving optimum yield (Entry 5) as $FeCl_2$ was observed to gradually dissolve in the THF upon stirring. The yield dropped marginally when B_2cat_2 was not included in the pre-stirred mixture and added in one proportion prior to the addition of the solution of the peroxy acetal (Entry 6). Therefore, it was anticipated that Fe(II) formed soluble complex with DME ligand in THF and led to optimal reactivity.

2.2.5. Fe(III)–catalyzed dealkenylative borylation: Fe source, ligand, and additive screening

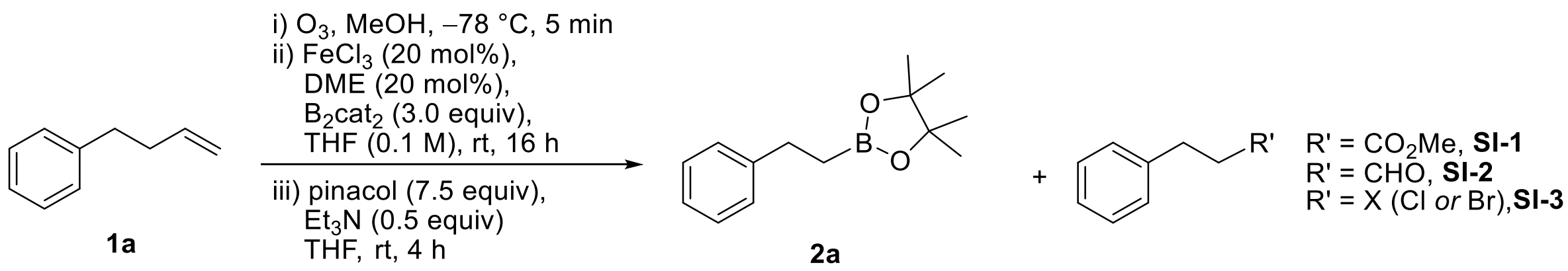

Entry[a,b]	Deviation from standard conditions	% yield of **2a**	% yield of **SI-1**	% yield of **SI-2**	Mass Balance[c]
1	None	41	16	14	71
2	20% of diglyme instead of DME	40	18	17	75
3	40% of $FeCl_3$ and DME	38	18	14	70
4	10% of $FeCl_3$ and DME	28	26	17	71
5	No DME	46 (43)	15	12	73
6	20% of $Fe(acac)_3$ instead of $FeCl_3$	43	18	21	82
7	B_2pin_2 with NaOMe instead of B_2cat_2	trace	n.d.	n.d.	N/A
8	2.0 equiv of B_2cat_2 instead of 3.0 equiv	30	24	19	73

Table S5. Optimization for Fe(III)–catalyzed dealkenylative borylation with substrate 1a.

[a] Reactions performed on 0.2 mmol scale. [b] Yields were determined by quantitative GC-MS analysis using 1,3,5-trimethylbenzene as the internal standard. Isolated yields were reported in parentheses. [c] Trace amount of **SI-3** was detected in all reactions in which $FeCl_3$ was employed.

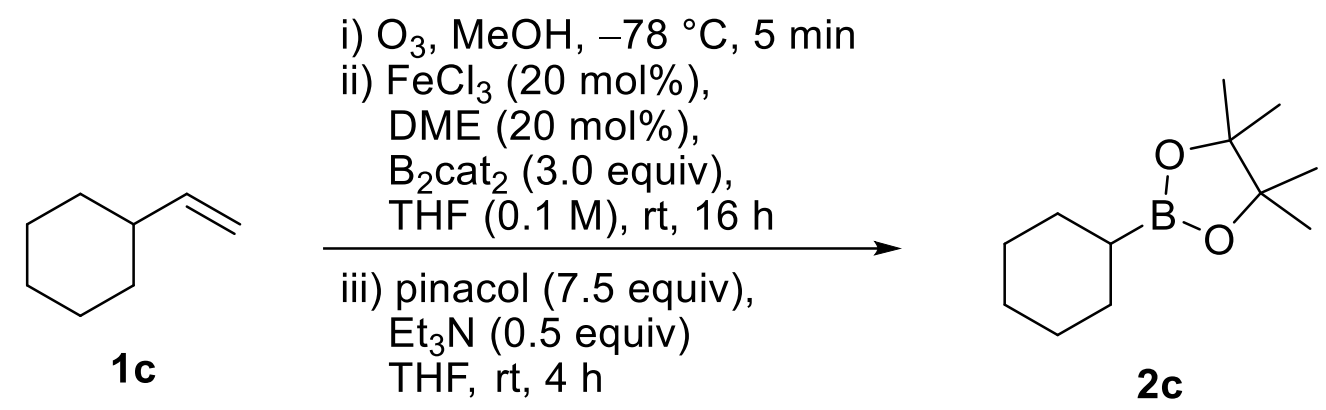

Entry[a,b]	Deviation from standard conditions	% yield of **2c**
1	None	35
2	20% of diglyme instead of DME	34
3	40% of $FeCl_3$ and DME	15
4	10% of $FeCl_3$ and DME	29
5	No DME	35
6	20% of $Fe(acac)_3$ instead of $FeCl_3$	45 (40)
7	B_2pin_2 with NaOMe instead of B_2cat_2	trace
8	2.0 equiv of B_2cat_2 instead of 3.0 equiv	23

Table S6. Optimization for Fe(III)–catalyzed dealkenylative borylation with substrate 1c.

[a] Reactions performed on 0.2 mmol scale. [b] Yields determined by quantitative GC-MS analysis using 1,3,5-trimethylbenzene as the internal standard. Isolated yields were reported in parentheses.

2.3. Synthesis of Alkene substrates

(3-methylbut-3-en-1-yl)benzene (1b)

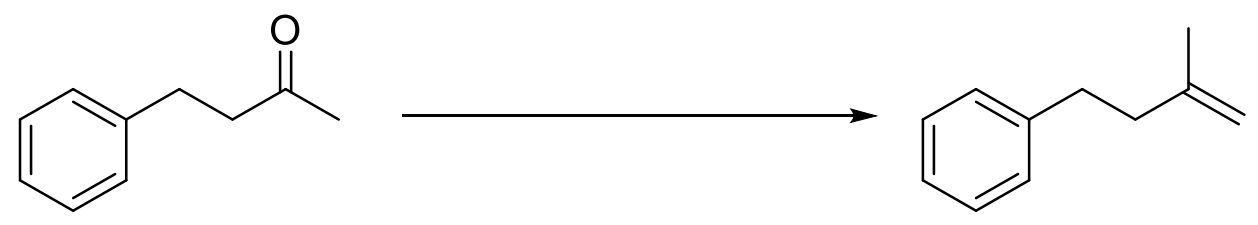

1b

Prepared following **General Procedure A** with 4-phenylbutan-2-one (0.74 g, 5.0 mmol, 1.0 equiv). The crude was purified by flash column chromatography (SiO_2; pentane) to afford the desired alkene substrate **1b** (0.54 g, 3.7 mmol, 74%) as a colorless oil.

TLC: R_f = 0.92 (20:1 pentane:EtOAc, $KMnO_4$).

NMR Spectroscopy (see spectra):

^{1}H NMR: (400 MHz, $CDCl_3$) δ 7.33–7.24 (m, 2H, 2 × Ar*H*), 7.23–7.14 (m, 3H, 3 ×Ar*H*), 4.75 (s, 1H, $CH_2C(CH_3)$=C*H*$_2$), 4.72 (s, 1H, $CH_2C(CH_3)$=C*H*$_2$), 2.76 (t, *J* = 8.0 Hz, 2H, C*H*$_2$), 2.33 (t, *J* = 8.0 Hz, 2H, C*H*$_2$), 1.78 (s, 3H, CH_2C(C*H*$_3$)=CH_2) ppm.

^{13}C NMR: (101 MHz, $CDCl_3$) δ 145.5 (CH_2*C*(CH_3)=CH_2), 142.4, 128.5, 128.4, 125.9, 110.3 ($CH_2C(CH_3)$=*C*H$_2$), 39.8 (Ar–*C*H$_2$), 34.4 (*C*H$_2$), 22.8 (CH_2C(*C*H$_3$)=CH_2) ppm.

All recorded spectroscopic data matched those previously reported in the literature.[3]

prop-1-en-2-ylcyclohexane (1d)

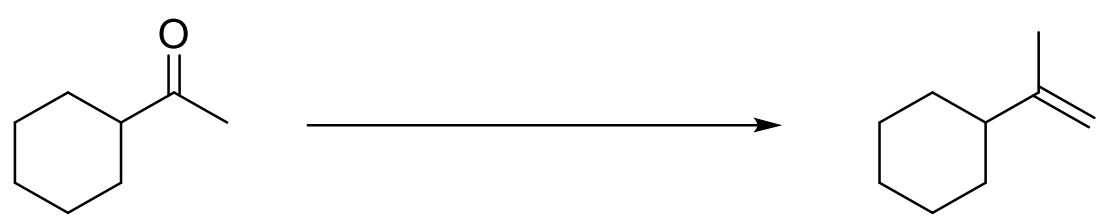

1d

Prepared following **General Procedure A** with 1-cyclohexylethan-1-one (0.63 g, 5.0 mmol, 1.0 equiv). The crude was purified by flash column chromatography (SiO_2; pentane) to afford the desired alkene substrate **1d** (0.51 g, 4.1 mmol, 82%) as a colorless oil.

TLC: R_f = 0.95 (20:1 pentane:EtOAc, $KMnO_4$).

NMR Spectroscopy (see spectra):

^{1}H NMR: (400 MHz, $CDCl_3$) δ 4.66 (s, 2H, CHC(CH_3)=C*H*$_2$), 1.86 (tt, *J* = 11.2, 3.4 Hz, 1H, C*H*C(CH_3)=CH_2), 1.80–1.63 (m, 5H), 1.71 (s, 3H, CHC(C*H*$_3$)=CH_2), 1.34–1.08 (m, 5H) ppm.

^{13}C NMR: (101 MHz, $CDCl_3$) δ 151.5 (CH*C*(CH_3)=CH_2), 108.0 (CHC(CH_3)=*C*H$_2$), 45.7 (*C*HC(CH_3)=CH_2), 32.1, 26.8, 26.5, 21.1 (CHC(*C*H$_3$)=CH_2) ppm.

All recorded spectroscopic data matched those previously reported in the literature.[4]

tert-butyl 4-(prop-1-en-2-yl)piperidine-1-carboxylate (1e)

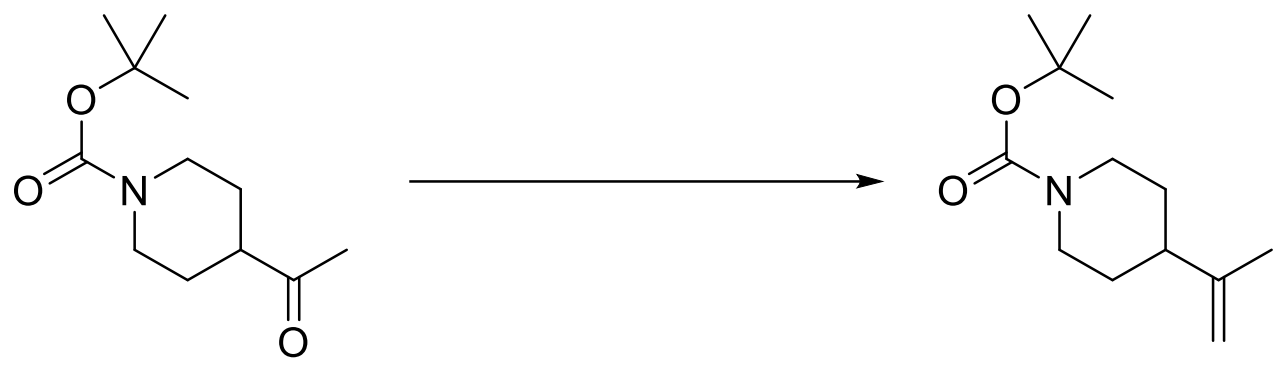

1e

Prepared following **General Procedure A** with *tert*-butyl 4-acetylpiperidine-1-carboxylate (1.14 g, 5.00 mmol, 1.00 equiv). The crude was purified by flash column chromatography (SiO_2; 8:1 pentane:EtOAc) to afford the desired alkene substrate **1e** (0.70 g, 3.1 mmol, 62%) as a colorless oil.

TLC: R_f = 0.71 (6:1 pentane:EtOAc, $KMnO_4$).

NMR Spectroscopy (see spectra):

^{1}H NMR: (400 MHz, $CDCl_3$) δ 4.72 (s, 1H, CHC(CH_3)=C*H*$_2$), 4.69 (s, 1H, CHC(CH_3)=C*H*$_2$), 4.16 (br, 2H, 2 × (Boc)N–C*H*$_2$), 2.68 (t, *J* = 12.0 Hz, 2H, 2 × (Boc)N–C*H*$_2$), 1.99 (tt, *J* = 12.0, 3.0 Hz, 1H, C*H*C(CH_3)=CH_2), 1.72 (s, 3H, CHC(C*H*$_3$)=CH_2), 1.71–1.60 (m, 2H), 1.46 (s, 9H, COOC(C*H*$_3$)$_3$), 1.35 (qd, *J* = 12.0, 4.0 Hz, 2H) ppm.

^{13}C NMR: (101 MHz, $CDCl_3$) δ 155.0 (*C*=O), 149.1 (CH*C*(CH_3)=CH_2), 109.2 (CHC(CH_3)=*C*H$_2$), 79.4 (COO*C*(CH_3)$_3$), 44.4 (2 × (Boc)N–*C*H$_2$), 43.7 (*C*HC(CH_3)=CH_2), 30.9, 28.6 (OC(*C*H$_3$)$_3$), 21.0 (CHC(*C*H$_3$)=CH_2) ppm.

All recorded spectroscopic data matched those previously reported in the literature.[5]

tert-butyldimethyl(((1R,2S,5R)-5-methyl-2-(prop-1-en-2-yl)cyclohexyl)oxy)silane (1h)

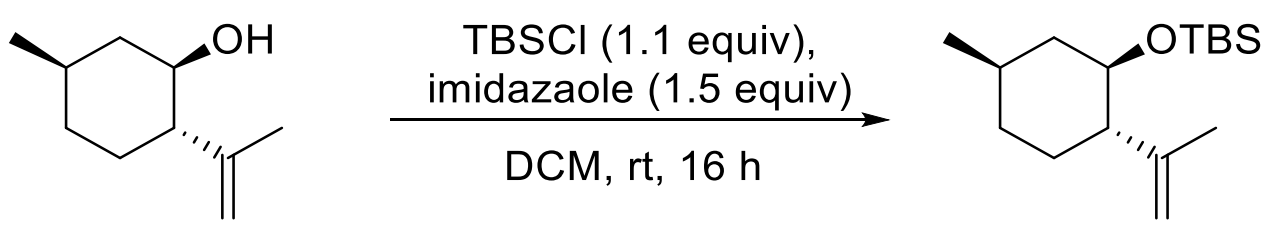

1h

To a solution of (–)-isopulegol (0.31 g, 2.0 mmol, 1.0 equiv) in DCM (4 mL, 0.5 M) was added imidazole (0.20 g, 3.0 mmol, 1.5 equiv) and *tert*-butyldimethylsilyl chloride (0.34 g, 2.2 mmol, 1.1 equiv). The reaction was allowed to stir at room temperature overnight. The reaction was quenched with water (10 mL), the two layers separated, and the aqueous layer was extracted with DCM (3 × 10 mL). The combined organic layers were washed with brine, dried over anhydrous $MgSO_4$, and concentrated under reduced pressure. The crude product was purified by flash column chromatography (SiO_2; 40:1 pentane:EtOAc) to afford the desired TBS-protected alkene substrate **1h** (0.47 g, 1.8 mmol ,88%) as a colorless oil.

TLC: R_f = 0.85 (10:1 pentane:EtOAc, $KMnO_4$).

NMR Spectroscopy (see spectra):

^{1}H NMR: (400 MHz, $CDCl_3$) δ 4.76–4.71 (m, 2H, CHC(CH_3)=C*H*$_2$), 3.47 (td, *J* = 10.2, 4.4 Hz, 1H,

C*H*–OTBS), 1.93–1.82 (m, 2H), 1.68 (t, *J* = 1.2 Hz, 3H, CHC(*CH3*)=CH2), 1.65–1.56 (m, 2H), 1.50–1.37 (m, 1H, CH3C*H*), 1.36–1.23 (dtd, *J* = 12.8, 12.4 ,3.2 Hz, 1H), 1.06–0.95 (m, 1H), 0.91 (d, *J* = 6.4 Hz, 3H, C*H3*CH), 0.85 (s, 9H, OSi(CH3)2C(C*H3*)3), 0.09–0.03 (m, 1H), 0.02 (s, 3H, OSi(C*H3*)2C(CH3)3), 0.01 (s, 3H, OSi(C*H3*)2C(CH3)3) ppm.

13C NMR: (101 MHz, CDCl3) δ 148.1 (CH*C*(CH3)=CH2), 111.2 (CHC(CH3)=*C*H2), 73.5 (*C*H–OTBS), 53.7 (*C*HC(CH3)=CH2), 45.3 (*C*H2CH–OTBS), 34.6, 31.8 (*C*HCH3), 30.7, 26.0 (OSi(CH3)2*C*(CH3)3), 22.5 (CH*C*H3), 21.0 (CHC(*C*H3)=CH2), 18.2, –3.7 (OSi(*C*H3)2C(CH3)3), –4.7 (OSi(*C*H3)2C(CH3)3) ppm.

All recorded spectroscopic data matched those previously reported in the literature.[6]

tert-butyldimethyl(((1*R*,2*R*,5*R*)-2-methyl-5-(prop-1-en-2-yl)cyclohexyl)oxy)silane (1j)

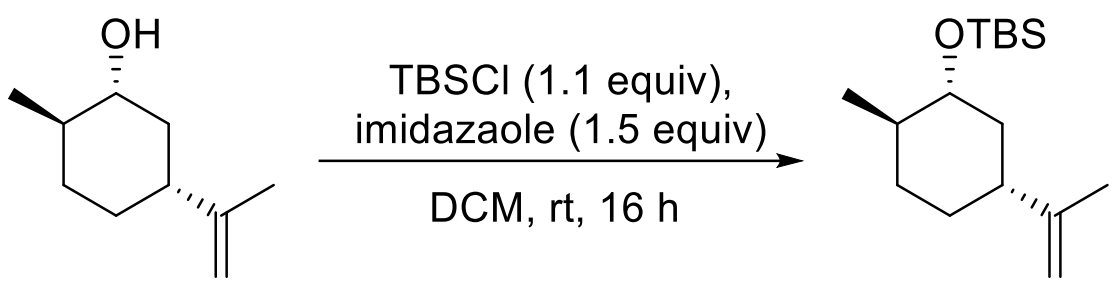

To a solution of (–)-dihydrocarveol (0.31 g, 2.0 mmol, 1.0 equiv) in DCM (10 mL, 0.2 M) was added imidazole (0.54 g, 8.0 mmol, 4.0 equiv) and *tert*-butyldimethylsilyl chloride (0.72 g, 4.8 mmol, 2.4 equiv). The reaction was allowed to stir at room temperature overnight. The reaction was quenched with water (10 mL), the two layers separated, and the aqueous layer was extracted with DCM (3 × 10 mL). The combined organic layers were washed with brine (10 mL), dried over anhydrous $MgSO_4$, and concentrated under reduced pressure. The crude product was purified by flash column chromatography (SiO_2; 40:1 pentane:EtOAc) to afford the desired TBS-protected alkene substrate **1j** (0.41 g, 1.5 mmol ,77%) as a colorless oil.

TLC: R_f = 0.90 (10:1 pentane:EtOAc, $KMnO_4$).

NMR Spectroscopy (*see spectra*):

1H NMR: (400 MHz, CDCl3) δ 4.70–4.66 (m, 2H, CHC(CH3)=C*H2*), 3.16 (ddd, *J* = 10.6, 9.6, 4.0 Hz, 1H, C*H*–OTBS), 1.99–1.90 (tt, *J* = 12.0, 3.2 Hz, 1H), 1.90–1.83 (m, 1H), 1.76–1.69 (m, 1H), 1.71 (t, *J* = 1.2 Hz, 3H, CHC(C*H3*)=CH2), 1.68–1.61 (m, 1H), 1.31–1.23 (m, 2H), 1.21–1.10 (m, 1H), 1.07–0.96 (m, 1H), 0.94 (d, *J* = 6.4 Hz, 3H, C*H3*CH), 0.90 (s, 9H, OSi(CH3)2C(C*H3*)3), 0.06 (2 × s, 6H, OSi(C*H3*)2C(CH3)3) ppm.

13C NMR: (101 MHz, CDCl3) δ 150.0 (CH*C*(CH3)=CH2), 108. 6 (CHC(CH3)=*C*H2), 77.3 (*C*H–OTBS), 44.5, 41.3, 40.4, 33.5, 31.3, 26.1 (OSi(CH3)2*C*(CH3)3), 20.9 (CHC(*C*H3)=CH2, 19.2, 18.3, –3.8 (OSi(*C*H3)2C(CH3)3), –4.5 (OSi(*C*H3)2C(CH3)3) ppm.

HRMS (m/z): (EI) calculated for $C_{16}H_{32}OSi$ $[M]^+$ 268.2217, found 268.2213.

IR (neat) ν_{max}: 2950, 2928, 2857, 1645, 1462, 1374, 1255, 1085, 937, 875, 831, 771, 666 cm^{-1}.

4-(2,2-difluorobenzo[*d*][1,3]dioxol-4-yl)-1-(pent-4-en-1-yl)-1*H*-pyrrole-3-carbonitrile (1n)

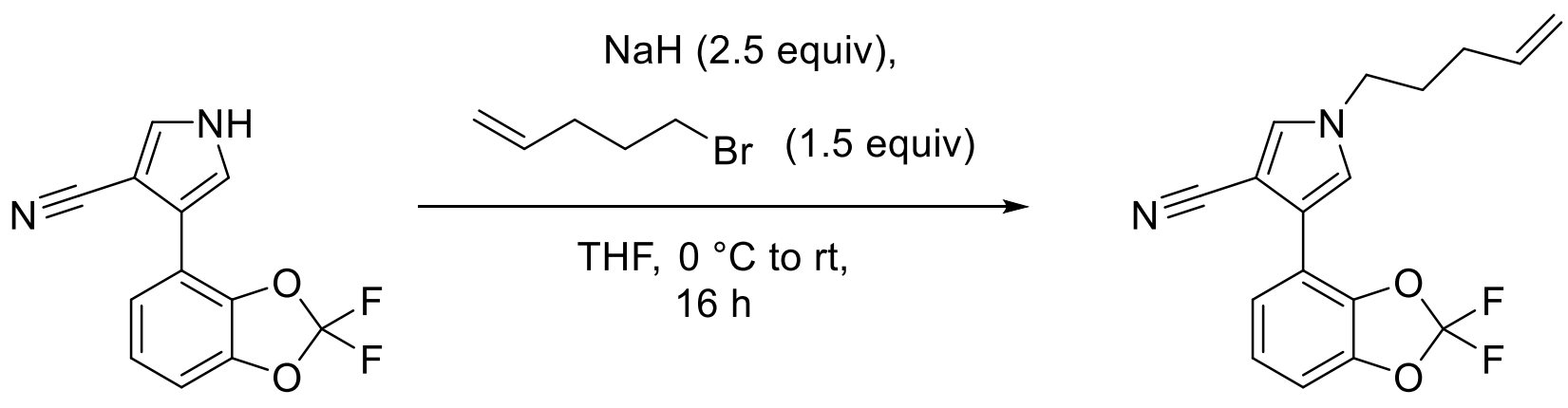

1n

To a solution of fludioxonil (1.24 g, 5.00 mmol, 1.00 equiv) in anhydrous THF (20 mL, 0.25 M) at 0 °C was added sodium hydride (0.30 g, 7.5 mmol, 1.5 equiv) slowly in proportions. The reaction was stirred at 0 °C for 30 min before dropwise addition of 5-bromopent-1-ene (0.90 mL, 7.5 mmol, 1.5 equiv). The reaction was warmed to room temperature and stirred overnight. The reaction was quenched with isopropyl alcohol (1 mL) with vigorous stirring and concentrated under reduced pressure. The crude product was purified by flash column chromatography (SiO_2; 10:1 pentane:EtOAc) to afford the desired alkene substrate **1n** (1.3 g, 4.1 mmol ,82%) as an off-white solid.

TLC: R_f = 0.82 (10:1 pentane:EtOAc, $KMnO_4$).

NMR Spectroscopy (<u>*see spectra*</u>):

^{1}H NMR: (400 MHz, $CDCl_3$) δ 7.72 (dd, *J* = 8.0, 1.2 Hz, 1H, Ar*H*), 7.23 (d, 1H, *J* = 2.4 Hz, Ar*H*), 7.17–7.11 (m, 2H, 2 × Ar*H*), 6.95 (dd, *J* = 8.0, 1.2 Hz, 1H, Ar*H*), 5.84–5.72 (m, 1H, C*H*=CH_2), 5.12–5.05 (m, 2H, CH=C*H$_2$*), 3.96 (t, *J* = 7.2 Hz, 2H, N–C*H$_2$*), 2.10 (td, *J* = 7.2, 7.2 Hz, 2H, C*H$_2$*CH=CH_2), 1.95 (tt, *J* = 7.2, 7.2 Hz, 2H, C*H$_2$*) ppm.

^{13}C NMR: (101 MHz, $CDCl_3$) δ 143.9, 139.9, 136.5 (CH_2*C*H=CH_2), 131.5 (t, *J* = 257 Hz, *C*F_2), 129.7, 124.2, 122.5, 121.3, 118.7, 116.7, 116.64, 116.62, 107.9, 91.2, 49.9 (N–*C*H_2), 30.4, 30.0 ppm.

^{19}F NMR: (377 MHz, $CDCl_3$) δ –49.5 ppm.

All recorded spectroscopic data matched those previously reported in the literature.[7]

(8*S*,9*S*,10*R*,13*S*,14*S*,17*R*)-10,13-dimethyl-17-(prop-1-en-2-yl)-1,2,6,7,8,9,10,11,12,13,14,15,16,17-tetradecahydro-3*H*-cyclopenta[*a*]phenanthren-3-one (1o)

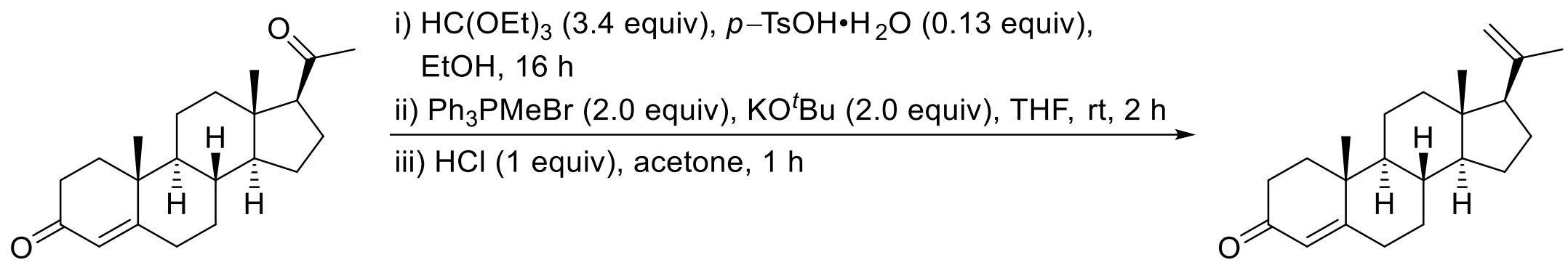

1o

Prepared by following a modified literature procedure.[8] *p*-toluenesulfonic acid monohydrate (25 mg, 0.26 mmol, 0.066 equiv) was added to a stirred solution of progesterone (1.26 g, 4.00 mmol, 1.00 equiv) and triethyl orthoformate (1.13 mL, 6.80 mmol, 1.70 equiv) in EtOH (50 mL, 0.08 M). Another proportion of *p*-toluenesulfonic

acid monohydrate (25 mg, 0.26 mmol, 0.066 equiv) was added and stirred at room temperature overnight. The reaction was quenched with saturated aqueous $NaHCO_3$ (50 mL), the two layers separated, and the aqueous layer was extracted with EtOAc (3 × 25 mL). The combined organic layers were washed with water and brine, dried over anhydrous $MgSO_4$, and concentrated under reduced pressure, affording the ethyl enol ether as a white solid and used in the next step without further purification. A flame-dried 50 mL RBF was charged with a magnetic stirrer bar and methyltriphenylphosphonium bromide (2.9 g, 8.0 mmol, 2.0 equiv) followed by the addition of THF (20 mL, 0.2 M). The mixture was then cooled to 0 °C followed by addition of potassium *tert*-butoxide (0.90 g, 8.0 mmol, 2.0 equiv). The yellow suspension was allowed to warm up to room temperature and stirred for 1 h before cooling down to 0 °C again followed by dropwise addition of the ethyl enol ether in THF (5 mL). The reaction mixture was then allowed to stir at room temperature overnight. The reaction was quenched by the addition of water (25 mL), and the resulting mixture was stirred vigorously for 15 minutes. The aqueous phase was extracted with EtOAc (3 × 20 mL), and the combined organic layers were washed sequentially with water (25 mL), saturated aqueous $NaHCO_3$ (25 mL) and brine (25 mL), dried over anhydrous $MgSO_4$, and concentrated under reduced pressure. The resulting residue was then dissolved in DCM (5 mL) and slowly added to 75 mL of heptane at 0 °C, resulting in crystallization of triphenylphosphine oxide. The liquid was then filtered and concentrated under reduced pressure. The crude product was purified by flash column chromatography (SiO_2; 100:1 pentane:EtOAc with 0.5% Et_3N additive) and the ethyl enol ether protected alkene was dissolved in acetone (50 mL) under N_2 atmosphere and aqueous 1 M HCl (2.4 mL, 2.4 mmol, 1.0 equiv) was added. The reaction was allowed to stir for 1 h at room temperature before neutralization of the reaction with saturated aqueous $NaHCO_3$ (25 mL). The layers were separated, and the aqueous phase was extracted with EtOAc (3 × 25 mL), and the combined organic layers were washed with water (25 mL) and brine (25 mL), dried over anhydrous $MgSO_4$, and concentrated under reduced pressure. The crude product was purified by flash column chromatography (SiO_2; 10:1 pentane:EtOAc) to afford the desired alkene substrate **1o** (0.56 g, 1.8 mmol ,45%) as an amorphous white solid.

TLC: R_f = 0.48 (10:1 pentane:EtOAc, $KMnO_4$).

NMR Spectroscopy (*see spectra*):

^{1}H NMR: (400 MHz, $CDCl_3$) δ 5.73 (s, 1H, C$\underline{H}$=C–C=O), 4.86 (s, 1H, CHC(CH_3)=C$\underline{H}_2$), 4.71 (s, 1H, CHC(CH_3)=C$\underline{H}_2$), 2.49–2.23 (m, 4H, 2 × C$\underline{H}_2$), 2.07–1.98 (m, 2H), 1.94–1.82 (m, 2H), 1.76 (s, 3H, CHC(C$\underline{H}_3$)=CH_2), 1.73–1.64 (m, 3H, C$\underline{H}$C(CH_3)=CH_2 + C$\underline{H}_2$), 1.61–1.34 (m, 4H), 1.29–1.20 (m, 2H), 1.20 (s, 3H, C$\underline{H}_3$), 1.15–0.91 (m, 3H, 3 × C$\underline{H}$), 0.60 (s, 3H, C$\underline{H}_3$) ppm.

^{13}C NMR: (101 MHz, $CDCl_3$) δ 199.7 (CH=C–$\underline{C}$=O), 171.7 ($\underline{C}$H=C–C=O), 145.5 (CH$\underline{C}$(CH_3)=CH_2), 123.9 (CH=$\underline{C}$–C=O), 111.1 (CHC(CH_3)=$\underline{C}H_2$), 57.3, 55.8, 54.1, 43.3, 38.8, 38.7, 36.1, 35.8, 34.1, 33.0, 32.1, 25.5, 24.8, 24.3, 21.2, 17.5, 12.9 ppm.

All recorded spectroscopic data matched those previously reported in the literature.[9]

methyl (1*R*,3a*S*,5a*R*,5b*R*,7a*R*,9*S*,11a*R*,11b*R*,13a*R*,13b*R*)-9-hydroxy-5a,5b,8,8,11a-pentamethyl-1-(prop-1-en-2-yl)icosahydro-3a*H*-cyclopenta[*a*]chrysene-3a-carboxylate (1p)

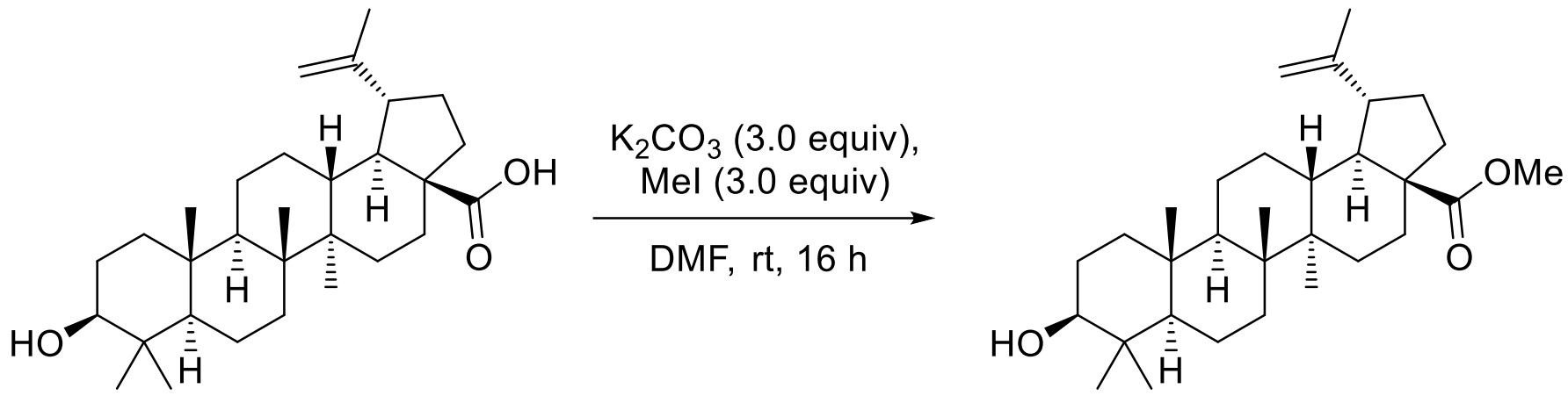

1p

A solution of betulinic acid (0.91 g, 2.0 mmol, 1.00 equiv) and K_2CO_3 (0.83 g, 3.0 mmol, 3.0 equiv) in DMF (20 mL, 0.1 M) was stirred at room temperature for 30 min before dropwise addition of iodomethane (0.36 mL, 6.0 mmol, 3.0 equiv). The reaction was then heated to 50 °C and allowed to stir overnight. The reaction was diluted with EtOAc (20 mL) and then washed with water (20 mL), saturated Na_2CO_3 (20 mL) and brine (20 mL). The organic layer was dried over anhydrous $MgSO_4$ and concentrated under reduced pressure. The crude product was purified by flash column chromatography (SiO_2; 50:1 pentane:EtOAc) to the desired alkene substrate **1p** (0.56 g, 1.2 mmol ,59%) as a white powder.

TLC: R_f = 0.58 (10:1 pentane:EtOAc, $KMnO_4$).

NMR Spectroscopy (*see spectra*):

^{1}H NMR: (400 MHz, $CDCl_3$) δ 4.73 (s, 1H, CHC(CH_3)=C*H*$_2$), 4.59 (s, 1H, CHC(CH_3)=C*H*$_2$), 3.66 (s, 3H, COOC*H*$_3$), 3.18 (dd, *J* = 11.2, 5.2 Hz, 1H, C*H*–OH), 3.01 (dt, *J* = 11.2, 4.8 Hz, 1H), 2.25–2.14 (m, 2H), 1.94–1.82 (m, 2H), 1.73–1.63 (m, 2H), 1.68 (s, 3H, CHC(C*H*$_3$)=CH_2), 1.63–1.45 (m, 4H), 1.44–1.30 (m, 9H), 1.29–1.17 (m, 3H), 1.17–1.11 (m, 1H), 1.08–0.97 (m, 1H), 0.96 (s, 6H, 2 × C*H*$_3$), 0.91 (s, 3H, C*H*$_3$), 0.81 (s, 3H, C*H*$_3$), 0.75 (s, 3H, C*H*$_3$), 0.70–0.65 (m, 1H) ppm.

^{13}C NMR: (101 MHz, $CDCl_3$) δ 176.8 (*C*=O), 150.7 (CH*C*(CH_3)=CH_2), 109.7 (CHC(CH_3)=*C*H_2), 79.1 (*C*H–OH), 56.7, 55.5, 51.4, 50.7, 49.6, 47.1, 42.5, 40.8, 39.0, 38.9, 38.4, 37.3, 37.1, 34.5, 32.3, 30.8, 29.8, 28.1, 27.6, 25.7, 21.0, 19.5, 18.4, 16.3, 16.1, 15.5, 14.9 ppm.

All recorded spectroscopic data matched those previously reported in the literature.[10]

methyl (1*R*,3a*S*,5a*R*,5b*R*,7a*R*,9*S*,11a*R*,11b*R*,13a*R*,13b*R*)-9-((tert-butyldimethylsilyl)oxy)-5a,5b,8,8,11a-pentamethyl-1-(prop-1-en-2-yl)icosahydro-3a*H*-cyclopenta[*a*]chrysene-3a-carboxylate (1q)

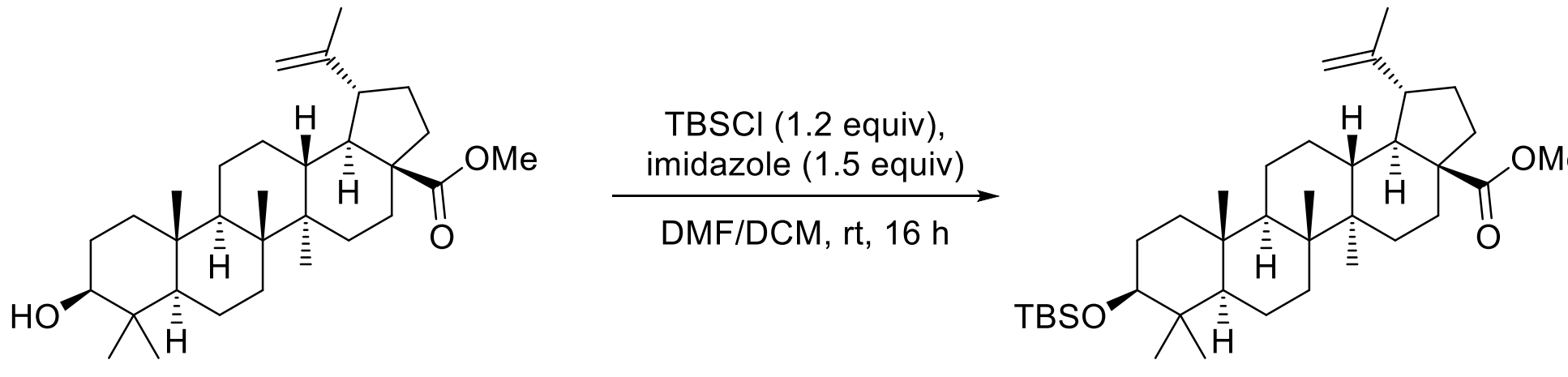

1p **1q**

To a solution of methyl (1*R*,3a*S*,5a*R*,5b*R*,7a*R*,9*S*,11a*R*,11b*R*,13a*R*,13b*R*)-9-hydroxy-5a,5b,8,8,11a-

pentamethyl-1-(prop-1-en-2-yl)icosahydro-3a*H*-cyclopenta[*a*]chrysene-3a-carboxylate **1p** (0.47 g, 1.0 mmol, 1.0 equiv) in DMF/DCM (2/8 mL, 0.1 M) was added imidazole (0.10 g, 1.5 mmol, 1.5 equiv) and tert-butyldimethylsilyl chloride (0.18 g, 1.2 mmol, 1.2 equiv). The reaction was allowed to stir at room temperature overnight. The reaction was quenched with water (10 mL), the two layers separated, and the aqueous layer was extracted with DCM (3 × 10 mL). The combined organic layers were washed with brine (10 mL), dried over anhydrous $MgSO_4$, and concentrated under reduced pressure. The crude product was purified by flash column chromatography (SiO_2; 75:1 pentane:EtOAc) to the desired TBS-protected alkene substrate **1q** (0.41 g, 0.70 mmol, 70%) as a white powder.

TLC: R_f = 0.89 (20:1 pentane:EtOAc, $KMnO_4$).

NMR Spectroscopy (<u>*see spectra*</u>):

^{1}H NMR: (400 MHz, $CDCl_3$) δ 4.73 (s, 1H, CHC(CH_3)=C<u>*H*$_2$</u>), 4.59 (s, 1H, CHC(CH_3)=C<u>*H*$_2$</u>), 3.66 (s, 3H, COOC<u>*H*$_3$</u>), 3.15 (dd, *J* = 11.2, 5.2 Hz, 1H, C<u>*H*</u>–OTBS), 3.00 (dt, *J* = 11.2, 4.8 Hz, 1H), 2.26–2.14 (m, 2H), 1.94–1.82 (m, 2H), 1.72–1.65 (m, 4H), 1.68 (s, 3H, CHC(C*H*$_3$)=CH_2), 1.63–1.54 (m, 3H), 1.53–1.44 (m, 2H), 1.44–1.26 (m, 9H), 1.26–1.19 (m, 2H), 1.13 (dd, *J* = 9.6, 2.4 Hz, 1H), 1.07–0.98 (m, 1H), 0.95 (s, 3H, C(C*H*$_3$)$_2$), 0.90 (s, 3H, C(C*H*$_3$)$_2$), 0.88 (s, 9H, OSi(CH_3)$_2$C(CH_3)$_3$), 0.87 (s, 3H, C*H*$_3$), 0.81 (s, 3H, C*H*$_3$), 0.71 (s, 3H, C*H*$_3$), 0.67–0.63 (m, 1H), 0.02 (s, 6H, OSi(CH_3)$_2$C(CH_3)$_3$) ppm.

^{13}C NMR: (101 MHz, $CDCl_3$) δ 176.8 (<u>*C*</u>=O), 150.7 (CH<u>*C*</u>(CH_3)=CH_2), 109.7 (CHC(CH_3)=<u>*C*</u>H_2), 79.6 (<u>*C*</u>H–OTBS), 56.7, 55.6, 51.4, 50.8, 49.7, 47.1, 42.5, 40.8, 39.6, 38.9, 38.4, 37.2, 37.1, 34.6, 32.3, 30.8, 29.8, 28.6, 28.0, 26.1 (OSi(CH_3)$_2$C(<u>*C*</u>H_3)$_3$), 25.7, 21.0, 19.5, 18.6, 18.3, 16.3, 16.1, 16.0, 14.8, –3.6 (OSi(<u>*C*</u>H_3)$_2$C(CH_3)$_3$), –4.8 (OSi(<u>*C*</u>H_3)$_2$C(CH_3)$_3$) ppm.

HRMS (m/z): (MALDI) calculated for $C_{37}H_{64}O_3SiNa$ $[M+Na]^+$ 607.4517, found 607.4510.

IR (neat) ν_{max}: 2948, 2856, 1730, 1643, 1462, 1275, 1260, 1098, 1067, 885, 836, 764, 750 cm^{-1}.

***tert*-butyl (1*R*,3a*S*,5a*R*,5b*R*,7a*R*,9*S*,11a*R*,11b*R*,13a*R*,13b*R*)-9-hydroxy-5a,5b,8,8,11a-pentamethyl-1-(prop-1-en-2-yl)icosahydro-3a*H*-cyclopenta[*a*]chrysene-3a-carboxylate (SI-4)**

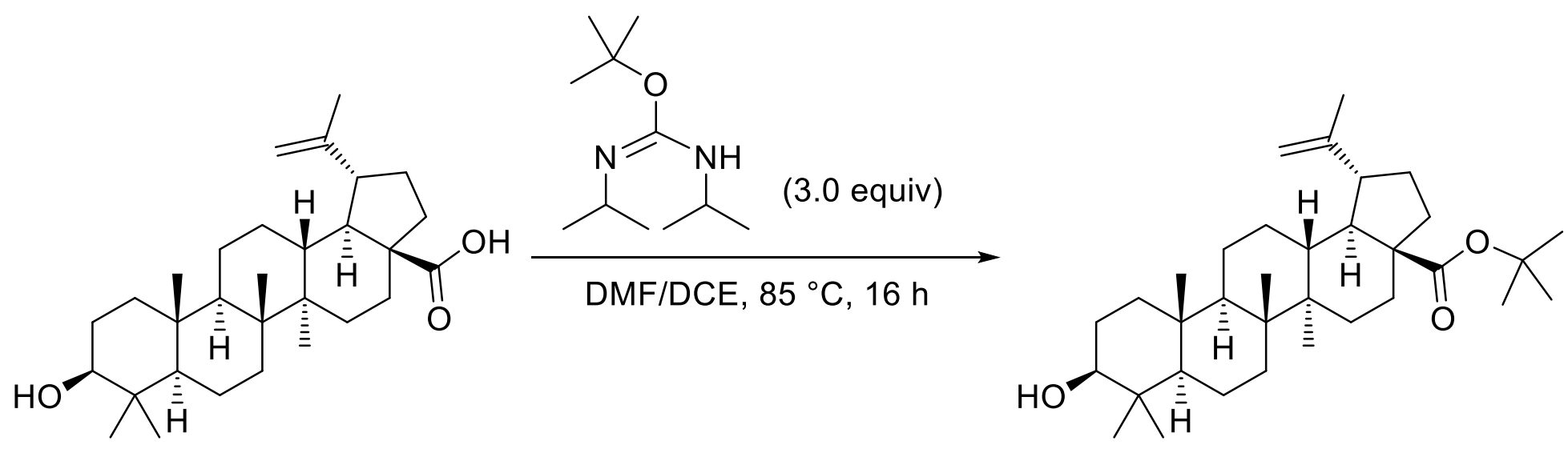

SI-4

Tert-butyl (*E*)-*N*,*N'*-diisopropylcarbamimidate (7.2 g, 24 mmol, 3.0 equiv) was added to a solution of betulinic acid (3.6 g, 8.0 mmol, 1.0 equiv) in DMF/DCE (10/30 mL, 0.2 M) at room temperature. The reaction was then heated to 85 °C and allowed to stir overnight. The reaction was cooled to room temperature, filtered through a short pad of celite and concentrated under reduced pressure. The crude product was purified by flash column

chromatography (SiO_2; 75:1 pentane:EtOAc) to give the desired *tert*-butyl ester **SI-4** (2.2 g, 4.2 mmol ,53%) as a white powder.

TLC: R_f = 0.61 (10:1 pentane:EtOAc, $KMnO_4$).

NMR Spectroscopy (*see spectra*):

¹H NMR: (400 MHz, $CDCl_3$) δ 4.73 (s, 1H, CHC(CH₃)=C*H₂*), 4.59 (s, 1H, CHC(CH₃)=C*H₂*), 3.18 (dd, *J* = 10.8, 4.4 Hz, 1H, C*H*–OH), 3.01 (dt, *J* = 11.2, 3.2 Hz, 1H), 2.29–2.14 (m, 2H), 1.98–1.77 (m, 2H), 1.72–1.62 (m, 2H), 1.68 (s, 3H, CHC(C*H₃*)=CH₂), 1.61–1.48 (m, 5H), 1.46 (s, 9H, COOC(C*H₃*)₃), 1.41–1.31 (m, 6H), 1.31–1.18 (m, 4H), 1.16–1.09 (m, 1H), 1.07–0.86 (m, 2H), 0.96 (s, 6H, 2 × C*H₃*), 0.94 (s, 3H, C*H₃*), 0.81 (s, 3H, C*H₃*), 0.75 (s, 3H, C*H₃*), 0.70–0.65 (m, 1H) ppm.

¹³C NMR: (101 MHz, $CDCl_3$) δ 175.7 (*C*=O), 151.7 (CH*C*(CH₃)=CH₂), 109.5 (CHC(CH₃)=*C*H₂), 79.7 (COO*C*(CH₃)₃), 79.1 (*C*H–OH), 57.0, 55.5, 50.8, 49.4, 47.2, 42.6, 40.9, 39.0, 38.9, 38.4, 37.4, 34.5, 32.6, 30.9, 29.8, 28.2, 28.1, 27.6, 25.8, 21.1, 19.6, 18.4, 16.3, 16.1, 15.5, 14.8 ppm.

HRMS (m/z): (MALDI) calculated for $C_{34}H_{56}O_3Na$ $[M+Na]^+$ 535.4122, found 535.4126.

IR (neat) ν_{max}: 3624, 2942, 2868, 1719, 1642, 1453, 1366, 1275, 1261, 1147, 1135, 764, 750 cm^{-1}.

***tert*-butyl (1*R*,3a*S*,5a*R*,5b*R*,7a*R*,9*S*,11a*R*,11b*R*,13a*R*,13b*R*)-9-((tert-butyldimethylsilyl)oxy)-5a,5b,8,8,11a-pentamethyl-1-(prop-1-en-2-yl)icosahydro-3a*H*-cyclopenta[*a*]chrysene-3a-carboxylate (1r)**

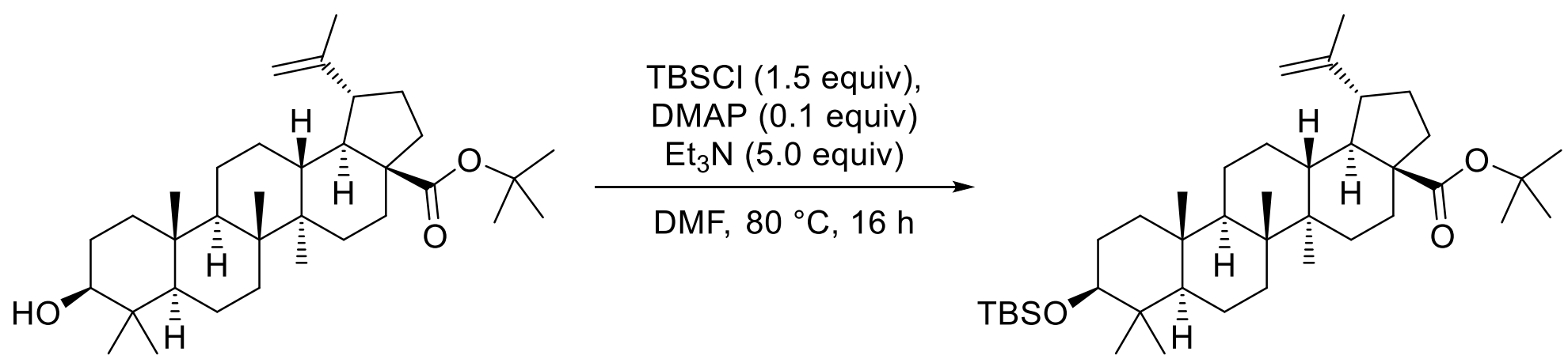

To a solution of *tert*-butyl (1*R*,3a*S*,5a*R*,5b*R*,7a*R*,9*S*,11a*R*,11b*R*,13a*R*,13b*R*)-9-hydroxy-5a,5b,8,8,11a-pentamethyl-1-(prop-1-en-2-yl)icosahydro-3a*H*-cyclopenta[*a*]chrysene-3a-carboxylate **SI-4** (2.1 g, 4.0 mmol, 1.0 equiv) in DMF (40 mL, 0.1 M) was added 4-dimethylaminopyridine (49 mg, 0.40 mmol, 0.10 equiv), triethylamine (2.8 mL, 20 mmol, 5.0 equiv) and tert-butyldimethylsilyl chloride (0.90 g, 6.0 mmol, 1.5 equiv). The reaction was heated to 80 °C and allowed to stir overnight. The reaction was quenched with water (20 mL), the two layers separated, and the aqueous layer was extracted with DCM (3 × 20 mL). The combined organic layers were washed with brine (20 mL), dried over anhydrous $MgSO_4$, and concentrated under reduced pressure. The crude product was purified by flash column chromatography (SiO_2; 100:1 pentane:EtOAc) to give the desired TBS-protected alkene substrate **1r** (1.8 g, 2.9 mmol ,73%) as a white powder.

TLC: R_f = 0.92 (20:1 pentane:EtOAc, $KMnO_4$).

NMR Spectroscopy (*see spectra*):

^{1}H NMR: (400 MHz, $CDCl_3$) δ 4.72 (d, *J* = 2.4 Hz, 1H, CHC(CH_3)=C*H*$_2$), 4.59 (dd, *J* = 2.4, 1.6 Hz, 1H, CHC(CH_3)=C*H*$_2$), 3.15 (dd, *J* = 11.2, 4.4 Hz, 1H, C*H*–OTBS), 3.01 (dt, *J* = 11.2, 3.2 Hz, 1H), 2.29–2.16 (m, 2H), 1.98–1.78 (m, 2H), 1.68 (s, 3H, CHC(C*H*$_3$)=CH_2), 1.64–1.48 (m, 5H), 1.46 (s, 9H, COOC(C*H*$_3$)$_3$), 1.44–1.31 (m, 8H), 1.30–1.17 (m, 3H), 1.16–1.09 (m, 1H), 1.05–0.96 (m, 1H), 0.95 (s, 3H, C*H*$_3$), 0.93 (s, 3H, C*H*$_3$), 0.88 (s, 9H, OSi(CH_3)$_2$C(C*H*$_3$)$_3$), 0.87 (s, 3H, C*H*$_3$), 0.85–0.83 (m, 1H), 0.81 (s, 3H, C*H*$_3$), 0.71 (s, 3H, C*H*$_3$), 0.67–0.63 (m, 1H), 0.02 (s, 6H, OSi(C*H*$_3$)$_2$C(CH_3)$_3$) ppm.

^{13}C NMR: (101 MHz, $CDCl_3$) δ 175.7 (*C*=O), 151.1 (CH*C*(CH_3)=CH_2), 109.5 (CHC(CH_3)=*C*H$_2$), 79.7 (COO*C*(CH_3)$_3$), 79.6 (*C*H–OTBS), 57.0, 55.6, 50.8, 49.4, 47.2, 42.6, 41.0, 39.6, 38.9, 38.4, 37.4, 37.2, 34.6, 32.6, 30.9, 29.8, 28.6, 28.2, 28.0, 26.1, 25.8, 21.1, 19.6, 18.7, 18.3, 16.3, 16.1, 16.0, 14.8, –3.6 (OSi(*C*H$_3$)$_2$C(CH_3)$_3$), –4.8 (OSi(*C*H$_3$)$_2$C(CH_3)$_3$) ppm.

HRMS (m/z): (MALDI) calculated for $C_{40}H_{70}O_3SiNa$ $[M+Na]^+$ 649.4984, found 649.4993.

IR (neat) ν_{max}**:** 2942, 2860, 1721, 1642, 1458, 1389, 1366, 1253 ,1147, 1131, 885, 836, 773 cm^{-1}.

***tert*-butyl 4-methylenepiperidine-1-carboxylate (3b)**

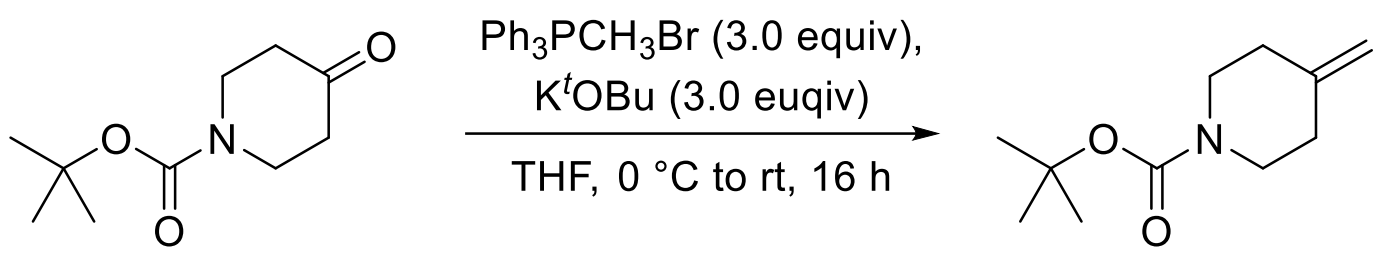

3b

Prepared following **General Procedure A** with *tert*-butyl 4-acetylpiperidine-1-carboxylate (1.0 g, 5.0 mmol, 1.0 equiv). The crude was purified by flash column chromatography (SiO_2; 8:1 pentane:EtOAc) to afford the desired alkene substrate **3b** (0.97 g, 4.9 mmol, 98%) as a colorless oil.

TLC: R_f = 0.58 (5:1 pentane:EtOAc, $KMnO_4$).

NMR Spectroscopy (*see spectra*):

^{1}H NMR: (400 MHz, $CDCl_3$) δ 4.74 (s, 2H, C(CH_2)$_2$=C*H*$_2$), 3.41 (t, *J* = 5.6 Hz, 4H, 2 × (Boc)N–C*H*$_2$), 2.17 (t, *J* = 5.6 Hz, 4H, 2 × C*H*$_2$), 1.46 (s, 9H, OC(C*H*$_3$)$_3$) ppm.

^{13}C NMR: (101 MHz, $CDCl_3$) δ 154.9 (*C*=O), 145.5 (C(CH_2)$_2$=*C*H$_2$), 109.2 (*C*(CH_2)$_2$=CH_2), 79.6 (O*C*(CH_3)$_3$), 45.7 ((Boc)N–*C*H$_2$), 34.7 (*C*H$_2$), 28.6 (OC(*C*H$_3$)$_3$) ppm.

All recorded spectroscopic data matched those previously reported in the literature.[11]

tert-butyl (1R,5S)-3-methylene-8-azabicyclo[3.2.1]octane-8-carboxylate (3d)

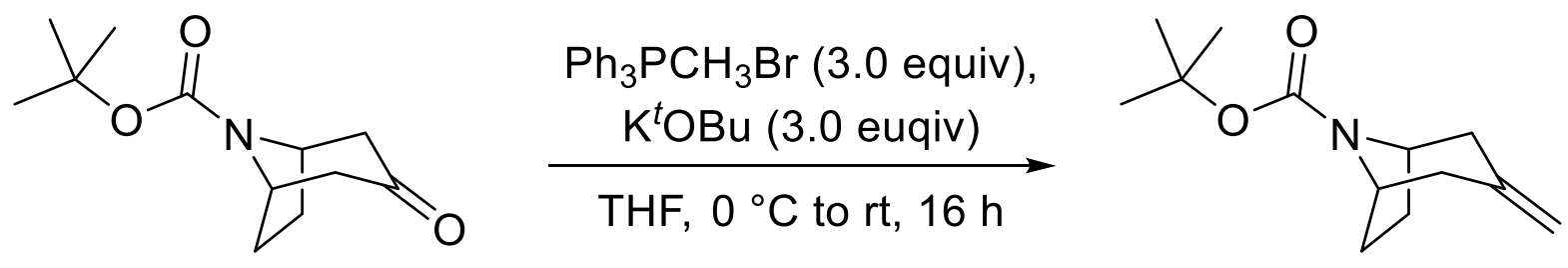

3d

Prepared following **General Procedure A** with *tert*-butyl (1*R*,5*S*)-3-oxo-8-azabicyclo[3.2.1]octane-8-carboxylate (1.1 g, 5.0 mmol, 1.0 equiv). The crude was purified by flash column chromatography (SiO_2; 10:1 pentane:EtOAc) to afford the desired alkene substrate **3d** (1.0 g, 4.6 mmol, 92%) as a colorless oil.

TLC: R_f = 0.78 (4:1 pentane:EtOAc, $KMnO_4$).

NMR Spectroscopy (*see spectra*):

^{1}H NMR: (400 MHz, $CDCl_3$) δ 4.85 (s, 2H, C(CH_2)$_2$=C<u>*H*$_2$</u>), 4.28 (br, 1H, (Boc)N–C<u>*H*</u>), 4.19 (br, 1H, (Boc)N–C<u>*H*</u>), 2.57–2.36 (m, 2H), 2.09 (br, 1H), 2.05 (br, 1H), 1.90–1.83 (m, 2H), 1.61–1.54 (m, 2H), 1.48 (s, 9H, OC(C<u>*H*$_3$</u>)$_3$) ppm.

^{13}C NMR: (101 MHz, $CDCl_3$) δ 153.7 (<u>*C*</u>=O), 142.5 (C(CH_2)$_2$=<u>*C*</u>H$_2$), 113.5 (<u>*C*</u>(CH_2)$_2$=CH_2), 79.3 (O<u>*C*</u>(CH_3)$_3$), 54.6 ((Boc)N–<u>*C*</u>H), 54.0 ((Boc)N–<u>*C*</u>H), 41.2, 40.5, 28.7 (OC(<u>*C*</u>H$_3$)$_3$) ppm.

All recorded spectroscopic data matched those previously reported in the literature.[12]

2.4. Substrate Scope

4,4,5,5-tetramethyl-2-phenethyl-1,3,2-dioxaborolane (2a)

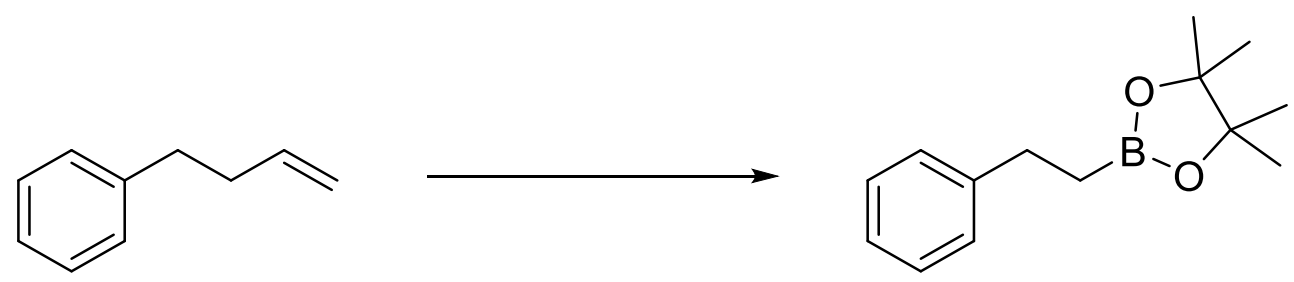

1a **2a**

Prepared following **General Procedure B** with but-3-en-1-ylbenzene **1a** (26.4 mg, 0.200 mmol, 1.00 equiv). The crude was purified by flash column chromatography (SiO_2; 20:1 pentane:EtOAc) to afford the desired boronic ester **2a** (21 mg, 0.090 mmol, 45%) as a pale-yellow solid.

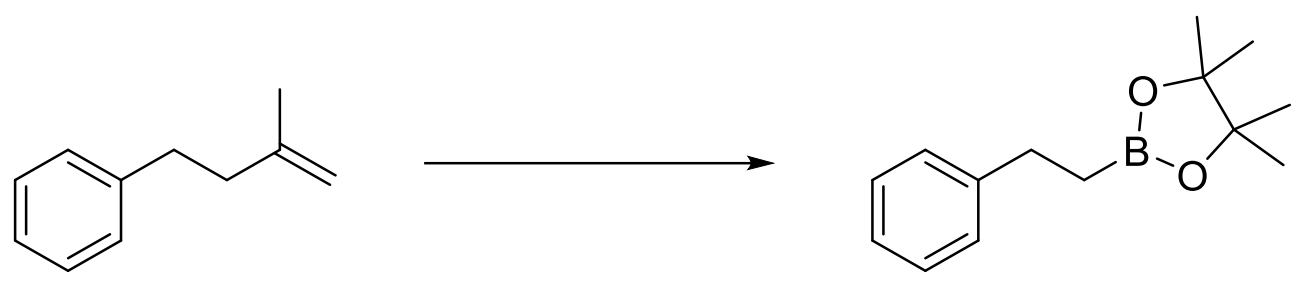

1b **2a**

Prepared following **General Procedure B** with (3-methylbut-3-en-1-yl)benzene **1b** (29.2 mg, 0.200 mmol, 1.00 equiv). The crude was purified by flash column chromatography (SiO_2; 20:1 pentane:EtOAc) to afford the desired boronic ester **2a** (29 mg, 0.13 mmol, 63%) as a pale-yellow solid.

TLC: R_f = 0.30 (20:1 pentane:EtOAc, CAM).

NMR Spectroscopy (*see spectra*):

¹H NMR: (400 MHz, $CDCl_3$) δ 7.29–7.20 (m, 4H, 4 × Ar*H*), 7.18–7.12 (m, 1H, Ar*H*), 2.75 (t, *J* = 8.0 Hz, 2H, Ar–C*H₂*), 1.22 (s, 12H, 2 × B–OC(C*H₃*)₂), 1.15 (t, *J* = 8.0 Hz, 2H, C*H₂*–Bpin) ppm.

¹³C NMR: (101 MHz, $CDCl_3$) δ 144.6, 128.3, 128.1, 125.6, 83.2 (B–O*C*(CH_3)$_2$), 30.1 (Ar–*C*H_2), 25.0 (B–OC(*C*H_3)$_2$) ppm. *The carbon attached to boron was not observed due to quadrupolar relaxation.*

All recorded spectroscopic data matched those previously reported in the literature.[13]

2-cyclohexyl-4,4,5,5-tetramethyl-1,3,2-dioxaborolane (2c)

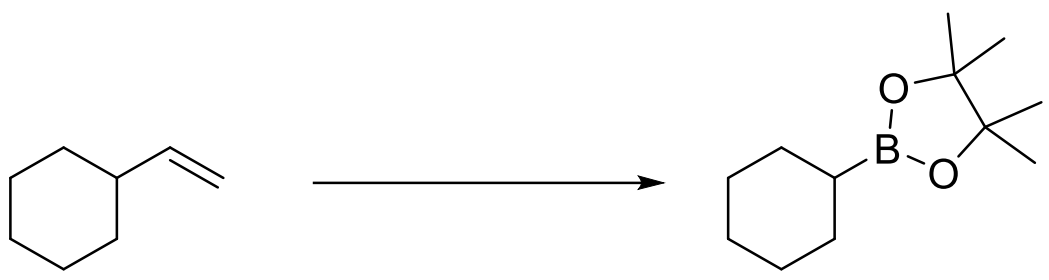

1c **2c**

Prepared following **General Procedure B** with vinylcyclohexane **1c** (22.0 mg, 0.200 mmol, 1.00 equiv). The crude was purified by flash column chromatography (SiO_2; 20:1 pentane:EtOAc) to afford the desired boronic ester **2c** (18 mg, 0.086 mmol, 43%) as a colorless oil.

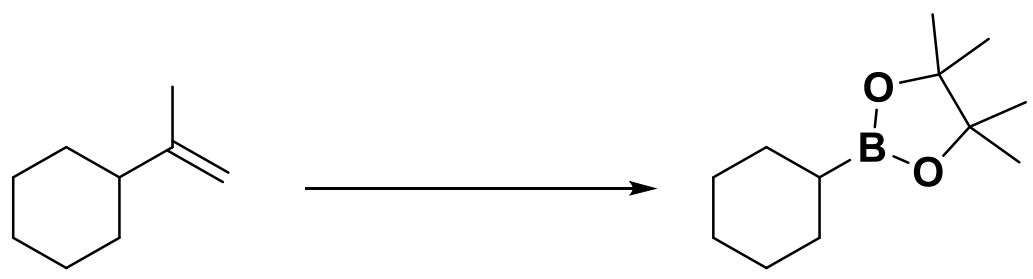

1d **2c**

Prepared following **General Procedure B** with prop-1-en-2-ylcyclohexane **1d** (24.8 mg, 0.200 mmol, 1.00 equiv). The crude was purified by flash column chromatography (SiO_2; 20:1 pentane:EtOAc) to afford the desired boronic ester **2c** (25 mg, 0.12 mmol, 60%) as a colorless oil.

TLC: R_f = 0.50 (40:1 pentane:EtOAc, CAM).

NMR Spectroscopy (*see spectra*):

¹H NMR: (400 MHz, $CDCl_3$) δ 1.70–1.52 (m, 5H), 1.39–1.24 (m, 5H), 1.23 (s, 12H, 2 × B–OC(C*H₃*)₂), 1.02–0.91 (m, 1H, C*H*–Bpin) ppm.

¹³C NMR: (101 MHz, $CDCl_3$) δ 82.9 (B–O*C*(CH_3)$_2$), 28.1, 27.3, 26.9, 24.9 (B–OC(*C*H_3)$_2$) ppm. *The carbon attached to boron was not observed due to quadrupolar relaxation.*

All recorded spectroscopic data matched those previously reported in the literature.[14]

tert-butyl 4-(4,4,5,5-tetramethyl-1,3,2-dioxaborolan-2-yl)piperidine-1-carboxylate (2e)

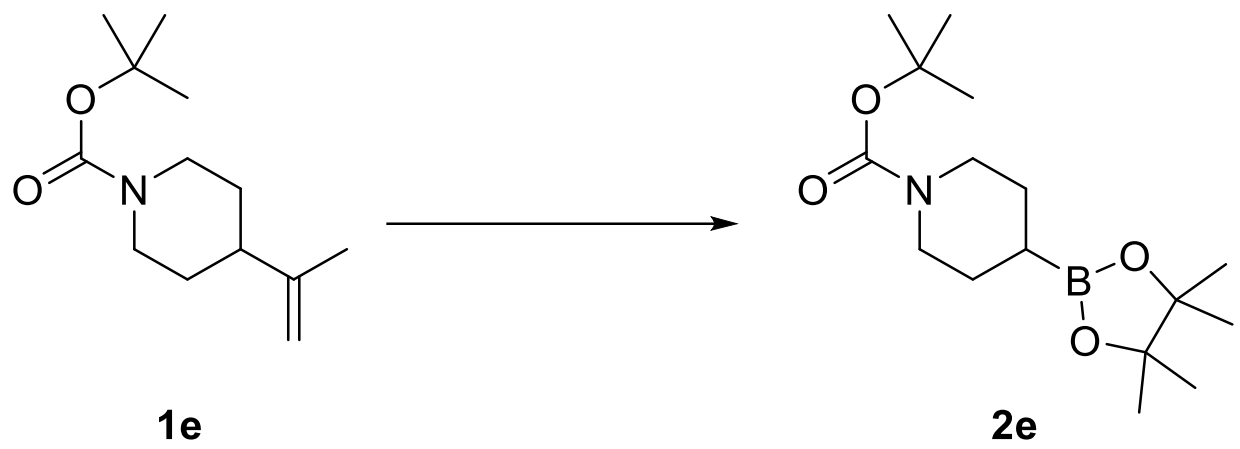

1e **2e**

Prepared following **General Procedure C** with *tert*-butyl 4-(prop-1-en-2-yl)piperidine-1-carboxylate **1e** (45.1 mg, 0.200 mmol, 1.00 equiv). The crude was purified by flash column chromatography (boric acid treated SiO_2; 12:1 pentane:EtOAc) to afford the desired boronic ester **2e** (37 mg, 0.12 mmol, 60%) as a colorless oil.

TLC: R_f = 0.60 (4:1 pentane:EtOAc, CAM).

NMR Spectroscopy (see spectra):

^{1}H NMR: (400 MHz, $CDCl_3$) δ 3.88–3.64 (m, 2H, 2 × (Boc)N–C*H*₂), 2.97–2.85 (m, 2H, 2 × (Boc)N–C*H*₂), 1.68–1.57 (m, 2H, 2 × C*H*₂), 1.53–1.40 (m, 2H, 2 × C*H*₂), 1.44 (s, 9H, OC(C*H*₃)₃), 1.23 (s, 12H, 2 × B–OC(C*H*₃)₂), 1.14–1.05 (m, 1H, C*H*–Bpin) ppm.

^{13}C NMR: (101 MHz, $CDCl_3$) δ 155.0 (*C*=O), 83.3 (B–O*C*(CH₃)₂), 79.2 (O*C*(CH₃)₃), 28.6 ((Boc)N–*C*H₂), 27.1, 25.2 (B–OC(*C*H₃)₃), 24.9 (OC(*C*H₃)₃) ppm. *The carbon attached to boron was not observed due to quadrupolar relaxation.*

All recorded spectroscopic data matched those previously reported in the literature.[15]

(2*R*)-2-methyl-5-(4,4,5,5-tetramethyl-1,3,2-dioxaborolan-2-yl)cyclohexan-1-one (2f)

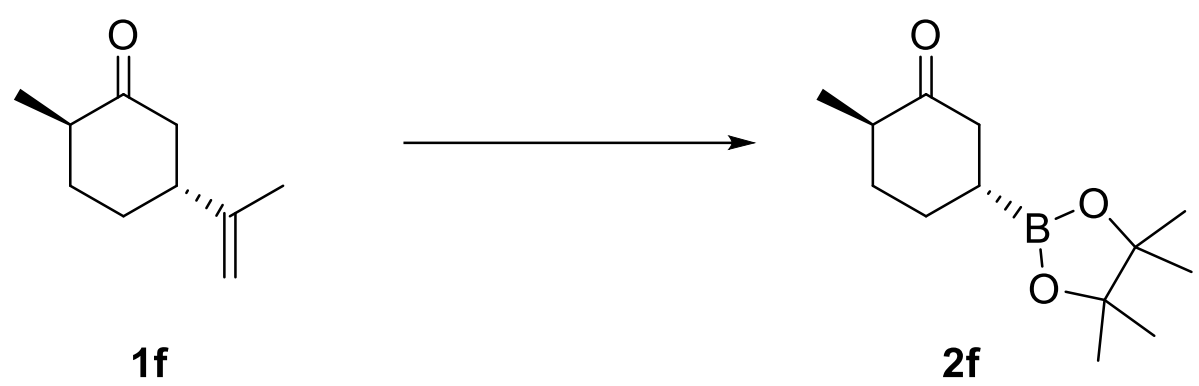

1f **2f**

Prepared following **General Procedure B** with (+)-dihydrocarvone **1f** (30.4 mg, 0.200 mmol, 1.00 equiv). The crude was purified by flash column chromatography (boric acid treated SiO_2; 15:1 pentane:EtOAc) to afford the desired boronic ester **2f** (21 mg, 0.090 mmol, 45%, 5.7:1 d.r., mixture of diastereomers) as a colorless oil.

TLC: R_f = 0.85 (4:1 pentane:EtOAc, CAM).

NMR Spectroscopy (see spectra):

^{1}H NMR: (400 MHz, $CDCl_3$) δ 2.42–2.34 (m, 2H, COC*H*₂), 2.31–2.22 (m, 1H, C*H*₂), 2.18–2.10 (m, 1H, C*H*–Bpin), 1.92–1.84 (m, 1H, C*H*₂), 1.72–1.59 (dq, *J* = 12.4, 3.2 Hz, 1H, C*H*CH₃), 1.47–1.35 (m, 2H, C*H*₂),1.24 (s, 10.3H, 0.86 × 2 × B–OC(C*H*₃)₂), 1.22 (s, 1.7H, 0.14 × 2 × B–OC(C*H*₃)₂), 1.05 (d, *J* = 6.4 Hz, 0.4H, 0.14 × C*H*CH₃), 1.01 (d, *J* = 6.4 Hz, 2.6H, 0.86 × C*H*CH₃) ppm.

^{13}C NMR (major diastereomer): (101 MHz, $CDCl_3$) δ 200.7 ($\underline{C}$=O), 83.6 (B–O$\underline{C}(CH_3)_2$), 45.4 (CO$\underline{C}HCH_3$), 42.8 (CO$\underline{C}H_2$), 38.5, 27.2, 25.2, 24.9 (B–OC($\underline{C}H_3)_2$), 24.8 (B–OC($\underline{C}H_3)_2$), 14.8 (CH$\underline{C}H_3$) ppm. *The carbon attached to boron was not observed due to quadrupolar relaxation.*

HRMS (m/z): (ESI) calculated for $C_{13}H_{24}BO_3$ $[M+H]^+$ 239.1813, found 239.1812.

IR (neat) ν_{max}: 2927, 2855, 1715, 1457, 1411, 1381, 1326, 1265, 1145, 1107, 855, 732 cm^{-1}.

(1*R*,2*R*,5*R*)-5-methyl-2-(4,4,5,5-tetramethyl-1,3,2-dioxaborolan-2-yl)cyclohexan-1-ol (2g)

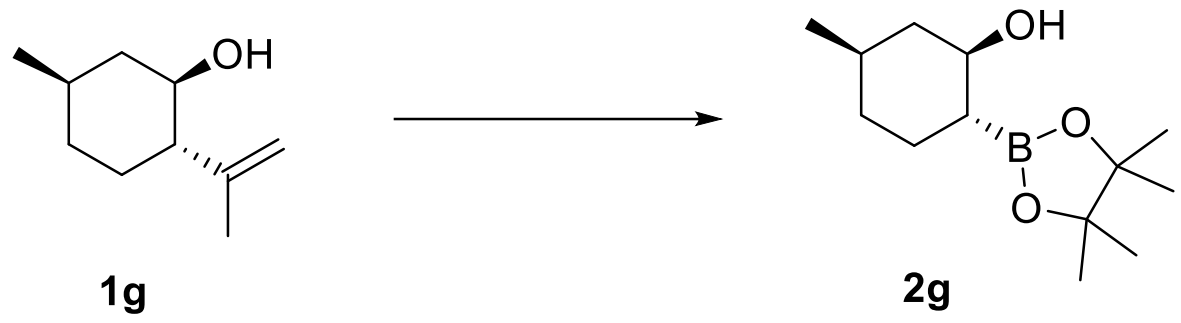

1g **2g**

Prepared following **General Procedure B** with (–)-isopulegol **1g** (30.8 mg, 0.200 mmol, 1.00 equiv). The crude was purified by flash column chromatography (boric acid treated SiO_2; 10:1 pentane:EtOAc) to afford the desired boronic ester **2g** (9.1 mg, 0.038 mmol, 19%, >20:1 d.r.) as a colorless oil.

TLC: R_f = 0.75 (4:1 pentane:EtOAc, CAM).

NMR Spectroscopy (*see spectra*):

^{1}H NMR: (400 MHz, $CDCl_3$) δ 3.57 (br, 1H, C$\underline{H}$OH), 1.98–1.89 (m, 2H, C$\underline{H_2}$–CHOH), 1.67 (br, 1H, O$\underline{H}$), 1.60–1.49 (m, 2H), 1.46–1.32 (m, 2H), 1.25 (s, 12H, 2 × B–OC(C$\underline{H_3})_2$), 0.94 (dt, J = 12.0, 12.0 Hz, 1H, C$\underline{H}$–Bpin), 0.89 (d, J = 6.4 Hz, 3H, CH–C$\underline{H_3}$), 0.74 (dq, J = 13.6, 4.0 Hz ,1H, C$\underline{H}$–CH_3) ppm.

^{13}C NMR: (101 MHz, $CDCl_3$) δ 83.5 (B–O$\underline{C}(CH_3)_2$), 72.5 ($\underline{C}$H–OH), 44.4 ($\underline{C}H_2$CHOH), 33.6, 32.1, 26.5, 24.9 (B–OC($\underline{C}H_3)_2$), 22.7 (CH$\underline{C}H_3$) ppm. *The carbon attached to boron was not observed due to quadrupolar relaxation.*

HRMS (m/z): (ESI) calculated for $C_{13}H_{25}BO_3Na$ $[M+Na]^+$ 263.1789, found 263.1785.

IR (neat) ν_{max}: 3613, 2925, 2855, 1453, 1371, 1326, 1144, 1052, 970, 862, 735 cm^{-1}.

tert-butyldimethyl(((1*R*,2*R*,5*R*)-5-methyl-2-(4,4,5,5-tetramethyl-1,3,2-dioxaborolan-2-yl)cyclohexyl)oxy)silane (2h)

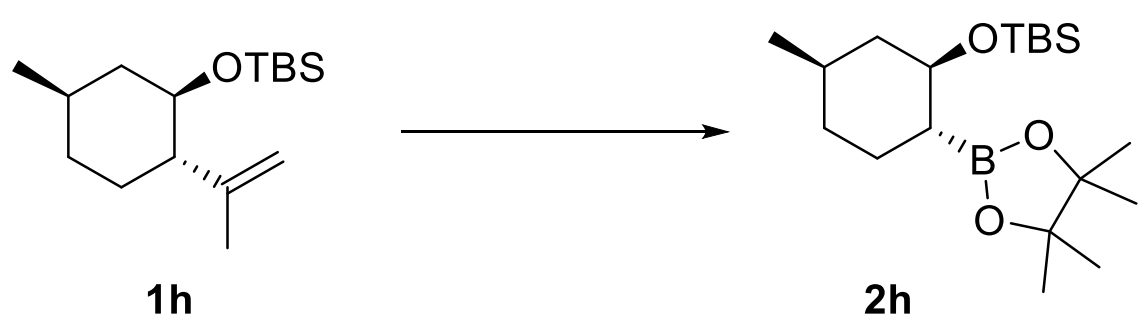

1h **2h**

Prepared following **General Procedure B** with *tert*-butyldimethyl(((1*R*,2*S*,5*R*)-5-methyl-2-(prop-1-en-2-yl)cyclohexyl)oxy)silane **1h** (53.7 mg, 0.200 mmol, 1.00 equiv). The crude was purified by flash column

chromatography (boric acid treated SiO_2; 20:1 pentane:EtOAc) to afford the desired boronic ester **2h** (48 mg, 0.13 mmol, 67%, >20:1 d.r.) as a colorless oil.

TLC: R_f = 0.85 (10:1 pentane:EtOAc, CAM).

NMR Spectroscopy (*see spectra*):

^{1}H NMR: (400 MHz, $CDCl_3$) δ 3.69 (ddd, *J* = 10.8, 10.8, 4.0 Hz, 1H, C*H*–OTBS), 1.91–1.83 (m, 1H, C*H*$_2$CH–OTBS), 1.74–1.65 (m, 1H, C*H*$_2$CH–OTBS), 1.60–1.52 (m, 1H), 1.42–1.32 (m, 1H), 1.24 (s, 6H, B–OC(C*H*$_3$)$_2$), 1.22 (s, 6H, B–OC(C*H*$_3$)$_2$), 1.29–1.14 (m, 1H, C*H*$_2$), 0.93 (ddd, *J* = 13.2, 10.8, 3.2 Hz, 1H, C*H*–Bpin), 0.90–0.82 (m, 13H, CH–C*H*$_3$ + OSi(CH$_3$)$_2$C(C*H*$_3$)$_3$ + C*H*$_2$), 0.80–0.68 (qd, *J* = 12.4, 3.4 Hz, 1H, C*H*–CH$_3$), 0.07 (s, 3H, OSi(C*H*$_3$)$_2$C(CH$_3$)$_3$), 0.06 (s, 3H, OSi(C*H*$_3$)$_2$C(CH$_3$)$_3$) ppm.

^{13}C NMR: (101 MHz, $CDCl_3$) δ 82.9 (B–O*C*(CH$_3$)$_2$), 72.8 (*C*H–OTBS), 46.0, 35.0, 31.7, 26.7, 26.2 (OSi(CH$_3$)$_2$C(*C*H$_3$)$_3$), 25.5 (B–OC(*C*H$_3$)$_2$), 24.6 (B–OC(*C*H$_3$)$_2$), 22.8, 18.2, –3.3 (OSi(*C*H$_3$)$_2$C(CH$_3$)$_3$), –4.0 (OSi(*C*H$_3$)$_2$C(CH$_3$)$_3$) ppm. *The carbon attached to boron was not observed due to quadrupolar relaxation.*

HRMS (m/z): (ESI) calculated for $C_{19}H_{40}BO_3Si$ $[M+H]^+$ 355.2834, found 355.2847.

IR (neat) ν_{max}: 2951, 2927, 2856, 1386, 1319, 1253, 1146, 1095, 1059, 876, 834, 774, 667 cm^{-1}.

(1*S*)-1-methyl-3-(4,4,5,5-tetramethyl-1,3,2-dioxaborolan-2-yl)cyclopentane-1-carbaldehyde (2i)

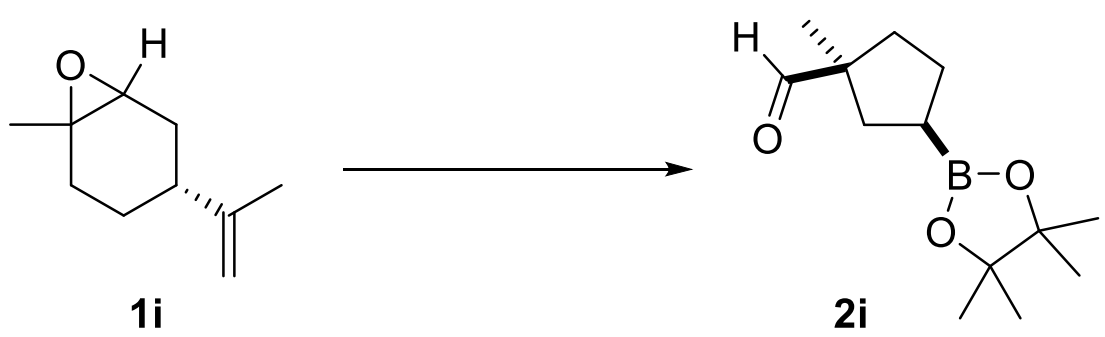

Prepared following **General Procedure B** with (–)-limonene oxide **1i** (1:1 mixture of cis and trans, 30.8 mg, 0.200 mmol, 1.00 equiv). The crude was purified by flash column chromatography (boric acid treated SiO_2; 15:1 pentane:EtOAc) to afford the desired boronic ester **2i** (13 mg, 0.055 mmol, 28%, 4.0:1 d.r., mixture of diastereomers) as a colorless oil.

TLC: R_f = 0.83 (4:1 pentane:EtOAc, CAM).

NMR Spectroscopy (*see spectra*):

^{1}H NMR: (400 MHz, $CDCl_3$) δ 9.48 (s, 0.8H, 0.8 × C*H*O, major diastereomer), 9.46 (s, 0.2H, 0.2 × C*H*O, minor diastereomer), 2.15 (dd, *J* = 12.8, 8.4 Hz, 1H), 2.05–1.93 (m, 1H), 1.91–1.81 (m, 1H), 1.75–1.63 (m, 1H), 1.47–1.28 (m, 3H), 1.26 (s, 2.4H, 0.20 × 2 × B–OC(C*H*$_3$)$_2$, minor diastereomer), 1.24 (s, 9.6H, 0.80 × 2 × B–OC(C*H*$_3$)$_2$, major diastereomer), 1.14 (s, 2.4H, 0.80 × C*H*$_3$, major diastereomer), 1.10 (s, 0.6H, 0.20 × C*H*$_3$, minor diastereomer) ppm.

^{13}C NMR (major diastereomer): (101 MHz, $CDCl_3$) δ 205.2 (*C*=O), 83.3 (B–O*C*(CH$_3$)$_2$), 54.7 (CO*C*HCH$_3$), 37.5, 35.3, 28.1, 25.2 (B–OC(*C*H$_3$)$_2$), 24.9 (B–OC(*C*H$_3$)$_2$), 21.1 (CH*C*H$_3$) ppm. *The carbon attached to boron was not observed due to quadrupolar relaxation.*

HRMS (m/z): (EI) calculated for $C_{13}H_{23}BO_3$ $[M]^+$ 238.1735, found 238.1731.

IR (neat) ν_{max}: 2932, 2862, 1725, 1381, 1319, 1145, 971, 860 ,751 cm^{-1}.

Note: Extending the time of the borylation step to 16 h results in significant amounts of product decomposition due to the instability of the aldehyde functionality to B_2cat_2, leading to <10% yield.

***tert*-butyldimethyl(((1*R*,2*R*,5*R*)-2-methyl-5-(4,4,5,5-tetramethyl-1,3,2-dioxaborolan-2-yl)cyclohexyl)oxy)silane (2j)**

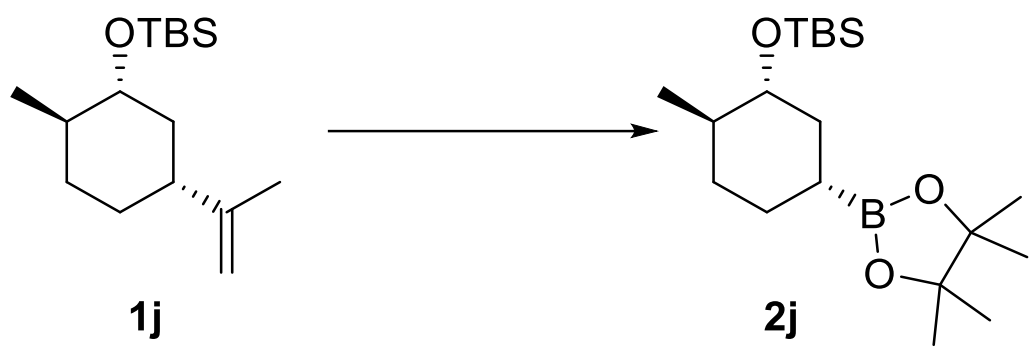

1j **2j**

Prepared following **General Procedure B** with *tert*-butyldimethyl(((1*R*,2*R*,5*R*)-2-methyl-5-(prop-1-en-2-yl)cyclohexyl)oxy)silane **1j** (53.7 mg, 0.200 mmol, 1.00 equiv). The crude was purified by flash column chromatography (boric acid treated SiO_2; 20:1 pentane:EtOAc) to afford the desired boronic ester **2j** (39 mg, 0.11 mmol, 55%, 4.5:1 d.r., mixture of diastereomers) as a colorless oil.

TLC: R_f = 0.92 (10:1 pentane:EtOAc, CAM).

NMR Spectroscopy (<u>*see spectra*</u>):

^{1}H NMR (major diastereomer): (400 MHz, $CDCl_3$) δ 3.06 (ddd, *J* = 10.2, 10.2, 4.4 Hz, 1H, C<u>*H*</u>–OTBS), 1.85–1.78 (m, 1H), 1.73–1.65 (dq, *J* = 13.2, 3.2 Hz, 1H), 1.65–1.58 (m, 1H), 1.39–1.24 (m, 2H), 1.22 (s, 12H, 2 × B–OC(C<u>*H₃*</u>)₂), 1.20–1.10 (m, 2H), 1.02–0.92 (m, 2H), 0.91 (d, *J* = 6.4 Hz, 3H, CHC<u>*H₃*</u>), 0.88 (s, 9H, $OSi(CH_3)_2C(C\underline{H_3})_3$), 0.04 (s, 3H, $OSi(C\underline{H_3})_2C(CH_3)_3$), 0.04 (s, 3H, $OSi(C\underline{H_3})_2C(CH_3)_3$) ppm.

^{13}C NMR (major diastereomer): (101 MHz, $CDCl_3$) δ 83.0 (B–O<u>*C*</u>$(CH_3)_2$), 78.1 (<u>*C*</u>H–OTBS), 40.6 (<u>*C*</u>H_2CH–OTBS), 37.1, 35.3, 27.4, 26.1 ($OSi(CH_3)_2\underline{C}(CH_3)_3$), 24.9 (B–OC(<u>*C*</u>$H_3)_2$), 24.8 (B–OC(<u>*C*</u>$H_3)_2$), 19.6 ,18.3 , –3.7 ($OSi(\underline{C}H_3)_2C(CH_3)_3$), –4.5 ($OSi(\underline{C}H_3)_2C(CH_3)_3$) ppm. *The carbon attached to boron was not observed due to quadrupolar relaxation.*

HRMS (m/z): (ESI) calculated for $C_{19}H_{40}BO_3Si$ $[M+H]^+$ 355.2834, found 355.2835.

IR (neat) ν_{max}: 2928, 2856, 1727, 1384, 1316, 1255, 1146, 1079, 937, 859, 835, 773, 668 cm^{-1}.

(4*R*,4a*S*,6*R*)-4,4a-dimethyl-6-(4,4,5,5-tetramethyl-1,3,2-dioxaborolan-2-yl)-4,4a,5,6,7,8-hexahydronaphthalen-2(3*H*)-one (2k)

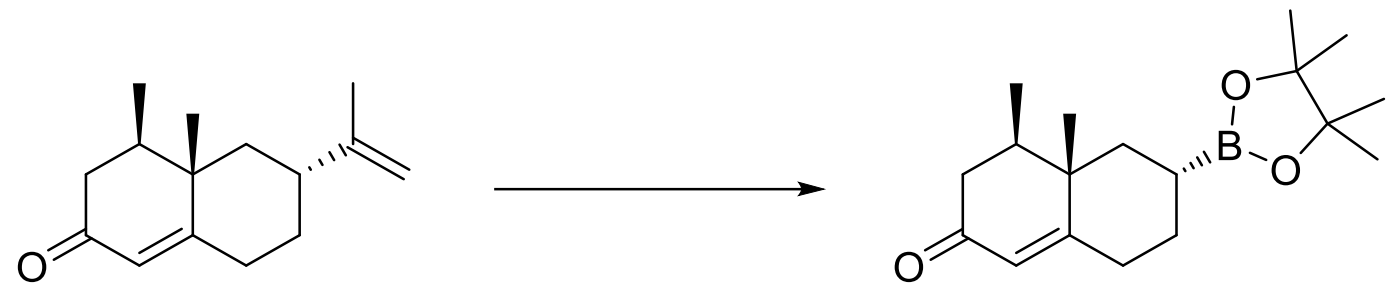

1k **2k**

Prepared following **General Procedure B** with (+)-nootkatone **1k** (43.8 mg, 0.200 mmol, 1.00 equiv) with controlled ozonolysis (50% output level for 120 s). The crude was purified by flash column chromatography (boric acid treated SiO_2; 4:1 pentane:EtOAc) to afford the desired boronic ester **2k** (37 mg, 0.12 mmol, 60%, >20:1 d.r.) as an off-white solid.

TLC: R_f = 0.53 (4:1 pentane:EtOAc, CAM).

NMR Spectroscopy (*see spectra*):

^{1}H NMR: (400 MHz, $CDCl_3$) δ 5.76 (s, 1H, C*H*=C), 2.46–2.24 (m, 4H, COC*H₂* + CH=CC*H₂*), 2.02–1.91 (m, 3H, C*H*CH₃ + C*H₂*)), 1.41–1.32 (m, 2H, C*H₂*), 1.25 (s, 12H, 2 × B–OC(C*H₃*)₂), 1.18–1.10 (m, 1H, C*H*–Bpin), 1.07 (s, 3H, CC*H₃*) 0.95 (d, *J* = 6.8 Hz, 3H, CHC*H₃*) ppm.

^{13}C NMR: (101 MHz, $CDCl_3$) δ 200.6 (*C*=O), 173.4 (CH=*C*), 124.5 (*C*H=C), 83.5 (B–O*C*(CH₃)₂), 42.4 (CO*C*H₂), 40.2 (*C*CH₃), 40.0 (*C*HCH₃), 39.4 (CH=C*C*H₂), 34.0, 28.3, 24.9 (B–OC(*C*H₃)₂), 16.3 (C*C*H₃), 15.1(CH*C*H₃) ppm. *The carbon attached to boron was not observed due to quadrupolar relaxation.*

HRMS (m/z): (ESI) calculated for $C_{18}H_{30}BO_3$ $[M+H]^+$ 305.2283, found 305.2274.

IR (neat) ν_{max}: 2964, 1716, 1649, 1469, 1362, 1261, 1096, 1023, 799, 764, 749 cm^{-1}.

All recorded spectroscopic data matched those previously reported in the literature.[16]

Note: Inseparable mixture with catechol impurity was present in the spectra. Yield was calculated by integration of the ^{1}H NMR.

4,4,5,5-tetramethyl-2-((4*S*,8*R*)-4,8,12-trimethyltridecyl)-1,3,2-dioxaborolane (2l)

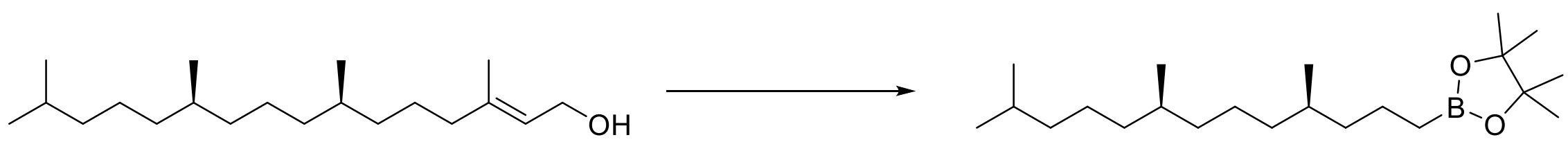

1l **2l**

Prepared following **General Procedure B** with (7*R*,11*R*,*E*)-3,7,11,15-tetramethylhexadec-2-en-1-ol **1l** (59.3 mg, 0.200 mmol, 1.00 equiv). The crude was purified by flash column chromatography (SiO_2; 25:1 pentane:EtOAc) to afford the desired boronic ester (48 mg, 0.14 mmol, 68%) as a colorless oil.

For 2 mmol scale reaction: Prepared following a modified **General Procedure B** with (7*R*,11*R*,*E*)-3,7,11,15-tetramethylhexadec-2-en-1-ol **1l** (0.59 g, 2.0 mmol, 1.0 equiv). During the evaporation of MeOH after ozonolysis,

the solvent was not removed to dryness and instead anhydrous THF (3 × 10 mL) was used to gradually replace the solvent. The crude was purified by flash column chromatography (SiO_2; 25:1 pentane:EtOAc) to afford the desired boronic ester (0.47 g, 1.3 mmol, 67%) as a colorless oil.

TLC: R_f = 0.85 (10:1 pentane:EtOAc, CAM).

NMR Spectroscopy (*see spectra*):

^{1}H NMR: (400 MHz, $CDCl_3$) δ 1.57–1.46 (m, 1H, $(CH_3)_2C\underline{H}CH_2$), 1.42–1.00 (m, 18H, 2 × C<u>*H*</u> + 8 × C<u>*H₂*</u>), 1.24 (s, 12H, 2 × B–OC($C\underline{H_3}$)$_2$), 0.86 (d, *J* = 6.4 Hz, 6H, 2 × $C\underline{H_3}$), 0.83 (d, *J* = 6.4 Hz, 6H, 2 × $C\underline{H_3}$), 0.75 (t, *J* = 7.8 Hz, 2H, $C\underline{H_2}$–Bpin) ppm.

^{13}C NMR: (101 MHz, $CDCl_3$) δ 83.0 ($O\underline{C}(CH_3)_2$), 40.2, 40.1, 39.5, 37.6, 37.5, 37.4, 33.0 ($CH\underline{C}H_3$), 32.8 ($CH\underline{C}H_3$), 28.1 ($\underline{C}(CH_3)_2$), 25.0(B–OC($\underline{C}H_3$)$_2$), 24.6, 22.9 ($C(\underline{C}H_3)_2$), 22.8 ($C(\underline{C}H_3)_2$), 21.6, 19.9 ($CH\underline{C}H_3$), 19.9 ($CH\underline{C}H_3$) ppm. *The carbon attached to boron was not observed due to quadrupolar relaxation.*

HRMS (m/z): (ESI) calculated for $C_{22}H_{46}BO_2$ $[M+H]^+$ 355.3585, found 355.3599.

IR (neat) ν_{max}: 2953, 2925, 2868, 1463, 1375, 1317, 1146, 969, 847, 731, 672, 578 cm^{-1}.

4,4,5,5-tetramethyl-2-(2-((1*r*,1'*r*,4*R*,4'*R*)-4'-(*p*-tolyl)-[1,1'-bi(cyclohexan)]-4-yl)ethyl)-1,3,2-dioxaborolane (2m)

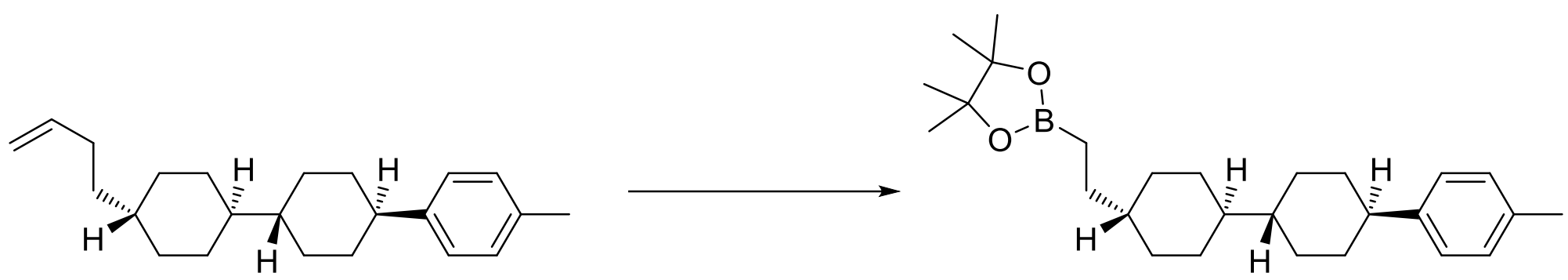

1m **2m**

Prepared following **General Procedure B** (Note: 2 mL anhydrous DCM was used together with the 4 mL of methanol for the ozonolysis to dissolve the starting material) with (1*s*,1'*r*,4*R*,4'*R*)-4-(but-3-en-1-yl)-4'-(*p*-tolyl)-1,1'-bi(cyclohexane) **1m** (62.1 mg, 0.200 mmol, 1.00 equiv). The crude was purified by flash column chromatography (boric acid treated SiO_2; 30:1 pentane:EtOAc) to afford the desired boronic ester (51 mg, 0.12 mmol, 62%) as a white solid.

TLC: R_f = 0.82 (10:1 pentane:EtOAc, CAM).

NMR Spectroscopy (*see spectra*):

^{1}H NMR: (400 MHz, $CDCl_3$) δ 7.10(s, 4H, 4 × Ar<u>*H*</u>), 2.40 (tt, *J* =12.0, 3,6 Hz, 1H, pinB–$CH_2CH_2C\underline{H}$), 2.31 (s, 3H, Ar–$C\underline{H_3}$), 1.93–1.86 (m, 2H), 1.85–1.70 (m, 6H), 1.47–1.35 (m, 2H), 1.34–1.26 (m, 2H), 1.25 (s, 12H, 2 × B–OC($C\underline{H_3}$)$_2$), 1.17–1.06 (m, 4H), 1.05–0.92 (m, 3H, 3 × C<u>*H*</u>), 0.89–0.81 (t, 2H, *J* = 12.0 Hz, $C\underline{H_2}$–Bpin), 0.80–0.73 (t, 2H, *J* = 8.0 Hz) ppm.

^{13}C NMR: (101 MHz, $CDCl_3$) δ 145.1, 135.3, 129.1, 126.8, 83.0 (B–$O\underline{C}(CH_3)_2$), 44.4, 43.6, 43.1, 40.4, 34.9, 33.3, 31.5, 30.6, 30.2, 25.0 (B–OC($\underline{C}H_3$)$_2$), 21.1 ($\underline{C}H_2CH_2$–Bpin) ppm. *The carbon attached to boron was*

not observed due to quadrupolar relaxation.

HRMS (m/z): (ESI) calculated for $C_{27}H_{44}BO_2$ $[M+H]^+$ 411.3356, found 411.3362.

IR (neat) ν_{max}: 2977, 2918, 2848, 1515, 1447, 1371, 1315, 1143, 969, 848, 812, 534 cm^{-1}.

4-(2,2-difluorobenzo[*d*][1,3]dioxol-4-yl)-1-(3-(4,4,5,5-tetramethyl-1,3,2-dioxaborolan-2-yl)propyl)-1*H*-pyrrole-3-carbonitrile (2n)

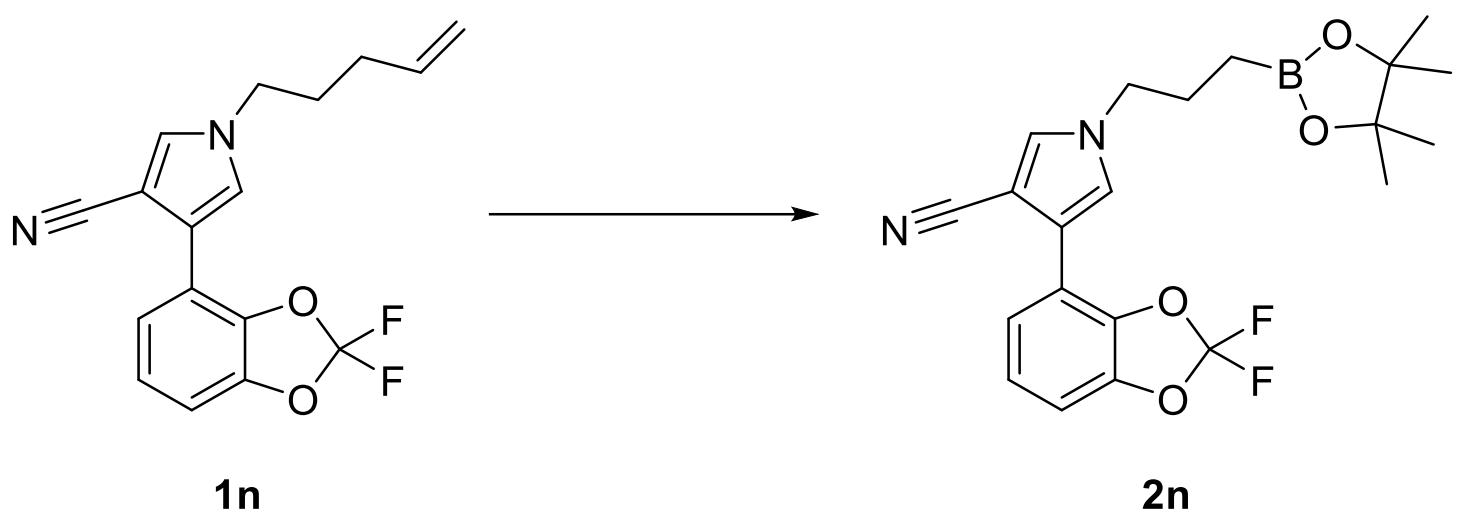

Prepared following **General Procedure B** with 4-(2,2-difluorobenzo[*d*][1,3]dioxol-4-yl)-1-(pent-4-en-1-yl)-1*H*-pyrrole-3-carbonitrile **1n** (63.3 mg, 0.200 mmol, 1.00 equiv). The crude was purified by preparative thin layer chromatography (6:1 pentane:EtOAc) to afford the desired boronic ester (39 mg, 0.094 mmol, 47%) as a white solid.

TLC: R_f = 0.55 (4:1 pentane:EtOAc, CAM).

NMR Spectroscopy (*see spectra*):

^{1}H NMR: (400 MHz, $CDCl_3$) δ 7.73–7.69 (dd, *J* = 8.0, 1.2 Hz, 1H, Ar*H*), 7.25–7.22 (d, 1H, *J* = 2.4 Hz, Ar*H*), 7.17–7.15 (d, 1H, *J* = 2.4 Hz, Ar*H*), 7.15–7.10 (t, 1H, *J* = 8.0 Hz, Ar*H*), 6.97–6.93 (dd, *J* = 8.0, 1.2 Hz, 1H, Ar*H*), 3.98–3.92 (t, *J* = 7.2 Hz, 2H, N–CH_2), 1.97–1.88 (tt, *J* = 7.2, 7.2 Hz, 2H, CH_2), 1.25 (s, 12H, 2 × B–OC(CH_3)$_2$), 0.81–0.75 (t, 2H, *J*= 12.0 Hz, CH_2–Bpin) ppm.

^{13}C NMR: (101 MHz, $CDCl_3$) δ 143.9, 139.9, 131.5 (t, *J* = 256 Hz, *C*F_2), 129.8, 124.2, 122.7, 121.3, 118.5, 116.8, 115.6, 107.8, 91.0, 83.6 (B–O*C*(CH_3)$_2$), 52.5, 25.8 (B–OC(*C*H_3)$_2$), 25.2 (B–OC(*C*H_3)$_2$), 25.0 ppm.

^{19}F NMR: (377 MHz, $CDCl_3$) δ –49.5 ppm.

HRMS (m/z): (MALDI) calculated for $C_{21}H_{23}BF_2N_2O_4Na$ $[M+Na]^+$ 439.615, found 439.1621.

IR (neat) ν_{max}: 3133, 2978, 2933, 2223, 1652, 1527, 1455, 1327, 1246, 1144, 1033, 721, 616 cm^{-1}.

(8*S*,9*S*,10*R*,13*R*,14*S*,17*S*)-10,13-dimethyl-17-(4,4,5,5-tetramethyl-1,3,2-dioxaborolan-2-yl)-1,2,6,7,8,9,10,11,12,13,14,15,16,17-tetradecahydro-3*H*-cyclopenta[*a*]phenanthren-3-one (2o)

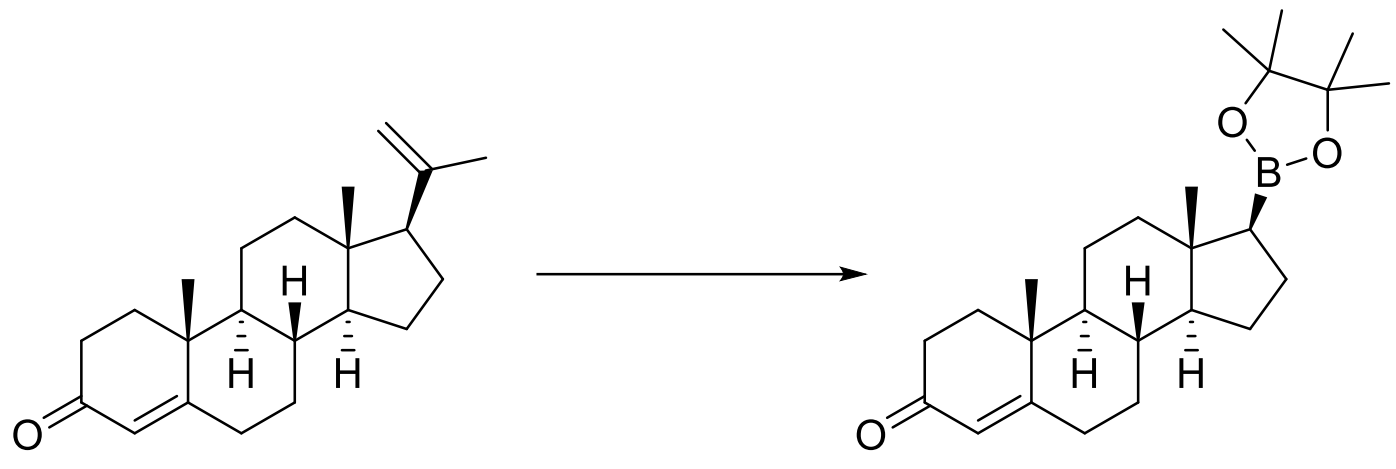

1o **2o**

Prepared following **General Procedure B** (Note: 2 mL anhydrous DCM was used together with the 4 mL of methanol for the ozonolysis to dissolve the starting material) with (8*S*,9*S*,10*R*,13*S*,14*S*,17*R*)-10,13-dimethyl-17-(prop-1-en-2-yl)-1,2,6,7,8,9,10,11,12,13,14,15,16,17-tetradecahydro-3*H*-cyclopenta[*a*]phenanthren-3-one **1o** (31.2 mg, 0.100 mmol, 1.00 equiv) with controlled ozonolysis (50% output level for 120 s). The crude was purified by flash column chromatography (boric acid treated SiO_2; 20:1 pentane:EtOAc) to afford the desired boronic ester **2o** (18 mg, 0.044 mmol, 44%, >20:1 d.r.) as a white solid.

TLC: R_f = 0.66 (4:1 pentane:EtOAc, CAM).

NMR Spectroscopy (*see spectra*):

^{1}H NMR: (400 MHz, $CDCl_3$) δ 5.73 (s, 1H, C*H*=C–C=O), 2.46–2.21 (m, 4H), 2.07–1.98 (m, 1H), 1.98–1.91 (dt, *J* = 12.4, 4.0 Hz, 1H), 1.89–1.82 (m, 1H), 1.81–1.65 (m, 4H), 1.53–1.36 (m, 3H), 1.29–1.20 (m, 2H), 1.24 (s, 6H, B–OC(CH_3)$_2$), 1.24 (s, 6H, B–OC(CH_3)$_2$), 1.18 (s, 3H, CH_3), 1.11–1.03 (m, 1H, C*H*), 1.02–0.91 (m, 2H, 2 × C*H*), 1.11–1.03 (m, 1H, C*H*–Bpin), 0.60 (s, 3H, CH_3) ppm.

^{13}C NMR: (151 MHz, $CDCl_3$) δ 199.9 (CH=C–*C*=O), 171.9 (*C*H=C–C=O), 123.9 (CH=*C*–C=O), 82.9 (B–O*C*(CH_3)$_2$), 57.1, 54.1, 43.5, 38.9, 38.8, 36.1, 35.9, 34.1, 33.2, 32.5, 25.9, 25.3 (B–OC(*C*H_3)$_2$), 24.9(B–OC(*C*H_3)$_2$), 23.4, 21.1, 17.5, 15.6 ppm. *The carbon attached to boron was not observed due to quadrupolar relaxation.*

HRMS (m/z): (ESI) calculated for $C_{25}H_{40}BO_3$ $[M+H]^+$ 399.3065, found 399.3064.

IR (neat) ν_{max}: 2928, 2853, 1677, 1616, 1370, 1275, 1261, 1144, 764, 750 cm^{-1}.

methyl (1*R*,3a*S*,5a*R*,5b*R*,7a*R*,9*S*,11a*R*,11b*R*,13a*R*,13b*R*)-9-hydroxy-5a,5b,8,8,11a-pentamethyl-1-(4,4,5,5-tetramethyl-1,3,2-dioxaborolan-2-yl)icosahydro-3a*H*-cyclopenta[*a*]chrysene-3a-carboxylate (2p)

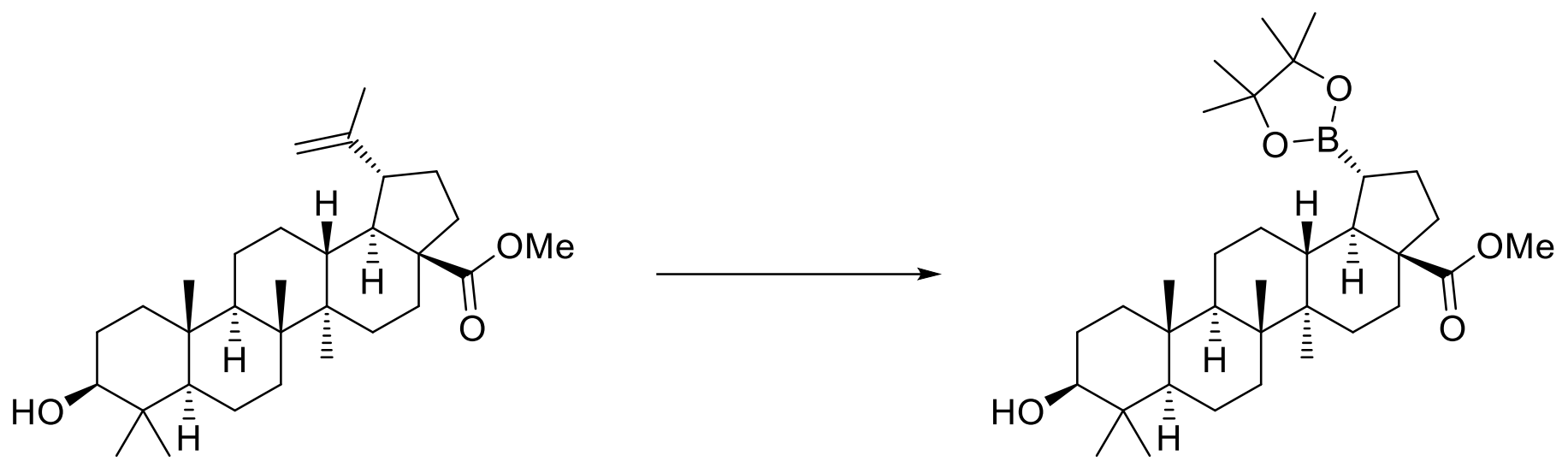

1p **2p**

Prepared following **General Procedure B** (Note: 2 mL anhydrous DCM was used together with the 4 mL of methanol for the ozonolysis to dissolve the starting material) with methyl (1*R*,3a*S*,5a*R*,5b*R*,7a*R*,9*S*,11a*R*,11b*R*,13a*R*,13b*R*)-9-hydroxy-5a,5b,8,8,11a-pentamethyl-1-(prop-1-en-2-yl)icosahydro-3a*H*-cyclopenta[*a*]chrysene-3a-carboxylate **1p** (47.1 mg, 0.100 mmol, 1.00 equiv). The crude was purified by flash column chromatography (boric acid treated SiO_2; 50:1 pentane:EtOAc) to afford the desired boronic ester **2p** (25 mg, 0.046 mmol, 46%, >20:1 d.r.) as a white solid.

TLC: R_f = 0.78 (4:1 pentane:EtOAc, CAM).

NMR Spectroscopy (*see spectra*):

^{1}H NMR: (400 MHz, $CDCl_3$) δ 3.62 (s, 3H, COOC*H₃*), 3.19 (dd, *J* = 11.2, 5.2 Hz, 1H, C*H*–OH), 2.23–2.16 (m, 1H), 2.11–2.04 (m, 1H), 1.97 (dd, *J* = 11.8, 8.0 Hz, 1H), 1.81–1.74 (m, 1H), 1.72–1.65 (m, 1H), 1.64–1.56 (m, 3H), 1.55–1.49 (m, 7H), 1.48–1.34 (m, 8H), 1.23 (s, 6H, B–OC(CH_3)$_2$), 1.22 (s, 6H, B–OC(CH_3)$_2$), 1.14–1.08 (m, 2H), 0.96 (s, 3H, C(CH_3)$_2$), 0.95 (s, 3H, C(CH_3)$_2$), 0.91 (s, 3H, CH_3), 0.82 (s, 3H, CH_3), 0.76 (s, 3H, CH_3), 0.69 (m, 1H) ppm.

^{13}C NMR: (101 MHz, $CDCl_3$) δ 176.9 (*C*=O), 82.9 (B–O*C*(CH_3)$_2$), 79.2 (*C*H–OH), 56.4, 55.6, 51.4 51.2, 50.6, 42.3, 40.8, 39.7, 39.0, 39.0, 38.1, 37.7, 37.4, 34.3, 32.0, 29.8, 28.1, 27.5, 25.6, 24.8 (B–OC(CH_3)$_2$), 24.8 (B–OC(CH_3)$_2$), 21.1, 18.5, 16.3, 16.1, 15.5, 14.9 ppm. *The carbon attached to boron was not observed due to quadrupolar relaxation.*

HRMS (m/z): (ESI) calculated for $C_{34}H_{58}BO_5$ $[M+H]^+$ 557.4372, found 557.4345.

IR (neat) ν_{max}: 3368, 2944, 2867, 1725, 1381, 1261, 1145, 1103, 1017, 799, 764, 750 cm^{-1}.

methyl (1*R*,3a*S*,5a*R*,5b*R*,7a*R*,9*S*,11a*R*,11b*R*,13a*R*,13b*R*)-9-((*tert*-butyldimethylsilyl)oxy)-5a,5b,8,8,11a-pentamethyl-1-(4,4,5,5-tetramethyl-1,3,2-dioxaborolan-2-yl)icosahydro-3a*H*-cyclopenta[*a*]chrysene-3a-carboxylate (2q)

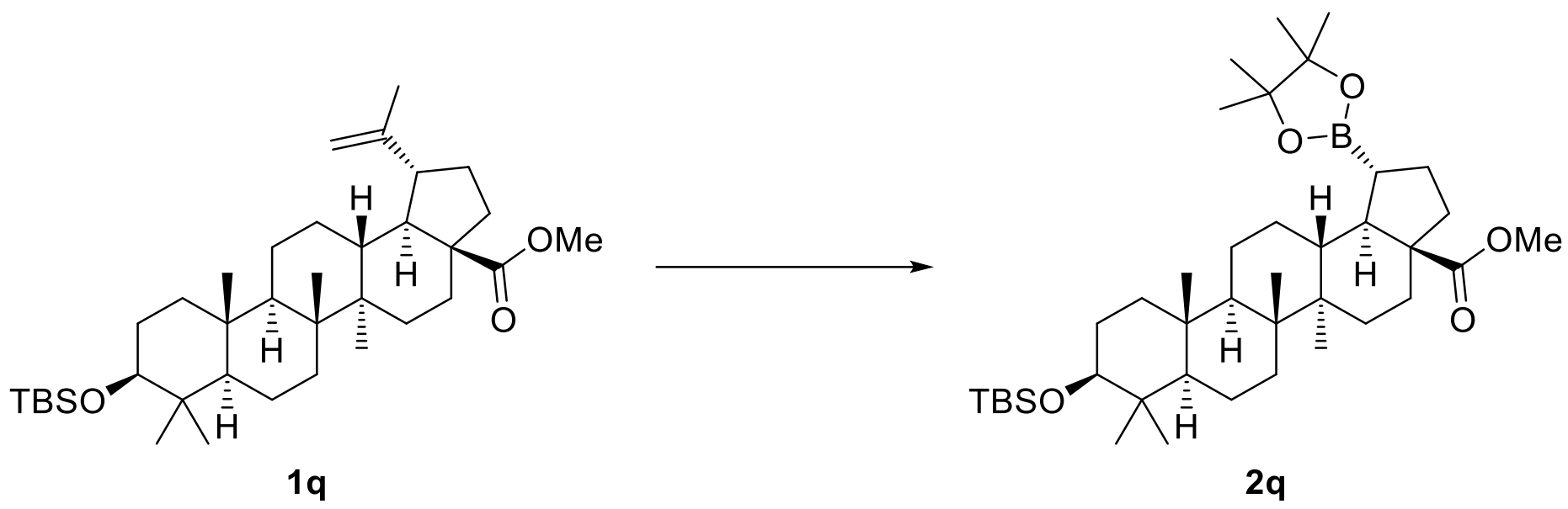

Prepared following **General Procedure B** (Note: 2 mL anhydrous DCM was used together with the 4 mL of methanol for the ozonolysis to dissolve the starting material) with methyl (1*R*,3a*S*,5a*R*,5b*R*,7a*R*,9*S*,11a*R*,11b*R*,13a*R*,13b*R*)-9-((tert-butyldimethylsilyl)oxy)-5a,5b,8,8,11a-pentamethyl-1-(prop-1-en-2-yl)icosahydro-3a*H*-cyclopenta[*a*]chrysene-3a-carboxylate **1q** (58.5 mg, 0.100 mmol, 1.00 equiv). The crude was purified by flash column chromatography (boric acid treated SiO_2; 75:1 pentane:EtOAc) to afford the desired boronic ester **2q** (36 mg, 0.053 mmol, 53%, >20:1 d.r.) as a white solid.

TLC: R_f = 0.47 (10:1 pentane:EtOAc, CAM).

NMR Spectroscopy (*see spectra*):

^{1}H NMR: (400 MHz, $CDCl_3$) δ 3.62 (s, 3H, COOC*H*$_3$), 3.15 (dd, *J* = 11.4, 4.8 Hz, 1H, C*H*–OTBS), 2.22–2.15 (m, 1H), 2.11–2.02 (m, 1H), 1.96 (dd, *J* = 11.8, 7.6 Hz, 1H), 1.83–1.70 (m, 1H), 1.67–1.57 (m, 4H), 1.55–1.41 (m, 7H), 1.39–1.28 (m, 7H), 1.22 (s, 6H, B–OC(C*H*$_3$)$_2$), 1.21 (s, 6H, B–OC(C*H*$_3$)$_2$), 1.16–1.07 (m, 2H), 0.94 (s, 3H, C(C*H*$_3$)$_2$), 0.90 (s, 3H, C(C*H*$_3$)$_2$), 0.88 (s, 9H, OSi(CH$_3$)$_2$C(C*H*$_3$)$_3$), 0.87 (s, 3H, C*H*$_3$), 0.82 (s, 3H, C*H*$_3$), 0.72 (s, 3H, C*H*$_3$), 0.69–0.62 (m, 1H), 0.01 (s, 6H, OSi(C*H*$_3$)$_2$C(CH$_3$)$_3$) ppm.

^{13}C NMR: (101 MHz, $CDCl_3$) δ 176.9 (*C*=O), 82.9 (B–O*C*(CH$_3$)$_2$), 79.7 (*C*H–OTBS), 56.4, 55.7, 51.4, 51.2, 50.7, 42.2, 40.8, 39.6, 39.0, 38.1, 37.7, 37.3, 34.5, 32.0, 29.8, 28.6, 27.9, 27.7, 26.1 (OSi(CH$_3$)$_2$C(*C*H$_3$)$_3$), 25.6, 24.8 (B–OC(*C*H$_3$)$_2$), 24.8 (B–OC(*C*H$_3$)$_2$), 21.1, 18.7, 18.3, 16.3, 16.0, 16.0, 14.9, –3.6, –4.8 ppm. *The carbon attached to boron was not observed due to quadrupolar relaxation.*

HRMS (m/z): (MALDI) calculated for $C_{40}H_{71}BO_5SiNa$ [M+Na]$^+$ 693.5063, found 693.5069.

IR (neat) ν_{max}**:** 2945, 2858, 1729, 1462, 1379, 1275, 1258, 1146, 1108, 885, 836, 765, 750, 676 cm^{-1}.

***tert*-butyl (1*R*,3a*S*,5a*R*,5b*R*,7a*R*,9*S*,11a*R*,11b*R*,13a*R*,13b*R*)-9-((*tert*-butyldimethylsilyl)oxy)-5a,5b,8,8,11a-pentamethyl-1-(4,4,5,5-tetramethyl-1,3,2-dioxaborolan-2-yl)icosahydro-3a*H*-cyclopenta[*a*]chrysene-3a-carboxylate (2r)**

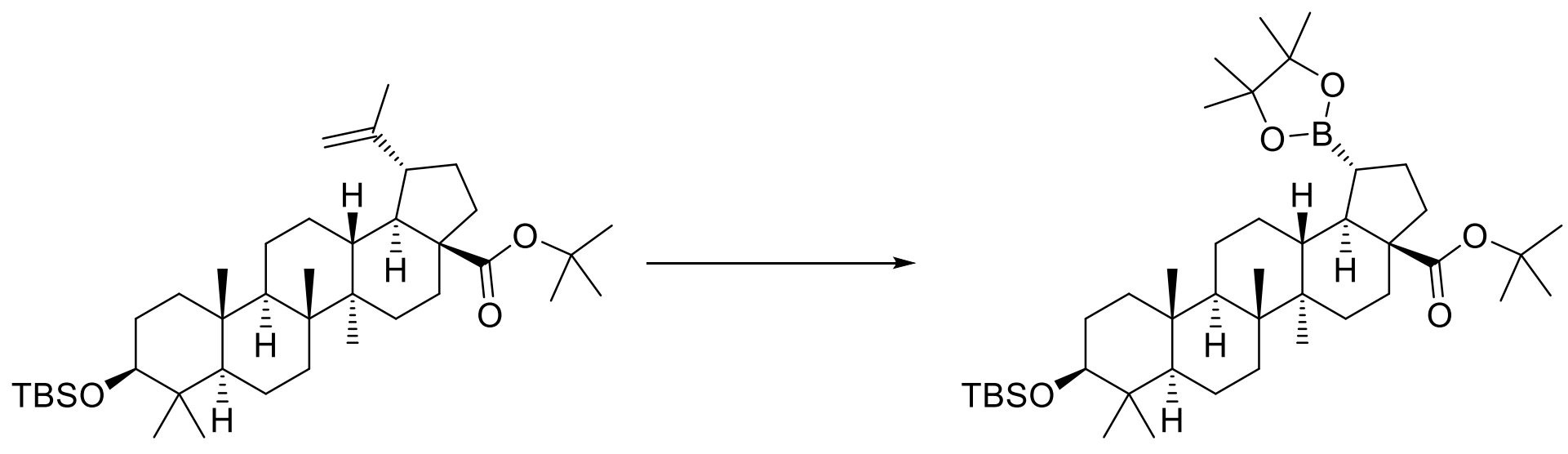

1r **2r**

Prepared following **General Procedure B** (Note: 2 mL anhydrous DCM was used together with the 4 mL of methanol for the ozonolysis to dissolve the starting material) with *tert*-butyl (1*R*,3a*S*,5a*R*,5b*R*,7a*R*,9*S*,11a*R*,11b*R*,13a*R*,13b*R*)-9-((*tert*-butyldimethylsilyl)oxy)-5a,5b,8,8,11a-pentamethyl-1-(prop-1-en-2-yl)icosahydro-3a*H*-cyclopenta[*a*]chrysene-3a-carboxylate **1r** (62.6 mg, 0.100 mmol, 1.00 equiv). The crude was purified by flash column chromatography (boric acid treated SiO_2; 90:1 pentane:EtOAc) to afford the desired boronic ester **2r** (39 mg, 0.054 mmol, 54%, >20:1 d.r.) as a white solid.

For 2 mmol scale reaction: Prepared following a modified **General Procedure B** with *tert*-butyl (1*R*,3a*S*,5a*R*,5b*R*,7a*R*,9*S*,11a*R*,11b*R*,13a*R*,13b*R*)-9-((*tert*-butyldimethylsilyl)oxy)-5a,5b,8,8,11a-pentamethyl-1-(prop-1-en-2-yl)icosahydro-3a*H*-cyclopenta[*a*]chrysene-3a-carboxylate **1r** (1.25 g, 2.00 mmol, 1.00 equiv). During the evaporation of MeOH after ozonolysis, the solvent was not removed to dryness and instead anhydrous THF (3 × 10 mL) was used to gradually replace the solvent. The crude was purified by flash column chromatography (boric acid treated SiO_2; 90:1 pentane:EtOAc) to afford the desired boronic ester **2r** (0.66 g, 0.92 mmol, 46%, >20:1 d.r.) as a white solid.

TLC: R_f = 0.47 (10:1 pentane:EtOAc, CAM).

NMR Spectroscopy (*see spectra*):

^{1}H NMR: (400 MHz, $CDCl_3$) δ 3.15 (dd, *J* = 11.4, 4.4 Hz, 1H, C*H*–OTBS), 2.22–2.10 (m, 2H), 1.93 (dd, *J* = 12.2, 8.0 Hz, 1H), 1.82–1.71 (m, 1H), 1.67–1.44 (m, 7H), 1.43 (s, 9H, COOC(C*H*$_3$)$_3$), 1.40–1.23 (m, 9H), 1.22 (s, 6H, B–OC(C*H*$_3$)$_2$), 1.21 (s, 6H, B–OC(C*H*$_3$)$_2$), 1.18–1.07 (m, 4H), 0.94 (s, 3H, C(C*H*$_3$)$_2$), 0.93 (s, 3H, C(C*H*$_3$)$_2$), 0.88 (s, 9H, OSi(CH$_3$)$_2$C(C*H*$_3$)$_3$), 0.87 (s, 3H, C*H*$_3$), 0.82 (s, 3H, C*H*$_3$), 0.72 (s, 3H, C*H*$_3$), 0.69–0.64 (m, 1H), 0.01 (s, 6H, OSi(C*H*$_3$)$_2$C(CH$_3$)$_3$) ppm.

^{13}C NMR: (126 MHz, $CDCl_3$) δ 175.9 (*C*=O), 82.8 (B–O*C*(CH$_3$)$_2$), 79.7 (COO*C*(CH$_3$)$_3$), 79.6 (*C*H–OTBS), 56.7, 55.7, 51.0, 50.9, 42.4, 40.9, 39.6, 39.0, 38.5, 37.7, 37.3, 34.5, 32.3, 29.8, 28.6, 28.3, 28.2, 28.0, 27.6, 26.1 (OSi(CH$_3$)$_2$C(*C*H$_3$)$_3$), 25.7, 24.8 (B–OC(*C*H$_3$)$_2$), 24.8 (B–OC(*C*H$_3$)$_2$), 21.2, 18.7, 18.3, 16.3, 16.2, 16.0, 14.9, –3.6 (OSi(*C*H$_3$)$_2$C(CH$_3$)$_3$), –4.8 (OSi(*C*H$_3$)$_2$C(CH$_3$)$_3$) ppm. *The carbon attached to boron was not observed due to quadrupolar relaxation.*

HRMS (m/z): (MALDI) calculated for $C_{43}H_{77}BO_5SiNa$ $[M+Na]^+$ 735.5562, found 735.5572.

IR (neat) ν_{max}: 2940, 2860, 1722, 1462, 1387, 1368, 1251, 1144, 1108, 1064, 886, 849, 836, 774 cm^{-1}.

methyl 6-(4,4,5,5-tetramethyl-1,3,2-dioxaborolan-2-yl)hexanoate (4a)

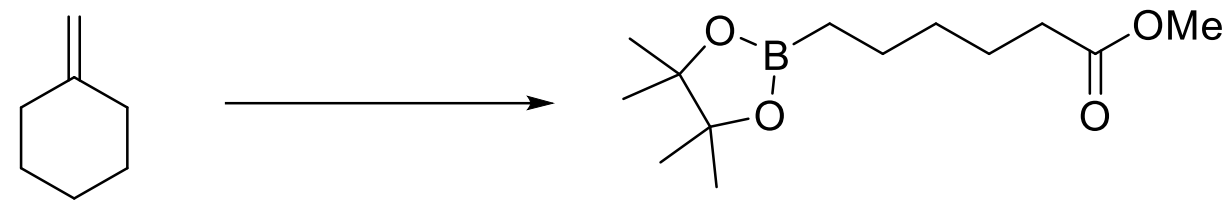

3a **4a**

Prepared following **General Procedure C** with methylenecyclohexane **3a** (19.2 mg, 0.200 mmol, 1.00 equiv). The crude was purified by flash column chromatography (SiO_2; 16:1 pentane:EtOAc) to afford the desired boronic ester **4a** (39 mg, 0.15 mmol, 76%) as a colorless oil.

TLC: R_f = 0.30 (15:1 pentane:EtOAc, CAM).

NMR Spectroscopy (*see spectra*):

^{1}H NMR: (400 MHz, $CDCl_3$) δ 3.64 (s, 3H, COOC*H*$_3$), 2.28 (t, *J* = 7.2 Hz, 2H, C*H*$_2$COOCH$_3$), 1.61 (tt, *J* = 7.2, 7.2 Hz, 2H), 1.45–1.36 (m, 2H), 1.36–1.25 (m, 2H),1.22 (s, 12H, 2 × B–OC(C*H*$_3$)$_2$), 0.75 (t, *J* = 8.0 Hz, 2H, C*H*$_2$–Bpin) ppm.

^{13}C NMR: (101 MHz, $CDCl_3$) δ 174.4 (*C*=O), 83.0 (B–O*C*(CH$_3$)$_2$), 51.5 (COO*C*H$_3$), 34.2, 32.0, 24.9 (B–OC(*C*H$_3$)$_2$), 24.9, 23.7 ppm. *The carbon attached to boron was not observed due to quadrupolar relaxation.*

All recorded spectroscopic data matched those previously reported in the literature.[17]

methyl 3-((*tert*-butoxycarbonyl)(2-(4,4,5,5-tetramethyl-1,3,2-dioxaborolan-2-yl)ethyl)amino)propanoate (4b)

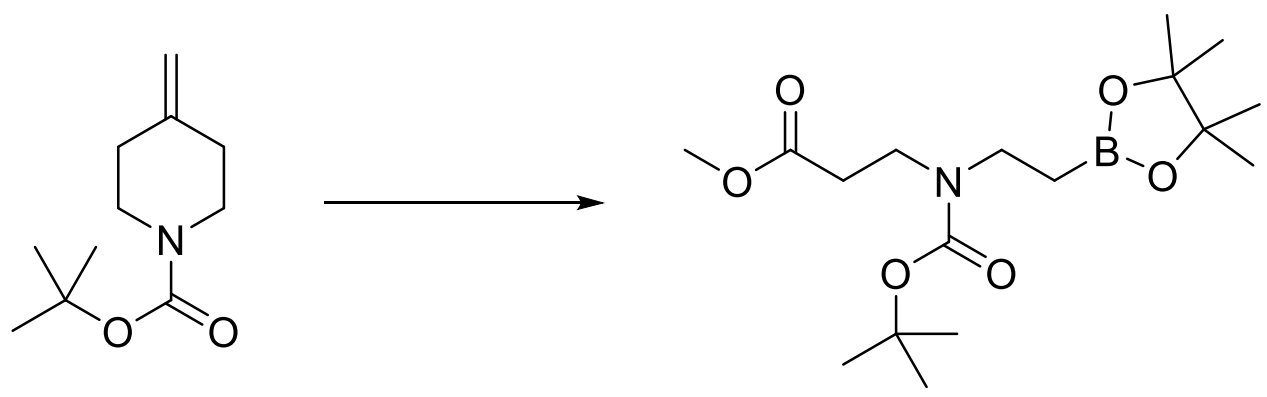

3b **4b**

Prepared following **General Procedure C** with *tert*-butyl 4-methylenepiperidine-1-carboxylate **3b** (39.5 mg, 0.200 mmol, 1.00 equiv). The crude was purified by flash column chromatography (boric acid treated SiO_2; 8:1 pentane:EtOAc) to afford the desired boronic ester (45 mg, 0.13 mmol, 63%) as a colorless oil.

TLC: R_f = 0.45 (3:1 pentane:EtOAc, CAM).

NMR Spectroscopy (*see spectra*):

^{1}H NMR: (400 MHz, $CDCl_3$) δ 3.67 (s, 3H, COOC*H*$_3$), 3.47 (br, 2H, N–C*H*$_2$), 3.30 (br, 2H, N–C*H*$_2$), 2.56 (t, *J* = 7.2 Hz, 2H, C*H*$_2$), 1.44 (s, 9H, OC(C*H*$_3$)$_3$),1.23 (s, 12H, 2 × B–OC(C*H*$_3$)$_2$), 1.07 (t, *J* = 8.4 Hz, 2H, C*H*$_2$–Bpin) ppm.

^{13}C NMR: (101 MHz, $CDCl_3$) δ 172.6 (*C*=O), 83.3 (B–O*C*(CH$_3$)$_2$), 79.5 (O*C*(CH$_3$)$_3$), 51.5 (COO*C*H$_3$), 43.3, 33.5, 28.6, 25.0 (B–OC(*C*H$_3$)$_2$) ppm. *The carbon attached to boron was not observed due to quadrupolar*

relaxation.

HRMS (ESI) calculated for $C_{17}H_{32}BNO_6Na$ $[M+Na]^+$ 380.2215, found 380.2230.

IR (neat) ν_{max}: 3387, 2978, 2933, 1740, 1694, 1367, 1166, 1145, 764, 750 cm^{-1}.

methyl (1*R*,3*S*)-2,2-dimethyl-3-(2-(4,4,5,5-tetramethyl-1,3,2-dioxaborolan-2-yl)ethyl)cyclobutane-1-carboxylate (4ca) and methyl 3-((1*S*,3*R*)-2,2-dimethyl-3-(4,4,5,5-tetramethyl-1,3,2-dioxaborolan-2-yl)cyclobutyl)propanoate (4cb)

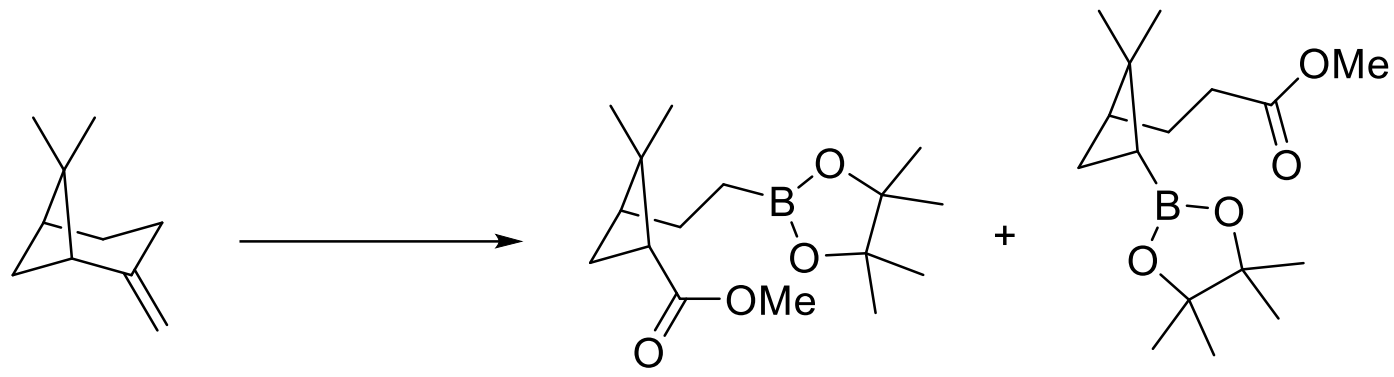

3c **4ca** **4cb**

Prepared following **General Procedure C** with (–)-β-pinene (27.2 mg, 0.200 mmol, 1.00 equiv). The crude was purified by flash column chromatography (SiO_2; 30:1 pentane:EtOAc) to afford the desired boronic ester (36 mg, 0.12 mmol, 61%, inseparable mixture of two isomers, 34% of **4ca** and 27% of **4cb**, 2.4:1 d.r.) as a colorless oil.

TLC: R_f = 0.80 (10:1 pentane:EtOAc, CAM).

NMR Spectroscopy (*see spectra*):

^{1}H NMR: (400 MHz, $CDCl_3$) δ 3.65 (s, 1.5H, 0.5 × COOC$\underline{H_3}$, **4cb** major and minor diastereomer), 3.64 (s, 1.5H, 0.5 × COOC$\underline{H_3}$, **4ca**), 2.62 (m, 0.5H, 0.5 × C$\underline{H_2}$COOCH$_3$, **4ca**), 2.24–2.10 (m, 1H, 0.5 × C$\underline{H_2}$COOCH$_3$, **4ca** + 0.5 × C$\underline{H}$COOCH$_3$, **4cb** major and minor diastereomer), 2.01–1.78 (m, 2H), 1.73–1.26 (m, 3.5H), 1.25 (s, 0.9H, 0.15 × B–OC(C$\underline{H_3}$)$_2$, **4cb** minor diastereomer), 1.25 (s, 0.9H, 0.15 × B–OC(C$\underline{H_3}$)$_2$, **4cb** minor diastereomer), 1.24 (s, 6H, 0.5 × 2 × B–OC(C$\underline{H_3}$)$_2$, **4ca**), 1.23 (s, 2.1H, 0.35 × B–OC(C$\underline{H_3}$)$_2$, **4cb** major diastereomer), 1.22 (s, 2.1H, 0.35 × B–OC(C$\underline{H_3}$)$_2$, **4cb** major diastereomer), 1.17 (s, 1.5H, 0.5 × C$\underline{H_3}$, **4ca**), 1.10 (s, 1.05H, 0.35 × C$\underline{H_3}$, **4cb** major diastereomer), 1.07 (s, 0.90H, 0.15 × 2 × C$\underline{H_3}$, **4cb** minor diastereomer), 1.01 (s, 1.05H, 0.35 × C$\underline{H_3}$, **4cb** major diastereomer), 0.90 (s, 1.5H, 0.5 × C$\underline{H_3}$, **4ca**), 0.74–0.57 (m, 1H, 0.5 × C$\underline{H_2}$–Bpin, **4ca**) ppm.

^{13}C NMR: (101 MHz, $CDCl_3$) δ 174.57 ($\underline{C}$=O, **4cb** minor diastereomer), 174.51 ($\underline{C}$=O, **4cb** major diastereomer), 173.84 ($\underline{C}$=O, **4ca**), 83.13 (B–O$\underline{C}$(CH$_3$)$_2$, **4cb** minor diastereomer), 83.09 (B–O$\underline{C}$(CH$_3$)$_2$, **4ca**), 83.05 (B–O$\underline{C}$(CH$_3$)$_2$, **4cb** major diastereomer), 51.6, 51.2, 45.9, 45.7, 45.0, 44.0, 42.98, 41.0, 39.8, 32.4, 32.2, 31.9, 30.6, 28.9, 26.4, 26.1, 25.20, 25.15, 25.1, 24.98, 24.95, 24.92, 24.6, 24.5, 23.8, 23.4, 20.3, 17.4.ppm. *The carbon attached to boron was not observed due to quadrupolar relaxation.*

HRMS (ESI) calculated for $C_{16}H_{30}BO_4$ $[M+H]^+$ 297.2232, found 297.2222.

IR (neat) ν_{max}: 2977, 2951, 1738, 1371, 1275, 1145, 969, 854, 764, 750 cm^{-1}.

tert-butyl 2-(2-methoxy-2-oxoethyl)-5-((4,4,5,5-tetramethyl-1,3,2-dioxaborolan-2-yl)methyl)pyrrolidine-1-carboxylate (4d)

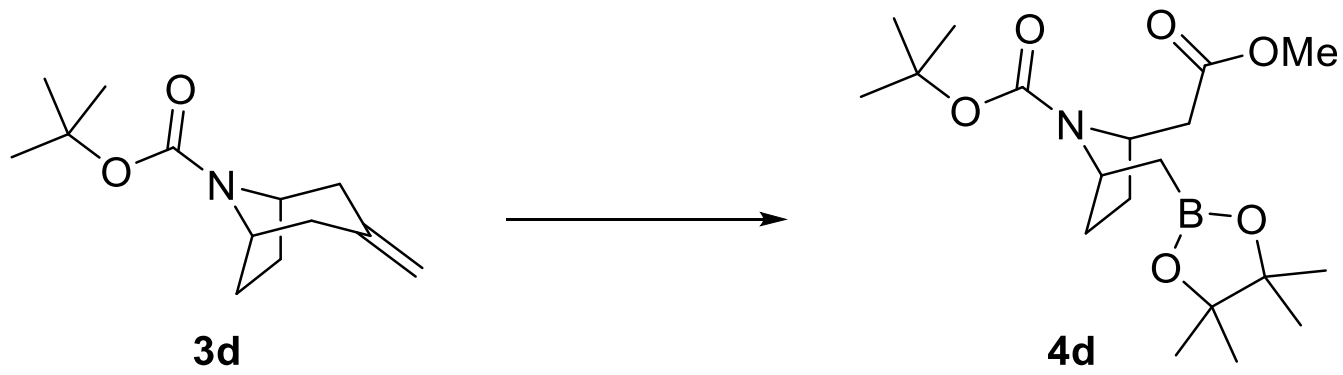

Prepared following **General Procedure B** with *tert*-butyl (1*R*,5*S*)-3-methylene-8-azabicyclo[3.2.1]octane-8-carboxylate **3d** (44.6 mg, 0.200 mmol, 1.00 equiv). The crude was purified by flash column chromatography (boric acid treated SiO_2; 12:1 pentane:EtOAc) to afford the desired boronic ester **4d** (54 mg, 0.14 mmol, 70%) as a colorless oil.

TLC: R_f = 0.58 (3:1 pentane:EtOAc, CAM).

NMR Spectroscopy (*see spectra*):

^{1}H NMR: (400 MHz, $CDCl_3$) δ 4.12 (br, 1H, (Boc)N–C*H*), 3.91 (br, 1H, (Boc)N–C*H*), 3.65 (s, 3H, COOC*H₃*), 2.30–2.38 (m, 1H, (Boc)N–CHC*H₂*), 2.11–1.93 (m, 2H, (Boc)N–CHC*H₂*), 1.74–1.66 (m, 1H, (Boc)N–CHC*H₂*), 1.45 (s, 9H, OC(C*H₃*)₃), 1.30–1.17 (m, 1H, C*H₂*–Bpin) 1.23 (s, 6H, B–OC(C*H₃*)₂), 1.22 (s, 6H, B–OC(C*H₃*)₂), 0.96–0.83 (m, 1H, C*H₂*–Bpin) ppm.

^{13}C NMR: (101 MHz, $CDCl_3$) δ 172.3 (*C*=O), 143.3 (*C*OOMe), 83.1 (B–O*C*(CH_3)₂), 79.4 (O*C*(CH_3)₃), 55.7 ((Boc)N–*C*H), 55.5 ((Boc)N–*C*H), 51.6 (COO*C*H_3), 31.7, 28.6, 25.1 (B–OC(*C*H_3)₂), 24.9 (OC(*C*H_3)₃) ppm. *The carbon attached to boron was not observed due to quadrupolar relaxation.*

HRMS (ESI) calculated for $C_{19}H_{35}BNO_6$ $[M+H]^+$ 384.2552, found 384.2559.

IR (neat) ν_{max}: 3342, 2975, 1732, 1690, 1439, 1393, 1368, 1257, 1167, 1098, 1062, 745 cm^{-1}.

methyl (1*R*,2*S*)-2-isopropyl-2-(2-(4,4,5,5-tetramethyl-1,3,2-dioxaborolan-2-yl)ethyl)cyclopropane-1-carboxylate (4e)

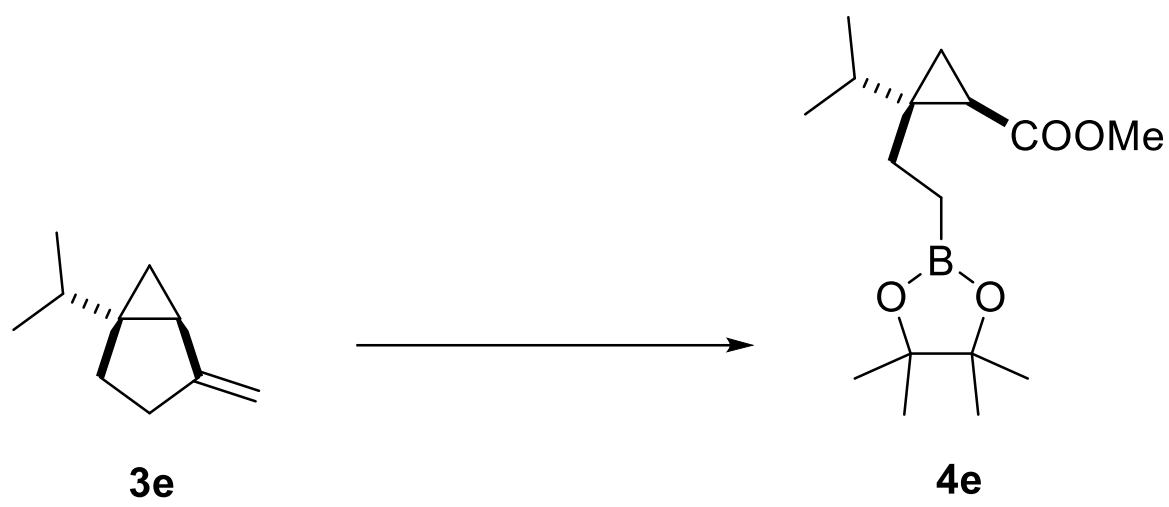

Prepared following **General Procedure C** with (±)-sabinene **3e** (27.2 mg, 0.200 mmol, 1.00 equiv). The crude was purified by flash column chromatography (SiO_2; 15:1 pentane:EtOAc) to afford the desired boronic ester **4e** (49 mg, 0.16 mmol, 82%) as a colorless oil.

TLC: R_f = 0.83 (3:1 pentane:EtOAc, CAM).

NMR Spectroscopy (see spectra):

^{1}H NMR: (400 MHz, $CDCl_3$) δ 3.65 (s, 3H, COOC*H_3*), 1.67–1.52 (m, 4H), 1.23 (s, 12H, 2 × B–OC(C*H_3*)$_2$), 1.03 (dd, *J* = 4.8, 4.4 Hz, 1H, C*H*COOCH_3), 0.90 (d, *J* = 6.8 Hz, 3H, C*H_3*), 0.91–0.81 (m, 2H, C*H_2*–Bpin), 0.85 (d, *J* = 6.8 Hz, 3H, C*H_3*), 0.76–0.67 (m, 1H) ppm.

^{13}C NMR: (101 MHz, $CDCl_3$) δ 173.7 (*C*=O), 83.1 (B–O*C*(CH_3)$_2$), 51.7 (COO*C*H_3), 37.6, 32.3, 25.0 (B–OC(*C*H_3)$_2$), 25.0 (B–OC(*C*H_3)$_2$), 24.4, 22.8, 19.7, 19.4, 19.0 ppm. *The carbon attached to boron was not observed due to quadrupolar relaxation.*

HRMS (ESI) calculated for $C_{16}H_{29}BO_4Na$ $[M+Na]^+$ 319.2057, found 319.2052.

IR (neat) ν_{max}: 2976, 1731, 1437, 1372, 1320, 1167, 1146, 1103, 969, 848 cm^{-1}.

2.5 Comparison of Fe(II)/Fe(III) conditions with selected substrates

Substrate | **Product**

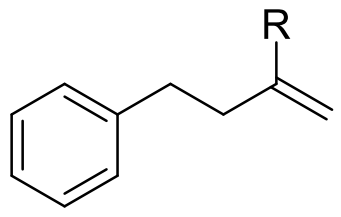

R = H
R = Me

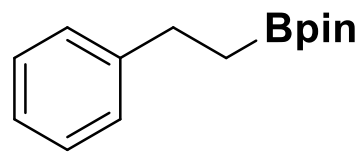

$FeCl_2$, 45%, $Fe(acac)_3$, 43%
$FeCl_2$, 63%, $Fe(acac)_3$, 51%

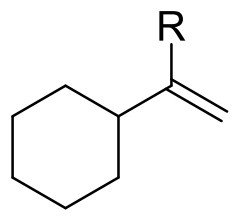

R = H
R = Me

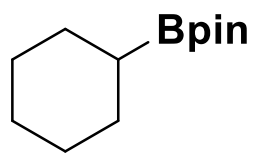

$FeCl_2$, 43%, $Fe(acac)_3$, 40%
$FeCl_2$, 60%, $Fe(acac)_3$, 55%

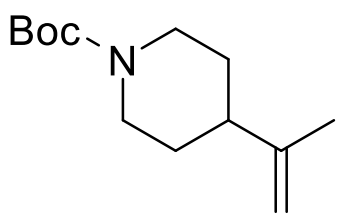

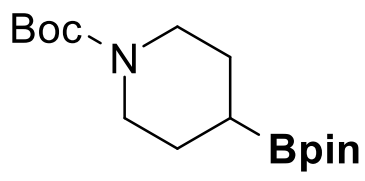

$FeCl_2$, 45%, $Fe(acac)_3$, 60%

Substrate | **Product**

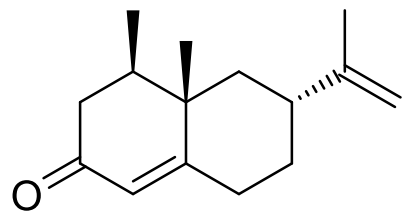

(+)-nootkatone

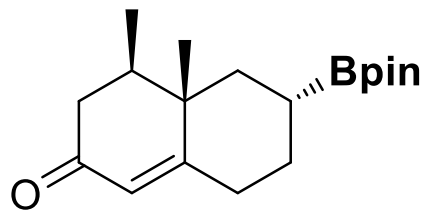

$FeCl_2$, 60%, > 20:1 d.r.
$Fe(acac)_3$, 30%, > 20:1 d.r.

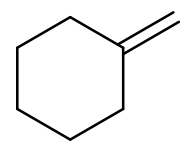

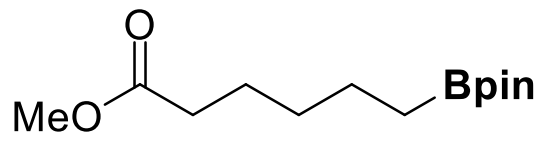

$FeCl_2$, 76%, $Fe(acac)_3$, 67%

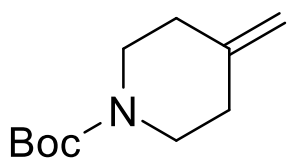

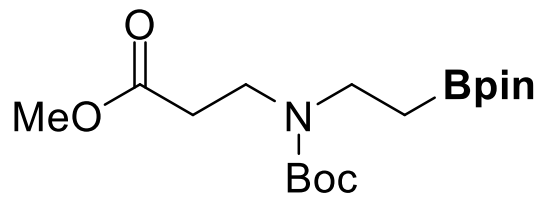

$FeCl_2$, 48%, $Fe(acac)_3$, 63%

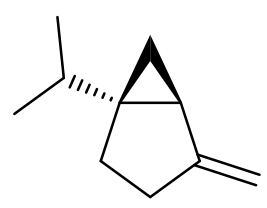

(±)-sabinene

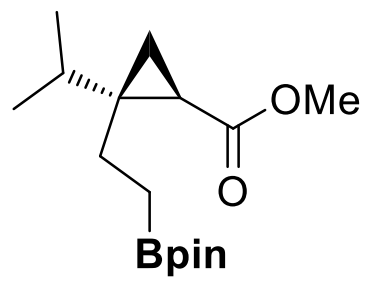

$FeCl_2$, 82%, $Fe(acac)_3$, 76%

Substrate | **Product**

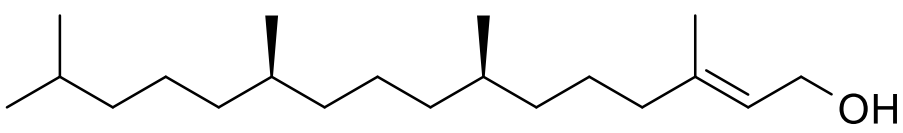

phytol

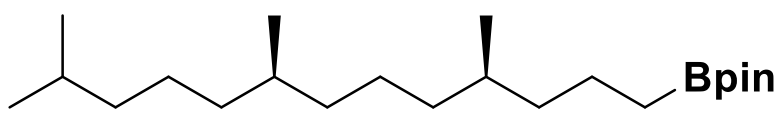

$FeCl_2$, 68%, $Fe(acac)_3$, 56%

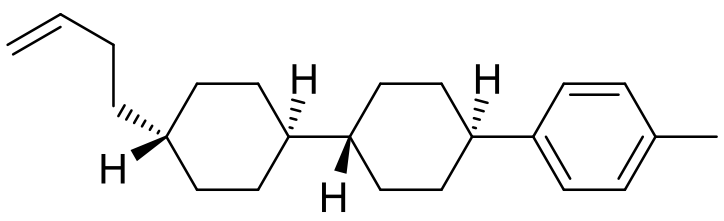

(±)-liquid crystal material

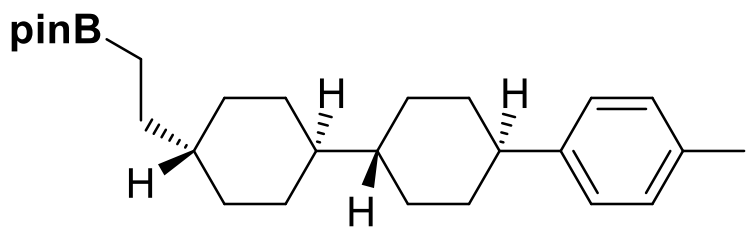

$FeCl_2$, no reaction, $Fe(acac)_3$, 62%

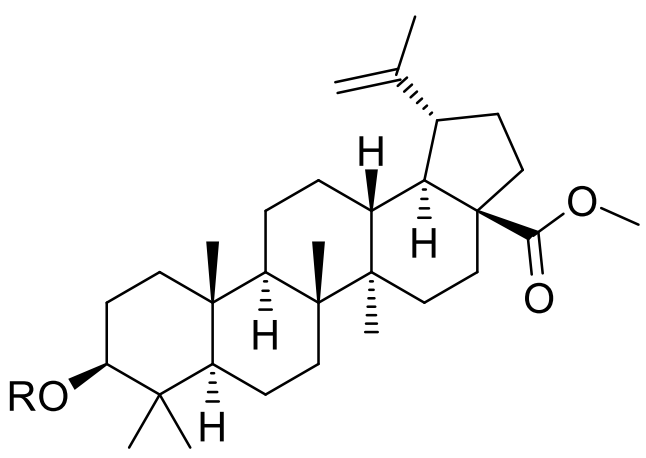

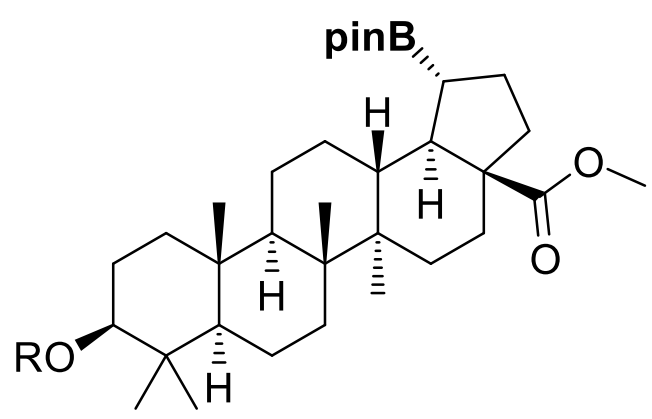

R = H — $FeCl_2$, 46%, > 20:1 d.r.
$Fe(acac)_3$, 20%, > 20:1 d.r.

R = TBS — $FeCl_2$, 53%, > 20:1 d.r.
$Fe(acac)_3$, 27%, > 20:1 d.r.

2.6. Failed Substrates

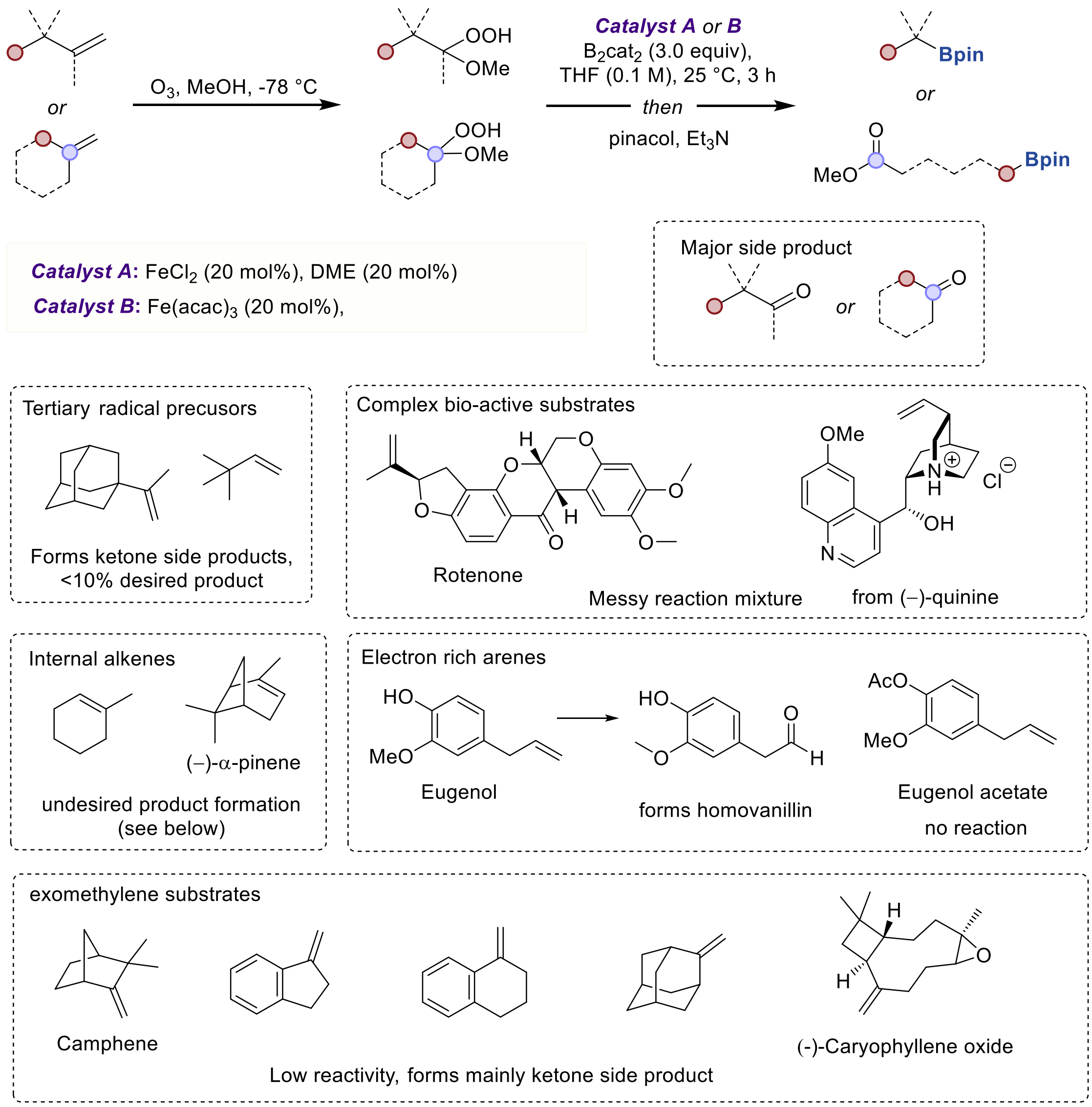

1,5-bis(4,4,5,5-tetramethyl-1,3,2-dioxaborolan-2-yl)pentan-1-ol (4f)

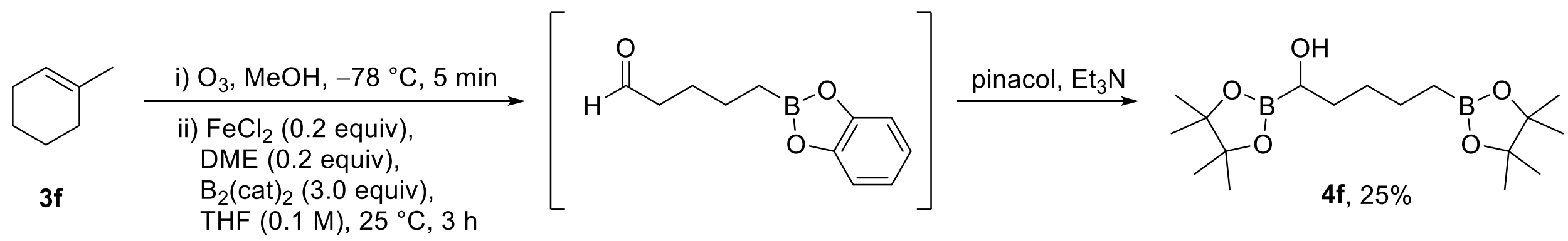

Prepared following **General Procedure B** with 1-methylcyclohex-1-ene **3f** (27.2 mg, 0.200 mmol, 1.00 equiv). Under the standard conditions, the aldehyde ring opening product further reacted with B_2cat_2 in the reaction mixture to generate **4f** upon transesterification with pinacol. The crude was purified by flash column chromatography (boric acid treated SiO_2; 15:1 pentane:EtOAc) to afford **4f** (17 mg, 0.050 mmol, 25%) as a colorless oil.

TLC: R_f = 0.85 (4:1 pentane:EtOAc, CAM).

NMR Spectroscopy:

^{1}H NMR: (400 MHz, $CDCl_3$) δ 5.02 (t, *J* = 5.2 Hz, 1H, C*H*OHBpin), 1.61–1.56 (m, 3H), 1.49–1.36 (m, 4H), 1.24 (s, 12H, 2 × B–OC(C$\underline{H_3}$)$_2$), 1.18 (s, 12H, 2 × B–OC(C$\underline{H_3}$)$_2$), 0.78 (t, *J* = 7.2 Hz, 2H, C$\underline{H_2}$–Bpin) ppm.

^{1}H NMR (400 MHz, $CDCl_3$) of **4f**

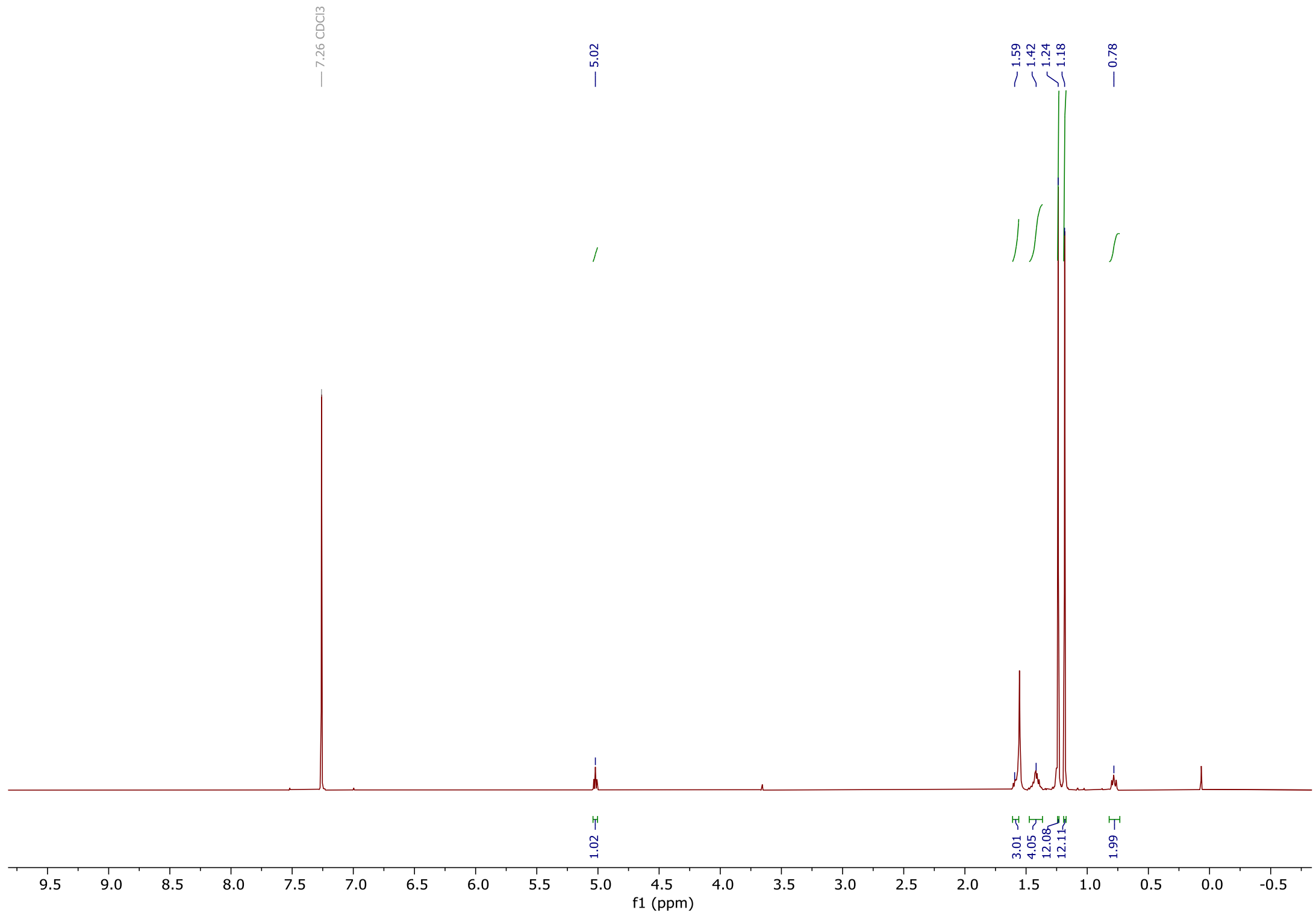

2.7. Benchmarking analysis against previous report of dealkenylative borylation

Luo, *Chem. Eur. J.* **2023**

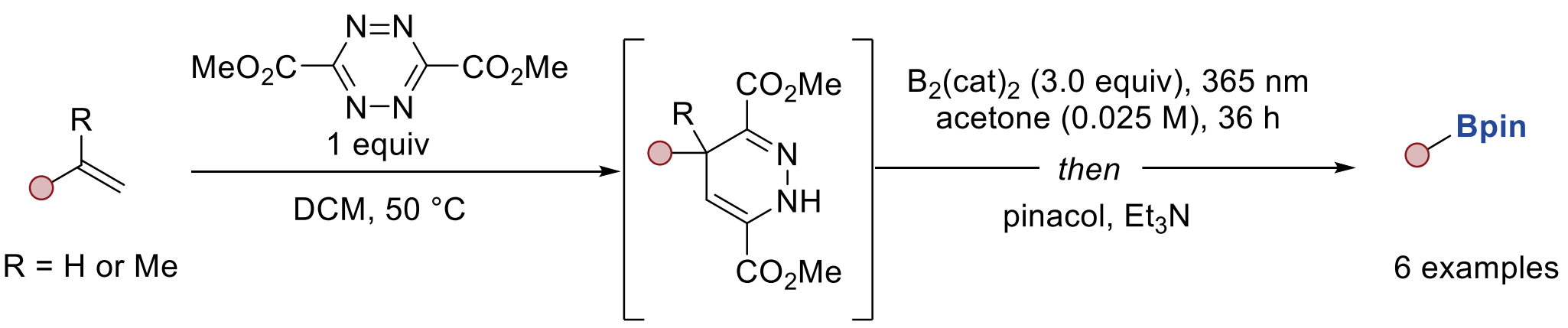

This work

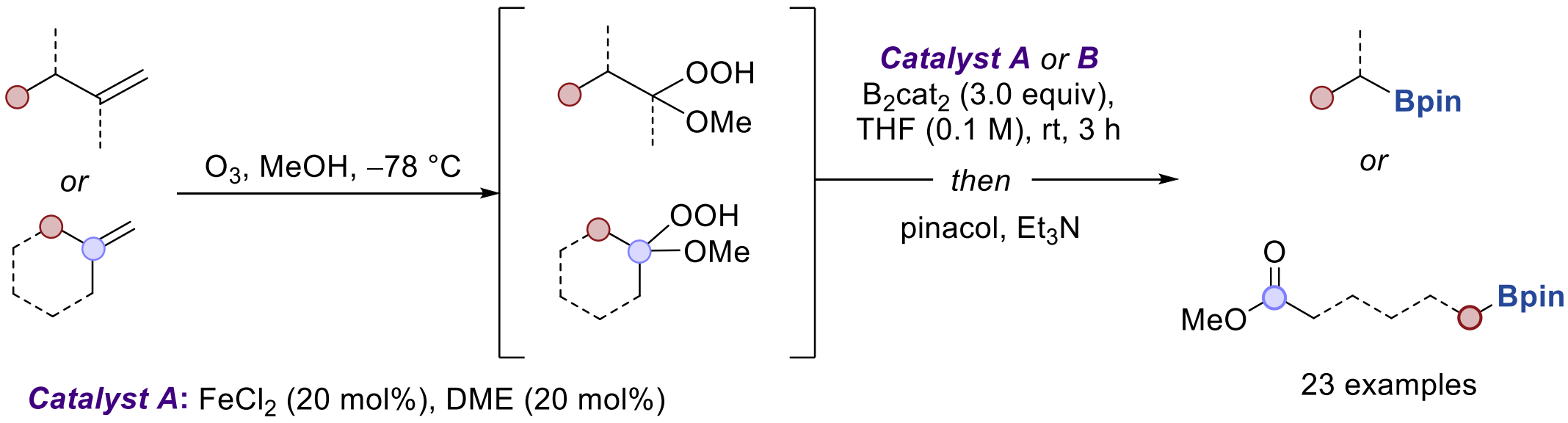

Catalyst A: $FeCl_2$ (20 mol%), DME (20 mol%)
Catalyst B: $Fe(acac)_3$ (20 mol%),

Common examples:

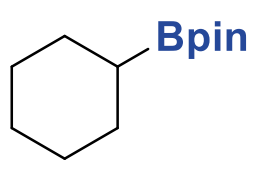

Luo 2023: 43%
this work: 43%

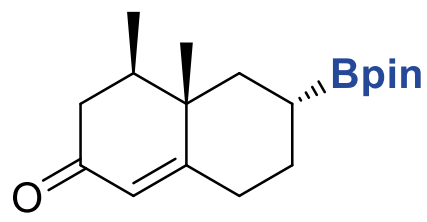

Luo 2023: 48%, d.r. not specifed
this work: 60%, >20:1 d.r.

Comparison	This work	Luo, *Chem. Eur. J.* **2023**
Activation of alkene	Ozonolysis, 5 min	Tetrazine cycloaddition, 5 min to 24 h
Intermediate isolation	not required, solvent switch to THF	not required, solvent switch to acetone
Borylation process	Fe catalyzed, rt, 3h	UV irradiation (365 nm), 36 h
Equiv of B_2cat_2	3.0	3.0
Number of examples	23	6

2.8. Synthesis of Rice Moth Pheromone

(2*R*,6*R*,10*R*)-6,10,14-trimethylpentadecan-2-ol (5)

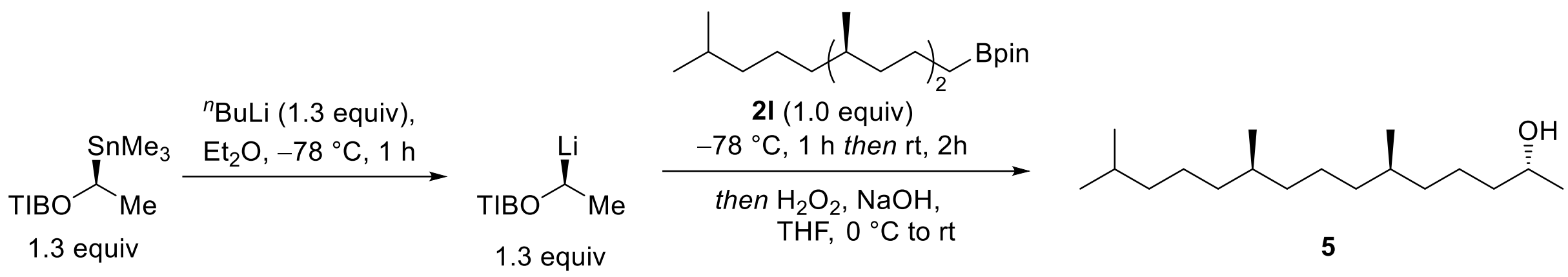

A Schlenk tube equipped with a magnetic stirrer bar was charged with (*S*)-1-(trimethylstannyl)ethyl 2,4,6-triisopropylbenzoate (66.2 mg, 0.156 mmol, 1.30 equiv) under an N_2 atmosphere followed by the addition of anhydrous Et_2O (0.2 M). The mixture was cooled to –78 °C and ^{n}BuLi (94 µL, 0.16 mmol, 1.3 equiv) was added dropwise. The reaction was stirred for 1 h at –78 °C and the boronic ester **2l** (40.6 mg, 0.12 mmol, 1.0 equiv, 0.5 M in anhydrous Et_2O) was then added dropwise. After this addition, the reaction was stirred at –78 °C for 1 h and then stirred at room temperature for 2 h. The reaction mixture was filtered through a short pad of silica and washed with Et_2O (5 mL). The solvent was removed under reduced pressure and the crude boronic ester was redissolved in THF (2 mL) and cooled to 0 °C. This was followed by the addition of aqueous sodium hydroxide (3.0 M, 0.2 mL) and aqueous hydrogen peroxide (30% (w/w), 0.4 mL). The reaction mixture was warmed to room temperature, stirred for 4 h (reaction was monitored by TLC), and quenched with saturated aqueous $Na_2S_2O_3$ (2 mL). The reaction mixture was diluted with ethyl acetate (5 mL) and the aqueous and organic layers were separated. The aqueous layer was extracted with ethyl acetate (3 × 5 mL) and the combined organic layers were dried over anhydrous $MgSO_4$, filtered and concentrated under reduced pressure followed by silica gel flash column chromatography (SiO_2; 10:1 pentane: EtOAc) to afford **5** as a colorless oil (22 mg, 0.083 mmol, 69%).

TLC: R_f = 0.25 (10:1 pentane:EtOAc, $KMnO_4$).

NMR Spectroscopy (*see spectra*):

^{1}H NMR: (400 MHz, $CDCl_3$) δ 3.80 (tq, *J* = 6.0 Hz, 1H, C*H*–OH), 1.52 (sept, *J* = 6.4 Hz, 1H, C*H*), 1.46–1.20 (m, 15H, 6 × CH_2 + CH–O*H* + 3 × C*H*), 1.19 (d, *J* = 6.4 Hz, 3H, CH_3), 1.17–1.00 (m, 6H, 3 × CH_2), 0.88–0.81 (4 × d, 12H, 4 × CH_3) ppm.

^{13}C NMR: (101 MHz, $CDCl_3$) δ 68.40 (*C*H–OH), 39.87, 39.51, 37.52, 37.43, 37.26, 37.16, 32.94, 32.90, 28.12, 24.96, 24.61, 23.67, 23.39, 22.87, 22.77, 19.90, 19.83 ppm.

HRMS (EI) calculated for $C_{18}H_{36}$ $[M–H_2O]^+$ 252.2812, found 252.2806.

Specific Rotation: $[\alpha]_D^{23}$= –7.4 (*c* = 1.08, pentane)

IR (neat) ν_{max}: 3340, 2954, 2925, 2868, 1462, 1377, 1078, 750 cm^{-1}.

All recorded spectroscopic data matched those previously reported in the literature.[18]

2.8.1 Literature synthesis of rice moth pheromone[18]

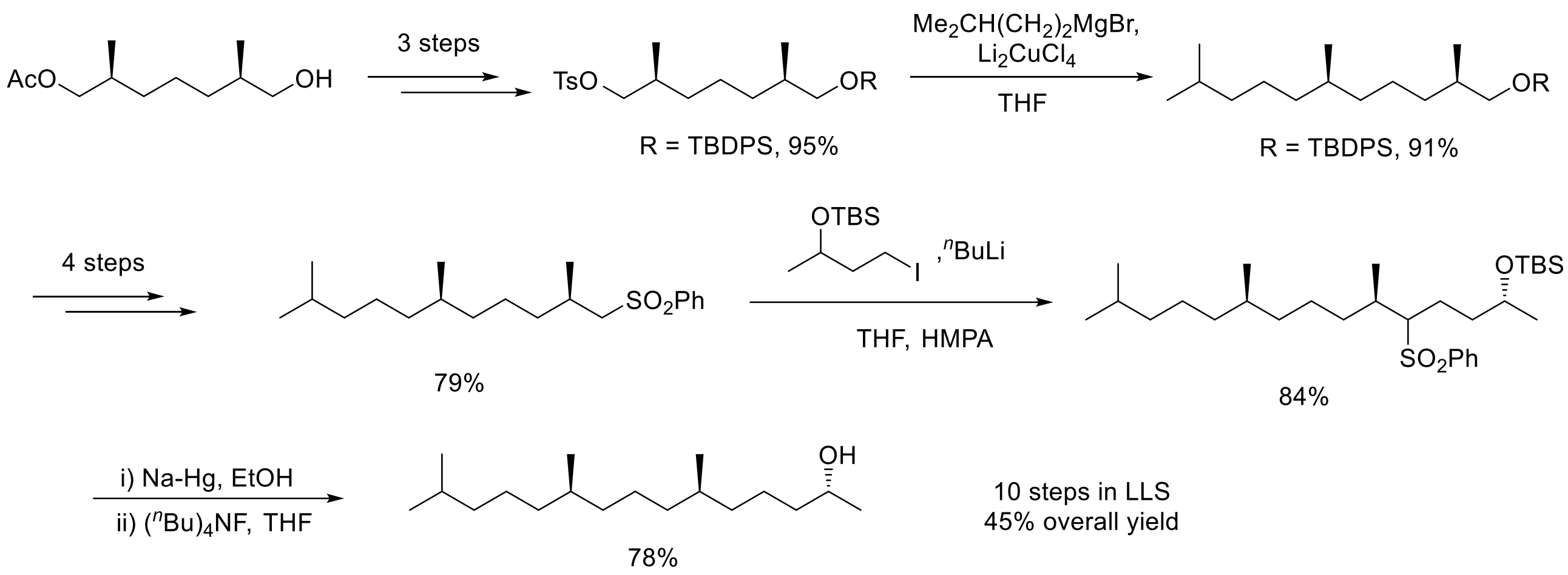

rice moth pheromone

2.9. Synthesis of Solanoquinone

2,3-dimethylnaphthalene-1,4-dione (6)

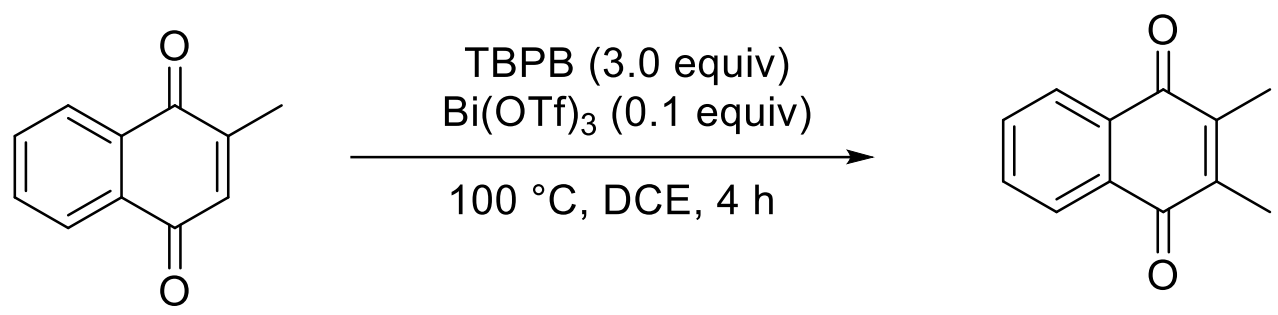

vitamin K_3 **6**

Synthesized following a modified literature procedure.[18] A flame-dried 100 mL RBF was charged with vitamin K_3 (1.72 g, 10.0 mmol, 1.00 equiv), $Bi(OTf)_3$ (0.66 g, 1.0 mmol, 0.10 equiv), and DCE (50 mL). *tert*-Butyl peroxybenzoate (TBPB, 5.6 mL, 30 mmol, 3.0 equiv) was added dropwise and the reaction mixture was stirred at 100 °C for 16 h. After completion, the reaction was quenched with saturated aqueous $Na_2S_2O_3$ (100 mL) and stirred for 3 h at rt. The mixture was extracted with EtOAc (3 × 50 mL). The combined organic layers were washed with aqueous HCl (1 M, 50 mL), to remove the benzoic acid by-product, then with brine (50 mL). The solution was dried over anhydrous $MgSO_4$, filtered and concentrated under reduced pressure followed by silica gel flash column chromatography (SiO_2; 15:1 pentane: EtOAc) to afford **6** (1.5 g, 7.9 mmol, 79%) as a yellow solid.

TLC: R_f = 0.73 (10:1 pentane:EtOAc, UV).

NMR Spectroscopy (*see spectra*):

^{1}H NMR: (400 MHz, $CDCl_3$) δ 8.08 (dd, *J* = 5.6, 3.2 Hz, 2H, 2 × Ar*H*), 7.69 (dd, *J* = 5.6, 3.2 Hz, 2H, 2 × Ar*H*), 2.18 (s, 6H, 2 × C*H*3) ppm.

^{13}C NMR: (101 MHz, $CDCl_3$) δ 185.1 (*C*=O), 143.6 (C=*C*–CO), 133.5, 132.3, 126.4, 13.0 (*C*H3) ppm.

All recorded spectroscopic data matched those previously reported in the literature.[19]

1,4-dimethoxy-2,3-dimethylnaphthalene (7)

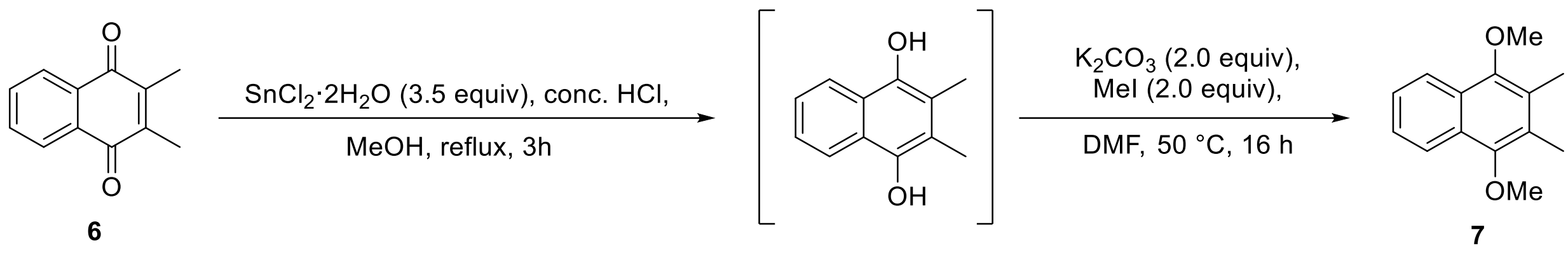

Synthesized following a literature procedure.[20] A flame-dried 25 mL RBF was charged with 2,3-dimethylnaphthalene-1,4-dione **6** (0.37 g, 2.0 mmol, 1.0 equiv) and $SnCl_2 \cdot 2H_2O$ (1.6 g, 7.0 mmol, 3.5 equiv) followed by addition of MeOH (4 mL, 0.5 M). Concentrated hydrochloric acid (12.0 M, 1.0 mL) was added dropwise to the reaction mixture under vigorous stirring. The mixture was heated to reflux for 3 h before cooling down to room temperature. MeOH was removed under reduced pressure until a precipitate was observed, and then further precipitation was achieved by addition of ice-cold water (0 °C, 2 mL). The solid was filtered and dissolved in EtOAc (5 mL), the solution was washed with brine (5 mL) and dried with anhydrous $MgSO_4$. The solvent was removed under reduced pressure to give the desired reduced naphthoquinone, which was used immediately for the next step without further purification. The intermediate was transferred to another flame-dried 25 mL RBF and dissolved in anhydrous DMF (5 mL). Potassium carbonate (0.55 g, 4.0 mmol. 2.0 equiv) was added to the reaction mixture which was stirred for 30 min. Iodomethane (0.25 mL, 4.0 mmol, 2.0 equiv) was added dropwise and the solution was stirred at 50 °C for 16 h. After this time, the reaction was allowed to cool to room temperature and quenched with water (5 mL). The two layers were separated, and the aqueous layer was extracted with EtOAc (2 × 10 mL). The combined organic layers were washed with water (10 mL) and brine (10 mL), dried over anhydrous $MgSO_4$, and concentrated under reduced pressure. The crude product was purified by flash column chromatography (SiO_2; 20:1 pentane:EtOAc) to afford **7** (0.27 g, 1.2 mmol, 62% over 2 steps) as a yellow solid.

TLC: R_f = 0.81 (10:1 pentane:EtOAc, UV).

NMR Spectroscopy (*see spectra*):

^{1}H NMR: (400 MHz, $CDCl_3$) δ 8.08–8.02 (m, 2H, 2 × Ar*H*), 7.49–7.41 (m, 2H, 2 × Ar*H*), 3.87 (s, 6H, 2 × OC*H*$_3$), 2.37 (s, 6H, 2 × C*H*$_3$) ppm.

^{13}C NMR: (101 MHz, $CDCl_3$) δ 149.9 (C=*C*–CO), 127.3, 127.1, 125.4, 122.2, 61.5 (O*C*H$_3$), 13.0 (*C*H$_3$) ppm.

HRMS (EI) calculated for $C_{14}H_{16}O_2$ $[M]^+$ 216.1145, found 216.1141.

IR (neat) ν_{max}: 3070, 2990, 2936, 2840, 1659, 1595, 1457, 1374, 1333, 1271, 1084, 1017, 767, 693 cm^{-1}.

5,8-dimethoxy-6,7-dimethylnaphthalen-2-ol (8)

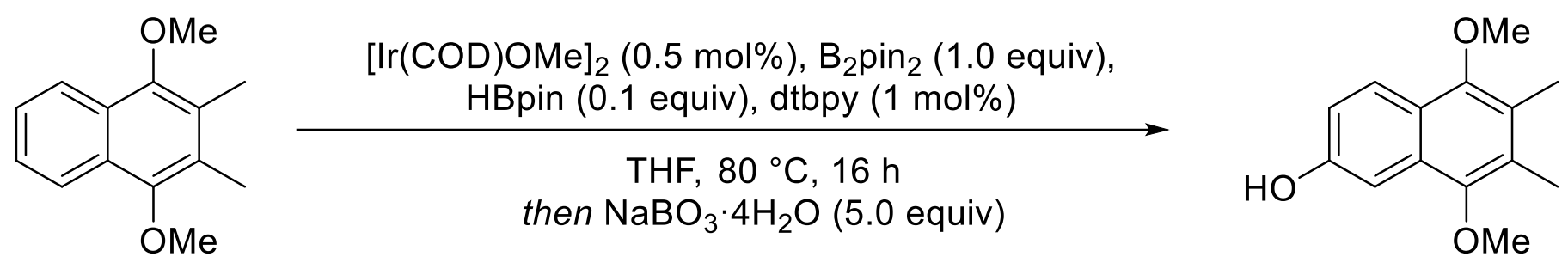

7 8

Synthesized following a literature procedure.[21] A flame-dried 8 mL Biotage® microwave reaction vial equipped with a magnetic stirrer bar was charged with 1,4-dimethoxy-2,3-dimethylnaphthalene **7** (216 mg, 1.00 mmol, 1.00 equiv), B_2pin_2 (254 mg, 1.00 mol, 1.00 equiv), (1,5-cyclooctadiene)(methoxy)iridium(I) dimer (3.3 mg, 5.0 µmol, 0.50 mol%) and 4,4'-di-*tert*-butyl-2,2'-bipyridine (2.7 mg, 0.01 mmol, 1.0 mol%). The flask was back filled with N_2 three times, followed by the addition of anhydrous THF (1 mL). HBpin (15 µL, 0.10 mmol, 0.10 equiv) was added to the solution and the reaction mixture was heated at 80 °C for 16 h, then cooled to 0 °C. The reaction was quenched carefully by the slow addition of MeOH (0.1 mL) until evolution of H_2 ceased. The solvent was removed under reduced pressure and the crude mixture was dissolved in THF (2 mL). To this solution was added $NaBO_3{\cdot}4H_2O$ (0.77 g, 5.0 mmol, 5.0 equiv) and H_2O (1 mL). The suspension was stirred at room temperature for 4 h and quenched by the addition of saturated aqueous $Na_2S_2O_3$ (2 mL). The mixture was stirred for 30 min, then acidified (pH <6) with 2 M HCl upon which the solution turned yellow. This aqueous solution was then extracted with EtOAc (3 × 5 mL), and the combined organic layers were washed with brine (10 mL), dried over anhydrous $MgSO_4$, filtered and concentrated under reduced pressure. The crude product was purified by flash column chromatography (SiO_2; 10:1 pentane:EtOAc) to afford **8** (0.17 g, 0.75 mmol, 75% over 2 steps) as a pale-yellow solid.

TLC: R_f = 0.32 (10:1 pentane:EtOAc, UV).

NMR Spectroscopy (*see spectra*):

^{1}H NMR: (400 MHz, $CDCl_3$) δ 7.96 (d, *J* = 9.2 Hz, 1H, Ar*H*), 7.36 (d, *J* = 2.4 Hz, 1H, Ar*H*), 7.06 (dd, *J* = 9.2, 2.4 Hz, 1H, Ar*H*), 5.07 (br, 1H, Ar–O*H*) 3.85 (s, 3H, OC*H*$_3$), 3.83 (s, 3H, OC*H*$_3$), 2.35 (s, 3H, C*H*$_3$), 2.32 (s, 3H, C*H*$_3$) ppm.

^{13}C NMR: (101 MHz, $CDCl_3$) δ 153.7 (Ar*C*–OH), 150.1 (Ar*C*–OMe), 148.6 (Ar*C*–OMe), 128.5, 128.1, 124.5, 124.4, 122.8, 117.2, 104.3, 61.6 (O*C*H$_3$), 61.2 (O*C*H$_3$), 13.2 (*C*H$_3$) 12.8 (*C*H$_3$) ppm.

HRMS (EI) calculated for $C_{14}H_{16}O_3$ $[M]^+$ 232.1094, found 232.1090.

IR (neat) ν_{max}: 3299, 2937, 2841, 1658, 1599, 1582, 1501, 1457, 1376, 1314, 1085, 1013, 729 cm^{-1}.

5,8-dimethoxy-6,7-dimethylnaphthalen-2-yl trifluoromethanesulfonate (9)

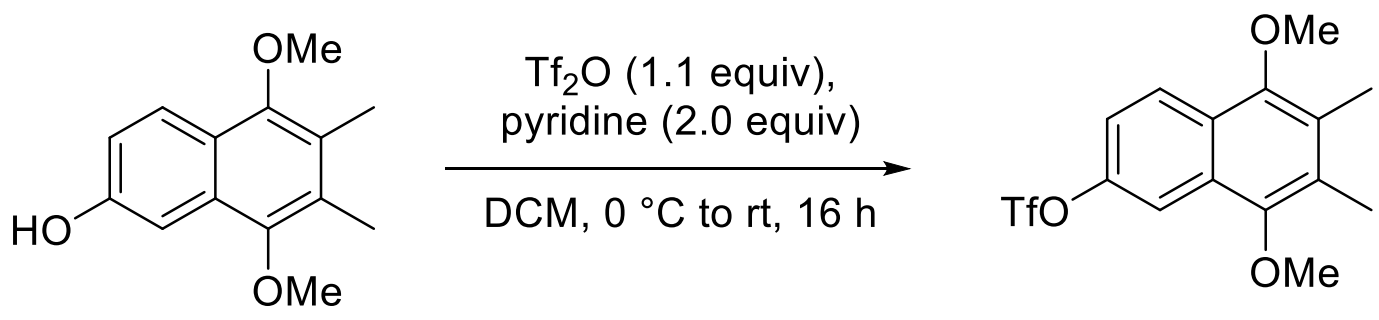

8 9

A flame-dried 8 mL Biotage® microwave reaction vial equipped with a magnetic stirrer bar was charged with 5,8-dimethoxy-6,7-dimethylnaphthalen-2-ol **8** (140 mg, 0.600 mmol, 1.00 equiv) followed by anhydrous DCM (3 mL, 0.2 M) and anhydrous pyridine (97 µL, 1.2 mmol, 2.0 equiv). The reaction was cooled to 0 °C and Tf_2O (1.0 M in DCM, 0.66 mL, 0.66 mmol, 1.1 equiv) was added dropwise under vigorous stirring. The reaction was allowed to warm to room temperature and stirred for 16 h. After completion, the reaction was quenched with water (2 mL). The layers were separated and the aqueous layer was extracted with DCM (3 × 5 mL). The combined organic layers were washed with 1 M HCl (5 mL), brine (5 mL), dried over anhydrous $MgSO_4$, filtered and concentrated under reduced pressure. The crude product was purified by flash column chromatography (SiO_2; 15:1 pentane:EtOAc) to afford **9** (0.18 g, 0.50 mmol, 83%) as an orange solid.

TLC: R_f = 0.55 (10:1 pentane:EtOAc, UV).

NMR Spectroscopy (*see spectra*):

^{1}H NMR: (400 MHz, $CDCl_3$) δ 8.12 (d, *J* = 9.2 Hz, 1H, Ar*H*), 7.92 (d, *J* = 2.4 Hz, 1H, Ar*H*), 7.32 (dd, *J* = 9.2, 2.4 Hz, 1H, Ar*H*), 3.86 (s, 3H, OC*H3*), 3.86 (s, 3H, OC*H3*), 2.38 (s, 3H, C*H3*), 2.38 (s, 3H, C*H3*) ppm.

^{13}C NMR: (101 MHz, $CDCl_3$) δ 149.8 (Ar*C*–OMe), 149.7 (Ar*C*–OMe), 147.4 (Ar*C*–OTf), 129.6, 128.9, 127.4, 126.3, 125.2, 118.90 (*C*F_3, q, *J* = 321 Hz), 118.86, 114.1, 61.6 (O*C*H_3), 61.5 (O*C*H_3), 13.1(*C*H_3), 13.0 (*C*H_3) ppm.

^{19}F NMR (377 MHz, $CDCl_3$) δ –72.7 ppm.

HRMS (EI) calculated for $C_{15}H_{15}O_5SF_3$ $[M]^+$ 364.0587, found 364.0580.

IR (neat) ν_{max}: 2998, 2943, 2842, 1738, 1666, 1599, 1454, 1372, 1366, 1210, 1139, 1087, 916, 865, 609 cm^{-1}.

2,3-dimethyl-6-((4*R*,8*R*)-4,8,12-trimethyltridecyl)naphthalene-1,4-dione (10)

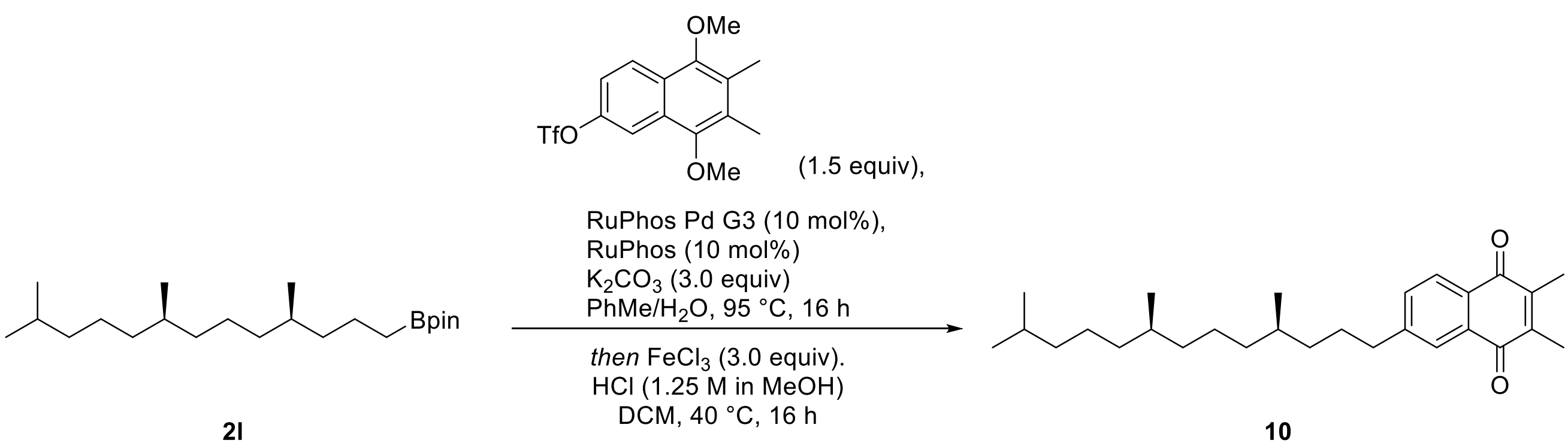

2l 10

A flame-dried 8 mL Biotage® microwave reaction vial equipped with a magnetic stirrer bar was charged with **2l** (35.2 mg, 0.10 mmol, 1.00 equiv), 5,8-dimethoxy-6,7-dimethylnaphthalen-2-yl trifluoromethanesulfonate **9** (55 mg, 0.15 mmol, 1.5 equiv), RuPhos Pd G3 (8.4 mg, 10 μmol, 0.10 equiv), RuPhos (4.7 mg, 10 μmol, 0.1 equiv) and K_2CO_3 (41 mg, 0.3 mmol, 3.0 equiv). The flask was back filled with N_2 three times, followed by the addition of toluene (1 mL, 0.1 M) and water (0.1 mL). The reaction was heated at 95 °C for 16 h before cooling to room temperature. The reaction mixture was passed through a short pad of silica, washing with pentane/EtOAc (10 mL, 9:1), and the solvent was removed under reduced pressure. The crude was dissolved in DCM (2 mL, 0.05 M) and $FeCl_3$ (48.8 mg ,0.30 mmol, 3.00 equiv) was added to the mixture followed by dropwise addition of hydrochloric acid (1.25 M in MeOH, 0.24 mL, 0.3 mmol, 3.0 equiv). The reaction was heated to 40 °C and allowed to stir for 16 h. The reaction was monitored by TLC and, upon completion, the reaction was diluted with Et_2O (5 mL), filtered through a short pad of silica, washed with Et_2O (10 mL) and concentrated under reduced pressure. The crude was purified by flash column chromatography (SiO_2; 30:1 pentane:EtOAc) to afford **10** (29 mg, 0.071 mmol, 71% over 2 steps) as a colorless oil.

TLC: R_f = 0.47 (20:1 pentane:EtOAc, CAM).

NMR Spectroscopy (*see spectra*):

^{1}H NMR: (400 MHz, $CDCl_3$) δ 7.98 (d, *J* = 8.0 Hz, 1H, Ar*H*), 7.87 (d, *J* = 1.6 Hz, 1H, Ar*H*), 7.48 (dd, *J* = 8.0, 1.6 Hz, 1H, Ar*H*), 2.70 (m, 2H, Ar–C*H*$_2$), 2.16 (s, 6H, 2 × C*H*$_3$), 1.74–1.57 (m, 2H, 2 × CH_3C*H*), 1.51 (tq, *J* = 6.8, 6.8 Hz, 1H, $(CH_3)_2$C*H*), 1.45–0.98 (m, 16H, 8 × C*H*$_2$), 0.88–0.80 (4 × d, *J* = 6.4 Hz, 12H, 4 × C*H*$_3$) ppm.

^{13}C NMR: (101 MHz, $CDCl_3$) δ 185.45 (*C*=O), 185.00 (*C*=O), 149.44 (Ar*C*–CH_2), 143.52 (C=*C*–CO), 143.34 (C=*C*–CO), 133.57, 132.27, 130.26, 126.63, 126.14, 39.50, 37.51, 37.41, 36.80, 36.71, 36.59, 32.91, 32.77, 28.68, 28.12, 24.94, 24.58, 22.86, 22.77, 19.83, 19.73, 13.03, 13.01 ppm.

HRMS (ESI) calculated for $C_{28}H_{43}O_2$ $[M+H]^+$ 411.3258, found 411.3251.

IR (neat) ν_{max}: 2954, 2927, 2867, 1781, 1728, 1698, 1662, 1601, 1462, 1377, 1275, 1262, 764, 750 cm^{-1}.

All recorded spectroscopic data matched those previously reported in the literature.[22]

2.9.1 Literature synthesis of solanoquinone[22]

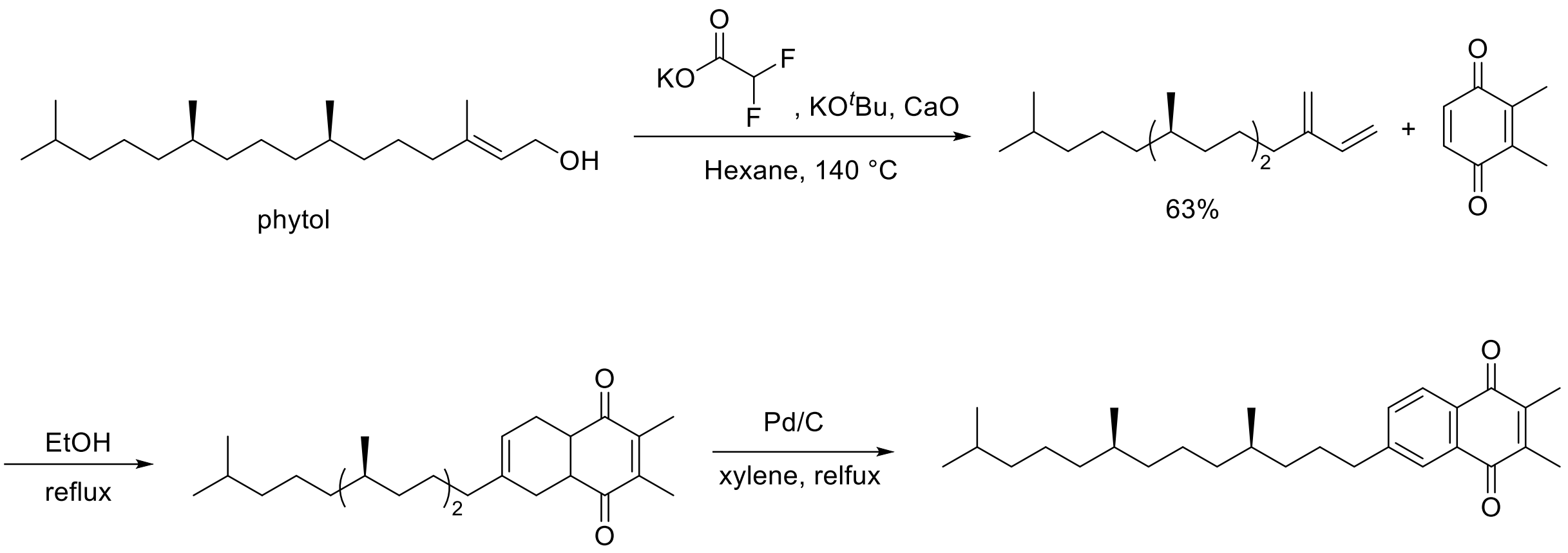

solanoquinone, 35% over 2 steps
22% overall yield

2.10. Synthetic diversifications on betulinic acid derivatives

***tert*-butyl (1*S*,3a*S*,5a*R*,5b*R*,7a*R*,9*S*,11a*R*,11b*R*,13a*R*,13b*R*)-9-((tert-butyldimethylsilyl)oxy)-5a,5b,8,8,11a-pentamethyl-1-vinylicosahydro-3a*H*-cyclopenta[*a*]chrysene-3a-carboxylate (11)**

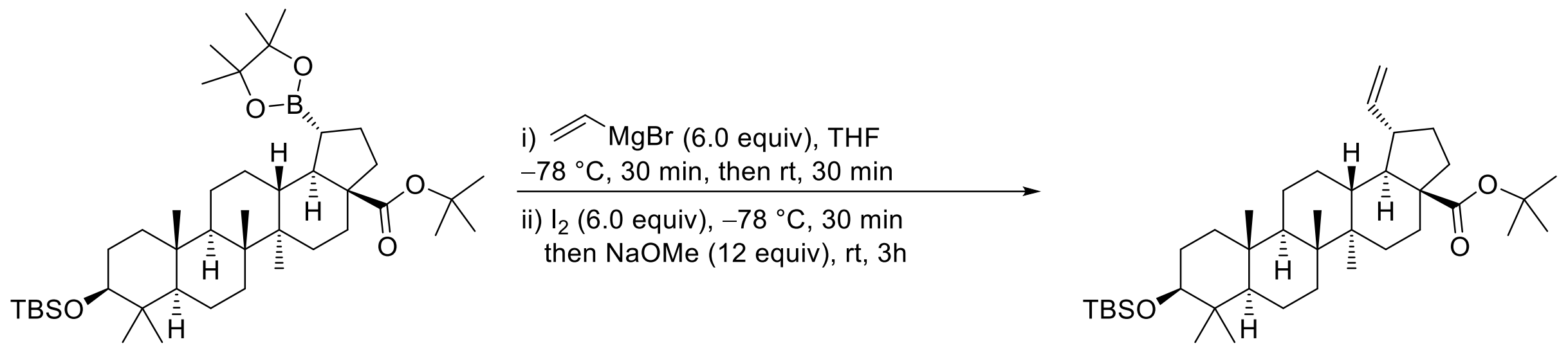

2r **11**

To a solution of boronic ester **2r** (71.3 mg, 0.10 mmol, 1.00 equiv) in anhydrous THF (1.0 mL) at –78 °C was added vinylmagnesium bromide (0.6 mL, 1.0 M in THF, 0.6 mmol, 6.0 equiv) and the resulting solution was stirred at –78 °C for 30 min. The reaction mixture was then warmed up to room temperature stirred for further 30 min and cooled down to –78 °C. A solution of iodine (0.15 g, 0.60 mmol, 6.00 equiv) in MeOH (2 mL) was added dropwise to the reaction mixture. After 30 min, a solution of NaOMe (2.4 mL, 0.5 M in MeOH, 1.2 mmol, 12 equiv) was added. The reaction mixture was then allowed to warm to room temperature and stirred for an additional 3 h. The reaction was monitored by TLC and, upon completion, was quenched with saturated aqueous $Na_2S_2O_3$ (2 mL). The phases were separated, and the aqueous layer was extracted with Et_2O (2 × 10 mL). The combined organic layers were dried over anhydrous $MgSO_4$, filtered and concentrated under reduced pressure. The crude product was purified by flash column chromatography (SiO_2; 100:1 pentane:EtOAc) to afford **11** (54 mg, 0.088 mmol, 88%, >20:1 d.r.) as a colorless oil.

TLC: R_f = 0.95 (20:1 pentane:EtOAc, CAM).

NMR Spectroscopy (*see spectra*):

^{1}H NMR: (400 MHz, $CDCl_3$) δ 5.69 (dt, *J* = 17.2, 10.2 Hz, 1H, CHC*H*=CH_2), 4.92 (d, *J* = 17.2, 1.6 Hz, 1H, CHCH=CH_2), 4.78 (dd, *J* = 10.2, 1.6 Hz, 1H, CHCH=CH_2), 3.15 (dd, *J* = 11.2, 4.4 Hz, 1H, C*H*–OTBS), 3.00–2.86 (m, 1H), 2.31–2.15 (m, 2H), 2.00–1.88 (m, 1H), 2.00–1.88 (m, 1H), 1.75–1.49 (m, 5H), 1.46 (s, 9H, COOC(CH_3)$_3$), 1.44–1.22 (m, 13H), 1.15–1.07 (m, 1H), 0.92 (s, 6H, 2 × CH_3), 0.88 (s, 9H, OSi(CH_3)$_2$C(CH_3)$_3$), 0.87 (s, 3H, CH_3), 0.81 (s, 3H, CH_3), 0.71 (s, 3H, CH_3), 0.67–0.62 (m, 1H), 0.02 (s, 6H, OSi(CH_3)$_2$C(CH_3)$_3$) ppm.

^{13}C NMR: (101 MHz, $CDCl_3$) δ 175.8, 147.1, 112.2, 79.7, 79.6, 57.0, 55.6, 53.6, 50.6, 44.6, 42.4, 40.9, 39.6, 38.9, 38.3, 37.2, 36.9, 34.5, 32.5, 31.4, 29.7, 28.5, 28.2, 28.0, 27.1, 26.1, 21.1, 18.6, 18.3, 16.3, 16.1, 16.0, 14.7, –3.6, –4.8.ppm.

HRMS (MALDI) calculated for $C_{39}H_{68}O_3SiNa$ $[M+Na]^+$ 635.4830, found 635.4839.

IR (neat) ν_{max}: 2945, 2867, 1719, 1638, 1462, 1366, 1257, 1152, 1133, 835, 765, 750 cm^{-1}.

***tert*-butyl (1*R*,3a*S*,5a*R*,5b*R*,7a*R*,9*S*,11a*R*,11b*R*,13a*R*,13b*R*)-9-((*tert*-butyldimethylsilyl)oxy)-5a,5b,8,8,11a-pentamethyl-1-(1-phenylvinyl)icosahydro-3a*H*-cyclopenta[*a*]chrysene-3a-carboxylate (12)**

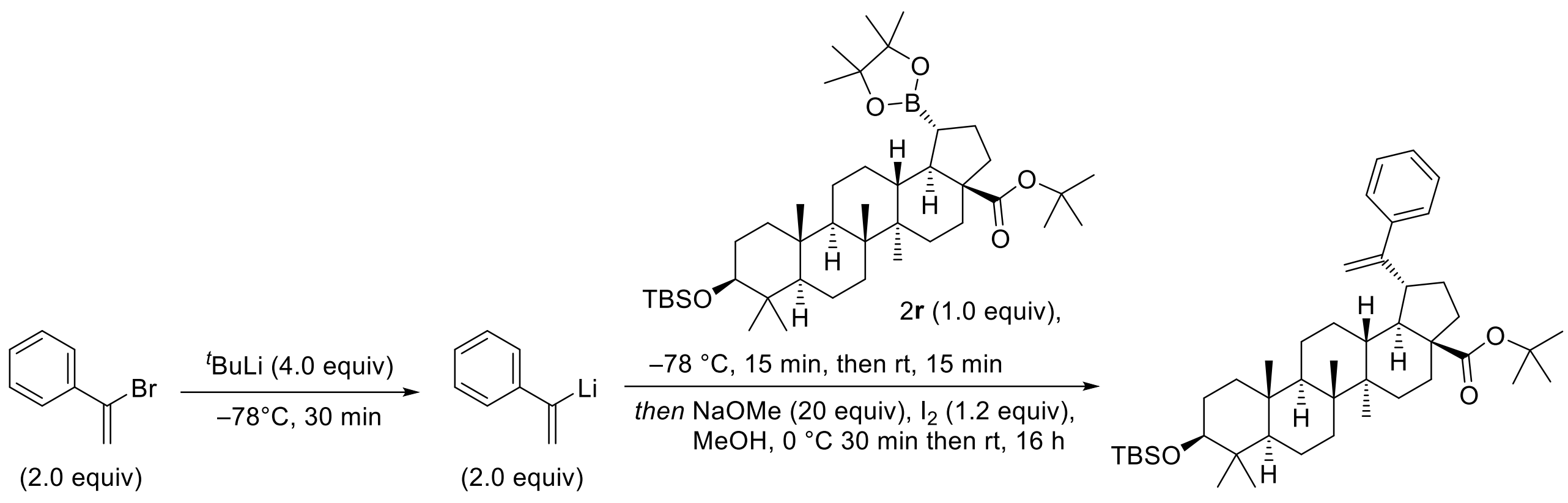

12

A stirred solution of α-bromostyrene (36.6 mg, 0.200 mmol, 2.00 equiv) in anhydrous THF (1.0 mL) was cooled to –78 °C and ^{t}BuLi (1.7 M in pentane, 0.24 mL, 0.40 mmol, 4.0 equiv) was added dropwise. The resulting solution was stirred at –78 °C for 30 min and then a solution of boronic ester **2r** (71.3 mg, 0.100 mmol, 1.00 equiv) in THF (1.0 mL) was added dropwise. The resulting solution was stirred at –78 °C for 15 min and then warmed to 0 °C and stirred for a further 15 min. After this time, the solution was cooled to –78 °C and a suspension of sodium methoxide (1 mL, 2 M in MeOH, 2.0 mmol, 20 equiv) was added in a single portion followed by the dropwise addition of a solution of iodine (31 mg, 0.5 M in MeOH, 0.24 mL, 0.12 mmol, 1.2 equiv). The resulting mixture was warmed to 0 °C and stirred for 30 min before being warmed to room temperature and stirred for 16 h. Saturated aqueous $Na_2S_2O_3$ (5 mL) was added followed by water (5 mL) and dichloromethane (10 mL). The organic layer was separated, and the aqueous layer was extracted with dichloromethane (2 × 10 mL). The combined organic layers were dried over $MgSO_4$, filtered and concentrated under reduced pressure. The crude product was purified by flash column chromatography (SiO_2; 8:1 pentane:DCM) to afford **12** (46 mg, 0.067 mmol, 67%, >20:1 d.r.) as a pale-yellow oil.

TLC: R_f = 0.95 (20:1 pentane:EtOAc, CAM).

NMR Spectroscopy (<u>*see spectra*</u>):

^{1}H NMR: (400 MHz, $CDCl_3$) δ 7.50–7.35 (m, 2H), 7.33–7.28 (m, 2H), 7.25–7.21 (m, 1H), 5.17 (s, 1H, PhC=C*H*$_2$), 5.07 (s, 1H, PhC=C*H*$_2$), 3.31 (dt, *J* = 11.2, 4.0 Hz, 1H, C*H*C=CH$_2$), 3.14 (dd, *J* = 11.2, 4.0 Hz, 1H, C*H*–OTBS), 2.28–2.14 (m, 3H), 1.90–1.77 (m, 2H), 1.63–1.56 (m, 2H), 1.53–1.49 (m, 3H), 1.47 (s, 9H, COOC(C*H*$_3$)$_3$), 1.45–1.40 (m, 3H), 1.38–1.28 (m, 6H), 1.23–1.08 (m, 4H), 0.95 (s, 3H, C*H*$_3$), 0.92 (s, 3H, C*H*$_3$), 0.88 (s, 9H, OSi(CH$_3$)$_2$C(C*H*$_3$)$_3$), 0.87 (s, 3H, C*H*$_3$), 0.78 (s, 3H, C*H*$_3$), 0.71 (s, 3H, C*H*$_3$), 0.66–0.62 (m, 1H), 0.02 (s, 6H, OSi(C*H*$_3$)$_2$C(CH$_3$)$_3$) ppm.

^{13}C NMR: (101 MHz, $CDCl_3$) δ 175.7, 156.2, 143.7, 128.3, 127.1, 126.7, 109.9, 79.8, 79.6, 57.1, 55.6, 52.0, 50.8, 43.7, 42.5, 41.0, 39.6, 38.9, 38.6, 37.2, 37.1, 34.6, 34.2, 32.6, 29.9, 28.6, 28.3, 28.0, 27.7, 26.1, 21.4, 18.6, 18.3, 16.3, 16.1, 16.0, 14.9, –3.6, –4.8.ppm.

HRMS (MALDI) calculated for $C_{45}H_{72}O_3SiNa$ $[M+Na]^+$ 711.5143, found 711.5151.

IR (neat) ν_{max}: 2929, 2855, 1718, 1625, 1462, 1275, 1260, 1147, 886, 764, 750 cm^{-1}.

***tert*-butyl (1*R*,3a*S*,5a*R*,5b*R*,7a*R*,9*S*,11a*R*,11b*R*,13a*R*,13b*S*)-9-((*tert*-butyldimethylsilyl)oxy)-1-(3-fluoropyridin-4-yl)-5a,5b,8,8,11a-pentamethylicosahydro-3a*H*-cyclopenta[*a*]chrysene-3a-carboxylate (13)**

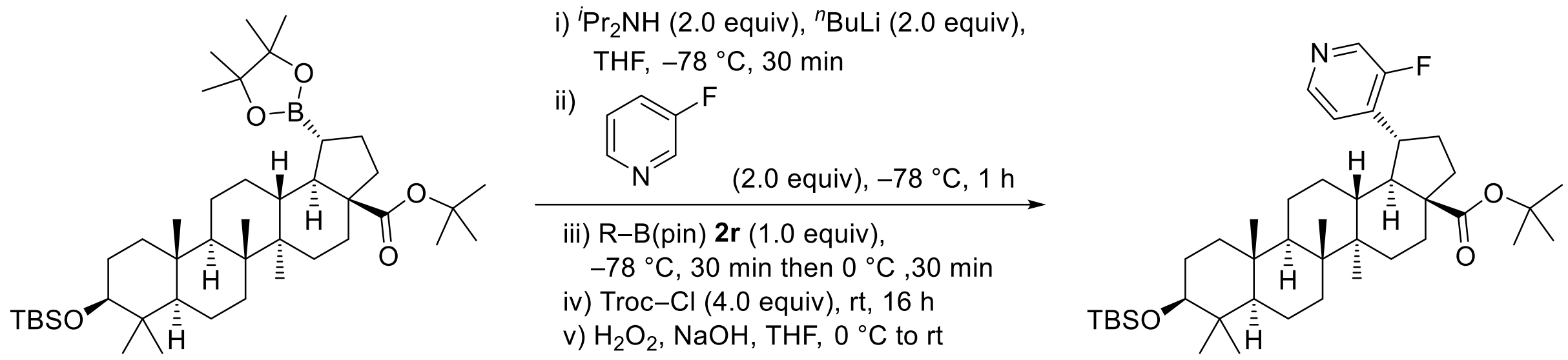

2r **13**

A solution of *N,N*–diisopropylamine (20 mg, 0.20 mmol, 2.0 equiv) in anhydrous THF (1.0 mL) was cooled to –78 °C and treated with ^{n}BuLi (2.4 M in hexanes, 83 µL, 0.2 mmol, 2.0 equiv) before being stirred at –78 °C for 30 min. A solution of 3-fluoropyridine (19.4 mg, 0.200 mmol, 2.00 equiv) in anhydrous THF (0.5 mL) was added dropwise. The mixture was stirred at –78 °C for 30 min and a solution of boronic ester **2r** (71.3 mg, 0.100 mmol, 1.00 equiv) in THF (0.5 mL) was added dropwise. The mixture was stirred at –78 °C for 30 min and then warmed to 0 °C and stirred for another 30 min. The reaction was cooled back to –78 °C and 2,2,2-trichloroethoxycarbonyl chloride (84.7 mg, 0.400 mmol, 4.00 equiv) was added dropwise. The mixture was allowed to warm to room temperature and stirred for 16 h. The crude mixture was diluted with Et_2O (5 mL), and H_2O (5 mL) was added to quench the reaction. The layers were separated and the aqueous phase was neutralized with saturated aqueous $NaHCO_3$ solution. The aqueous layer was extracted with Et_2O (2 × 10 mL) and the combined organic layers were dried over anhydrous $MgSO_4$, filtered and concentrated under reduced pressure. The residue was filtered through a short pad of silica, eluting with 20 mL of pentane:EtOAc (9:1). The solution was evaporated under reduced pressure, taken up in THF (1 mL) and cooled to 0 °C. NaOH (0.2 mL, 2 M) and H_2O_2 (0.2 mL, 30% w/w) were added, and the mixture was stirred for 12 h at room temperature. Et_2O (2 mL) was then added, the layers were separated and the aqueous phase was acidified with aqueous HCl solution (2 mL, 1 M) and extracted with Et_2O (2 × 10 mL). The aqueous phase was neutralized with saturated aqueous $NaHCO_3$ solution and extracted with Et_2O (10 mL). The combined organic layers were dried over anhydrous $MgSO_4$, filtered and concentrated under reduced pressure. The crude product was purified by flash column chromatography (SiO_2; 10:1 pentane:EtOAc) to afford **13** (42 mg, 0.062 mmol, 62%, >20:1 d.r.) as a white solid.

TLC: R_f = 0.24 (10:1 pentane:EtOAc, CAM).

NMR Spectroscopy (*see spectra*):

^{1}H NMR: (400 MHz, $CDCl_3$) δ 8.34 (br, 2H), 7.28 (br, 1H), 3.11 (dd, *J* = 10.8, 4.4 Hz, 1H, C*H*–OTBS), 2.34–2.24 (m, 3H), 2.02–1.95 (m, 1H), 1.59–1.51 (m, 3H), 1.49 (s, 9H, COOC(CH_3)$_3$), 1.45–1.13 (m, 14H), 0.92 (s, 3H, CH_3), 0.89 (s, 3H, CH_3), 0.86 (s, 9H, OSi(CH_3)$_2$C(CH_3)$_3$), 0.86 (s, 3H, CH_3), 0.78 (s, 3H, CH_3), 0.69 (s, 3H, CH_3), 0.63–0.59 (m, 1H), 0.00 (s, 6H, OSi(CH_3)$_2$C(CH_3)$_3$) ppm.

^{13}C NMR (major rotamer): (126 MHz, $CDCl_3$) δ 175.2, 157.9 (d, J = 292.3 Hz), 145.9, 145.3, 137.6, 123.0, 80.3, 79.5, 57.3, 55.5, 50.4, 42.5, 40.8, 39.5, 38.8, 38.2, 37.2, 34.5, 34.3, 32.4, 29.9, 29.6, 28.5, 28.2, 27.9, 26.08, 26.06, 22.5, 20.9, 18.6, 18.3, 16.2, 16.0, 14.6, 14.2, –3.6, –4.8.ppm.

^{19}F NMR: (377 MHz, $CDCl_3$) δ –128.4 (major rotamer), –132.9 (minor rotamer) ppm.

HRMS (MALDI) calculated for $C_{42}H_{69}NO_3FSi$ $[M+H]^+$ 682.5025, found 682.5029.

IR (neat) ν_{max}: 2948, 2857, 1719, 1604, 1462, 1415, 1390, 1367, 1257, 1146, 835, 765, 750 cm^{-1}.

***tert*-butyl (1*R*,3a*S*,5a*R*,5b*R*,7a*R*,9*S*,11a*R*,11b*R*,13a*R*,13b*S*)-9-((*tert*-butyldimethylsilyl)oxy)-1-(furan-2-yl)-5a,5b,8,8,11a-pentamethylicosahydro-3a*H*-cyclopenta[*a*]chrysene-3a-carboxylate (14)**

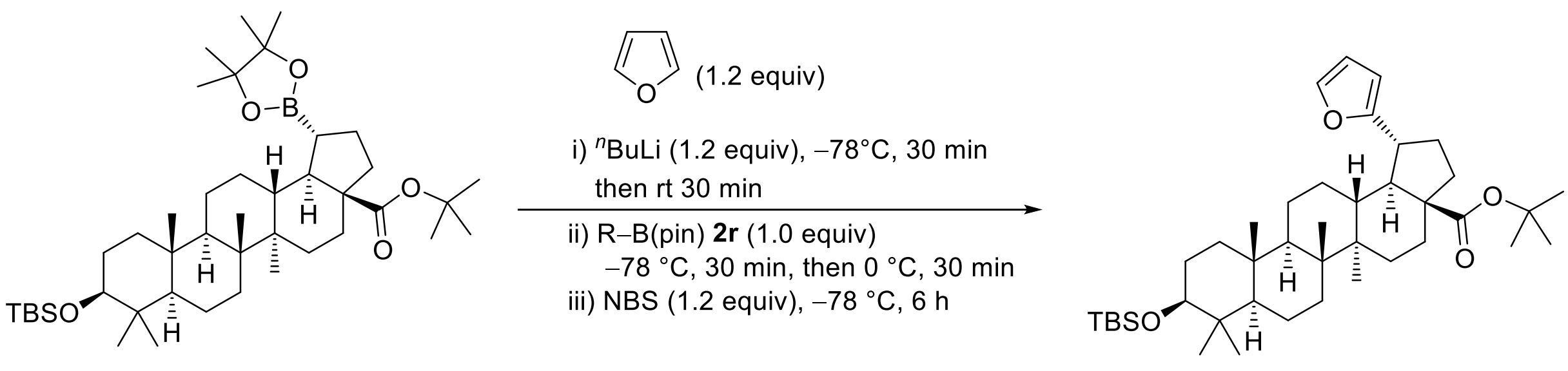

A solution of furan (8.7 μL ,0.12 mmol, 1.2 equiv) in anhydrous THF (1.0 mL) was cooled to –78 °C and treated with ^{n}BuLi (75 μL, 0.12 mmol, 1.2 equiv, 1.6 M in hexanes) under N_2. The mixture was stirred at –78 °C for 30 min before warming to room temperature for 30 min. The mixture was then cooled to –78 °C and a solution of boronic ester **2r** (71.3 mg, 0.100 mmol, 1.00 equiv) in THF (1.0 mL) was added dropwise. The mixture was stirred at –78 °C for 30 min and warmed up to 0 °C and stirred for another 30 min. The reaction was cooled back to –78 °C and a solution of NBS (21.4 mg, 0.120 mmol, 1.20 equiv) in THF (1.0 mL) was added dropwise. The reaction was quenched with saturated aqueous $Na_2S_2O_3$ solution (1 mL) and the reaction mixture was diluted with Et_2O (5 mL) and water (5 mL). The layers were separated, and the aqueous layer was extracted with Et_2O (2 × 5 mL). The combined organic layers were dried over anhydrous $MgSO_4$, filtered and concentrated under reduced pressure. The crude product was purified by flash column chromatography (SiO_2; 50:1 pentane:EtOAc) to afford **14** (35 mg, 0.053 mmol, 53%, >20:1 d.r.) as a white solid.

TLC: R_f = 0.95 (10:1 pentane:EtOAc, CAM).

NMR Spectroscopy (*see spectra*):

^{1}H NMR: (400 MHz, $CDCl_3$) δ 7.27 (dd, J = 1.8, 0.6 Hz, 1H), 6.24 (dd, J = 3.2, 1.8 Hz, 1H), 5.95 (dd, J = 3.2, 0.6 Hz, 1H), 3.54 (td, J = 10.6, 3.6 Hz, 1H), 3.12 (dd, J = 11.2, 4.8 Hz, 1H, C*H*–OTBS), 2.29–2.20 (m, 2H), 2.12–2.01 (m, 1H), 1.92–1.83 (m, 2H), 1.68–1.49 (m, 6H), 1.48 (s, 9H, COOC(C*H₃*)₃), 1.46–1.12 (m, 12H), 0.93 (s, 3H, C*H₃*), 0.92 (s, 3H, C*H₃*), 0.87 (s, 9H, $OSi(CH_3)_2C(C\underline{H_3})_3$), 0.86 (s, 3H, C*H₃*), 0.79 (s, 3H, C*H₃*), 0.70 (s, 3H, C*H₃*), 0.65–0.60 (m, 1H), 0.01 (s, 6H, $OSi(C\underline{H_3})_2C(CH_3)_3$) ppm.

^{13}C NMR: (101 MHz, $CDCl_3$) δ 175.5, 161.4, 140.2, 110.1, 103.7, 79.9, 79.6, 56.7, 55.6, 51.9, 50.6, 42.5,

40.9, 39.6, 38.9, 38.8, 38.0, 37.2, 36.8, 34.5, 32.3, 31.3, 29.7, 28.5, 28.2, 28.0, 26.1, 25.7, 21.0, 18.6, 18.3, 16.3 16.1, 16.0, 14.8, –3.6, –4.8 ppm.

HRMS (MALDI) calculated for $C_{41}H_{68}O_4SiNa$ $[M+Na]^+$ 675.4779, found 675.4772.

IR (neat) ν_{max}: 2931, 2856, 1802, 1717, 1462, 1366, 1275, 1260, 1147, 836, 764, 750 cm^{-1}.

3. MECHANISTIC STUDIES

3.1. Proposed mechanism for the Fe–catalyzed dealkenylative borylation

Ozonolysis

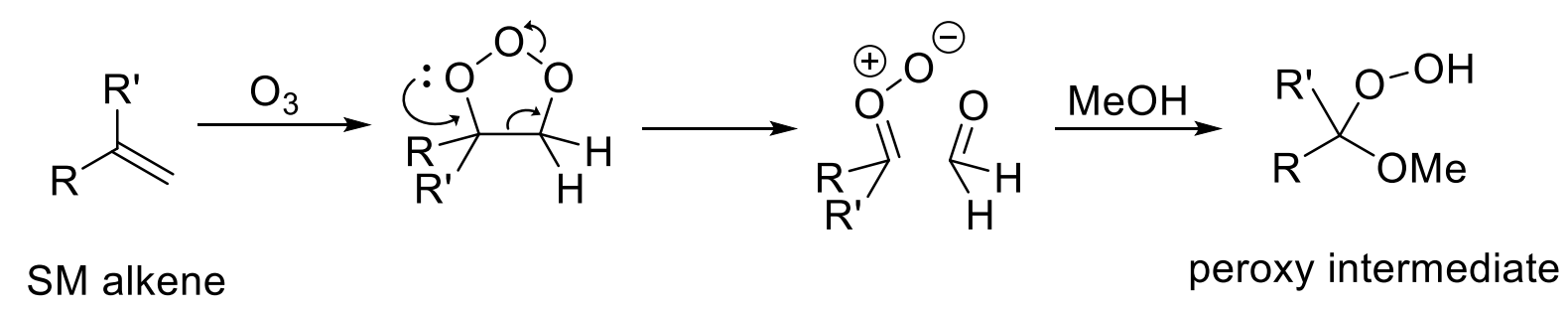

Fe(III) precatalyst activation

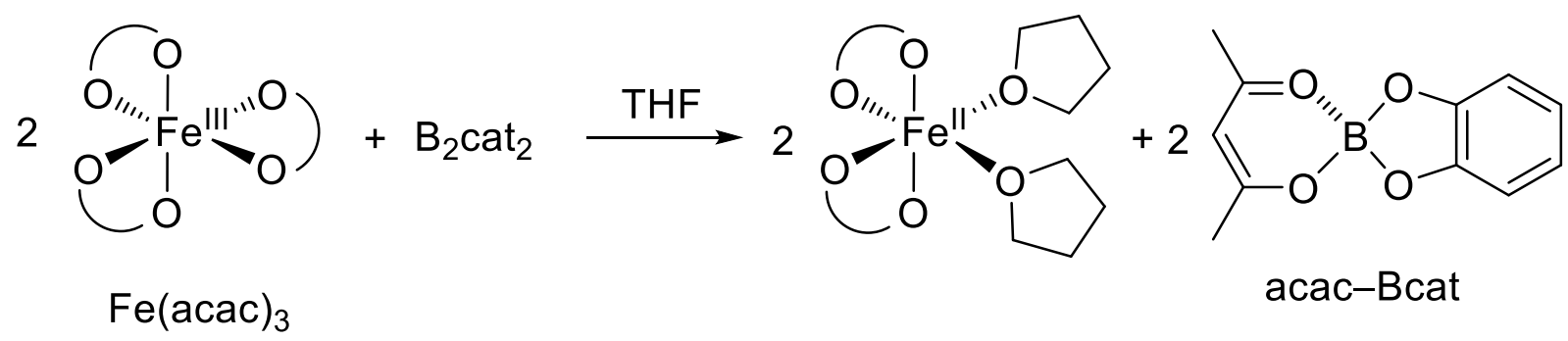

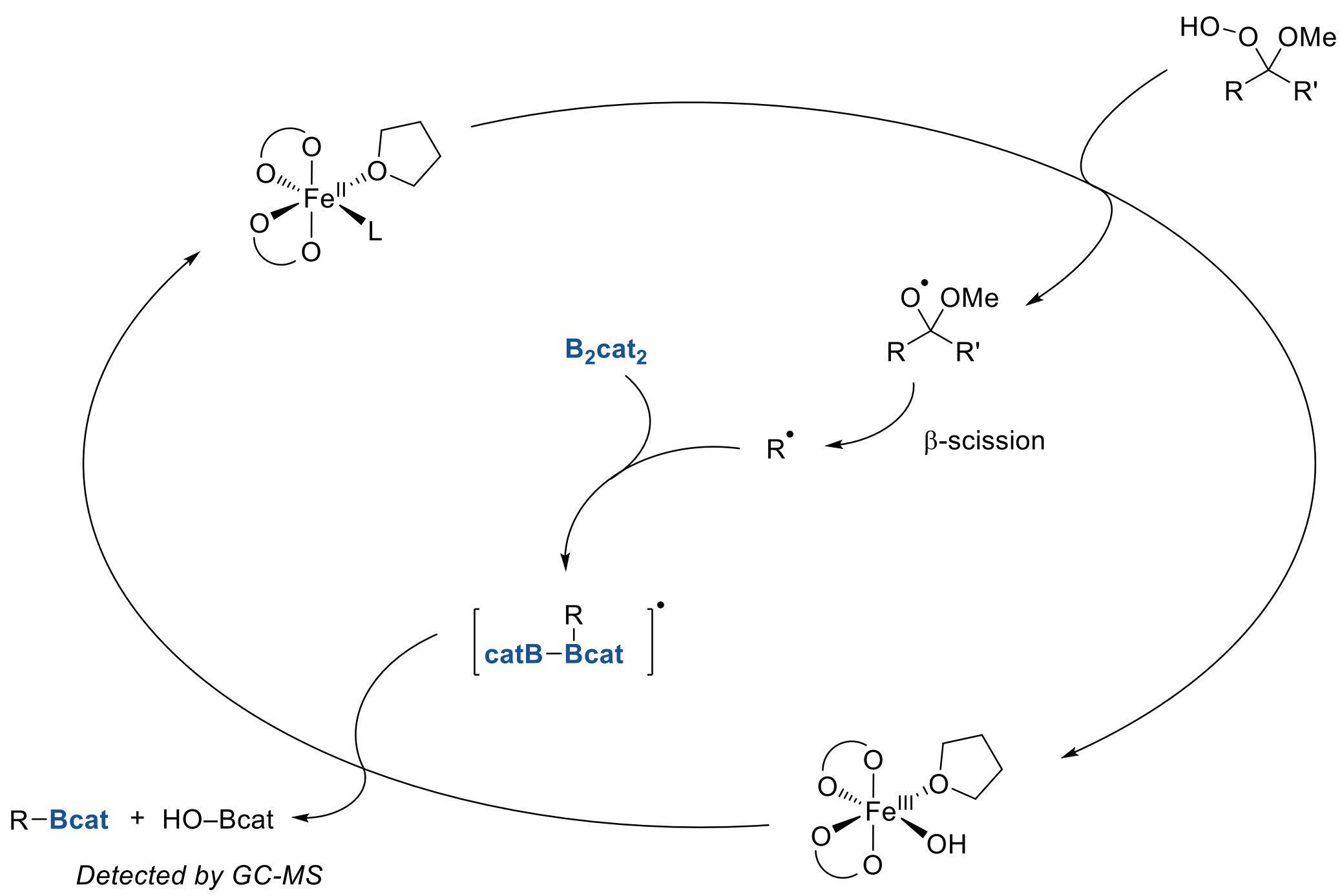

Figure S3. Proposed catalytic cycle for the Fe–catalyzed dealkenylative borylation.

3.2. Proposed mechanism for the formation of side products

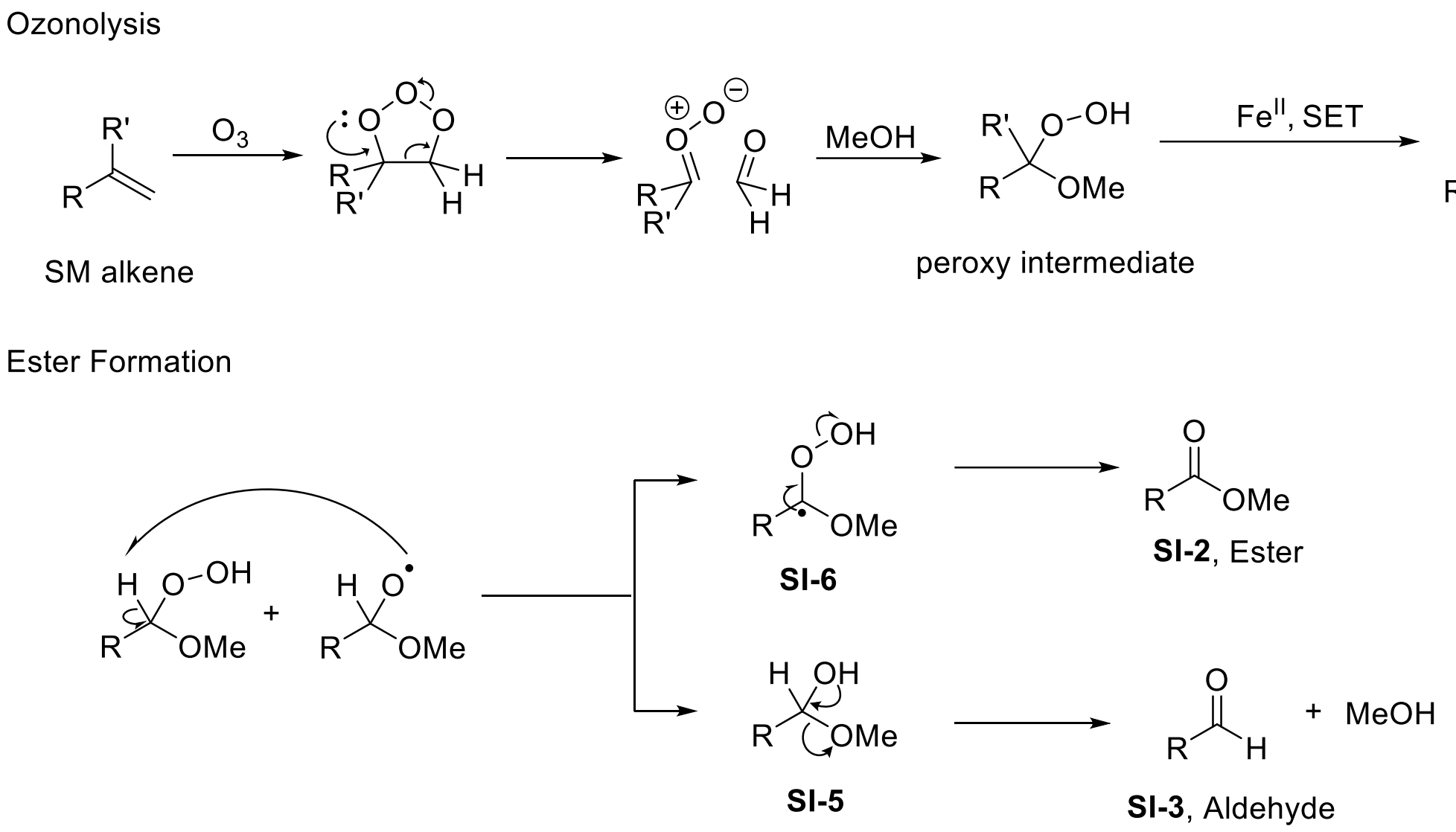

Figure S4. Proposed mechanism for the formation of ester/aldehyde side products.

We propose that the formation of the ester side product can occur when the peroxy acetal intermediate after ozonolysis is derived from a terminal alkene (**Figure S5**). Upon reduction, it is proposed that HAT of the hydroxy radical with another equivalent of the peroxy acetal, generating hemiacetal **SI-5** that collapses to form the aldehyde side product **SI-3**. The α-peroxy carbon-centered radical **SI-6** then undergoes radical elimination to form ester side product **SI-2**.

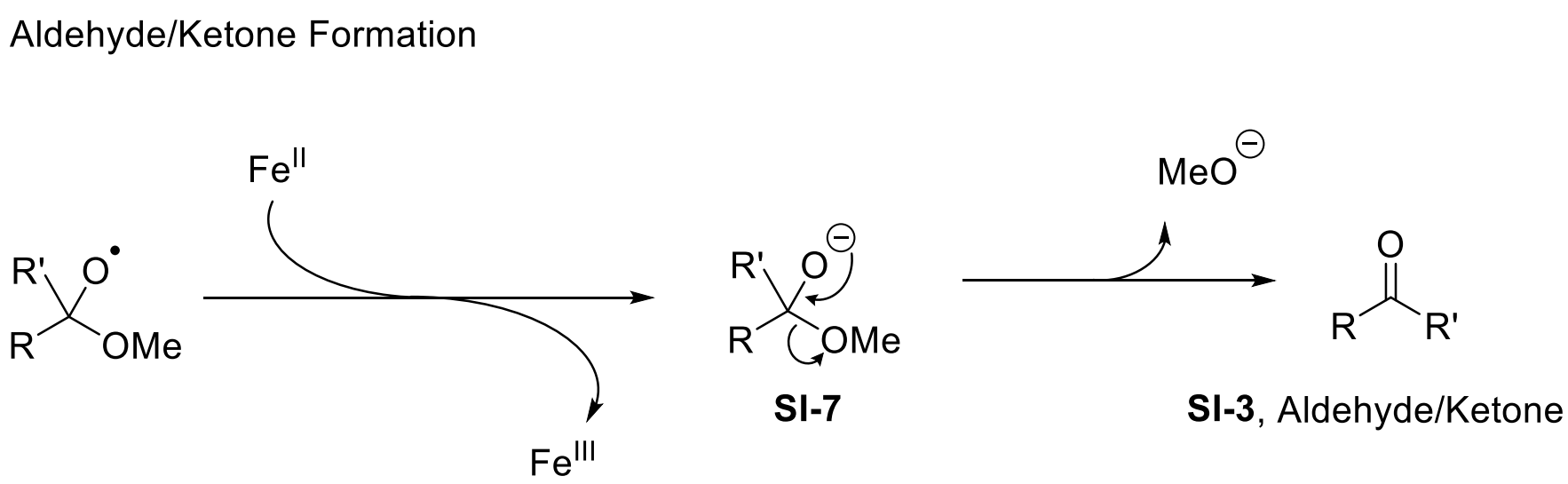

Figure S5. Proposed mechanism for the formation of aldehyde/ketone side products.

Alternatively, aldehyde/ketone side products can derive from the same alkoxy radical intermediate, even when the peroxy acetal is derived from a non-terminal alkene. Here, the alkoxy radical intermediate can be further reduced by Fe(II) to form alkoxide **SI-7**, which can then generate the corresponding aldehyde or ketone side products **SI-3**.

An important conclusion to be drawn is that when R' is not a hydrogen atom, the formation of the ester side product is not feasible. This conclusion aligns with the experimental data and explains why higher yields are achieved for 1,1-disubstitued terminal alkenes and exocyclic alkenes.

3.3. Radical trapping experiment with TEMPO

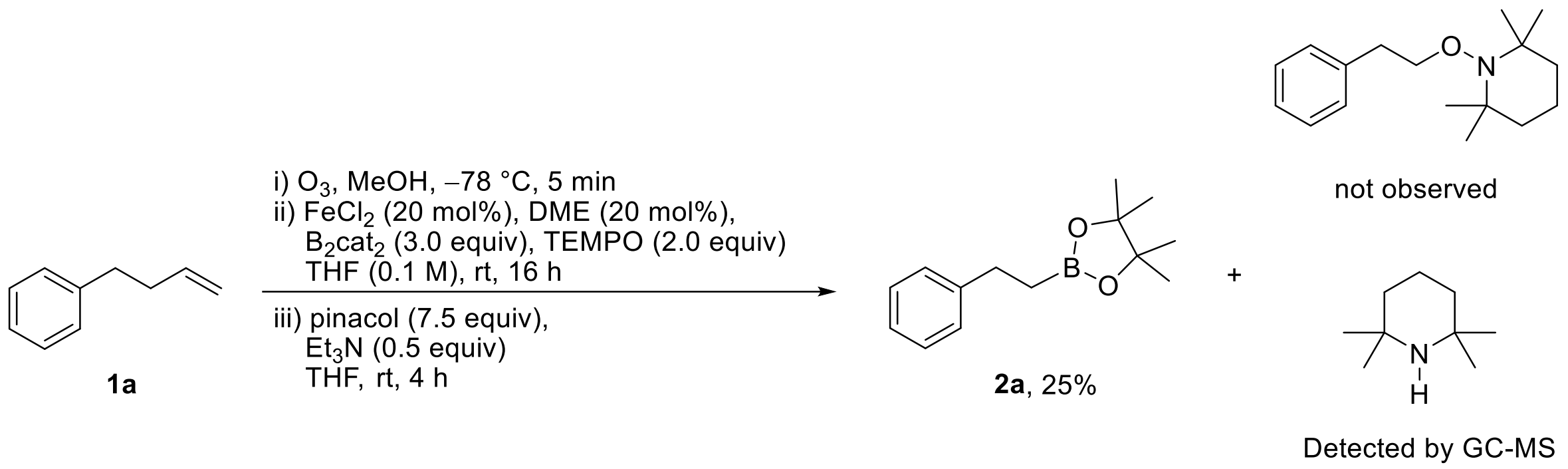

Radical trapping experiment was conducted by adding TEMPO (2.0 equiv) to the standard reaction mixture, which resulted in lower yields of the corresponding product. However, this result merely reflects the incompatibility of B_2cat_2 with TEMPO, which are known to react with each other, and is therefore inconclusive. No radical adducts with TEMPO were observed in this experiment.[23]

3.4. Fe(III) catalyst activation studies

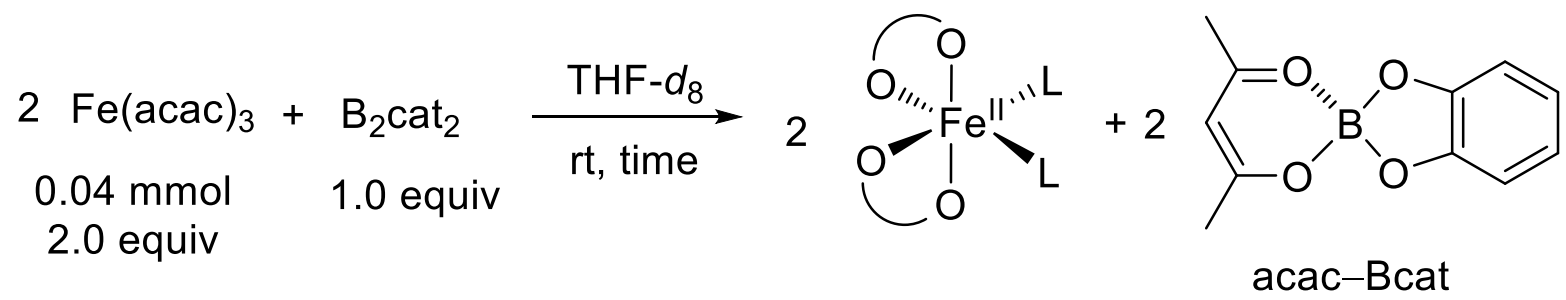

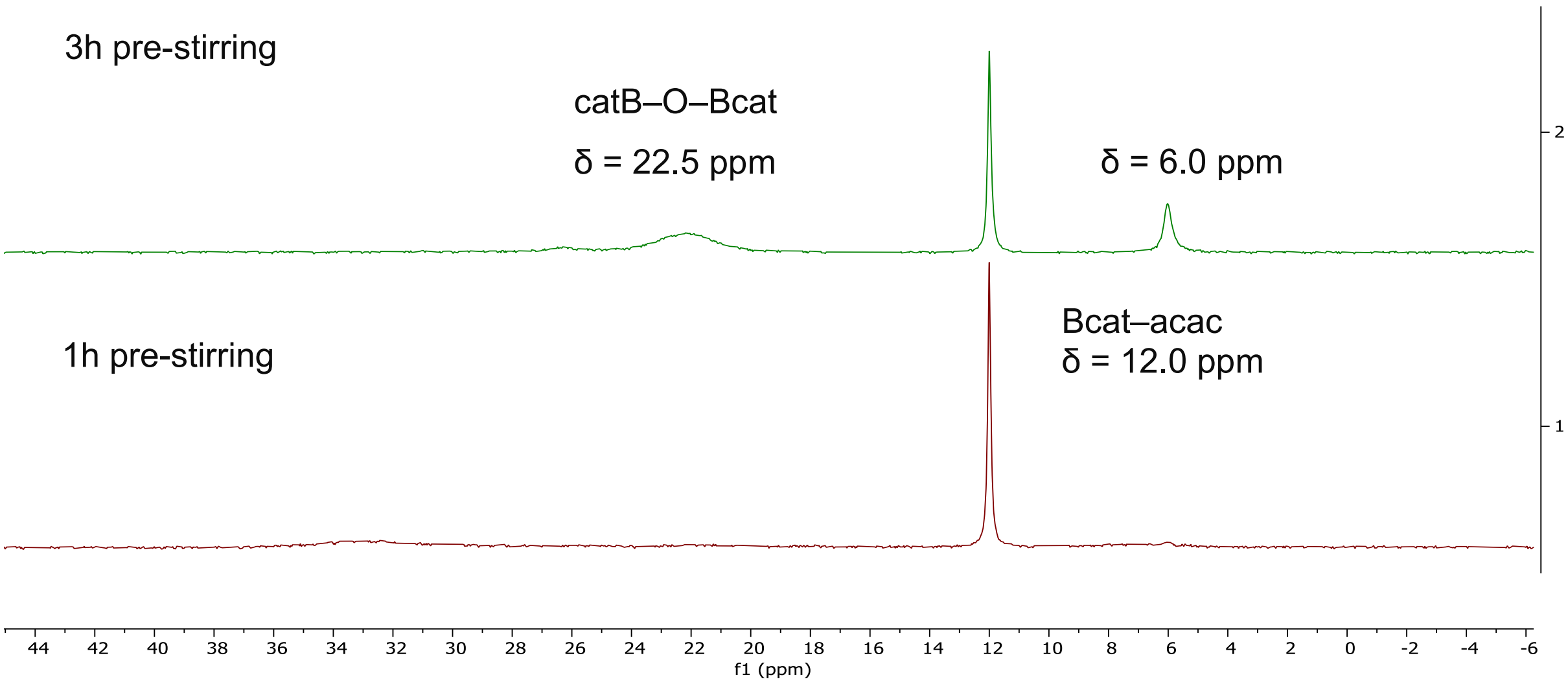

Figure S6. Pre-stirring of the reaction mixture

A flame-dried 8 mL Biotage® microwave reaction vial equipped with a magnetic stirrer bar was charged with $Fe(acac)_3$ (14.1 mg, 40.0 µmol, 1.00 equiv) and B_2cat_2 (9.5 mg, 40 µmol, 1.0 equiv) inside an argon-filled glovebox. The vial was sealed with a cap, taken outside of the glovebox, and THF-*d*$_8$ (1 mL) was added. The mixture was allowed to stir at room temperature for the time indicated in the figure above (1 h and 3 h respectively) and immediately submitted to ^{11}B NMR.

As indicated in the spectra shown above, after 1 h of stirring, the peak for B_2cat_2 is no longer visible and a peak at 12.0 ppm is observed. We propose that this peak corresponds to Bcat–acac and therefore synthesized this

compound independently to validate this hypothesis (**Section 3.4.2**).

3.4.1. ^{1}H NMR of the pre-stirring mixture using either $Fe(acac)_3$ or $FeCl_2$

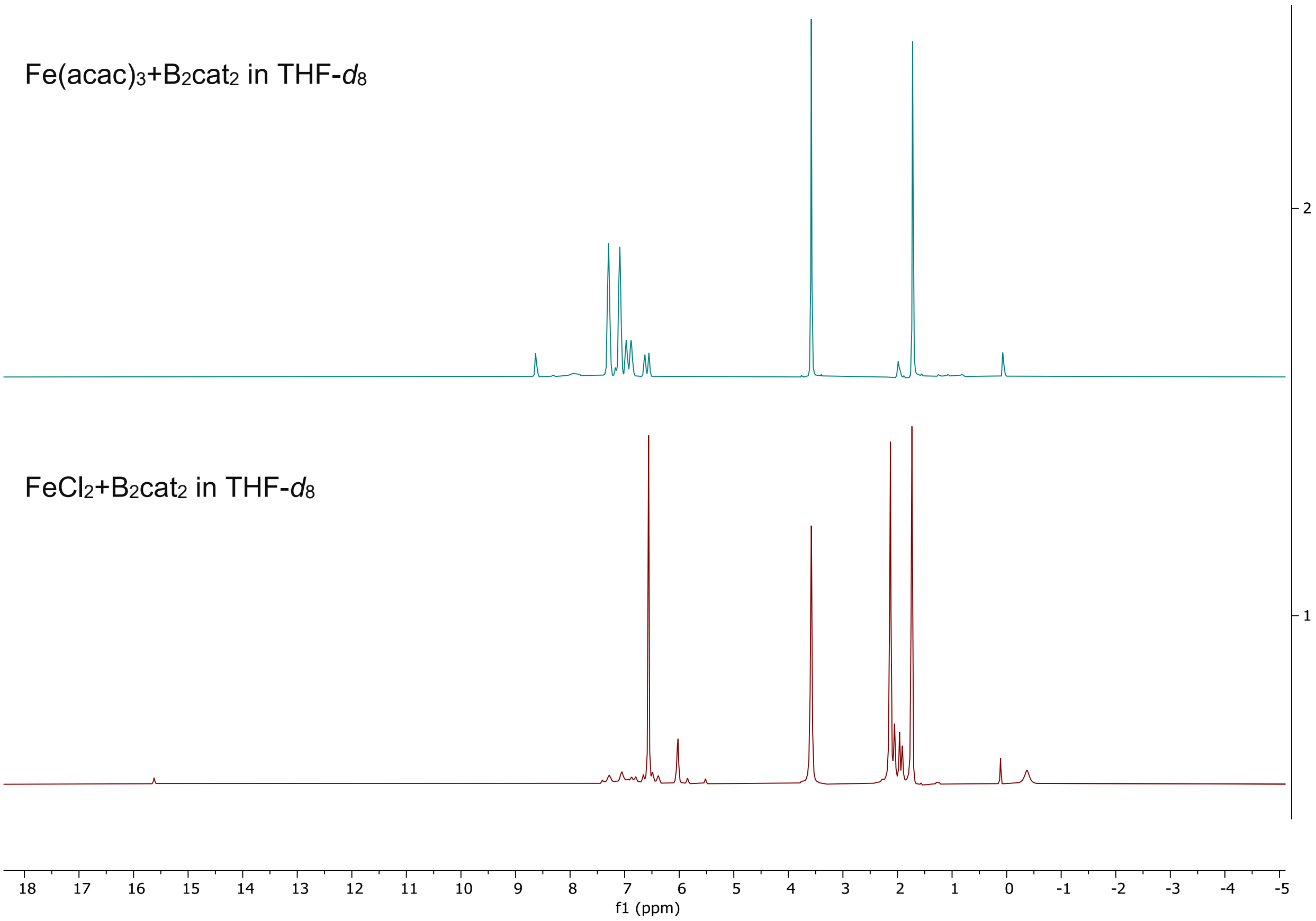

Figure S7. ^{1}H NMR of the reaction mixture after pre-stirring

As demonstrated by the spectra above, after pre-stirring the iron catalyst with B_2cat_2 in THF-d_8 for 3 h, the ^{1}H NMR showed sharp peaks that indicate that the complete reduction of paramagnetic Fe(**III**) to diamagnetic Fe(**II**) has occurred.

3.4.2. Independent synthesis of Bcat–acac

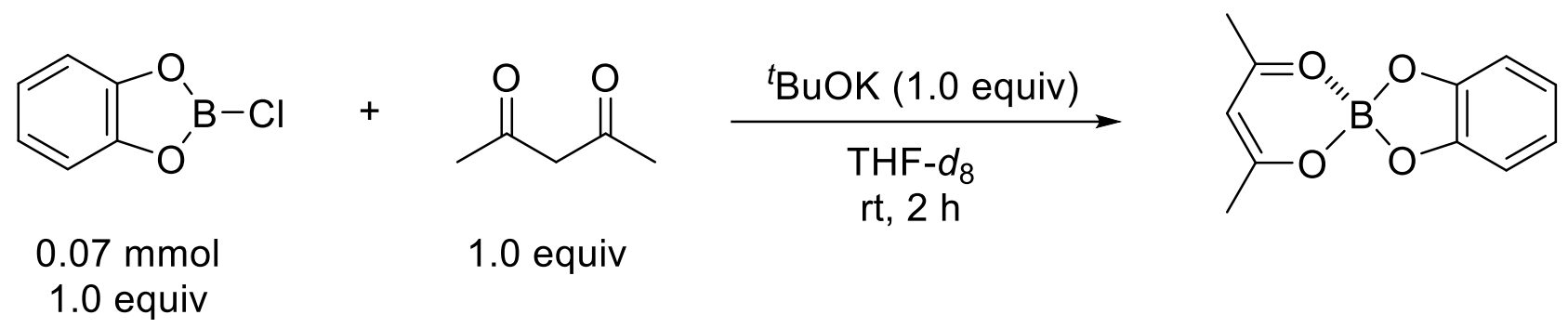

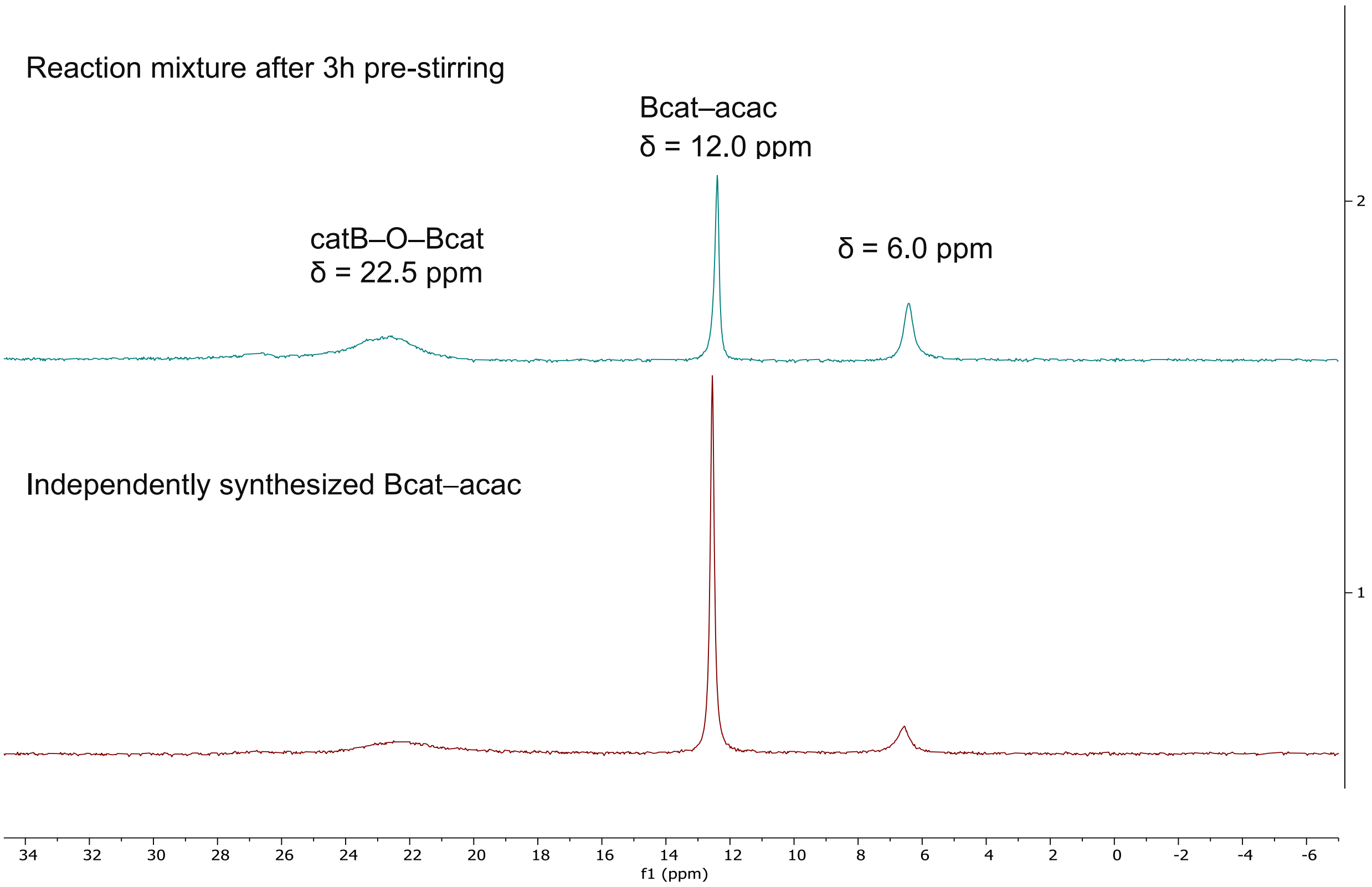

Figure S8. Independent synthesis of Bcat–acac and comparison of ^{11}B NMR

A flame-dried 8 mL Biotage® microwave reaction vial equipped with a magnetic stirrer bar was charged with 2-chloro-1,3,2-benzodioxaborole (15.4 mg, 0.100 mmol, 1.00 equiv) and potassium *tert*-butoxide (11.2 mg, 0.100 mmol, 1.00 equiv) inside an argon-filled glovebox. The vial was sealed with a cap, taken outside of the glovebox, and THF-d_8 (1 mL) and acetylacetone (10.0 mg, 0.100 mmol, 1.00 equiv) were added. The mixture was allowed to stir at room temperature for 2 h and immediately submitted to ^{11}B NMR.

^{11}B NMR spectra of the pre-stirring mixture matched that of independently synthesized Bcat–acac, confirming that Fe(**III**) reduction to a catalytically active Fe(**II**) complex occurs during pre-stirring with B_2cat_2.

4. X-RAY CRYSTALLOGRAPHIC ANALYSIS

4.1. Compound 2p (CCDC 2555898)

Experimental: Single colorless block-shaped-shaped crystals of **2p** were chosen from the sample as supplied.. A suitable crystal 0.194×0.101×0.053 mm^3 was selected and mounted on a MITIGEN holder in oil on a Rigaku 007HF diffractometer equipped with Arc)Sec VHF Varimax confocal mirrors and a UG2 goniometer and HyPix Arc-100 detector. The crystal was kept at a steady T = 100(2) K during data collection. The structure was solved with the ShelXT 2018/2 (Sheldrick, 2018) structure solution program using dual methods solution method and by using Olex2 1.5 (Dolomanov et al., 2009) as the graphical interface. The model was refined with version 2018/3 of ShelXL 2018/3 (Sheldrick, 2015) using full matrix least squares minimization on F^2 minimization.

Refine Special Details: The crystal was found to be twinned, and the best refinement was obtained using the hkl4 file. O1w displayed an elongated thermal ellipsoid, and has been modelled over two sites. These were initially modelled with complementary occupancies that were freely refined (with EADP constraints on the two sites), but these converged to a ratio of approximately 54:46. This was rounded to 0.5 for both sites. Hydrogen atoms were located in the difference map, given a U_{iso} of 1.5 times that of O1w/O2w, and fixed at an O-H distance of 0.84(1) A. One hydrogen position was common to both sites. Peaks of electron density near to the pendant dioxaborolane indicated that there may be a secondary orientation, despite acceptable thermal ellipsoids of the constituent atoms. A second orientation was manually modelled and the two orientations given complementary occupancies. The B-O bond lengths, and the two O4-C30 bond lengths, were restrained to have equal values using SADI commands. Minor disorder of O2 was modelled in a similar way. Pairs of equivalent atoms have been constrained to have equal U_{ij} values using EADP commands.

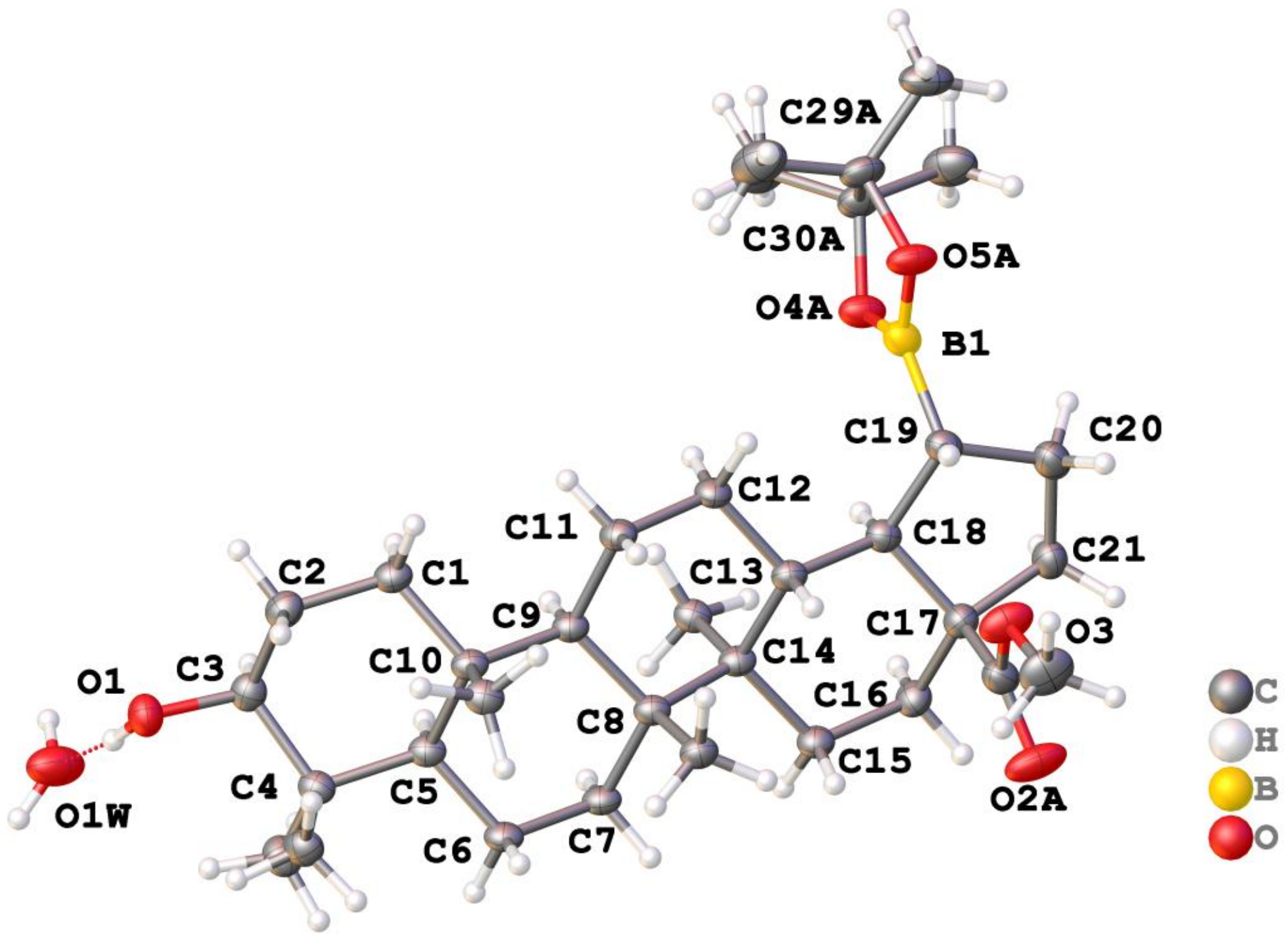

Figure S9. Compound 2p (CCDC 2555898)

Table S7 Crystal Data and structure refinement for compound 2p (CCDC 2555898)

**Compound**	**2p**
Formula	$C_{34}H_{59}BO_6$
$D_{calc.}$/ g cm^{-3}	1.153
m/mm^{-1}	0.600
Formula Weight	574.62
Color	colorless
Shape	block-shaped
Size/mm^3	0.194×0.101×0.053
T/K	100(2)
Crystal System	monoclinic
Flack Parameter	-0.10(12)
Hooft Parameter	-0.12(9)
Space Group	$P2_1$
a/Å	14.5593(4)
b/Å	6.9807(2)
c/Å	16.6823(4)
a/°	90
b/°	102.635(3)
g/°	90
V/Å^3	1654.43(8)
Z	2
Z'	1
Wavelength/Å	1.54184
Radiation type	Cu K_a
Q_{min}/°	3.111
Q_{max}/°	73.636
Measured Refl's.	50273
Indep't Refl's	6435
Refl's I≥2 s(I)	5941
R_{int}	0.1072
Parameters	428
Restraints	14
Largest Peak	0.214
Deepest Hole	-0.236
GooF	1.035
wR_2 (all data)	0.1064
wR_2	0.1017
R_1 (all data)	0.0476
R_1	0.0428

5. SPECTROSCOPIC DATA

^{1}H NMR (400 MHz, $CDCl_3$) of **1b** (*see procedure*)

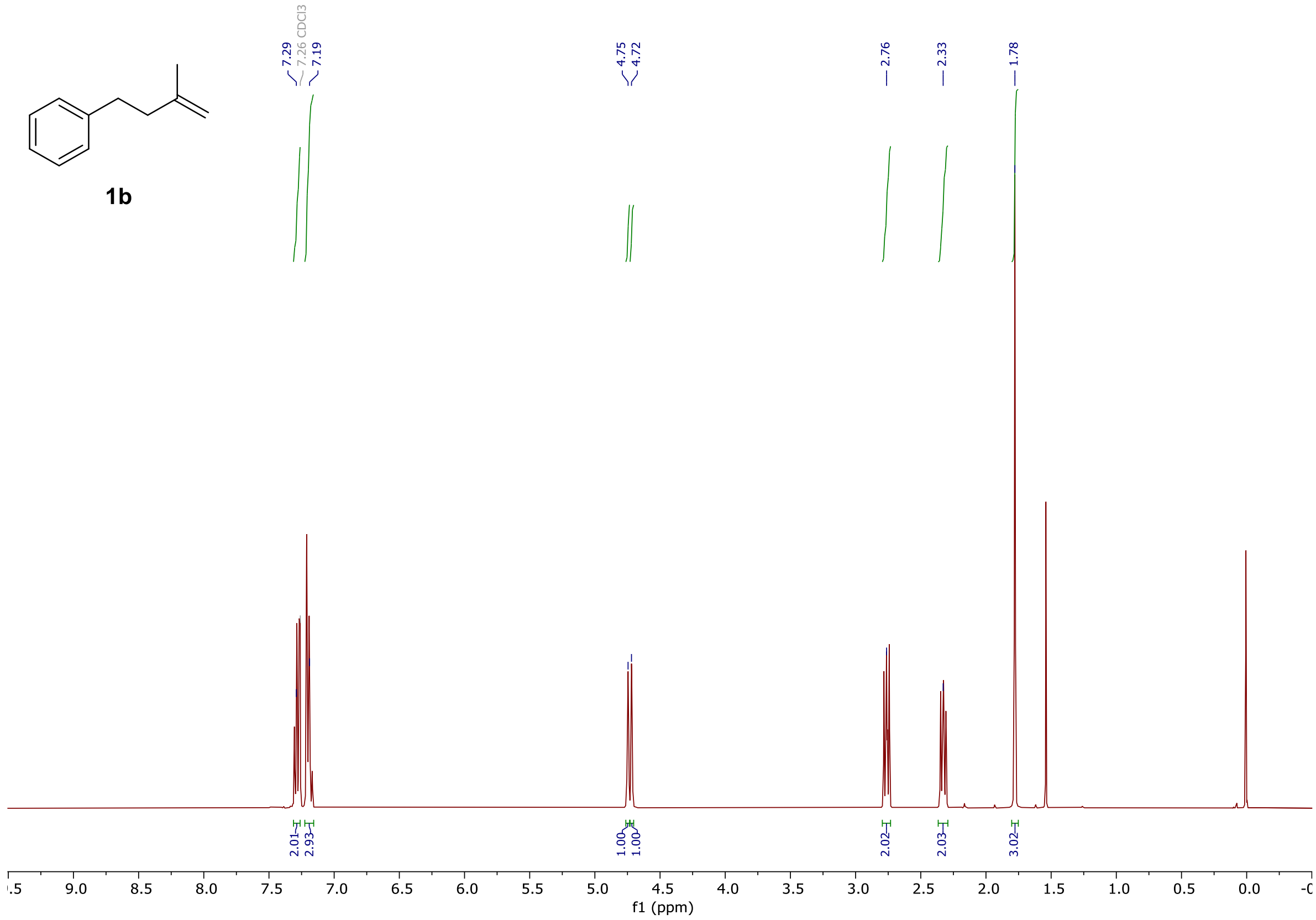

^{13}C NMR (101 MHz, $CDCl_3$) of **1b**

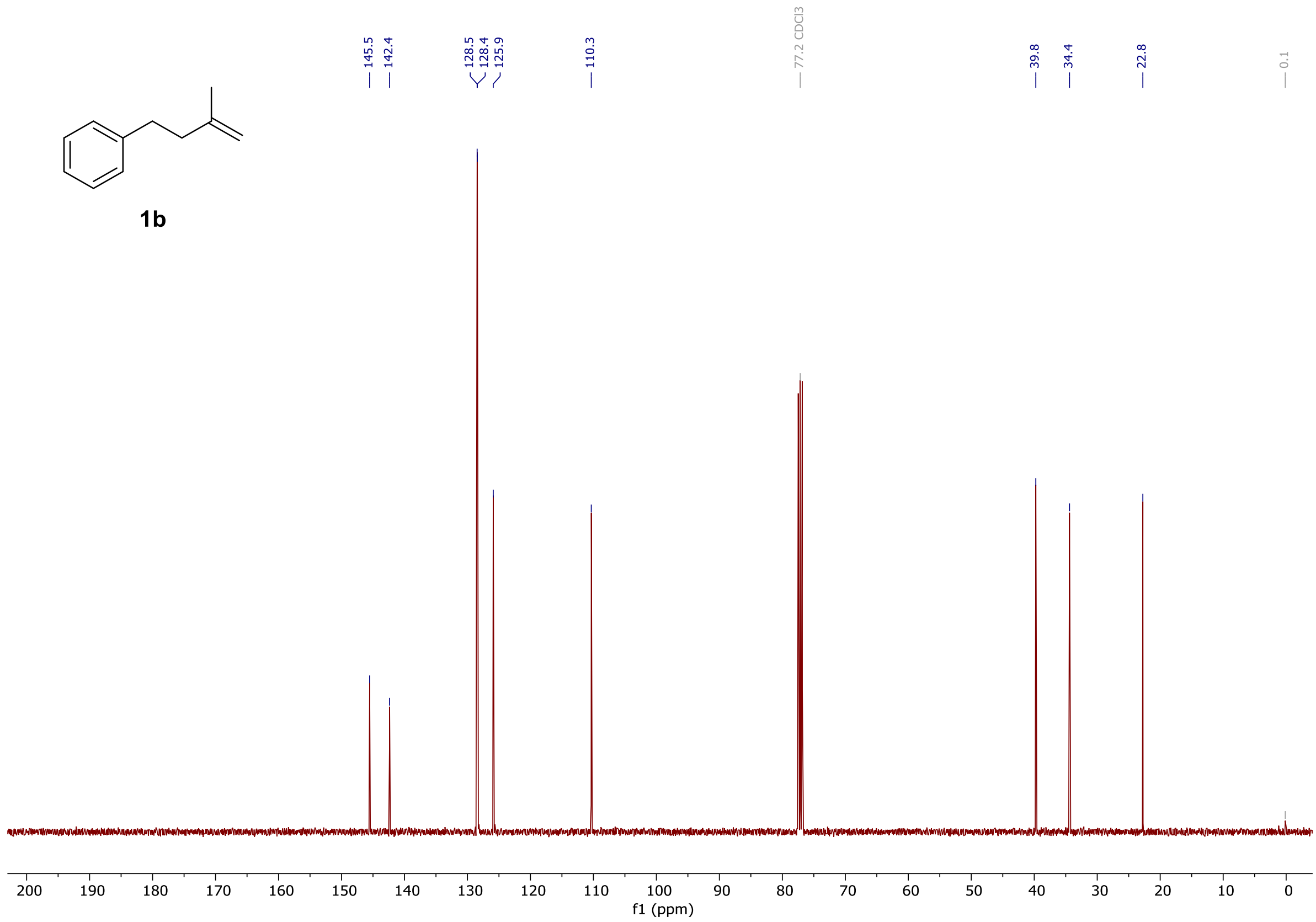

^{1}H NMR (400 MHz, $CDCl_3$) of **1d** (*see procedure*)

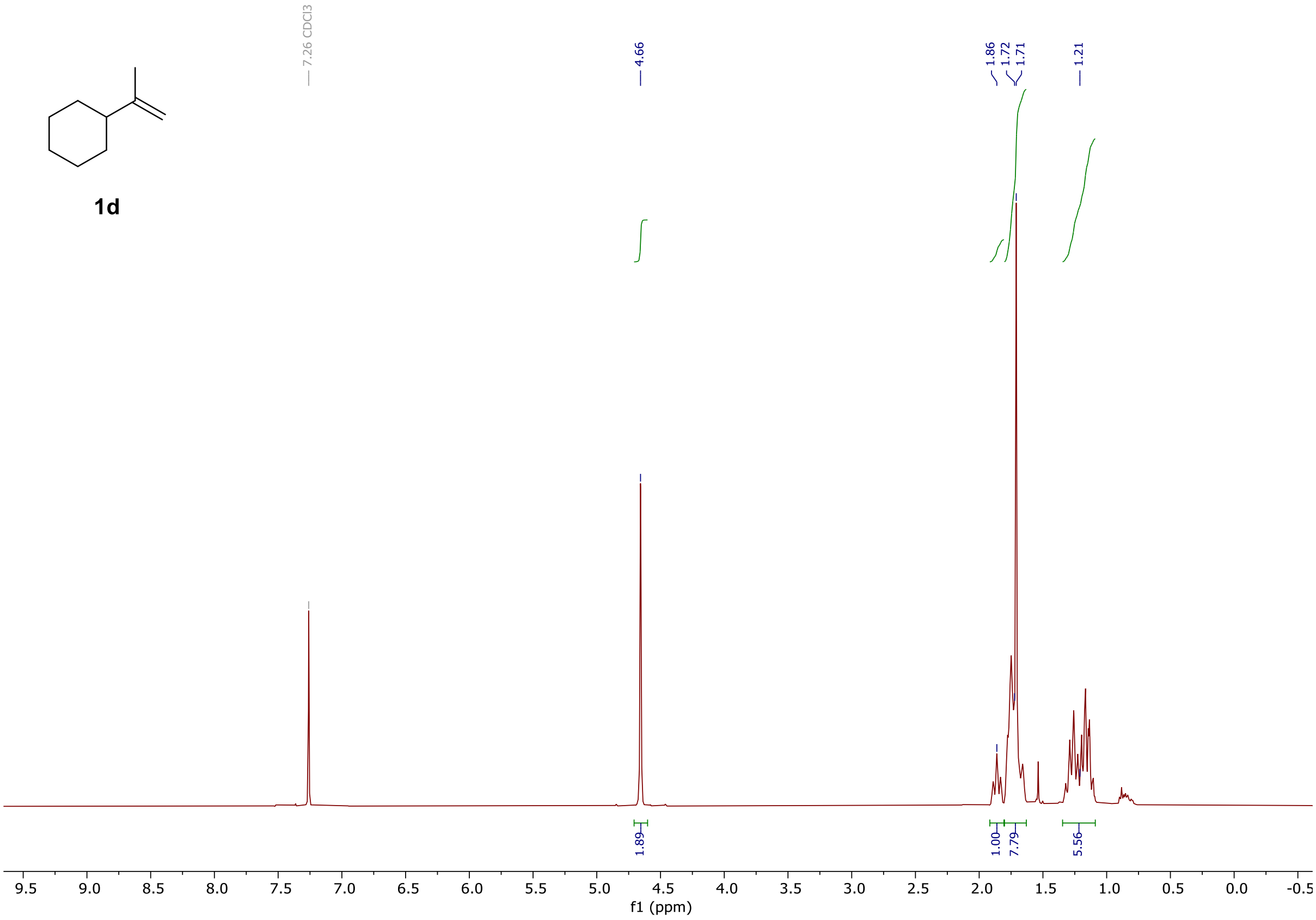

^{13}C NMR (101 MHz, $CDCl_3$) of **1d**

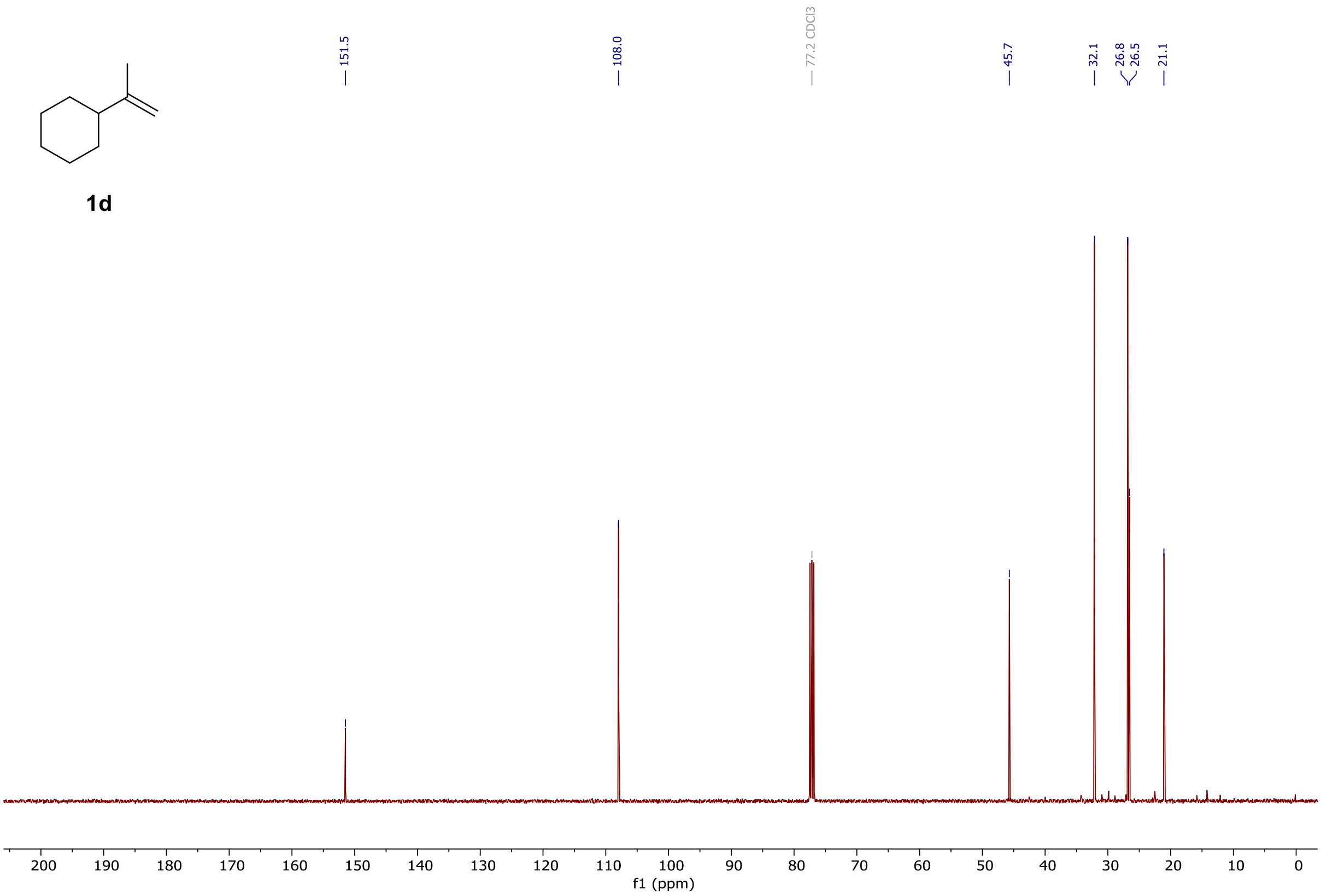

^{1}H NMR (400 MHz, $CDCl_3$) of **1e** (*see procedure*)

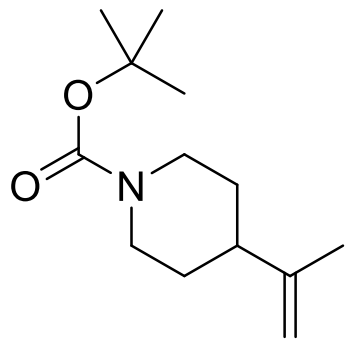

1e

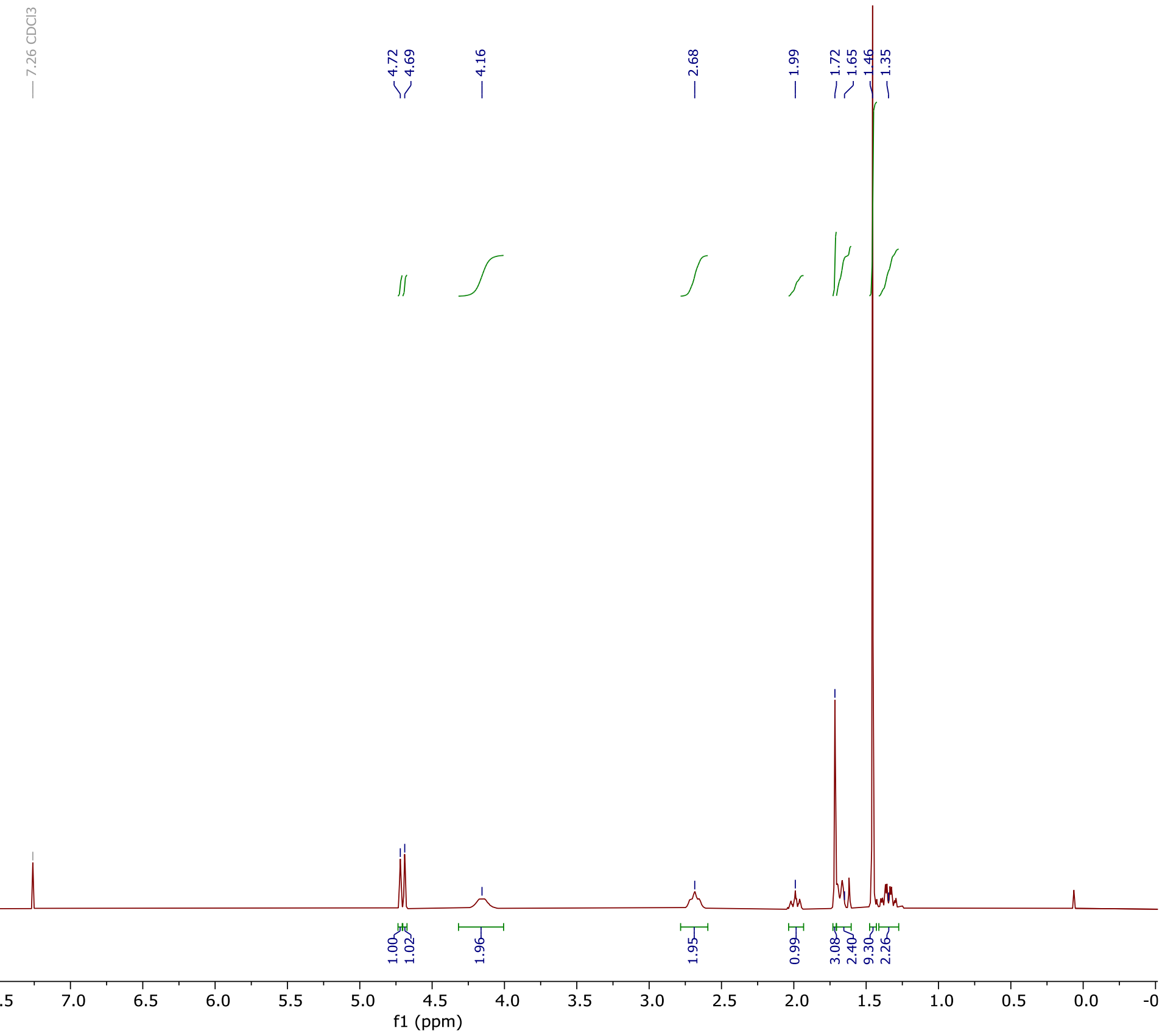

^{13}C NMR (101 MHz, $CDCl_3$) of **1e**

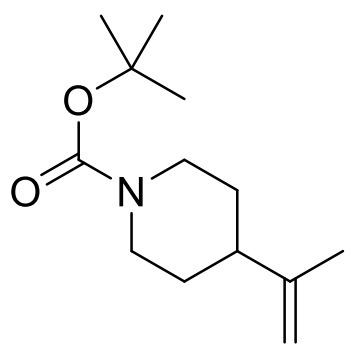

1e

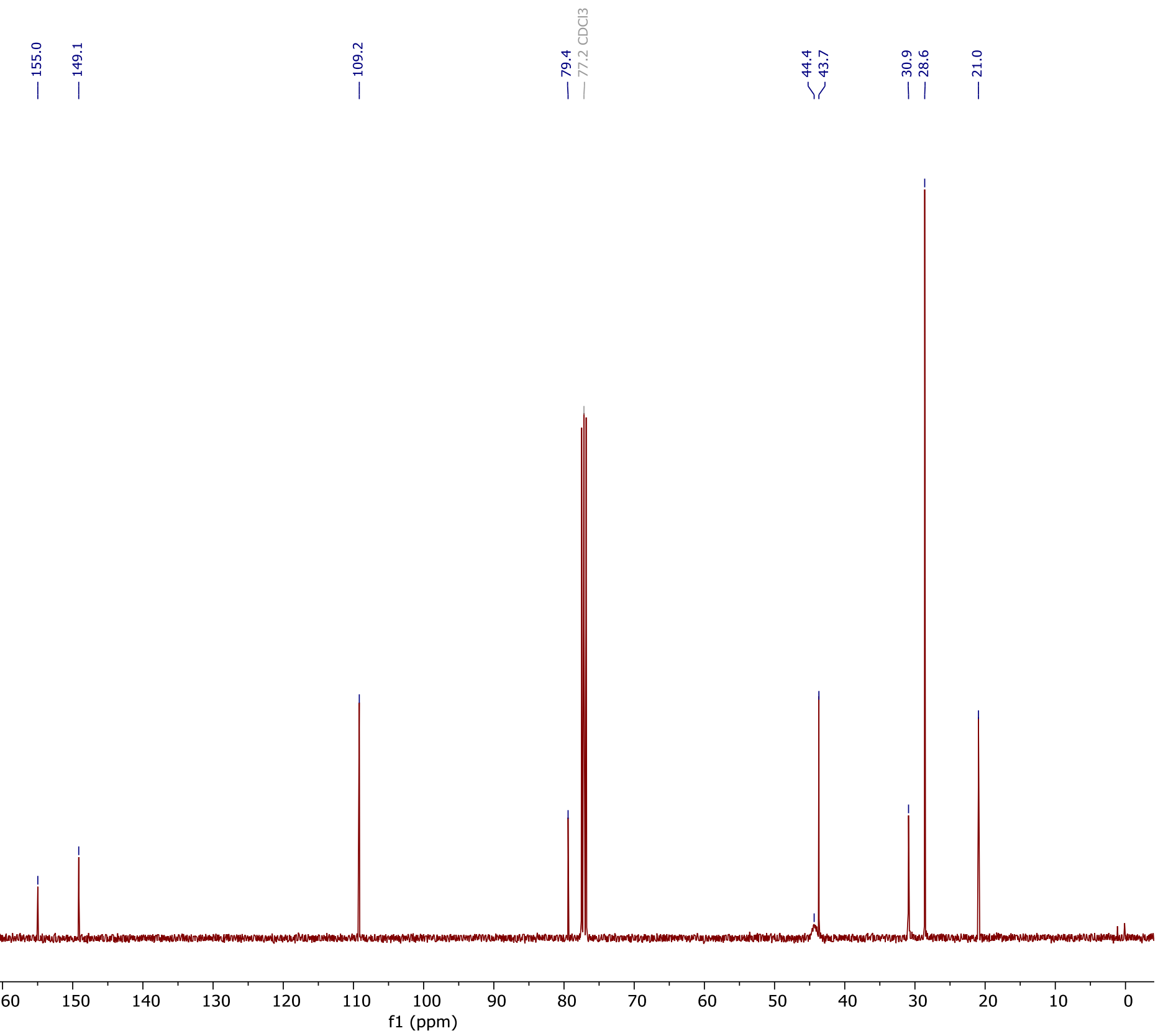

^{1}H NMR (400 MHz, $CDCl_3$) of **1h** (see procedure)

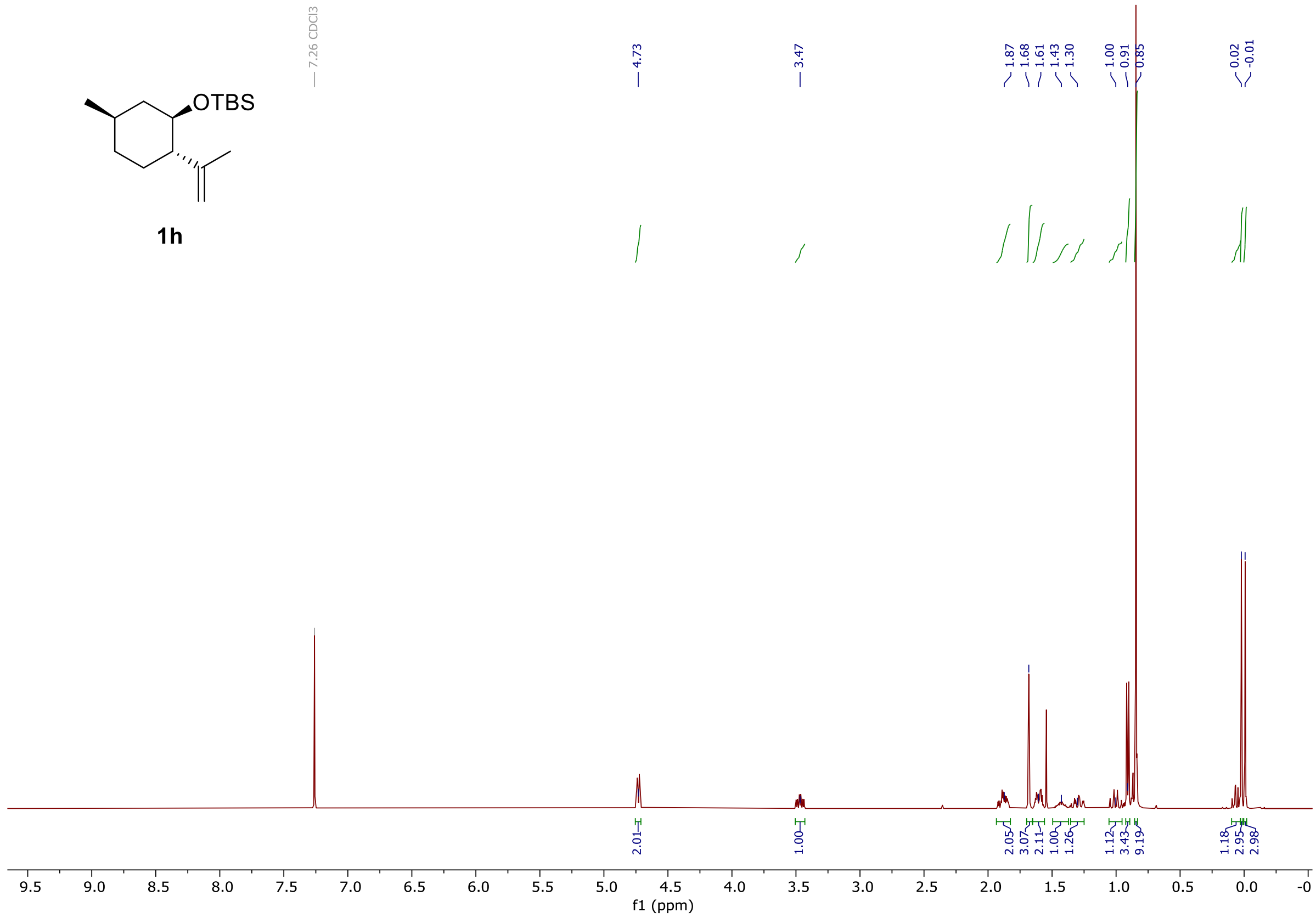

^{13}C NMR (101 MHz, $CDCl_3$) of **1h**

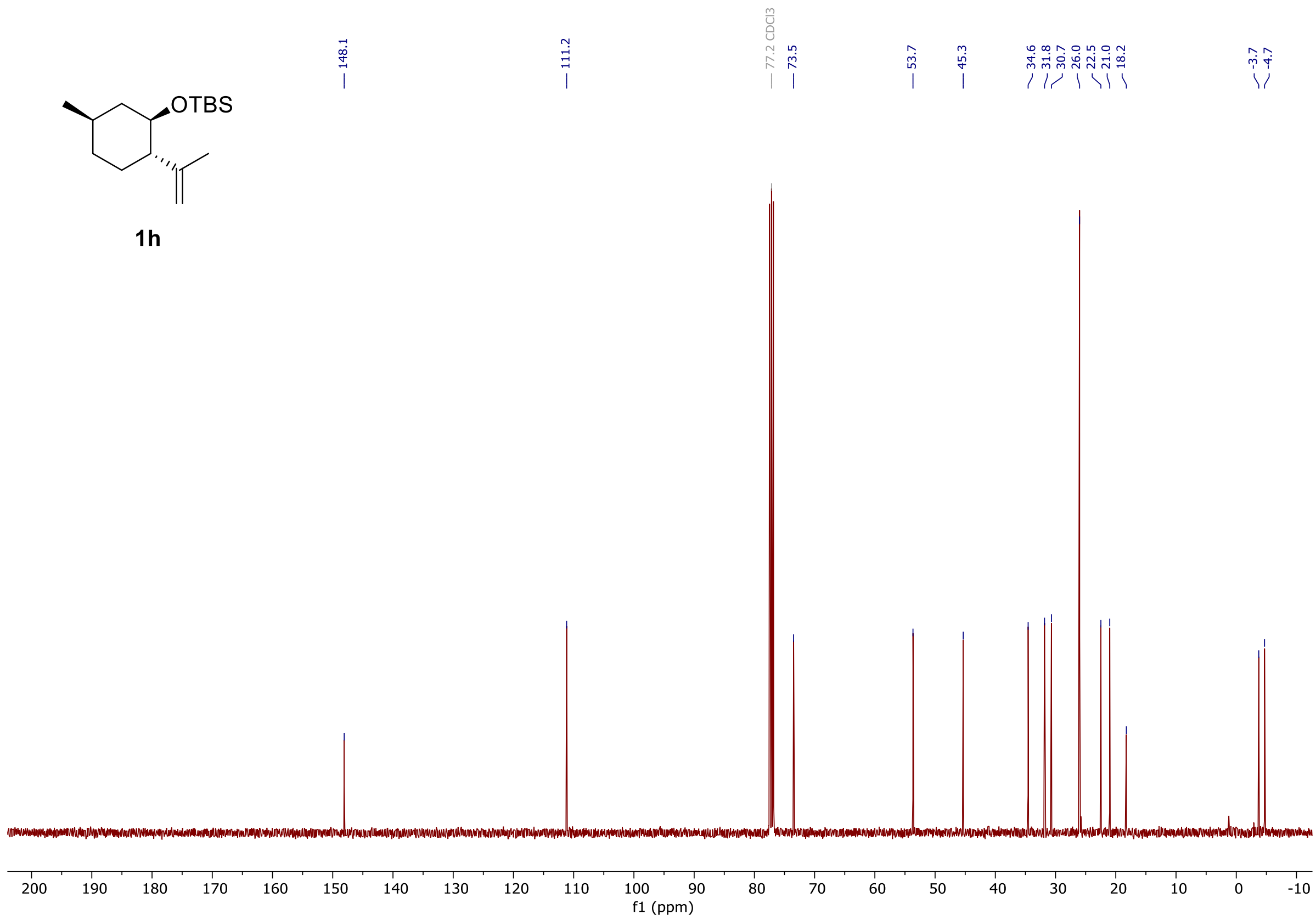

^{1}H NMR (400 MHz, $CDCl_3$) of **1j** (*see procedure*)

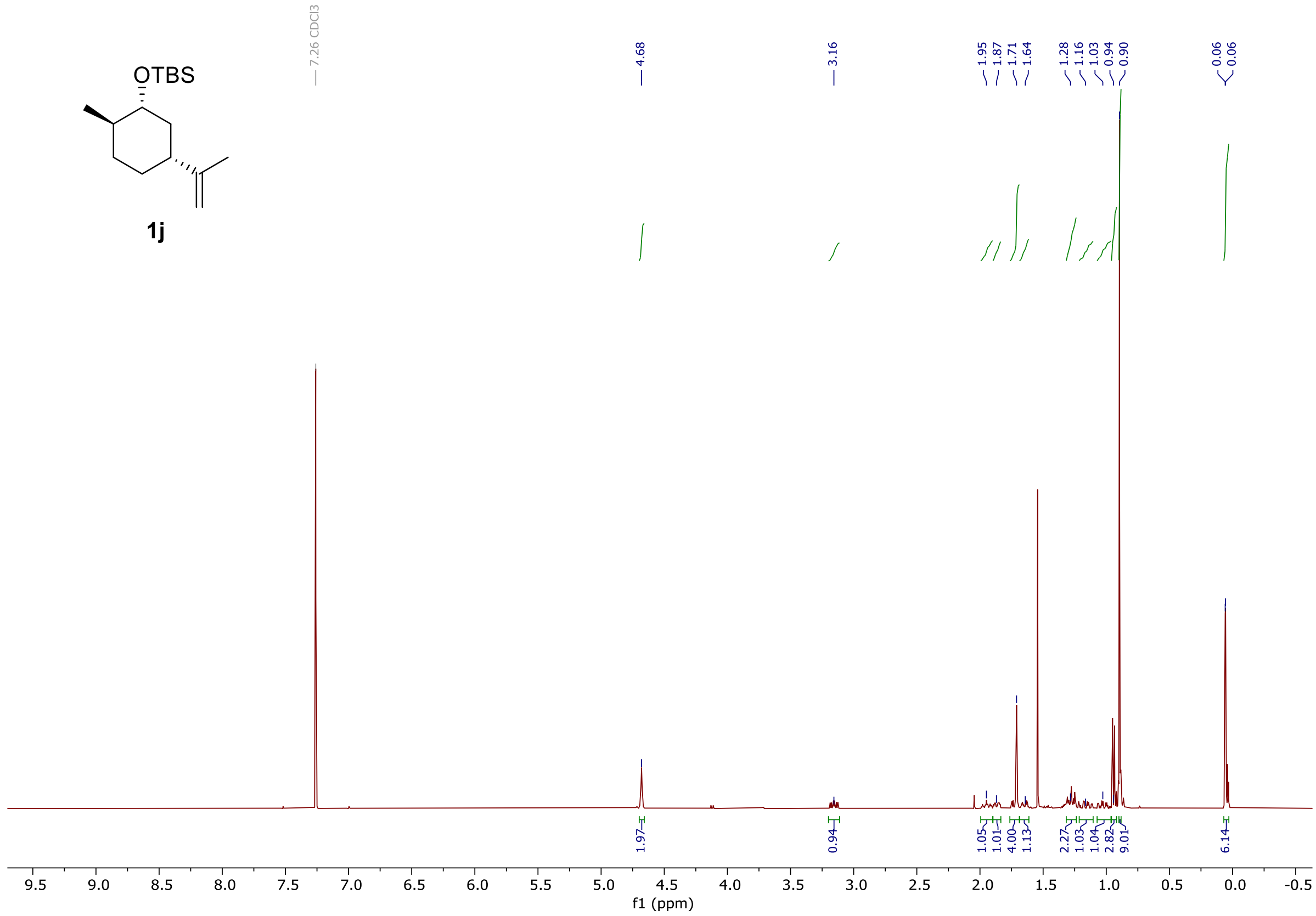

^{13}C NMR (101 MHz, $CDCl_3$) of **1j**

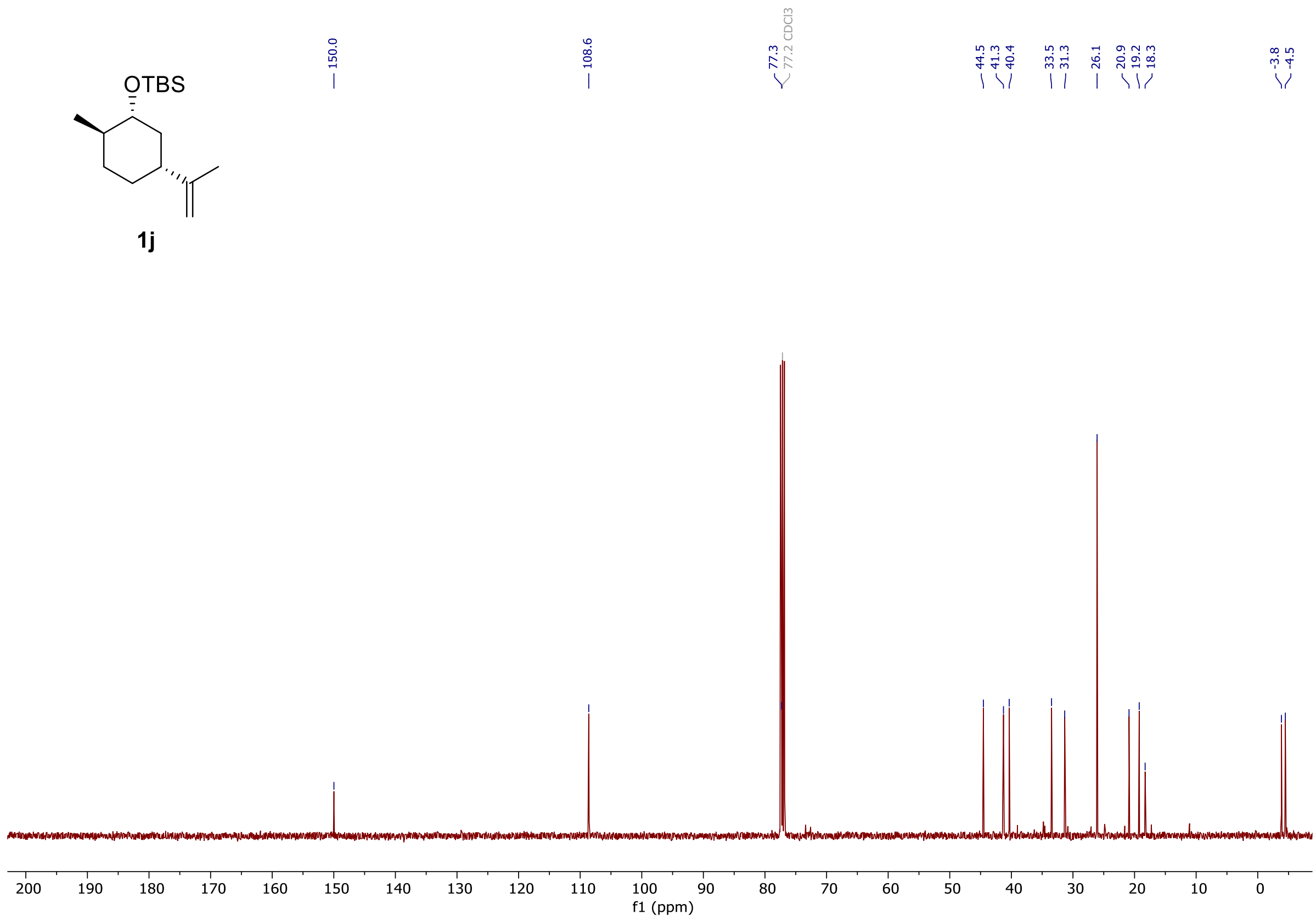

^{1}H NMR (400 MHz, $CDCl_3$) of **1n** (*see procedure*)

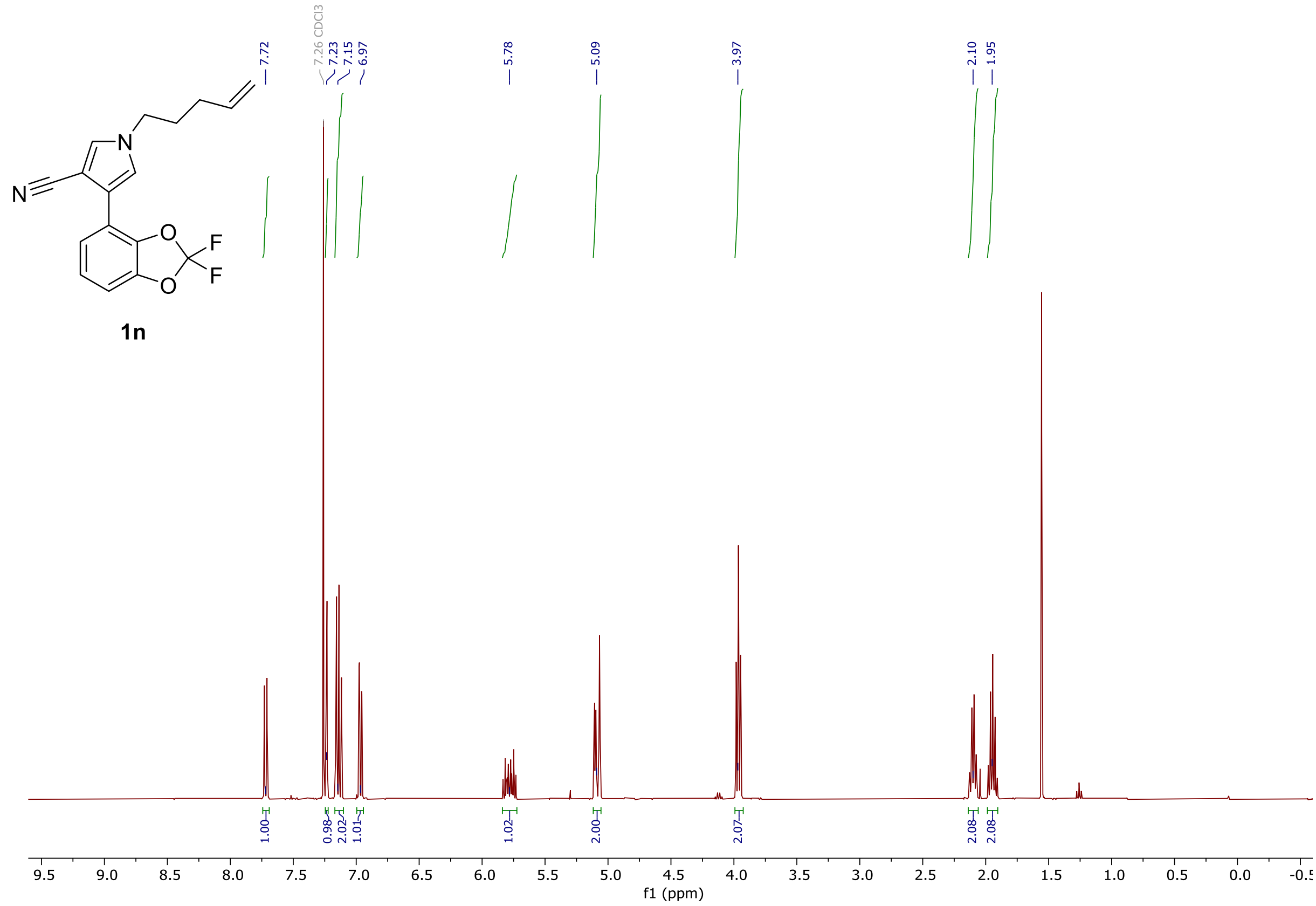

^{13}C NMR (101 MHz, $CDCl_3$) of **1n**

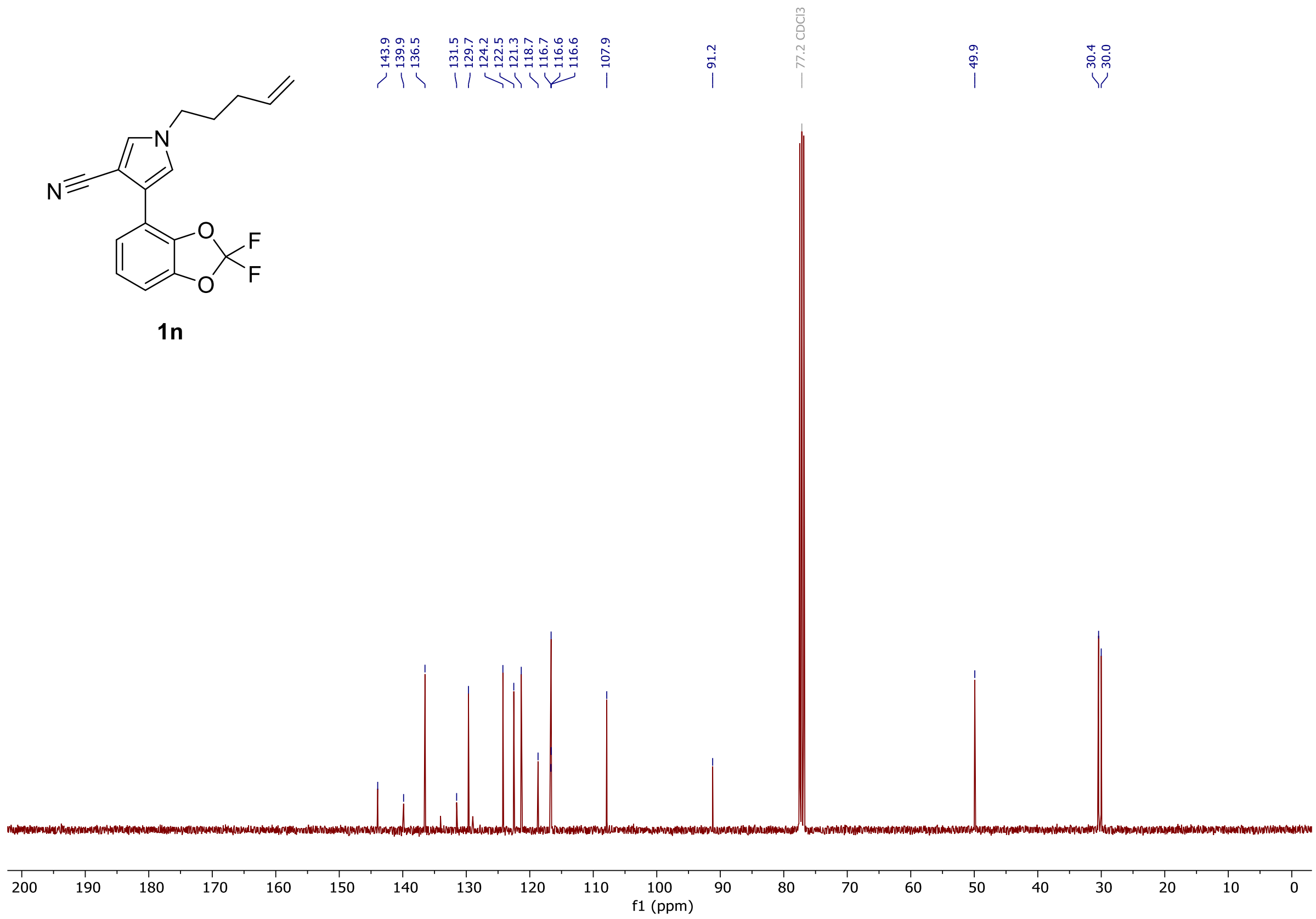

^{19}F NMR (377 MHz, $CDCl_3$) of **1n**

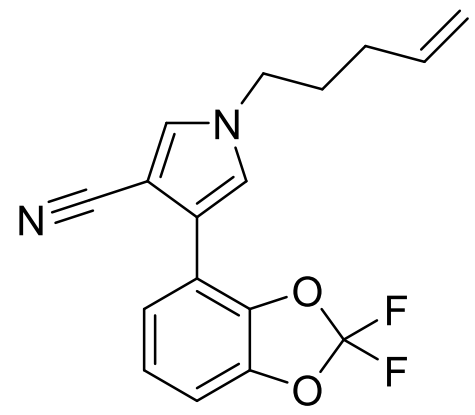

1n

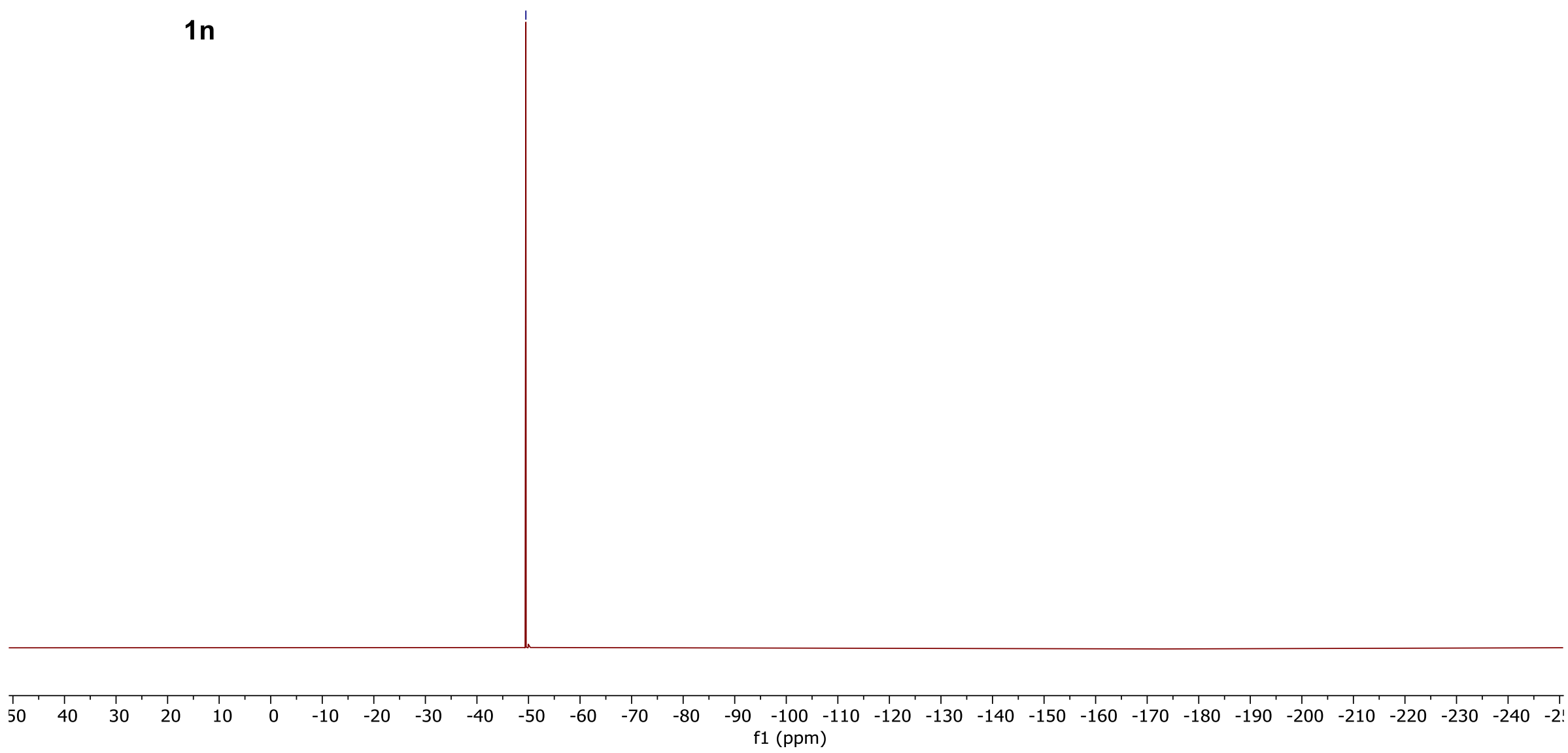

^{1}H NMR (400 MHz, $CDCl_3$) of **1o** (*see procedure*)

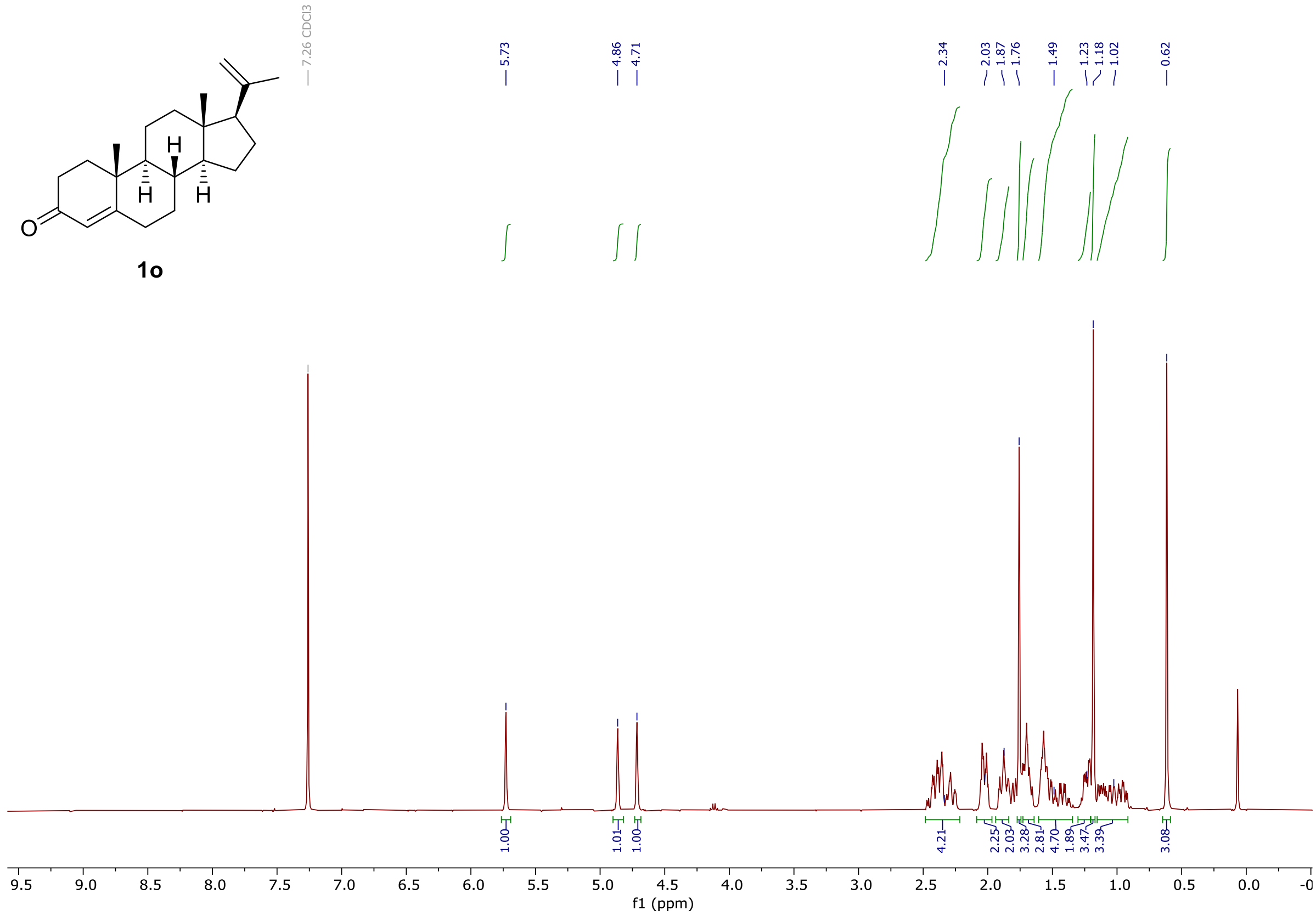

^{13}C NMR (101 MHz, $CDCl_3$) of **1o**

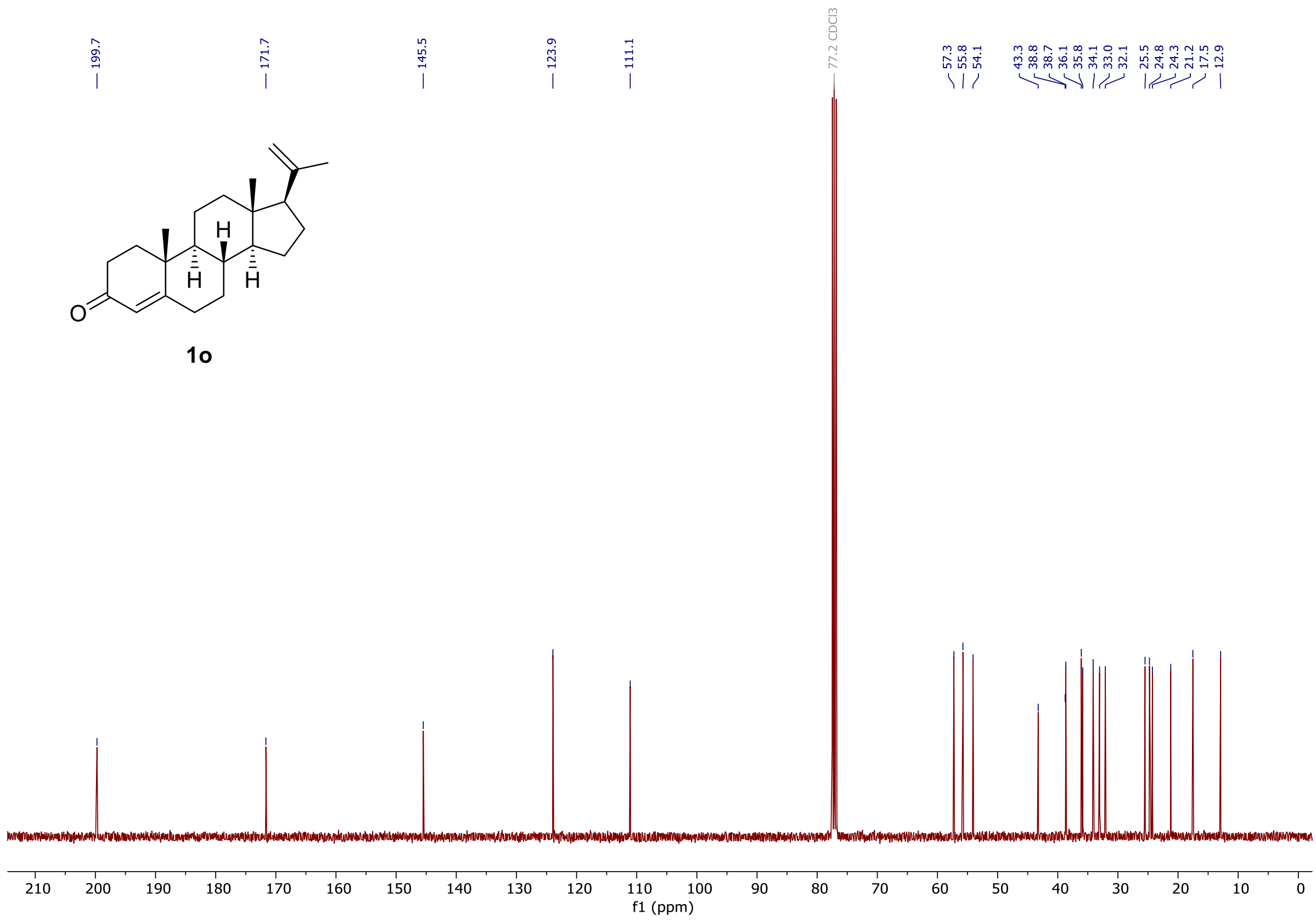

^{1}H NMR (400 MHz, $CDCl_3$) of **1p** (*see procedure*)

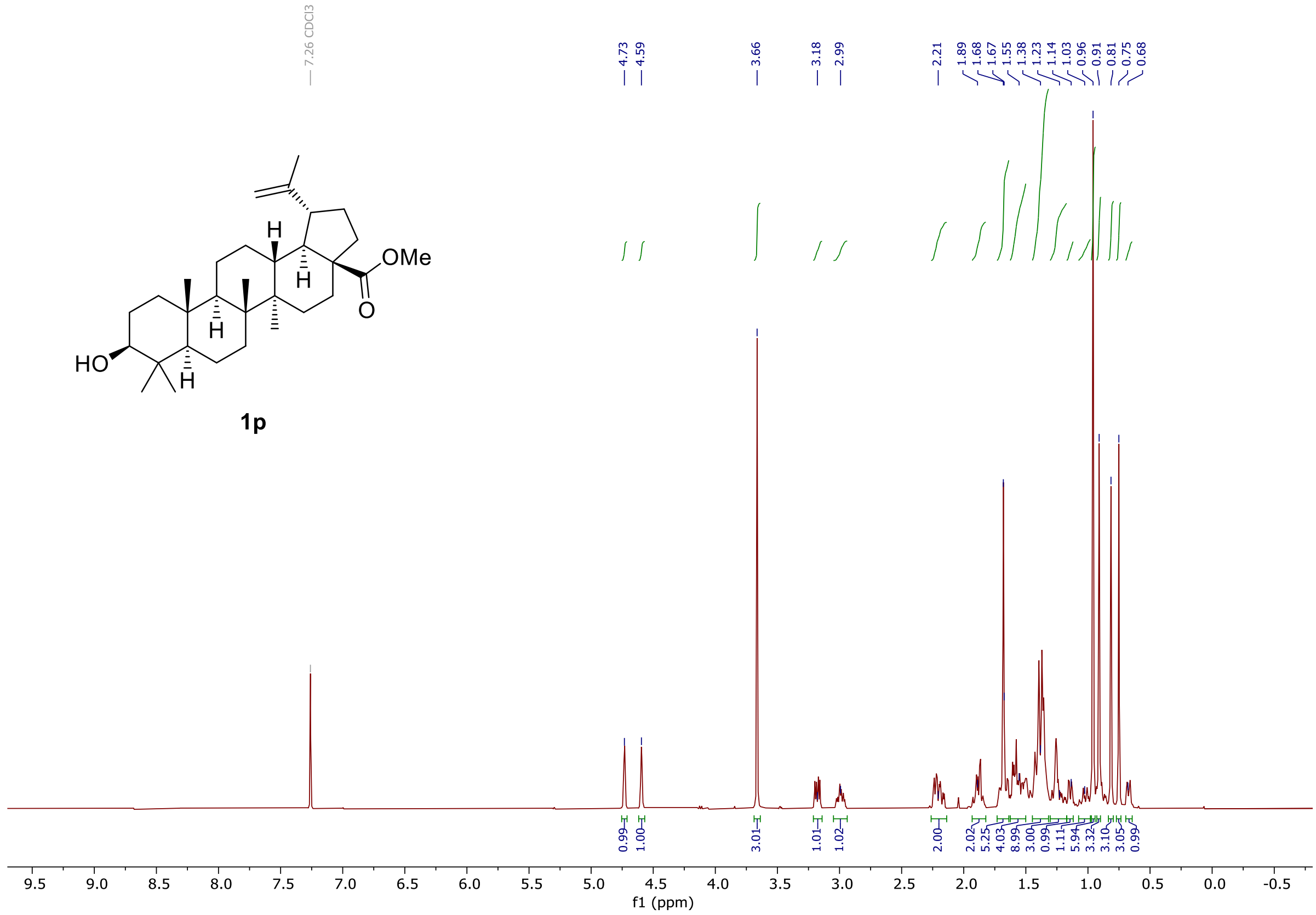

^{13}C NMR (101 MHz, $CDCl_3$) of **1p**

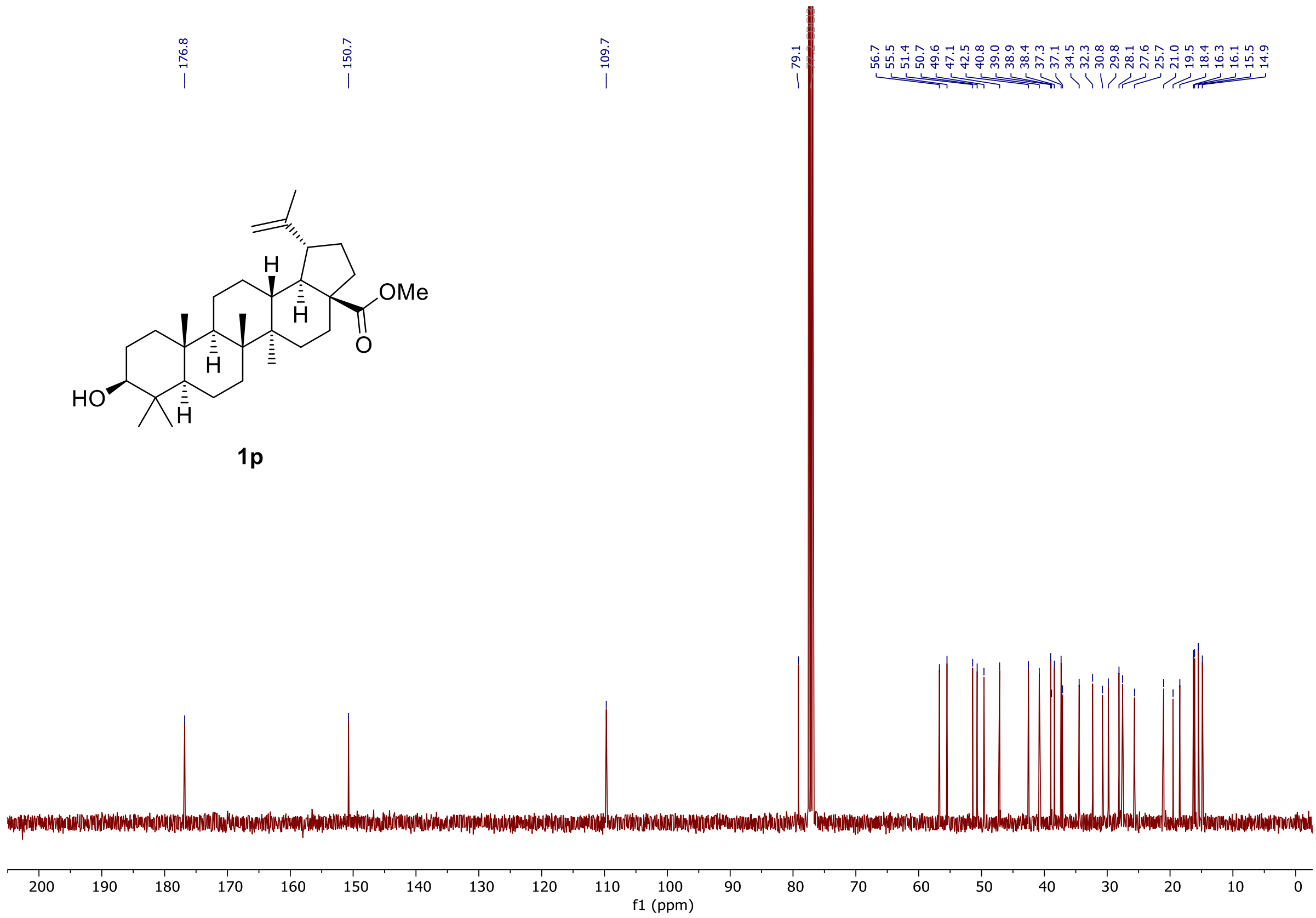

^{1}H NMR (400 MHz, $CDCl_3$) of **1q** (*see procedure*)

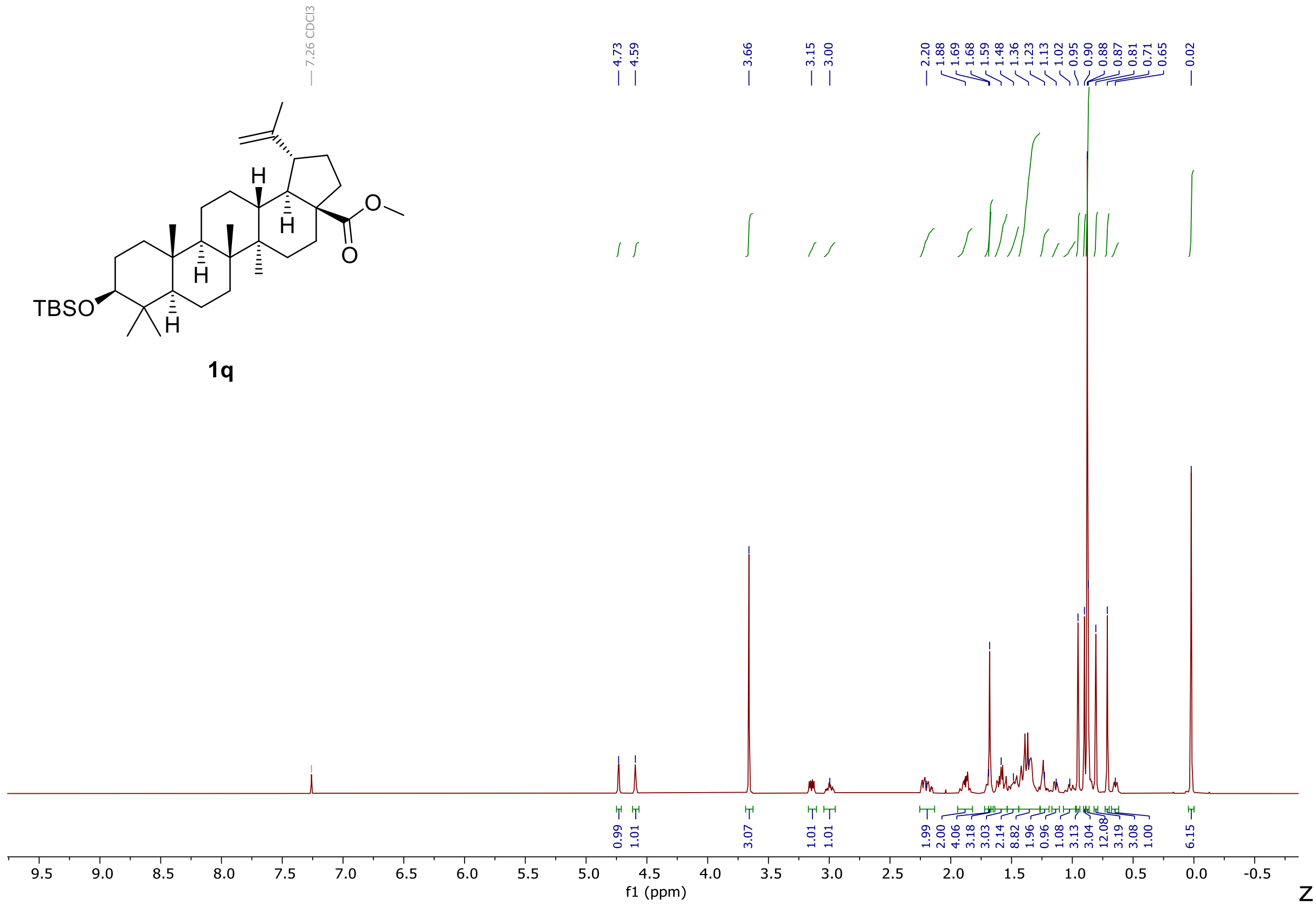

^{13}C NMR (101 MHz, $CDCl_3$) of **1q**

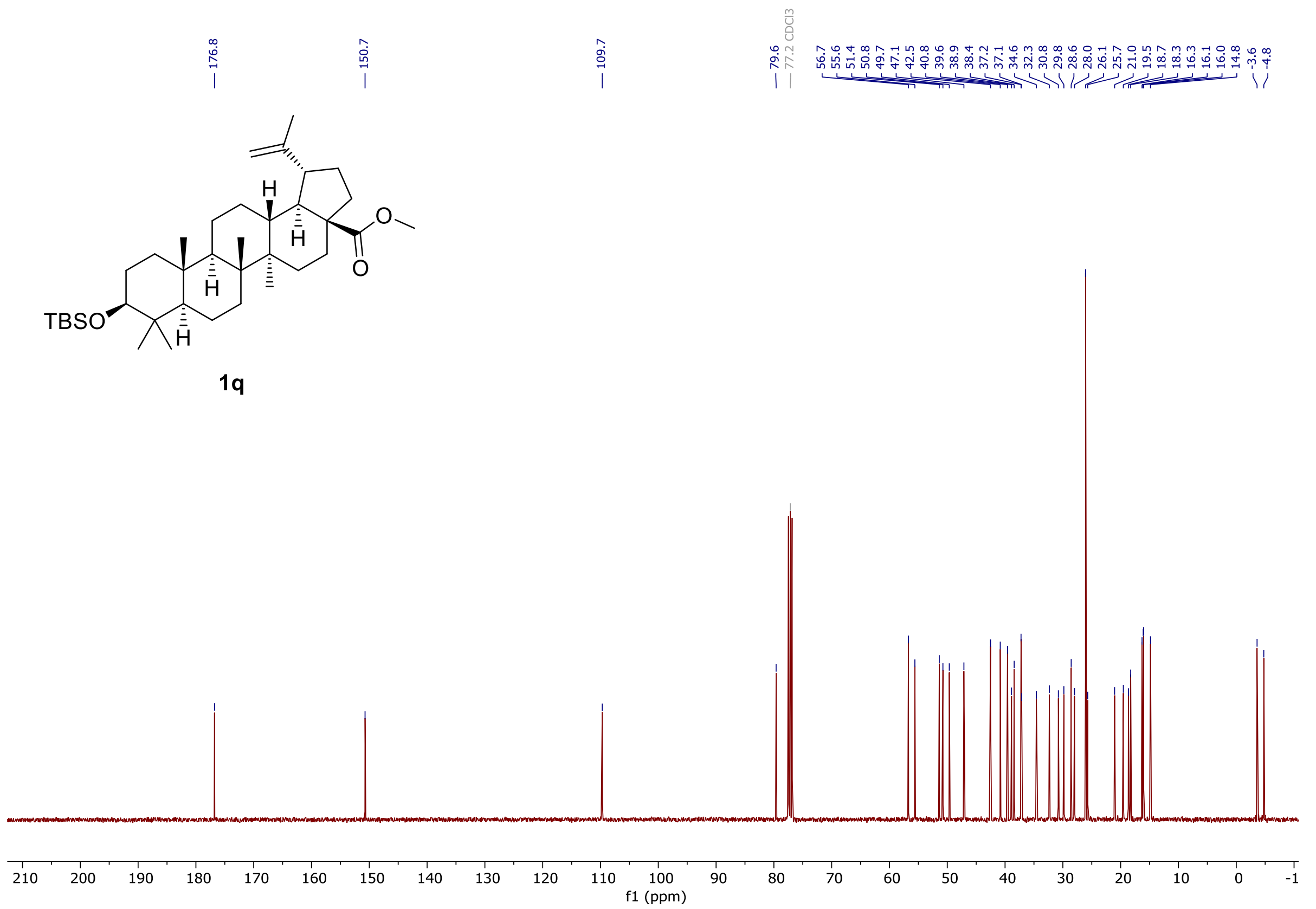

^{1}H NMR (400 MHz, $CDCl_3$) of **SI-4** (*see procedure*)

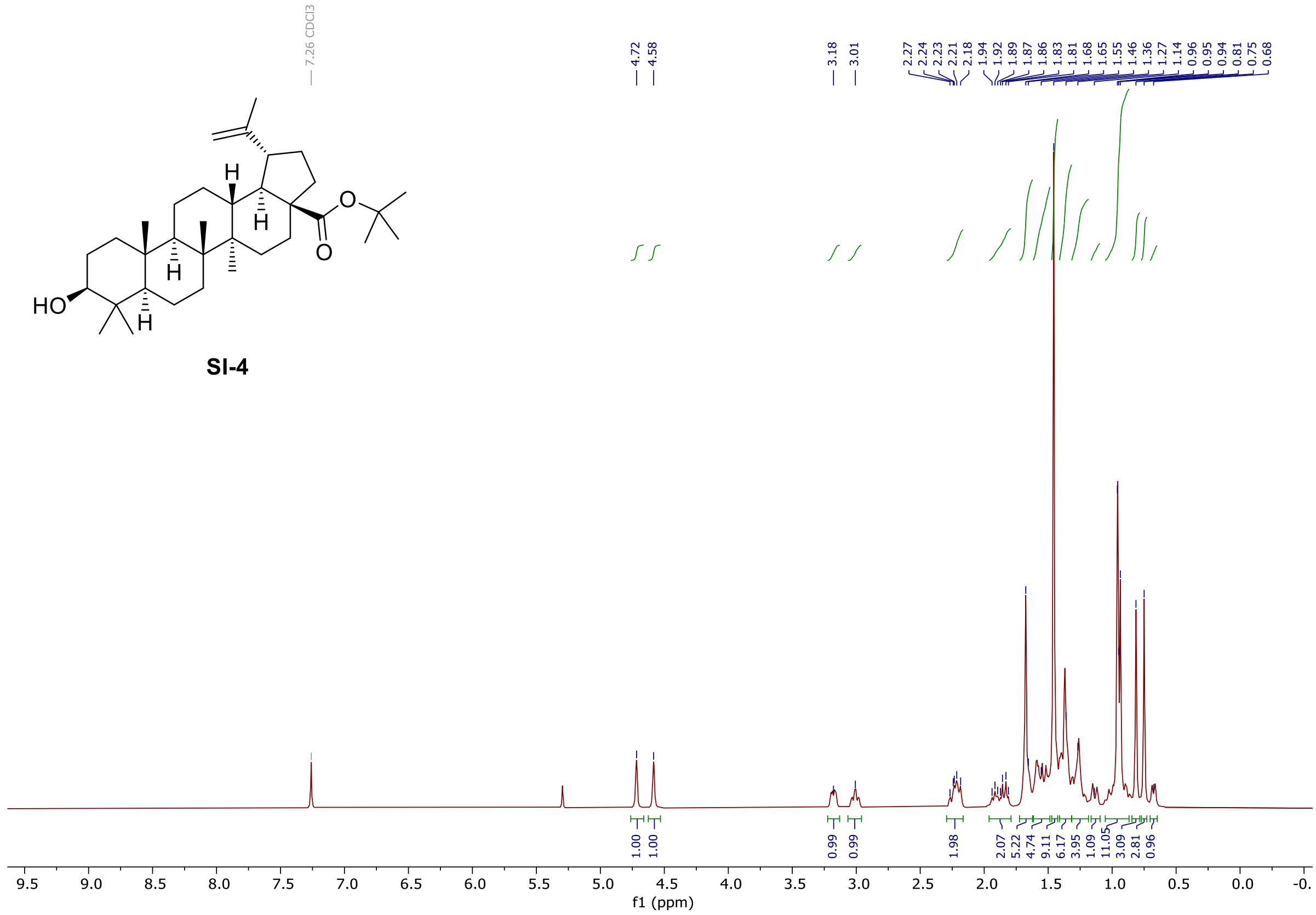

^{13}C NMR (101 MHz, $CDCl_3$) of **SI-4**

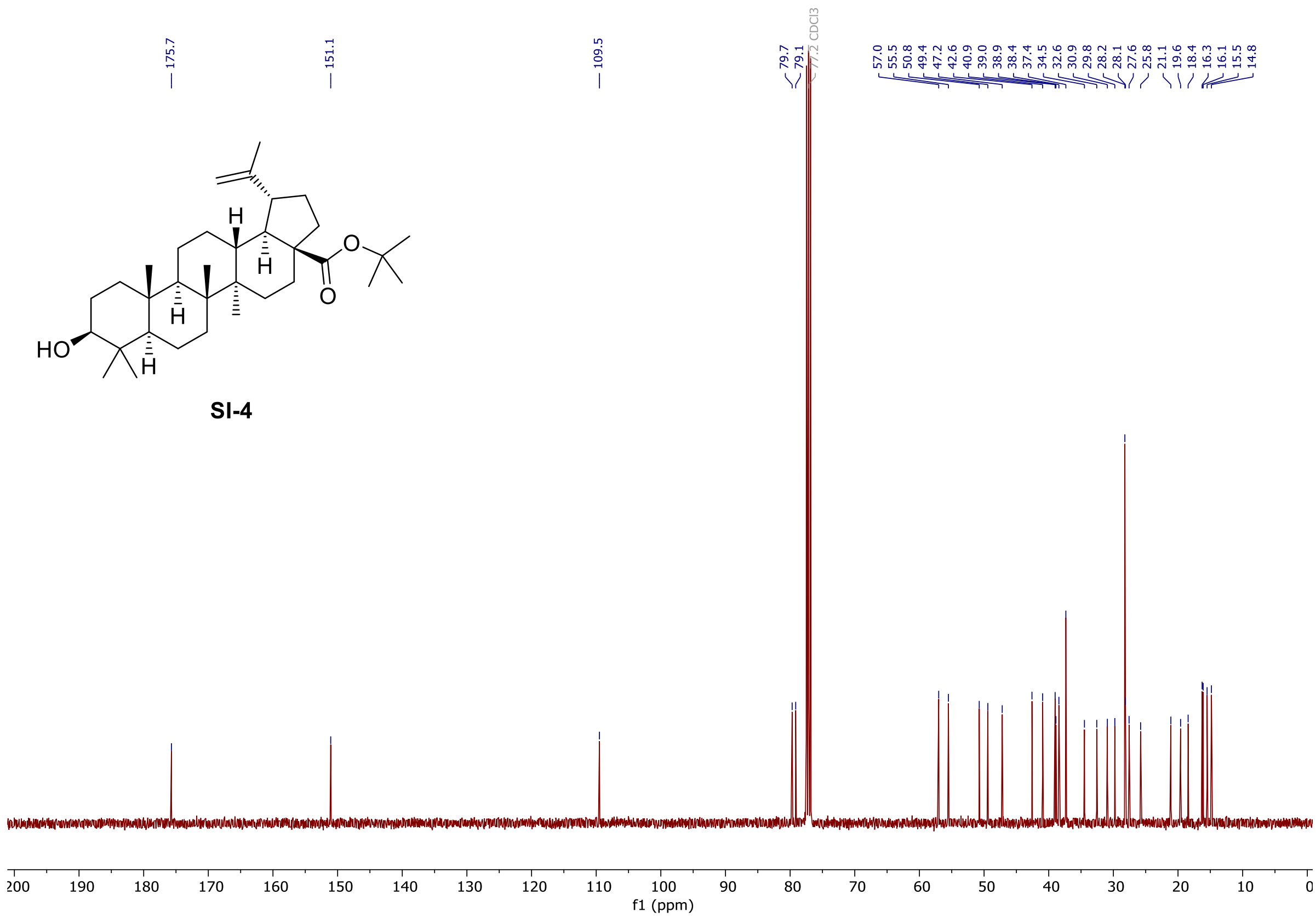

¹H NMR (400 MHz, $CDCl_3$) of **1r** (see procedure)

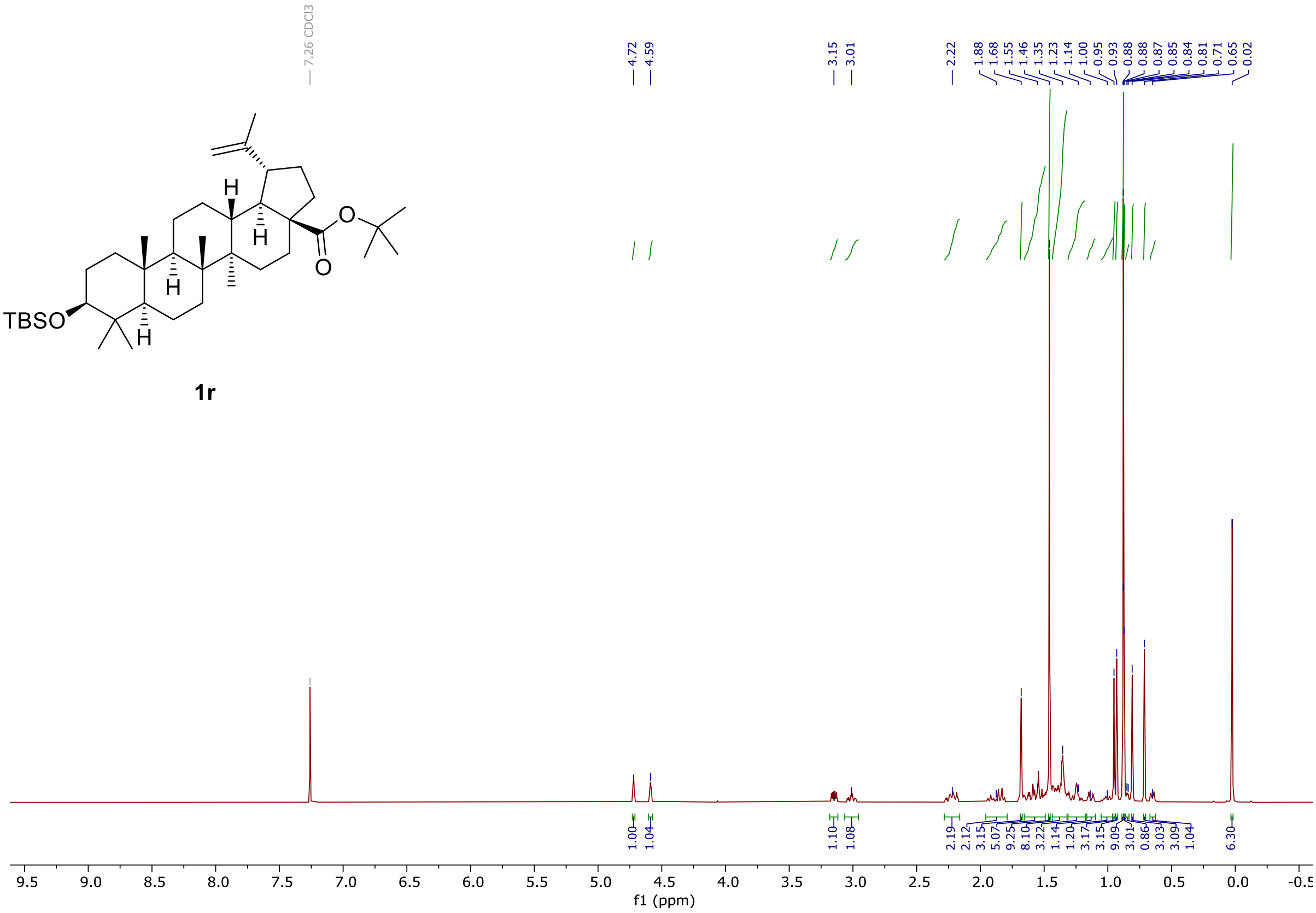

1r

¹³C NMR (101 MHz, $CDCl_3$) of **1r**

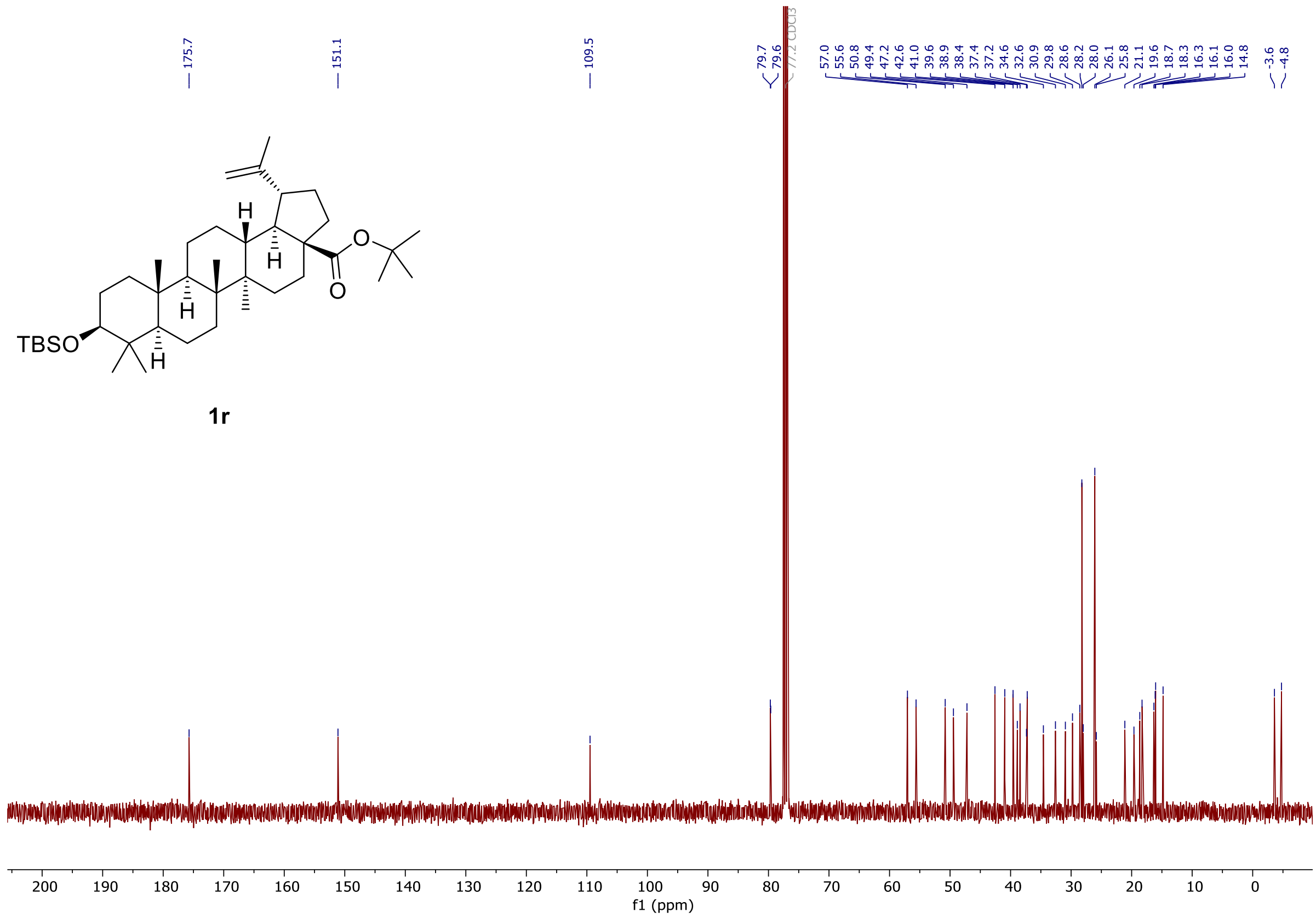

1r

^{1}H NMR (400 MHz, $CDCl_3$) of **3b** (*see procedure*)

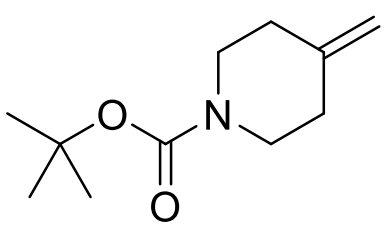

3b

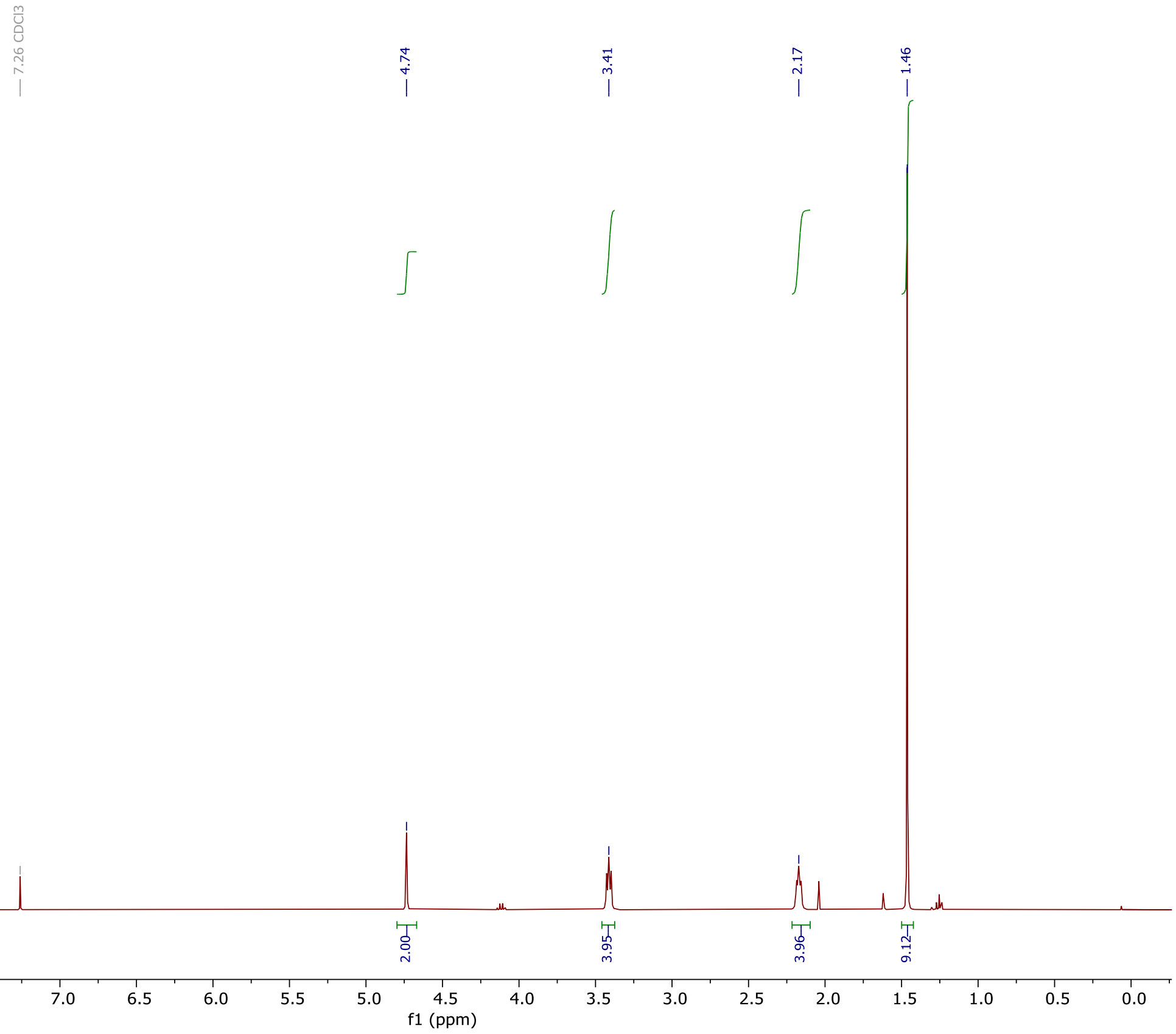

^{13}C NMR (101 MHz, $CDCl_3$) of **3b**

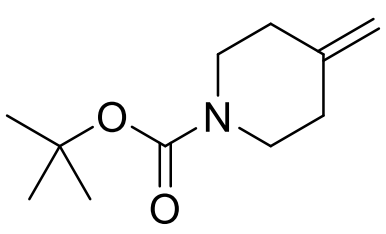

3b

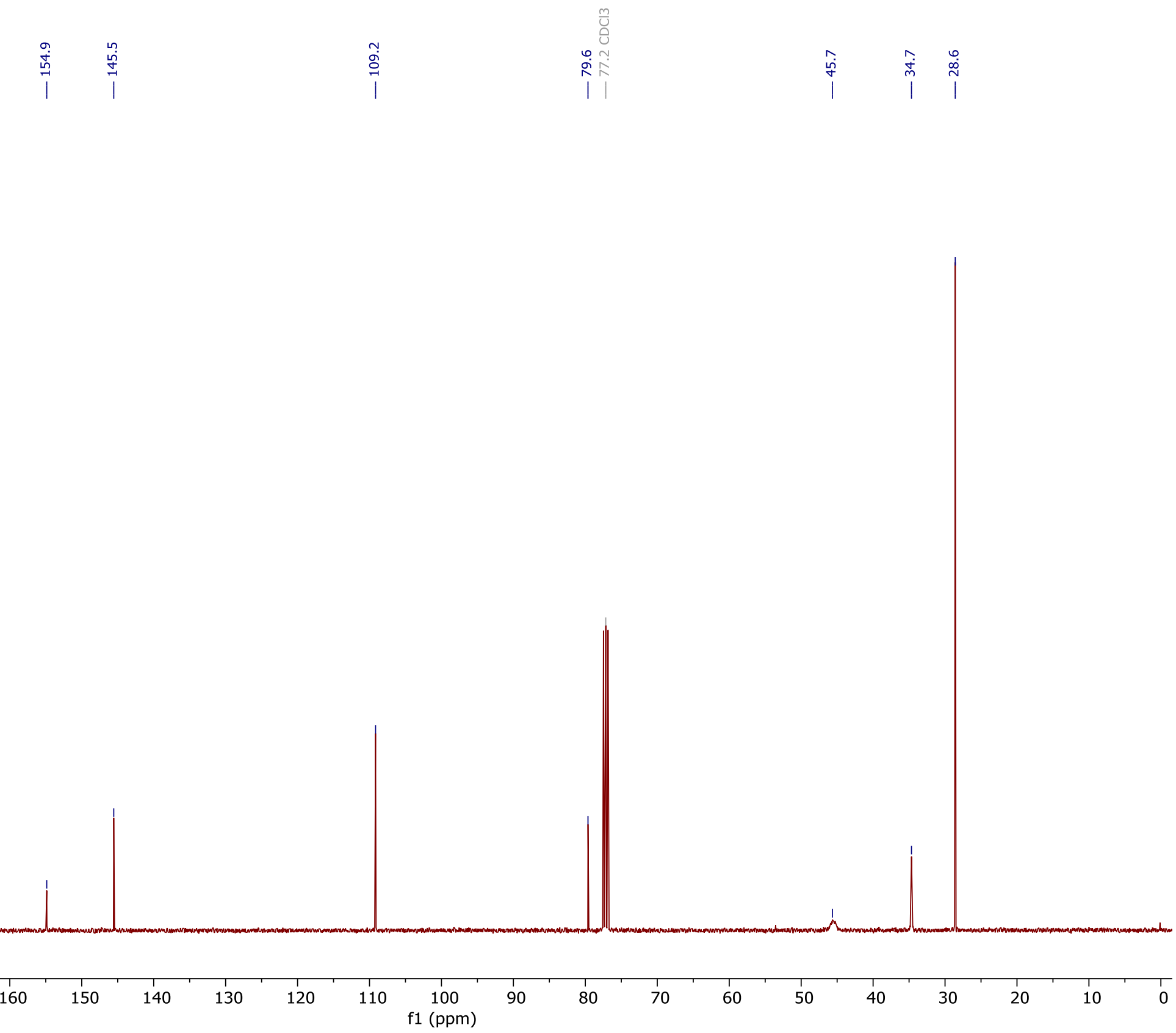

^{1}H NMR (400 MHz, $CDCl_3$) of **3d** (<u>*see procedure*</u>)

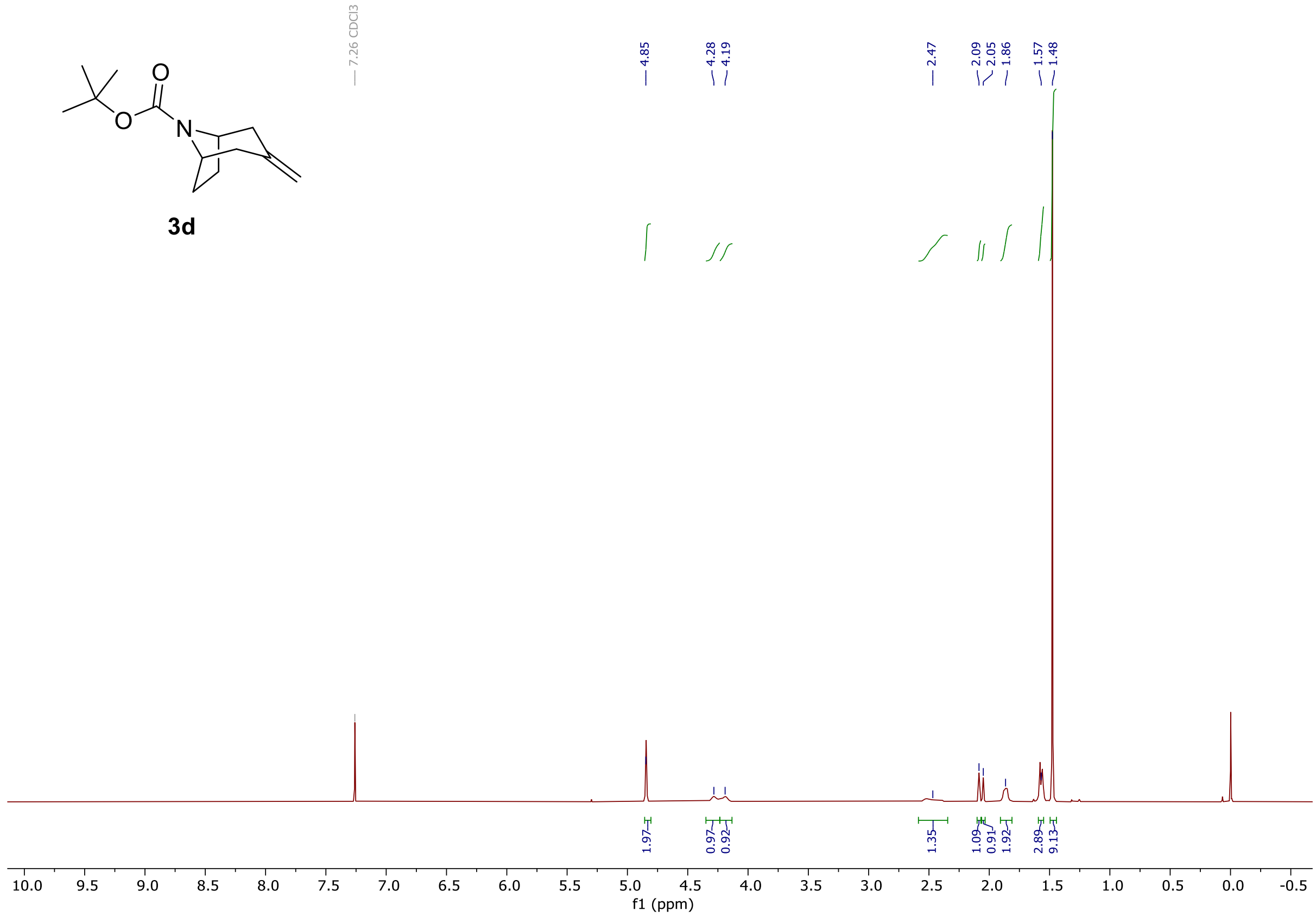

^{13}C NMR (101 MHz, $CDCl_3$) of **3d**

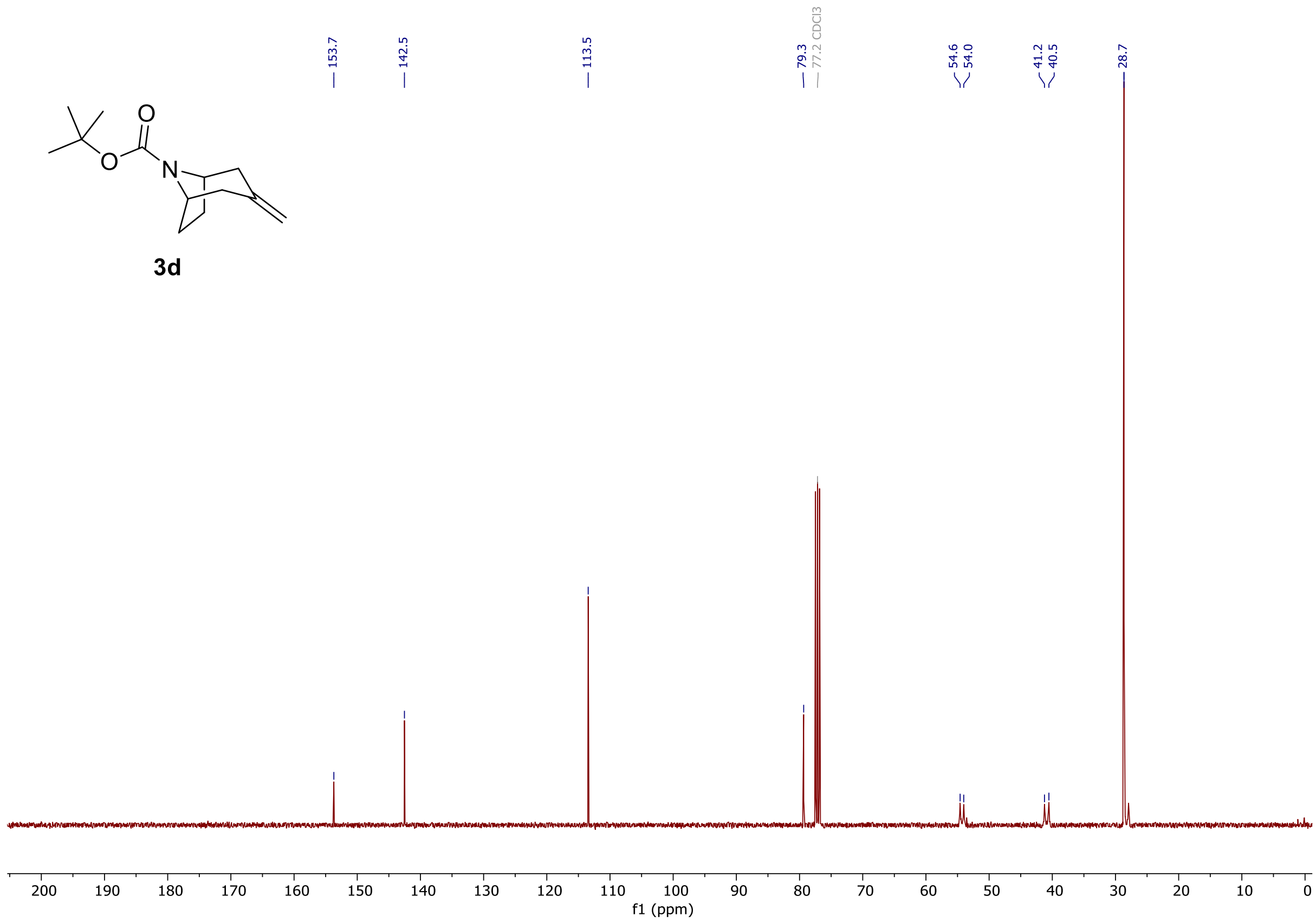

^{1}H NMR (400 MHz, $CDCl_3$) of **2a** (*see procedure*)

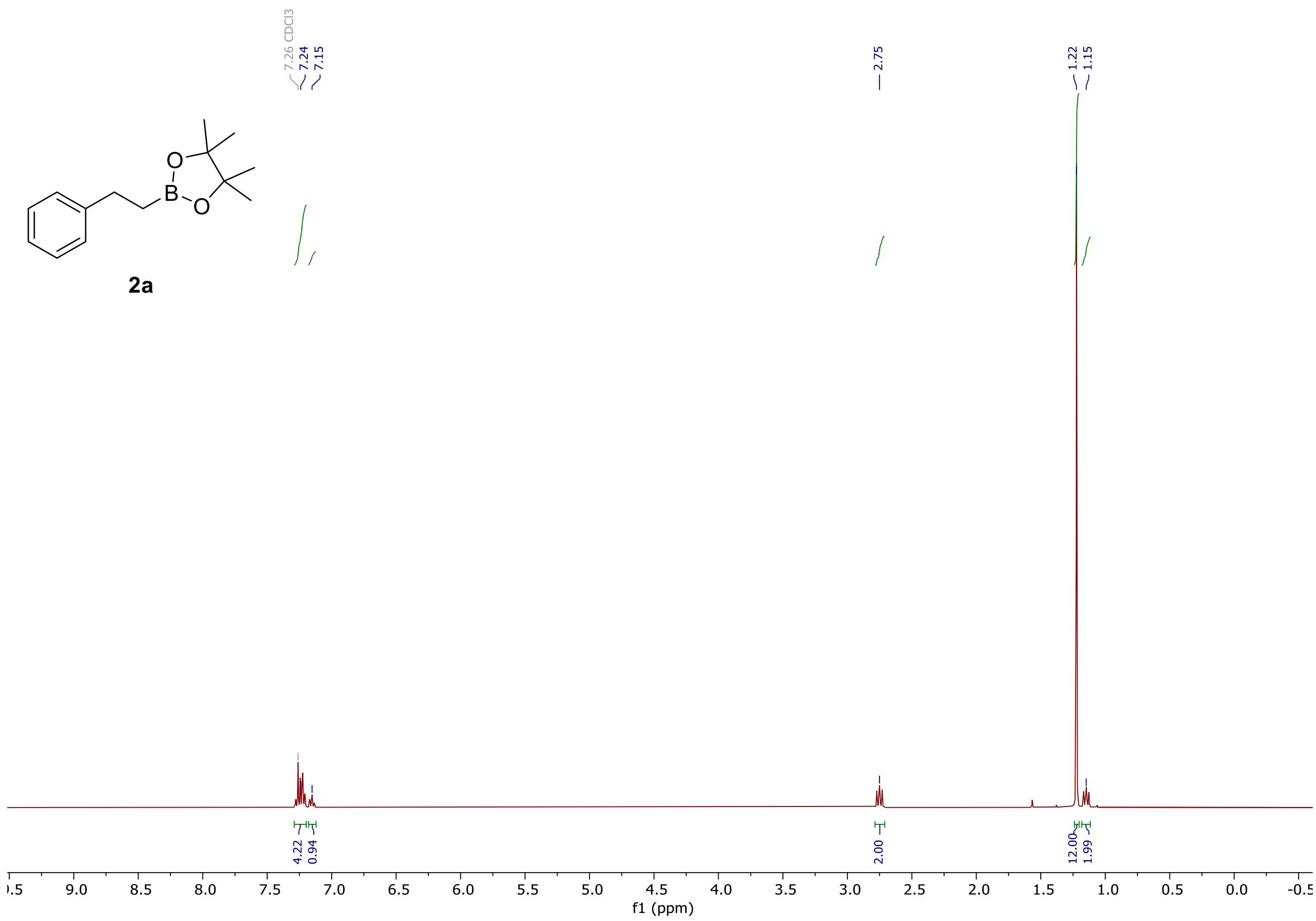

^{13}C NMR (101 MHz, $CDCl_3$) of **2a**

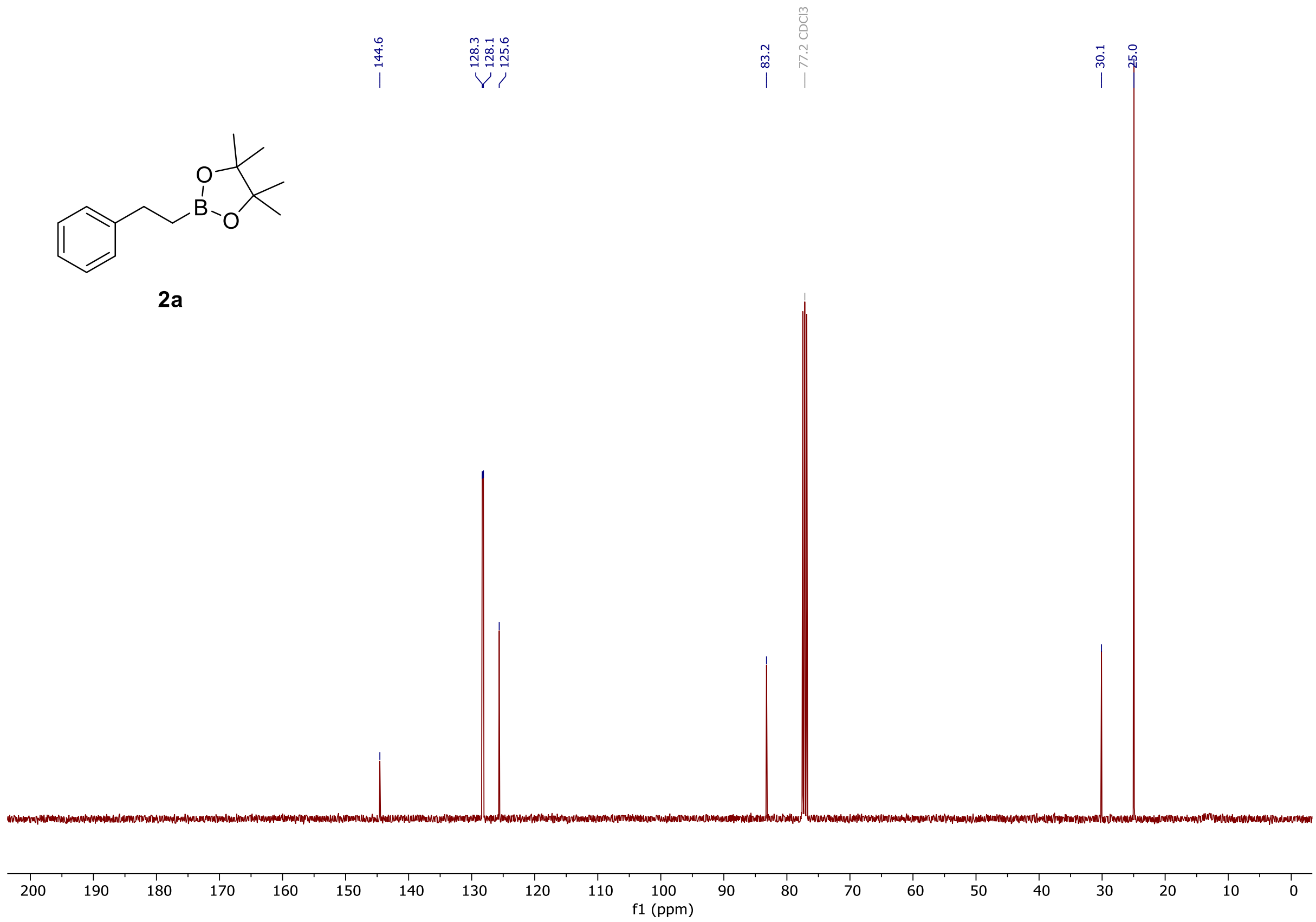

^{1}H NMR (400 MHz, $CDCl_3$) of **2c** (*see procedure*)

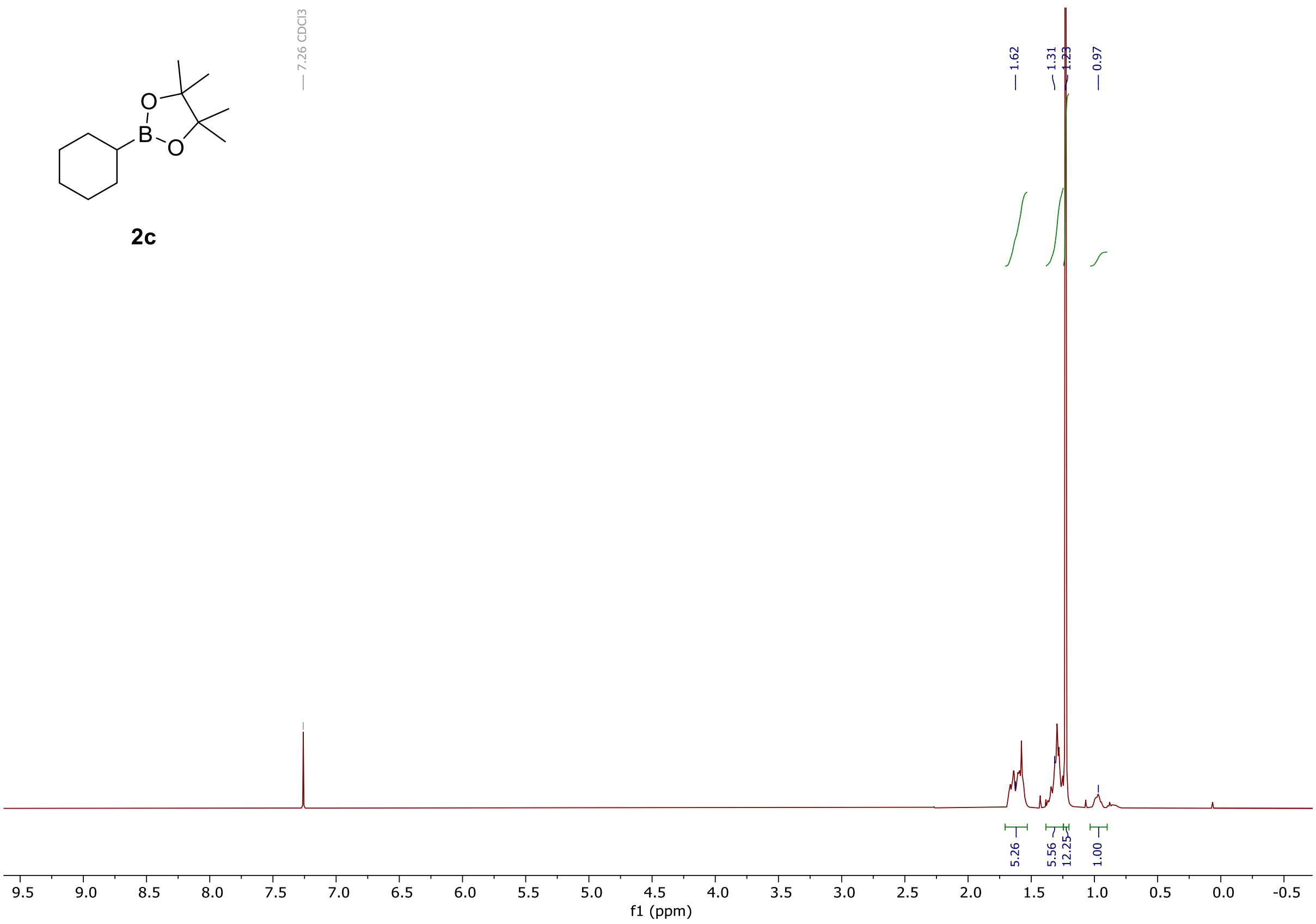

^{13}C NMR (101 MHz, $CDCl_3$) of **2c**

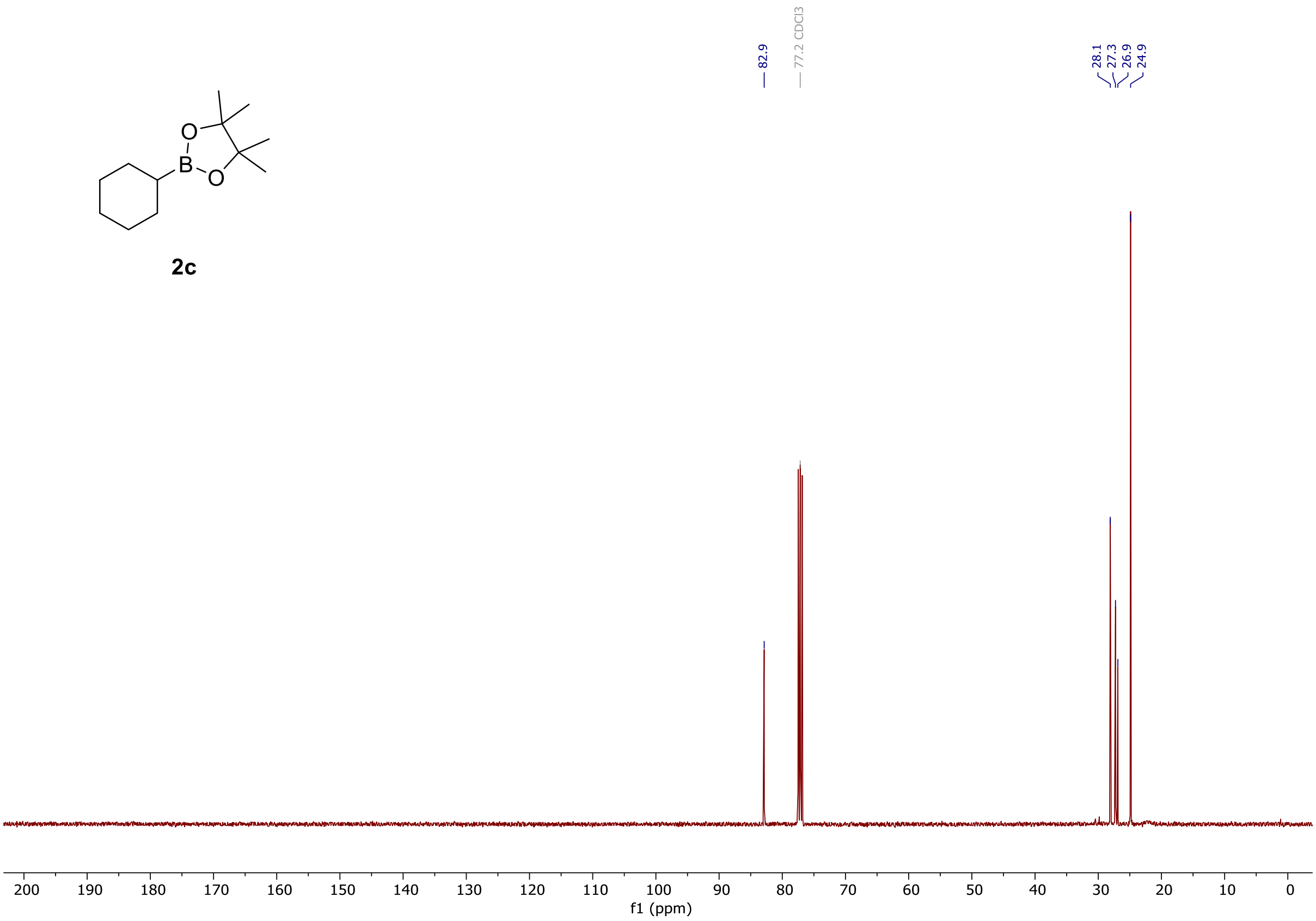

^{1}H NMR (400 MHz, $CDCl_3$) of **2e** (*see procedure*)

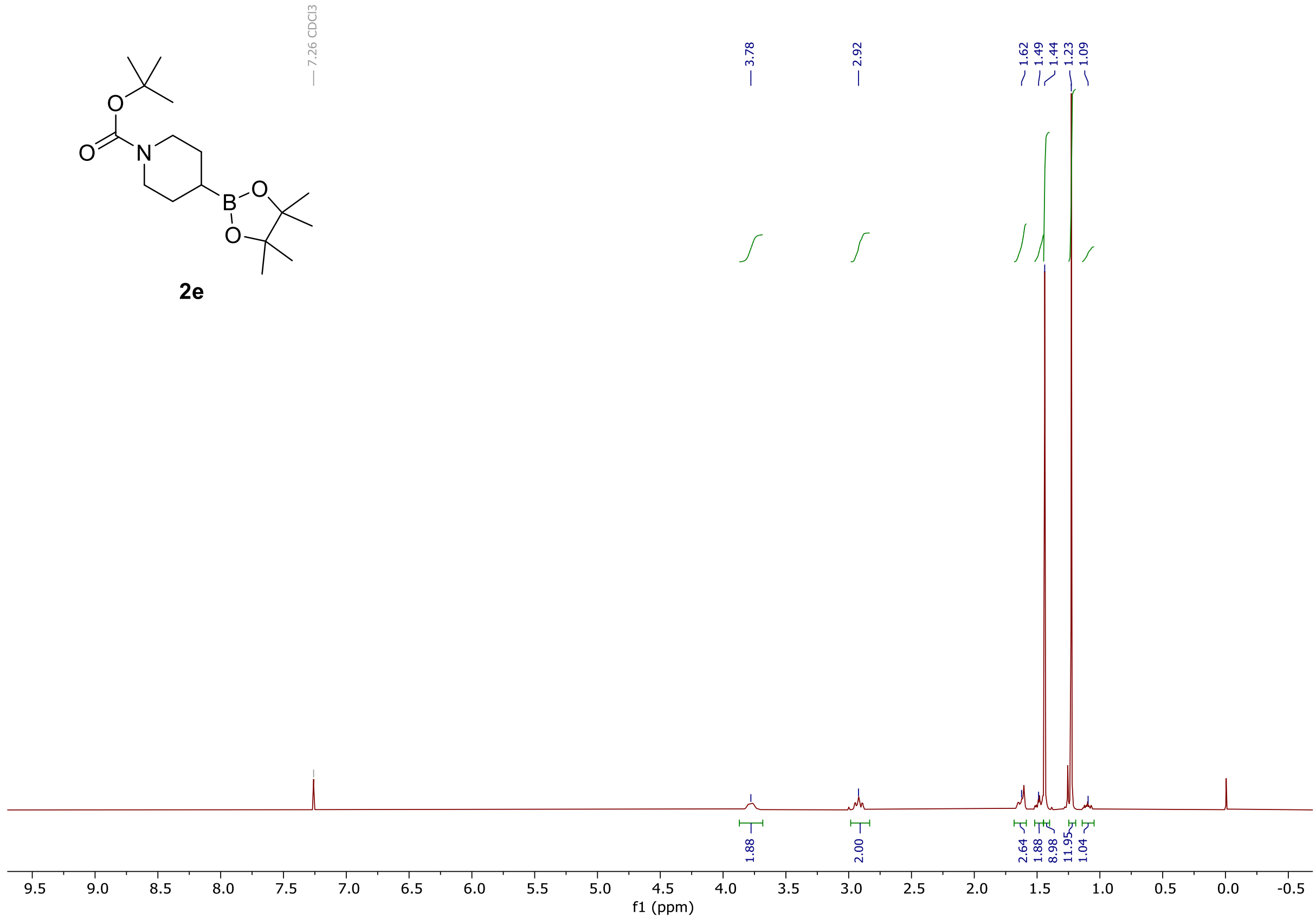

^{13}C NMR (101 MHz, $CDCl_3$) of **2e**

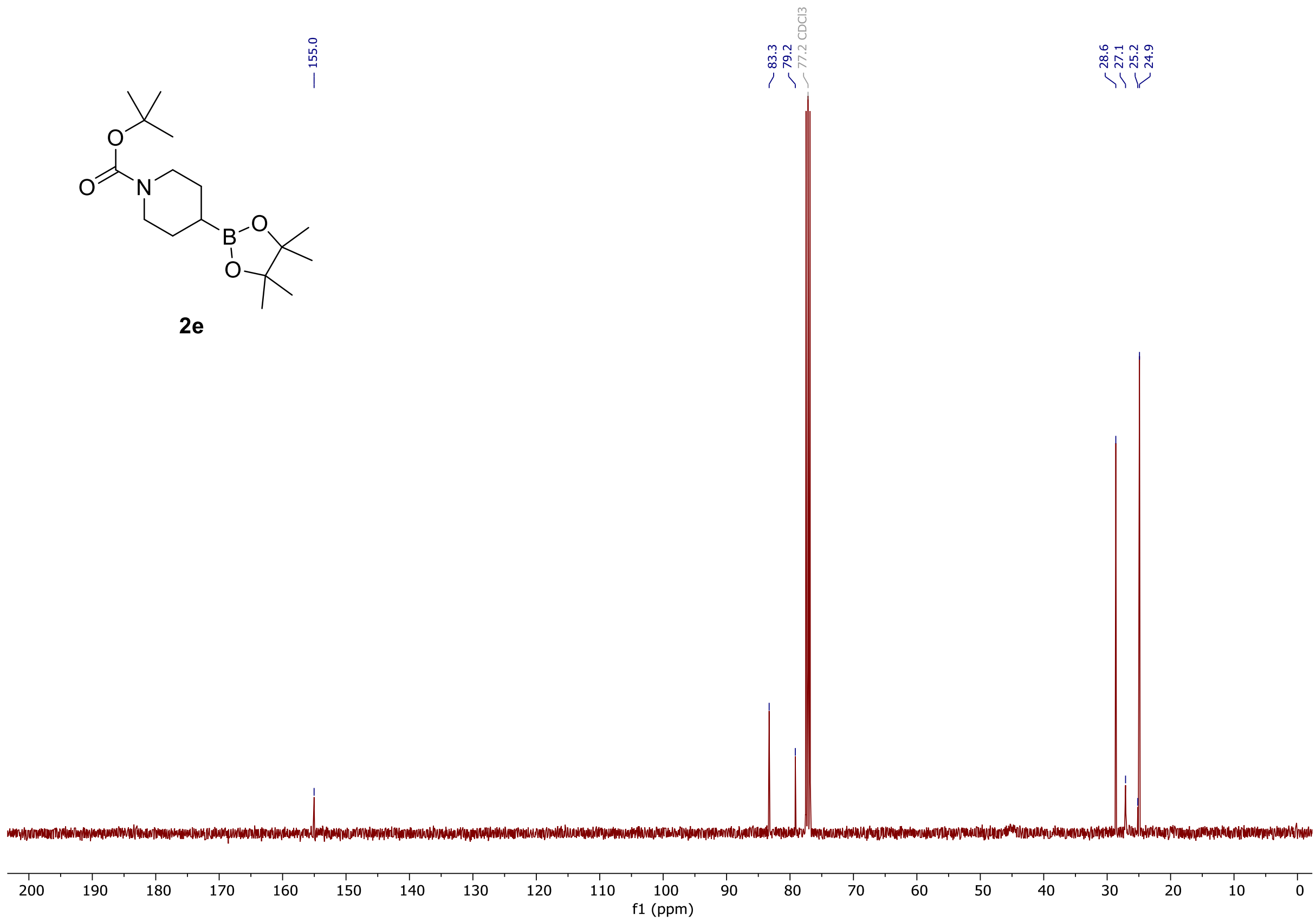

^{1}H NMR (400 MHz, $CDCl_3$) of **2f** (<u>*see procedure*</u>)

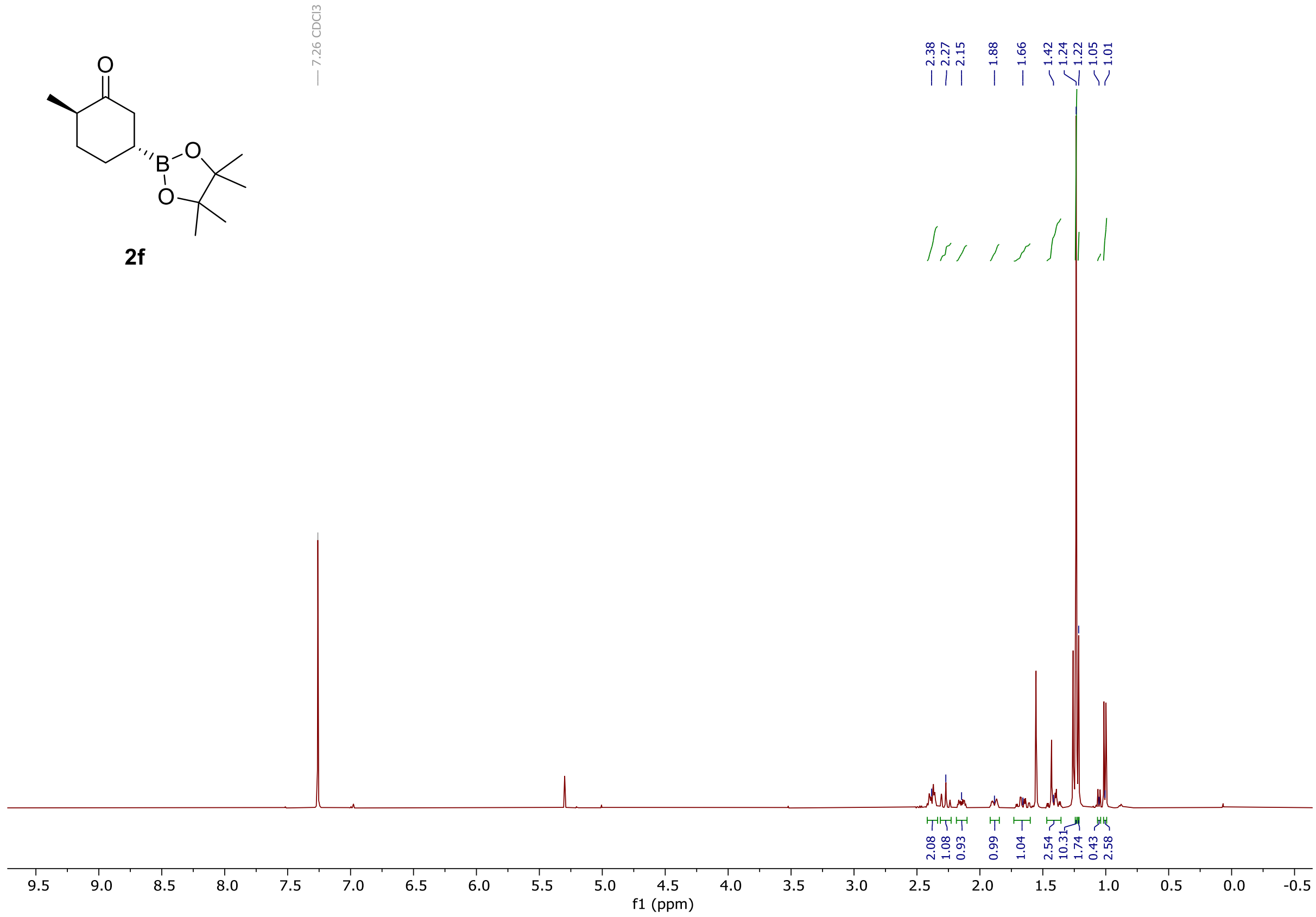

^{13}C NMR (101 MHz, $CDCl_3$) of **2f**

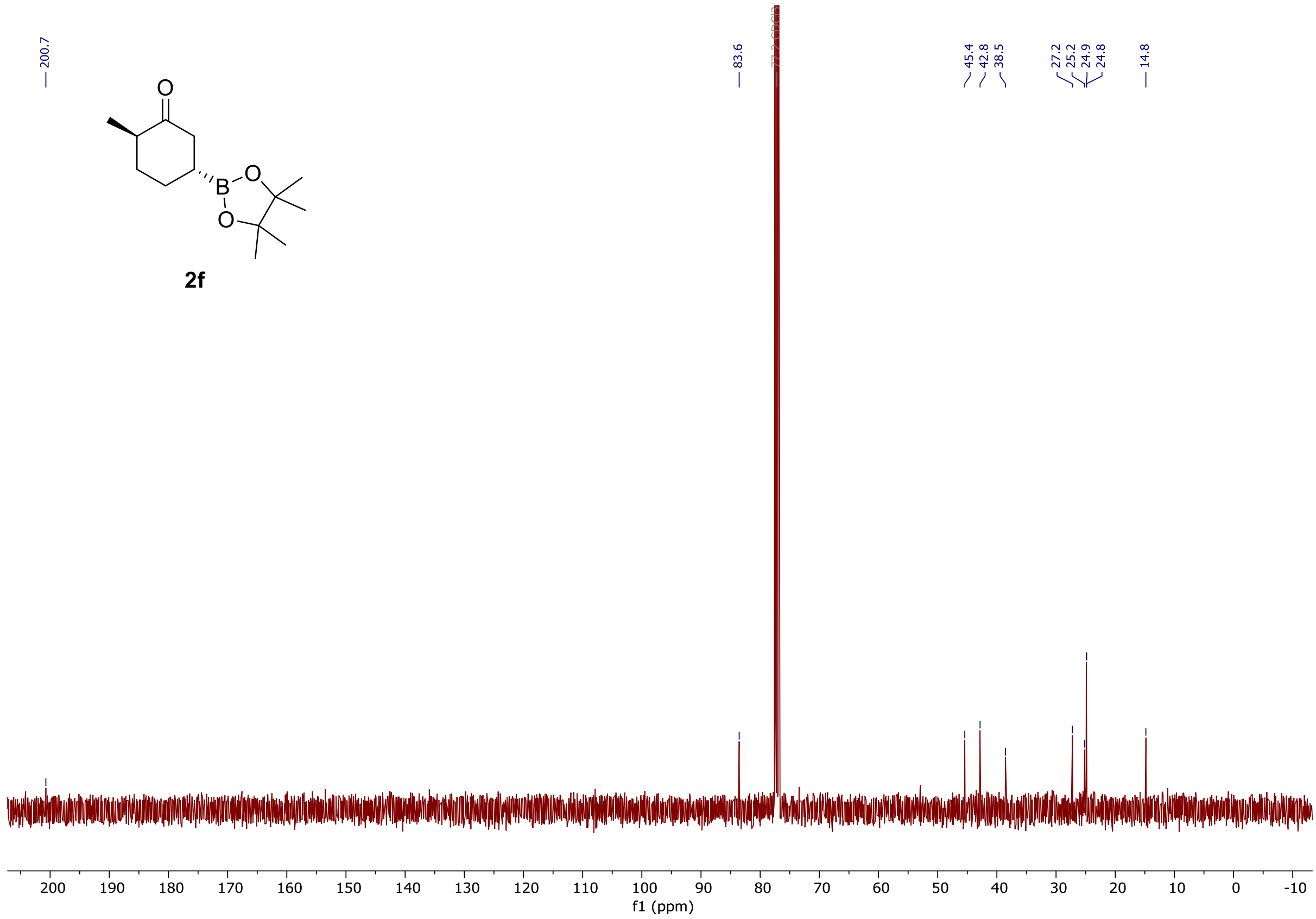

^{1}H NMR (400 MHz, $CDCl_3$) of **2g** (*see procedure*)

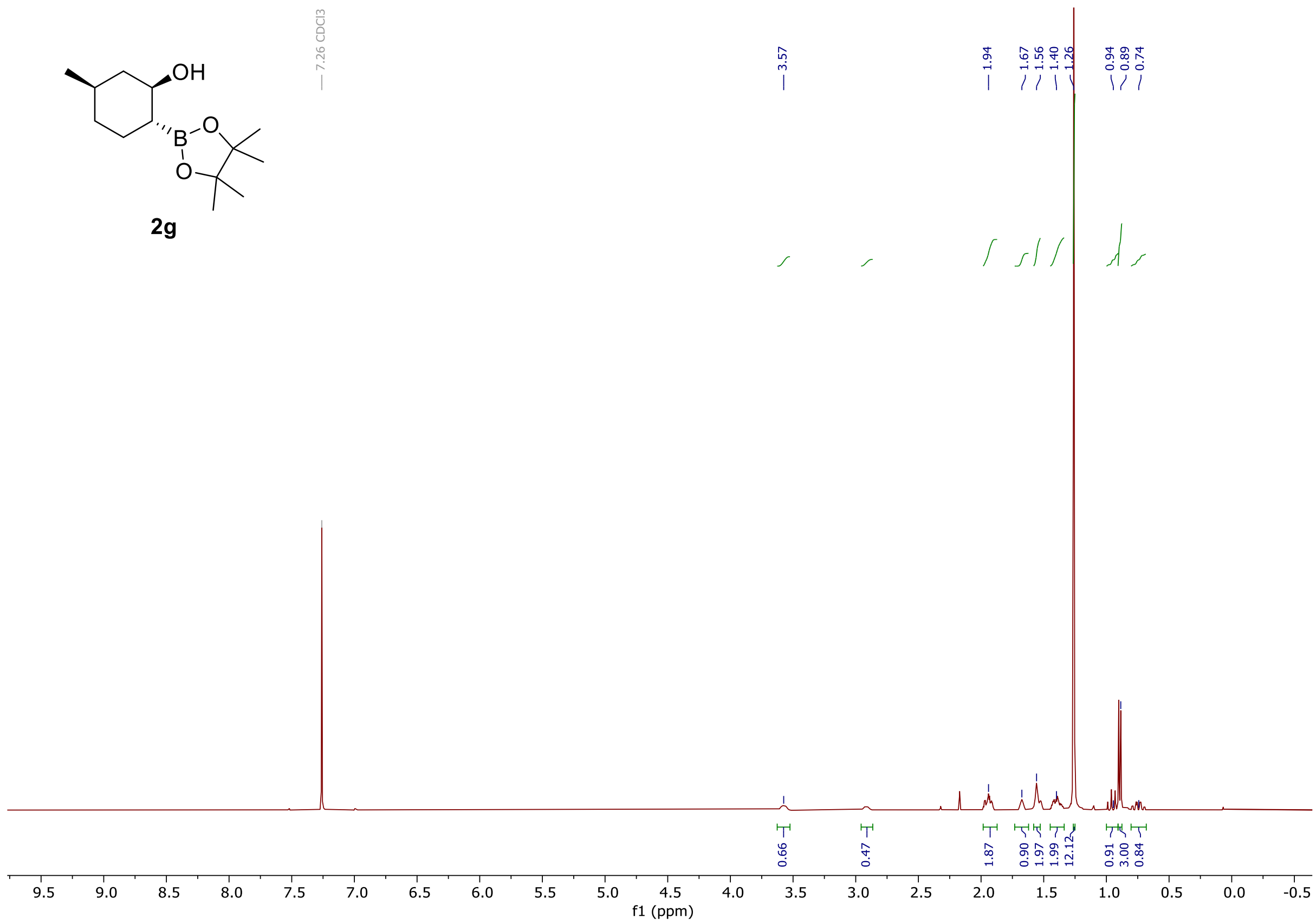

^{13}C NMR (101 MHz, $CDCl_3$) of **2g**

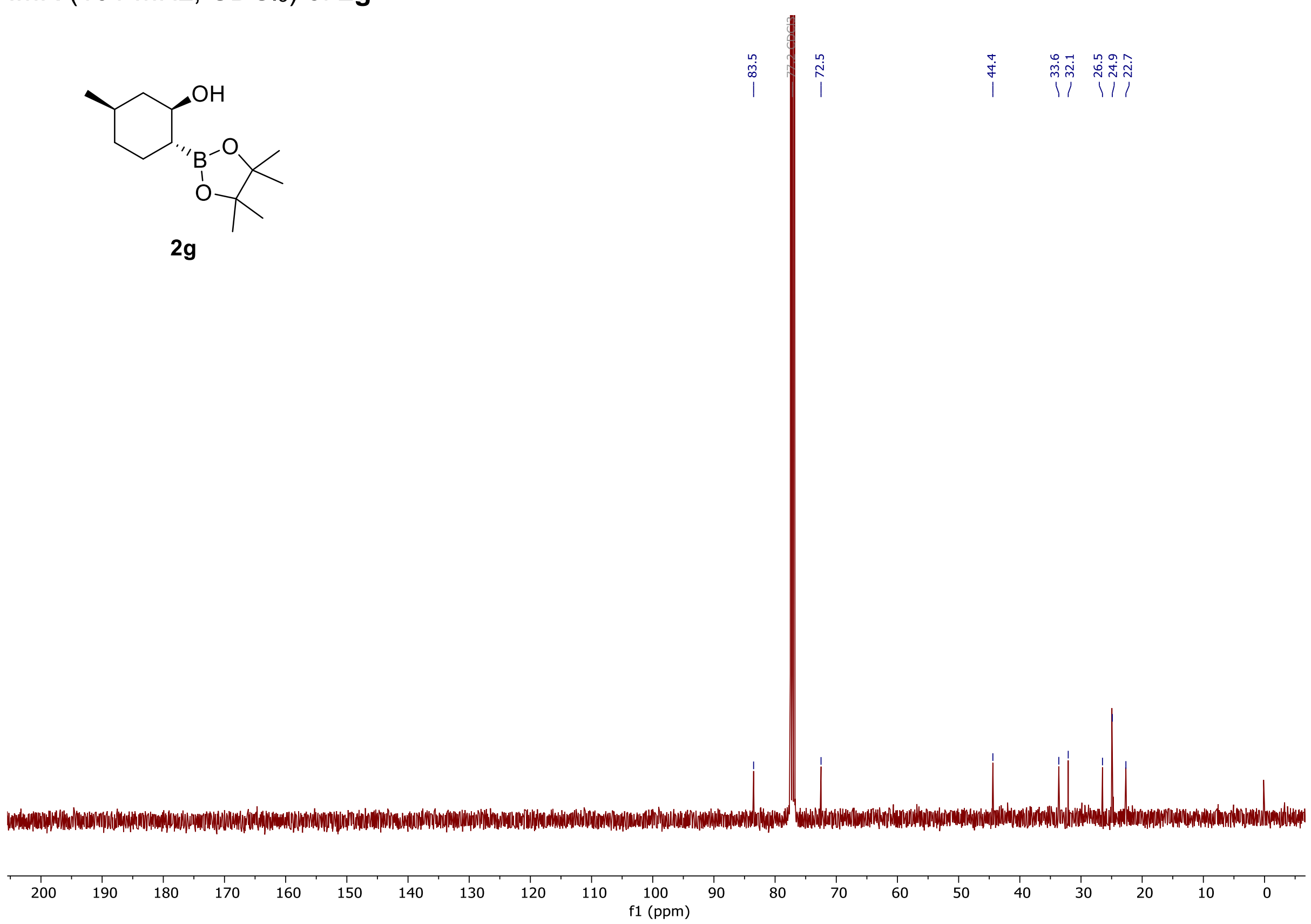

^{1}H NMR (400 MHz, $CDCl_3$) of **2h** (*see procedure*)

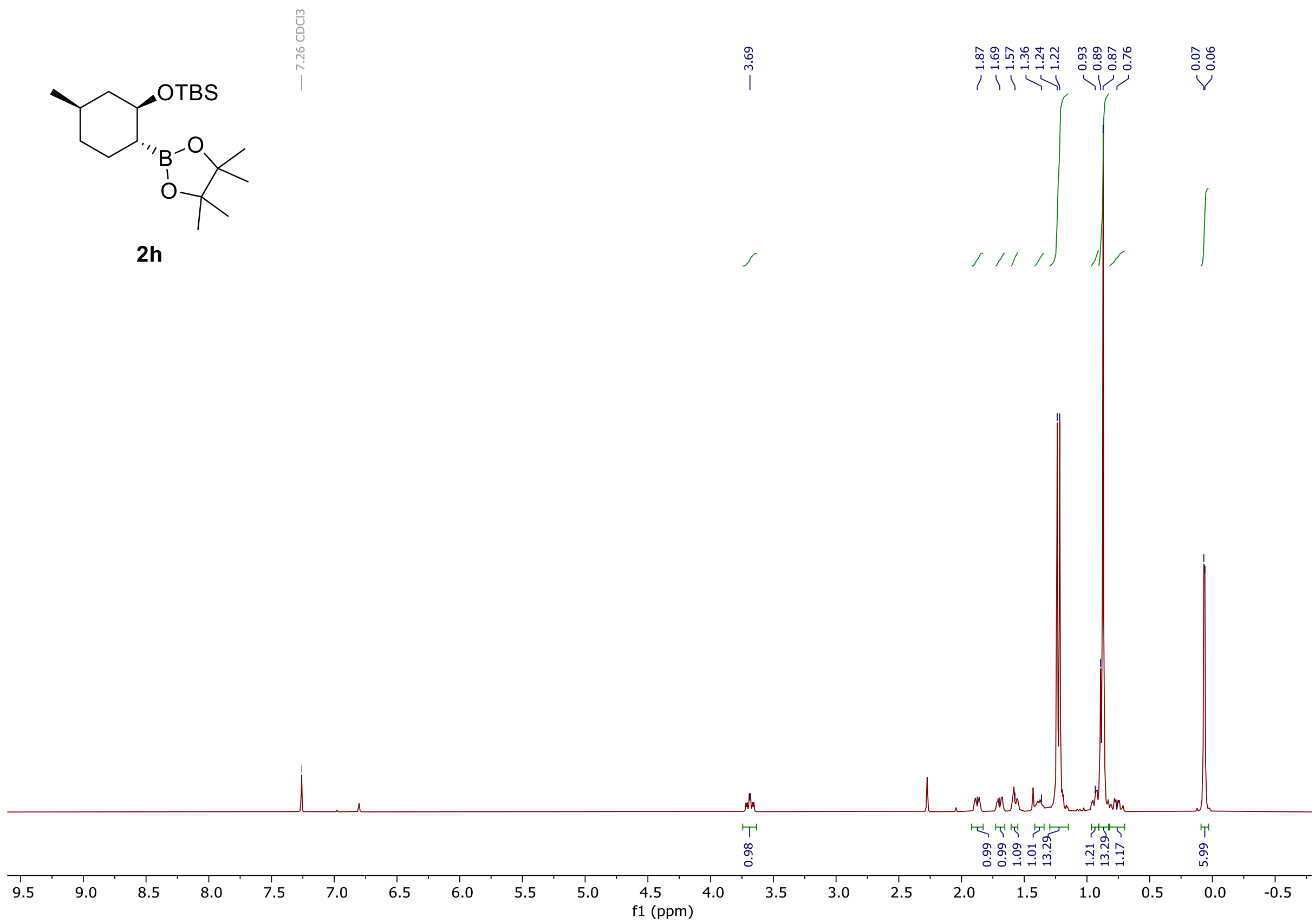

^{13}C NMR (101 MHz, $CDCl_3$) of **2h**

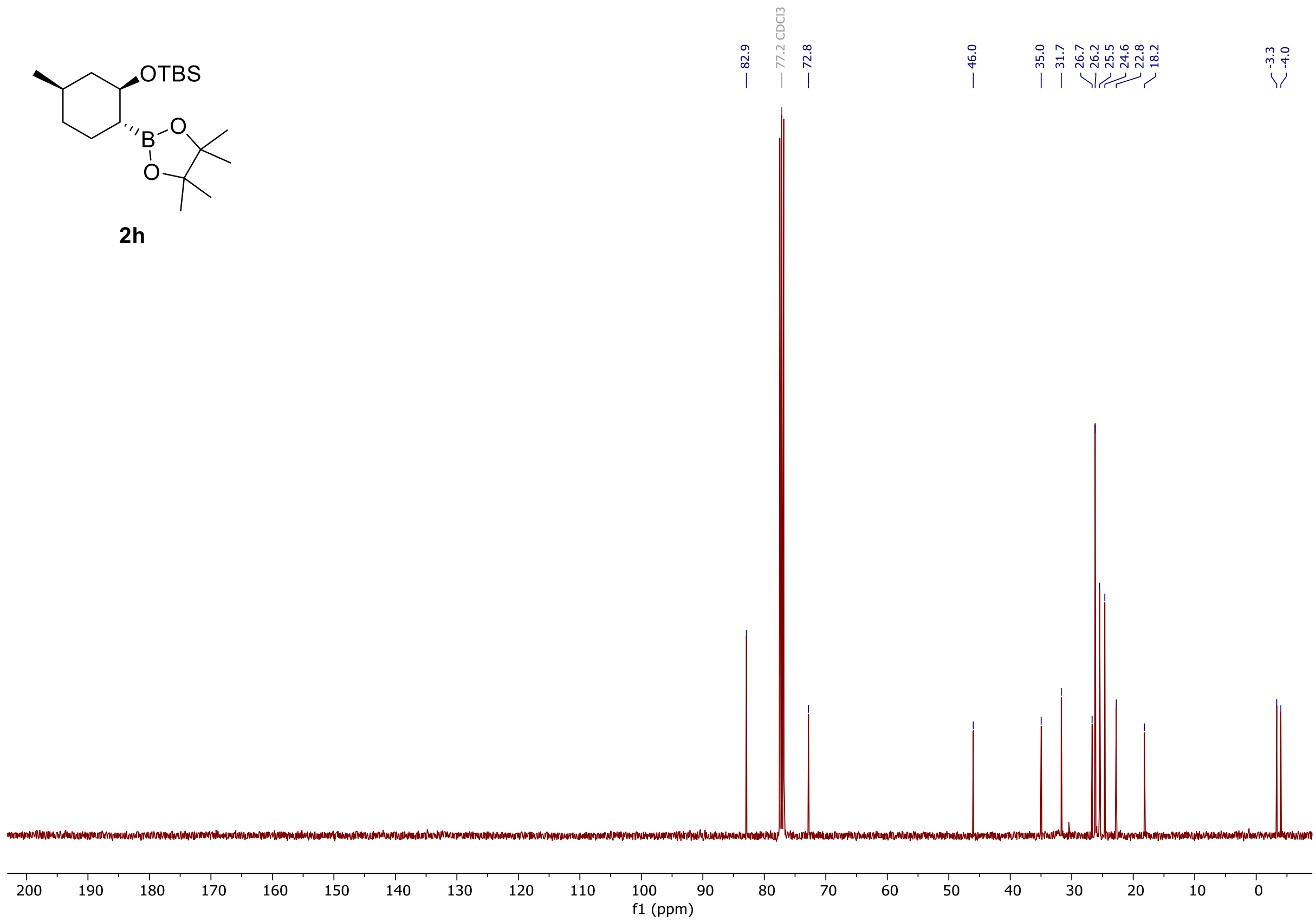

^{1}H NMR (400 MHz, $CDCl_3$) of **2i** (*see procedure*)

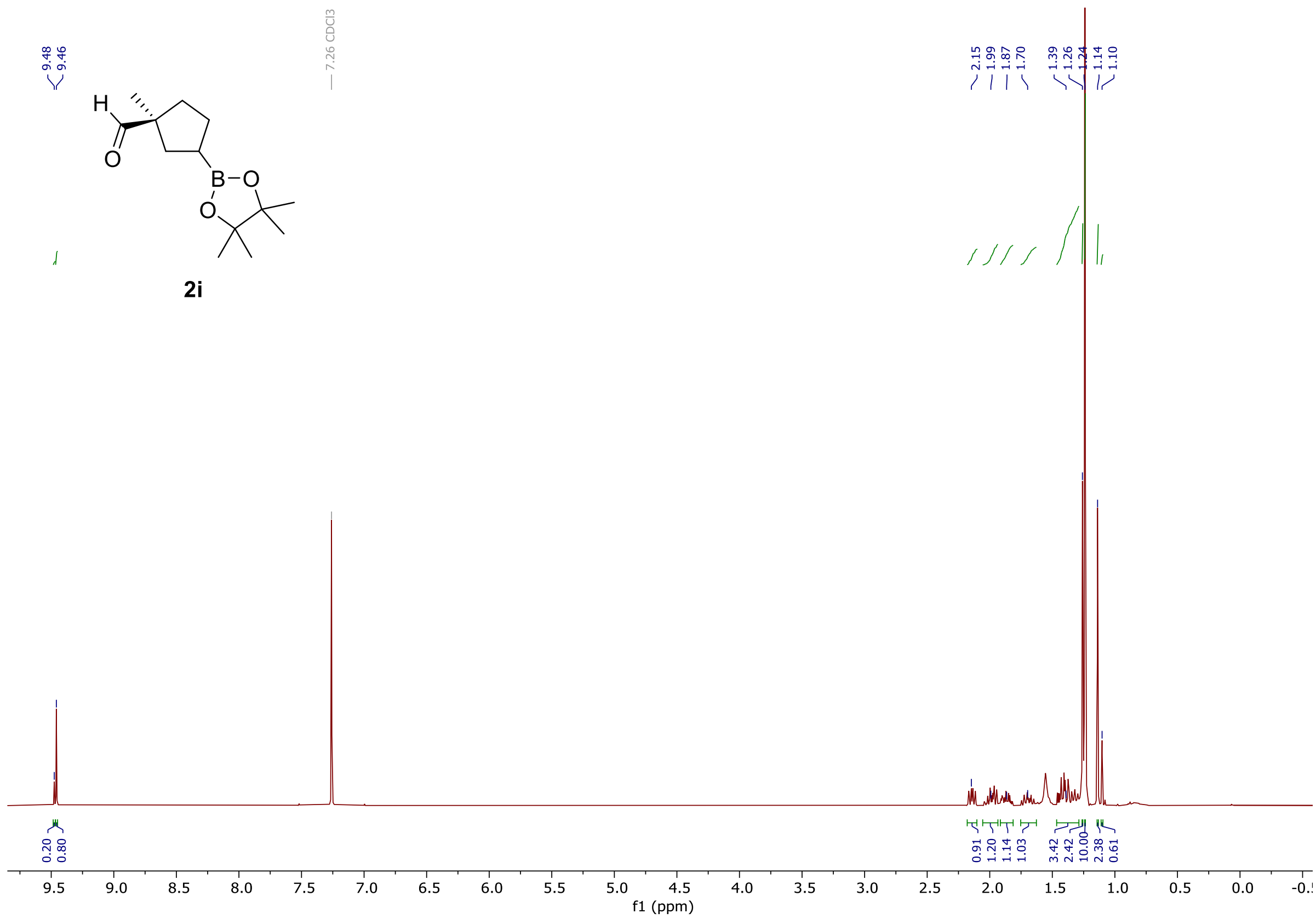

^{13}C NMR (101 MHz, $CDCl_3$) of **2i**

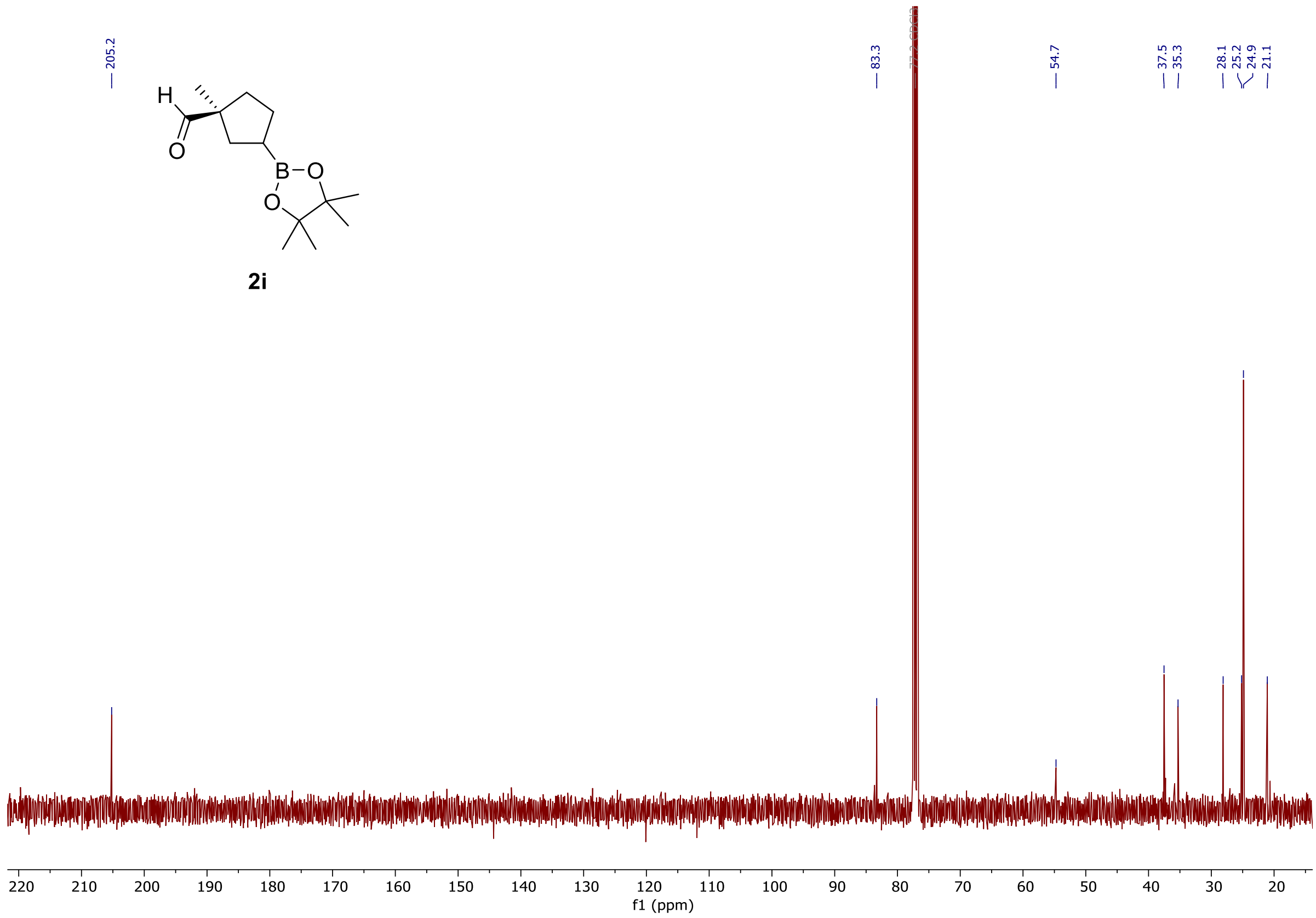

^{1}H NMR (400 MHz, $CDCl_3$) of **2j** (*see procedure*)

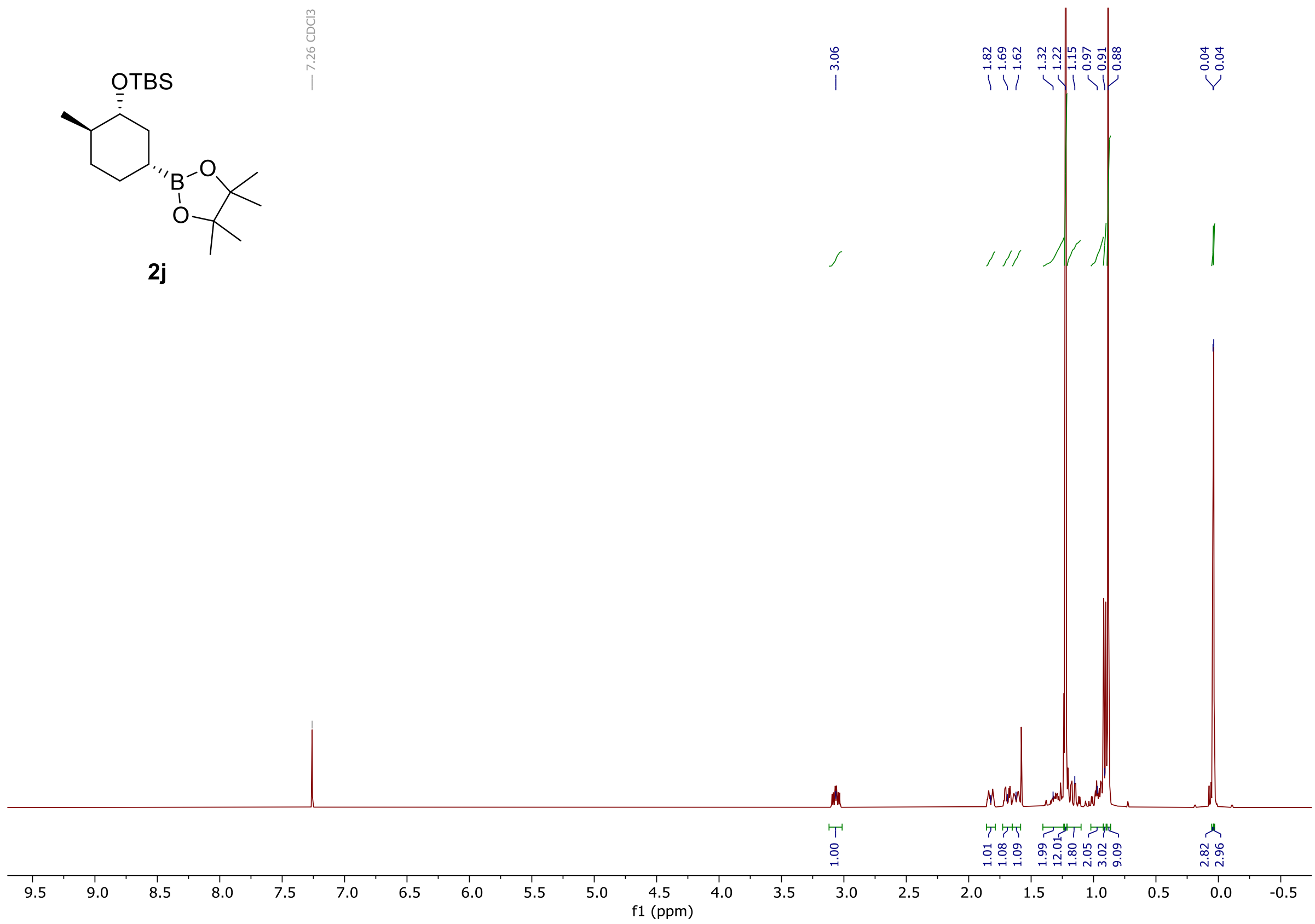

^{13}C NMR (101 MHz, $CDCl_3$) of **2j**

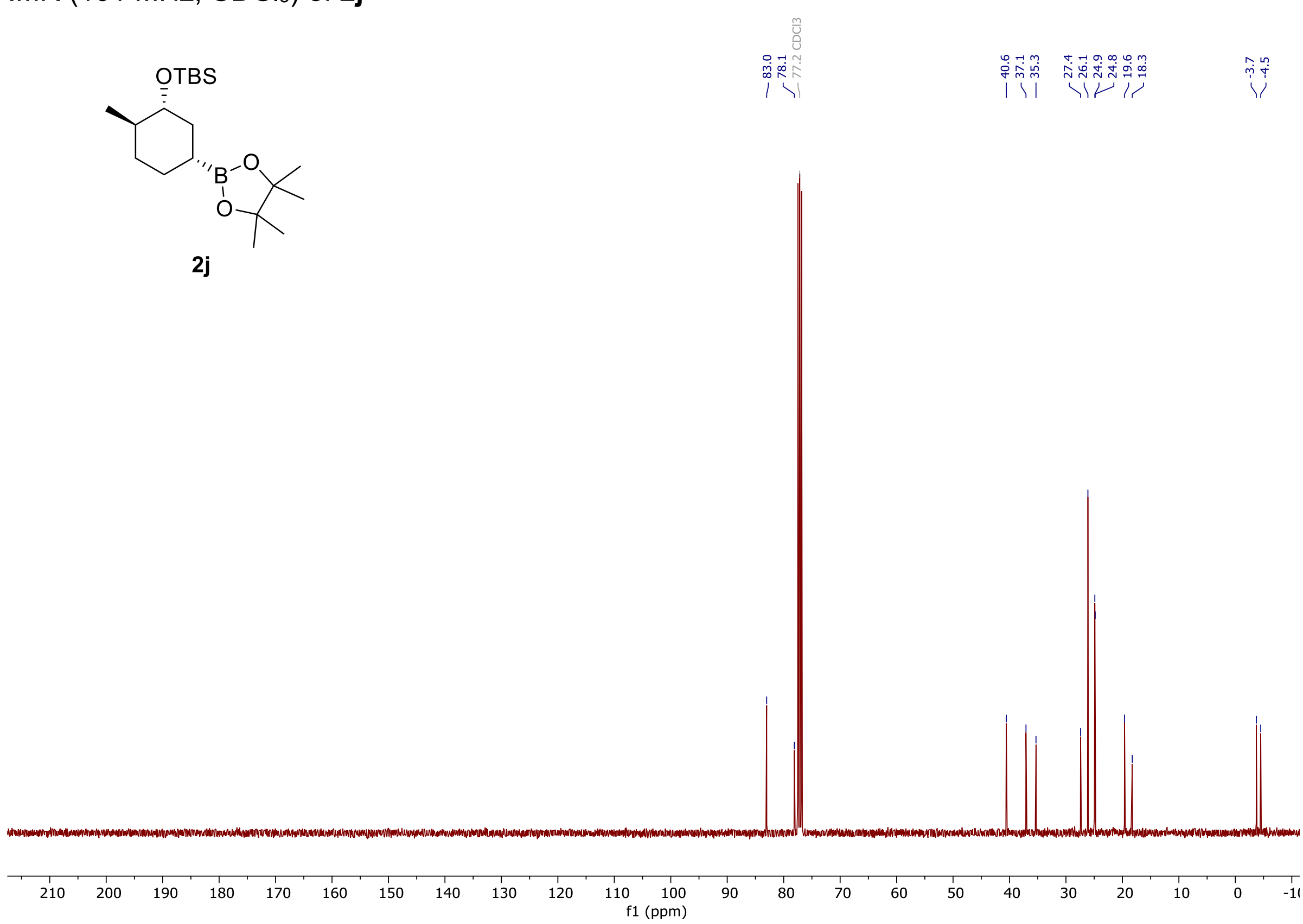

^{1}H NMR (400 MHz, $CDCl_3$) of **2k** (*see procedure*)

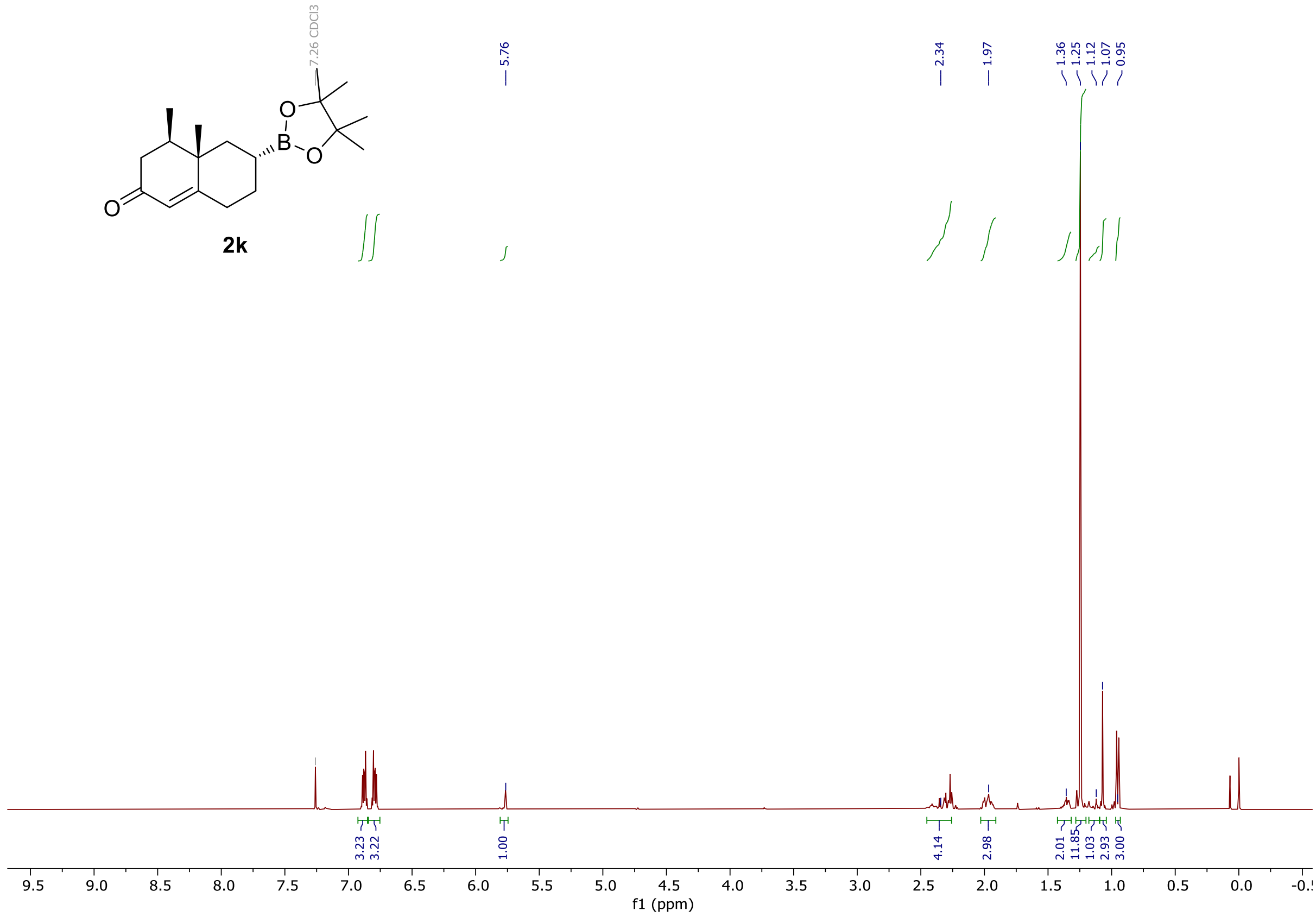

^{13}C NMR (101 MHz, $CDCl_3$) of **2k**

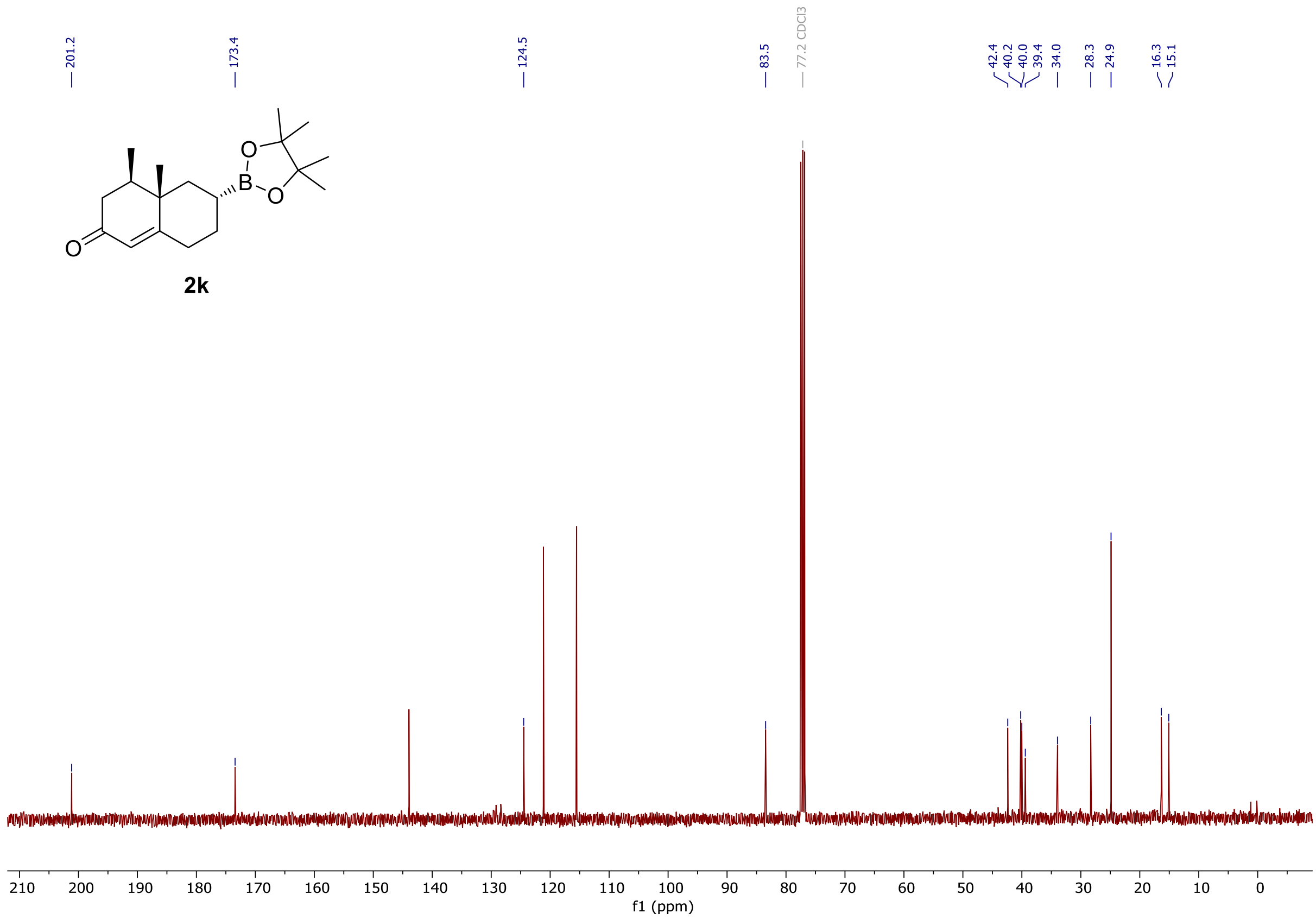

^{1}H NMR (400 MHz, $CDCl_3$) of **2l** (*see procedure*)

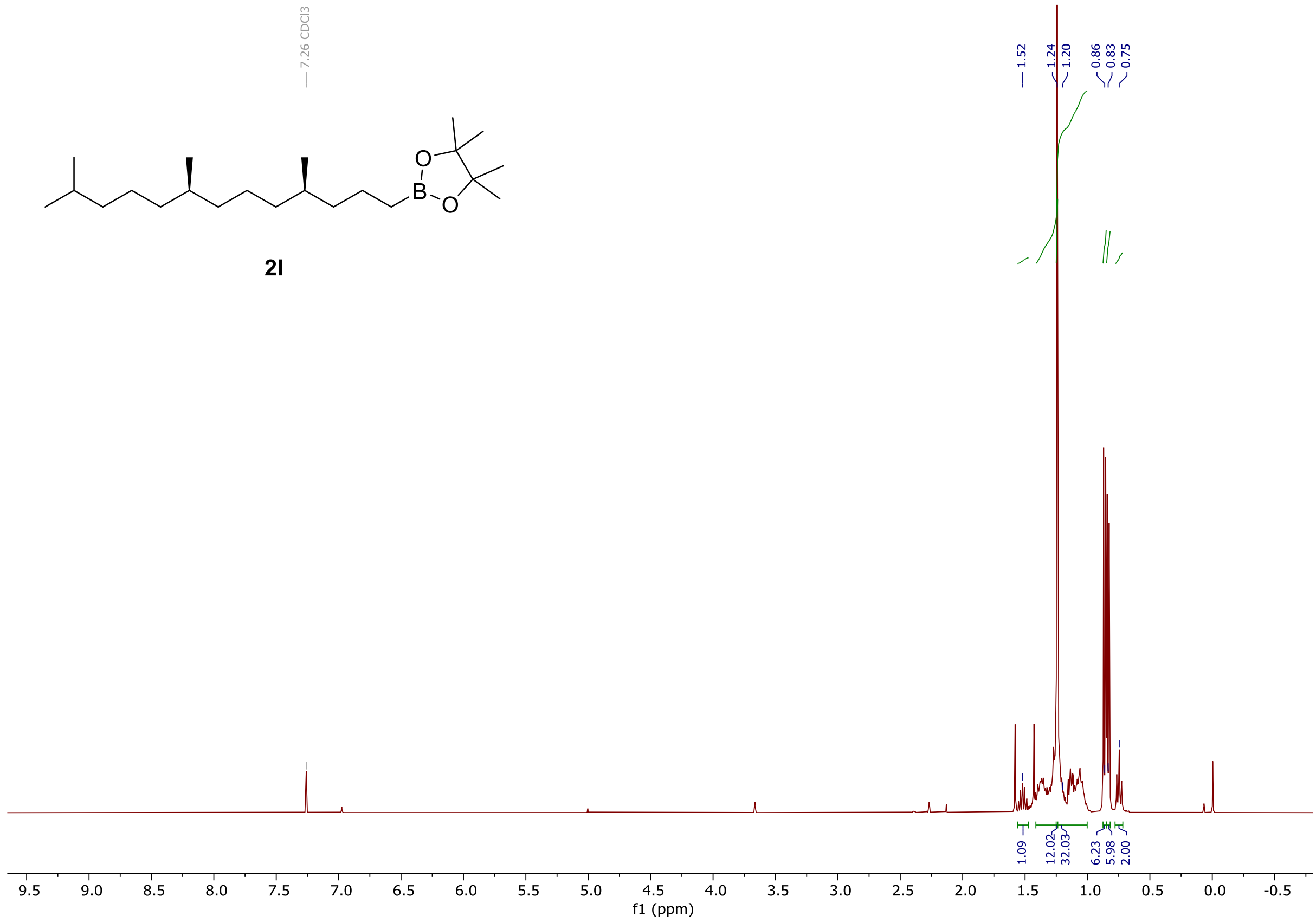

^{13}C NMR (101 MHz, $CDCl_3$) of **2l**

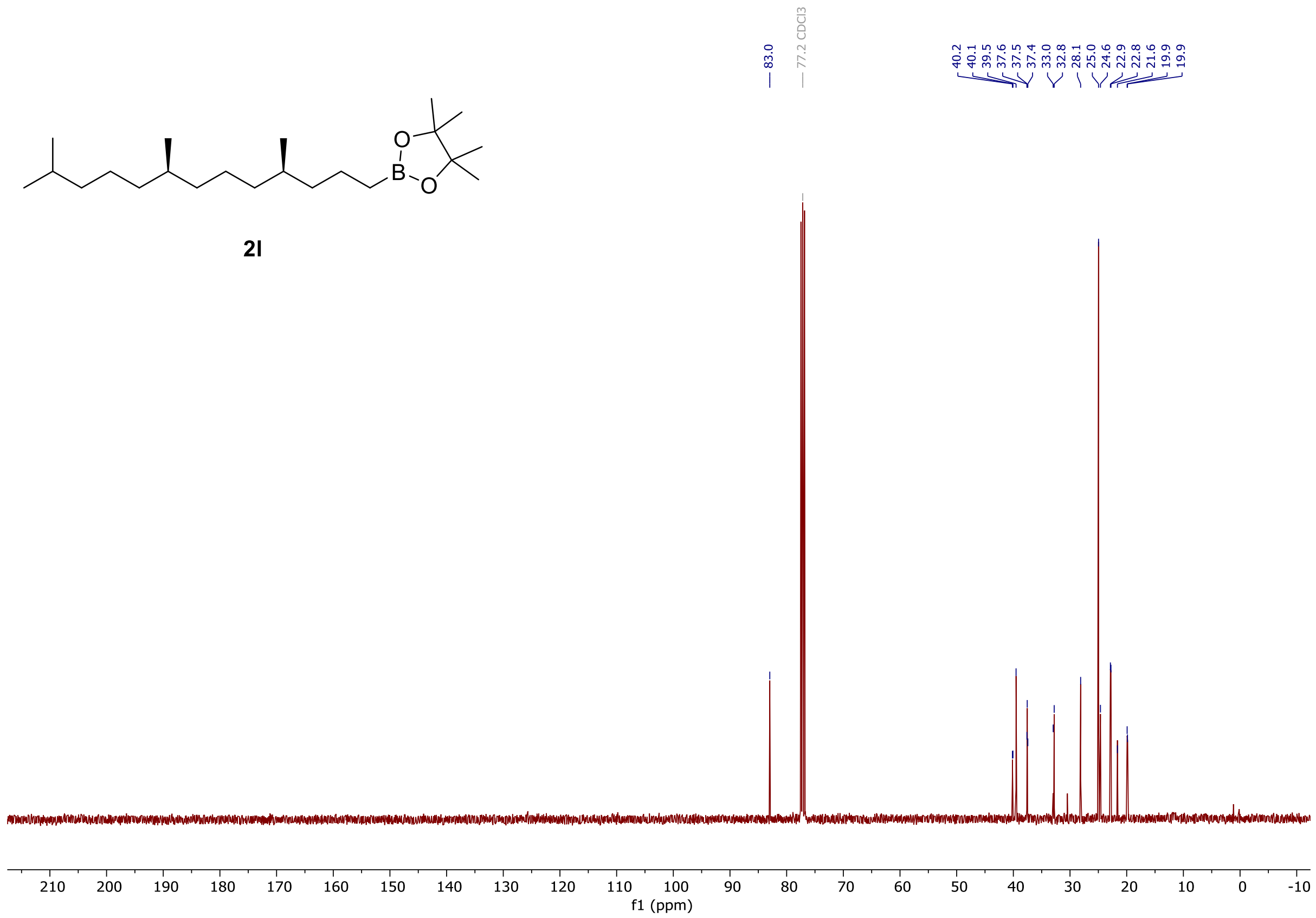

^{1}H NMR (400 MHz, $CDCl_3$) of **2m** (*see procedure*)

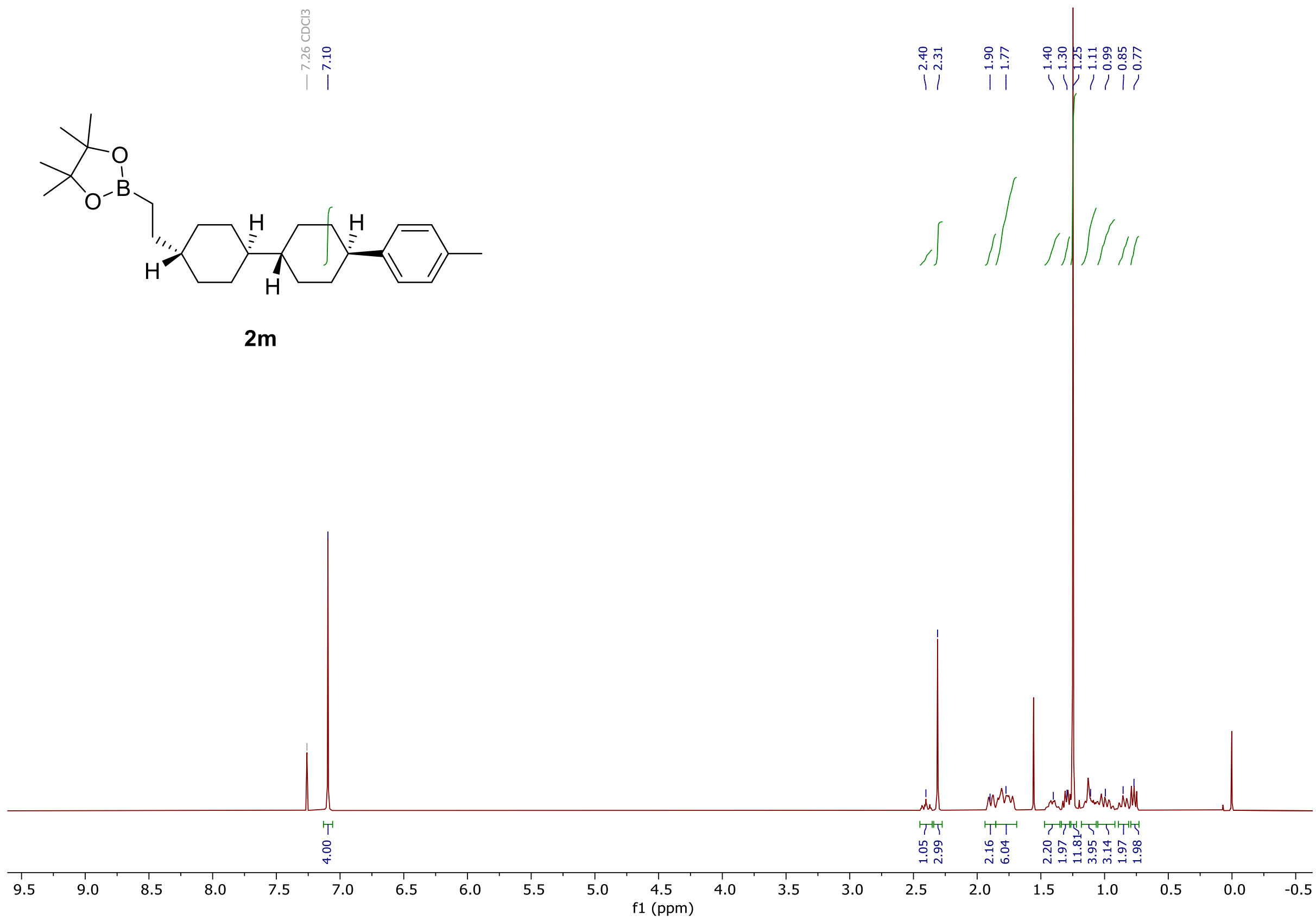

2m

^{13}C NMR (101 MHz, $CDCl_3$) of **2m**

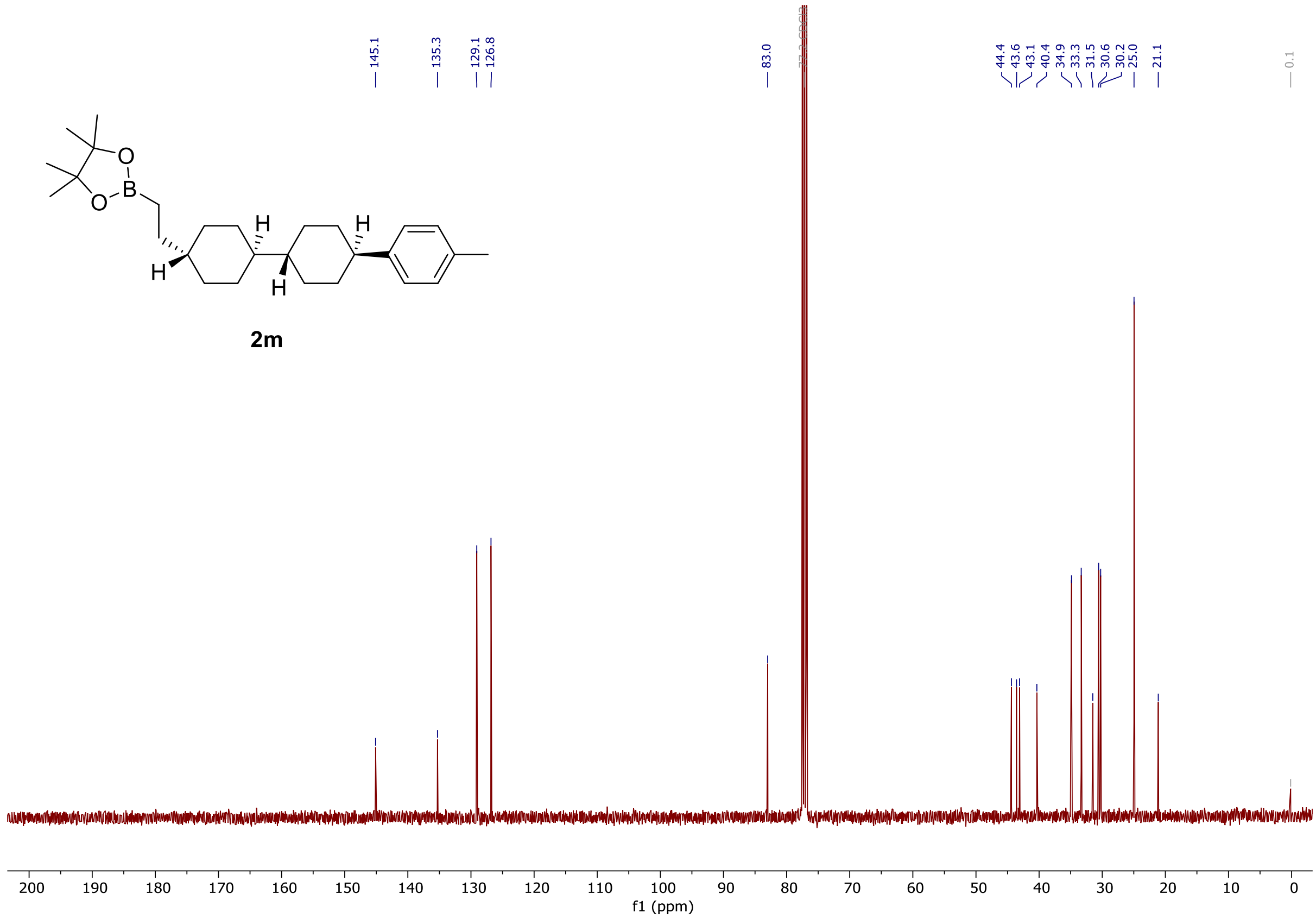

2m

^{1}H NMR (400 MHz, $CDCl_3$) of **2n** (see procedure)

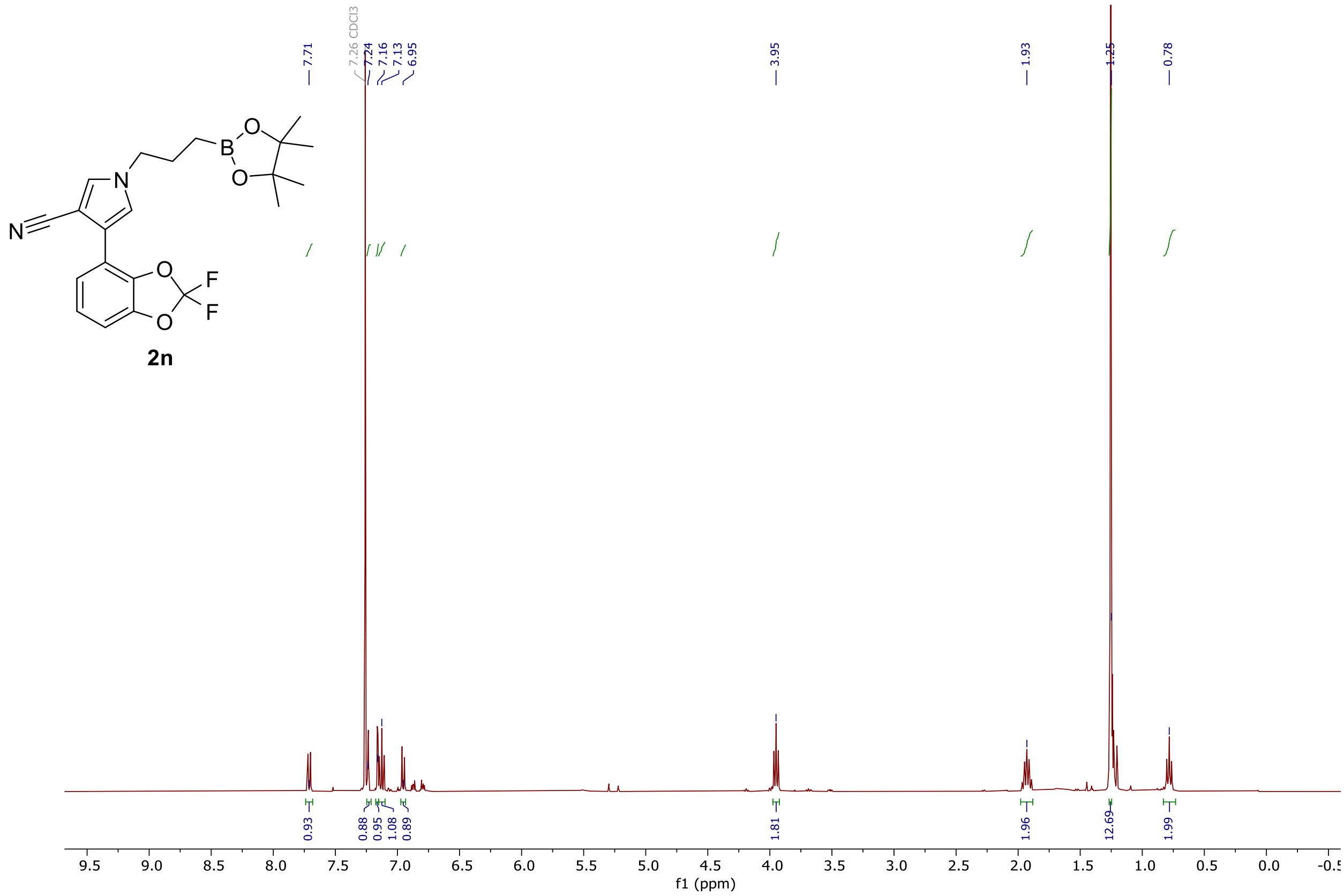

^{13}C NMR (101 MHz, $CDCl_3$) of **2n**

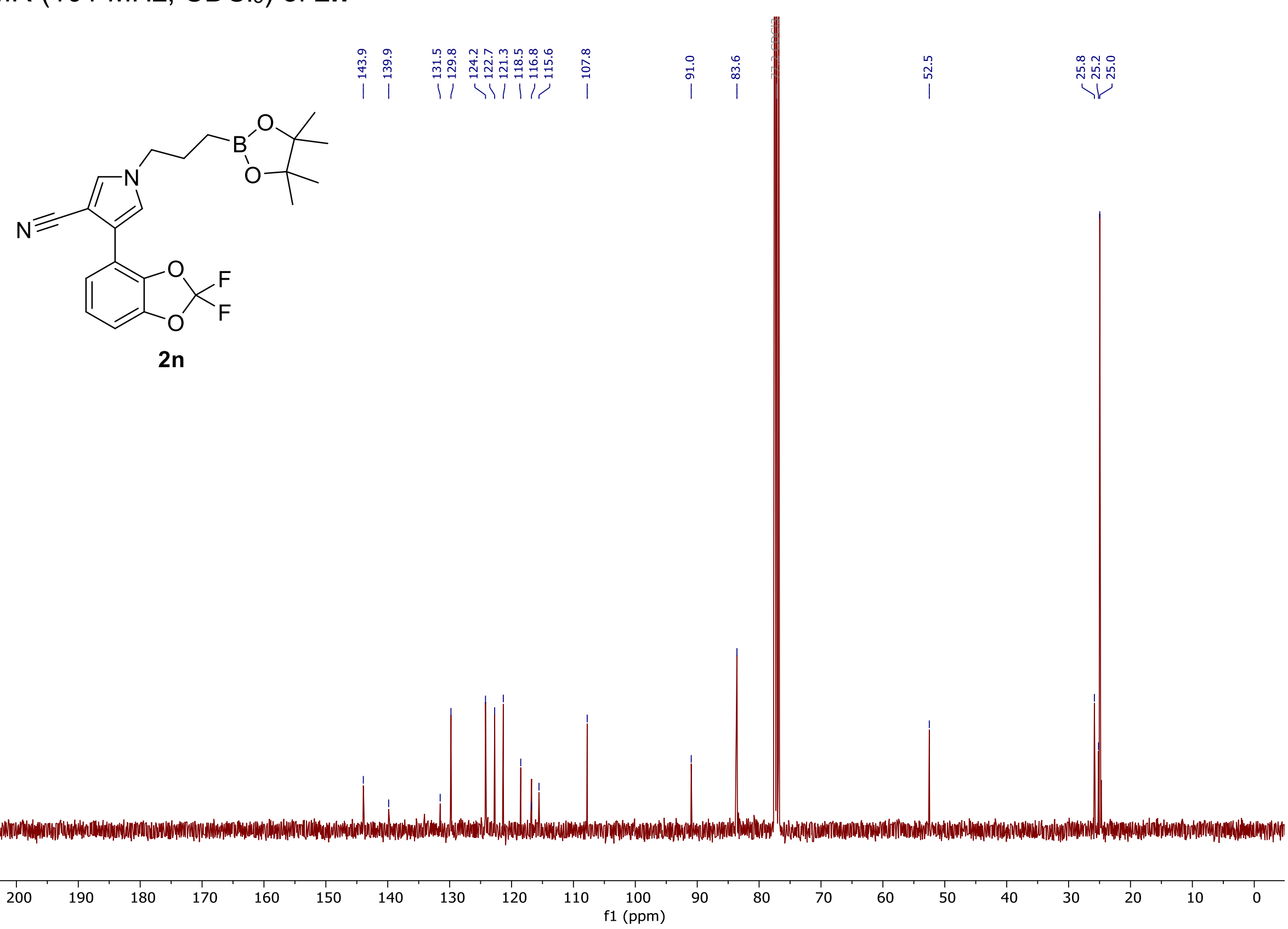

^{19}F NMR (377 MHz, $CDCl_3$) of **2n**

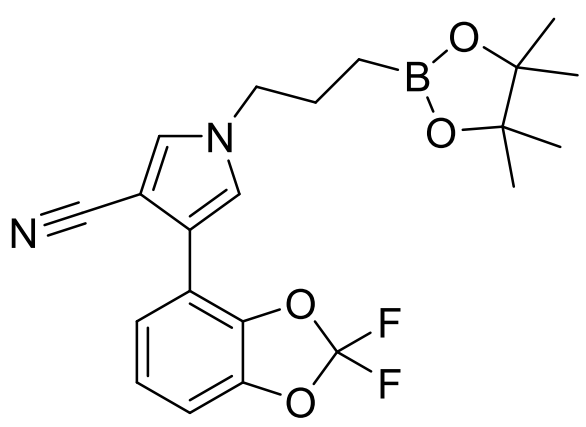

2n

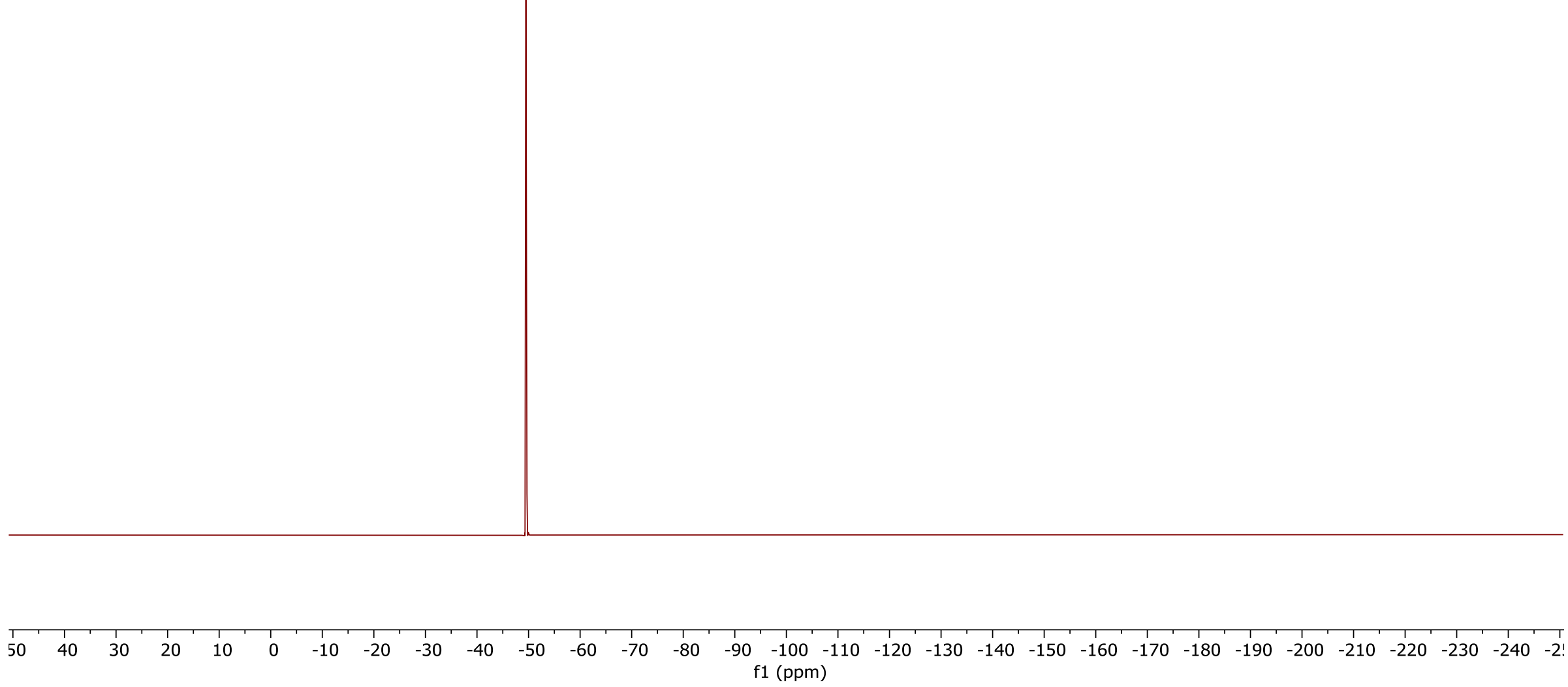

^{1}H NMR (400 MHz, $CDCl_3$) of **2o** (*see procedure*)

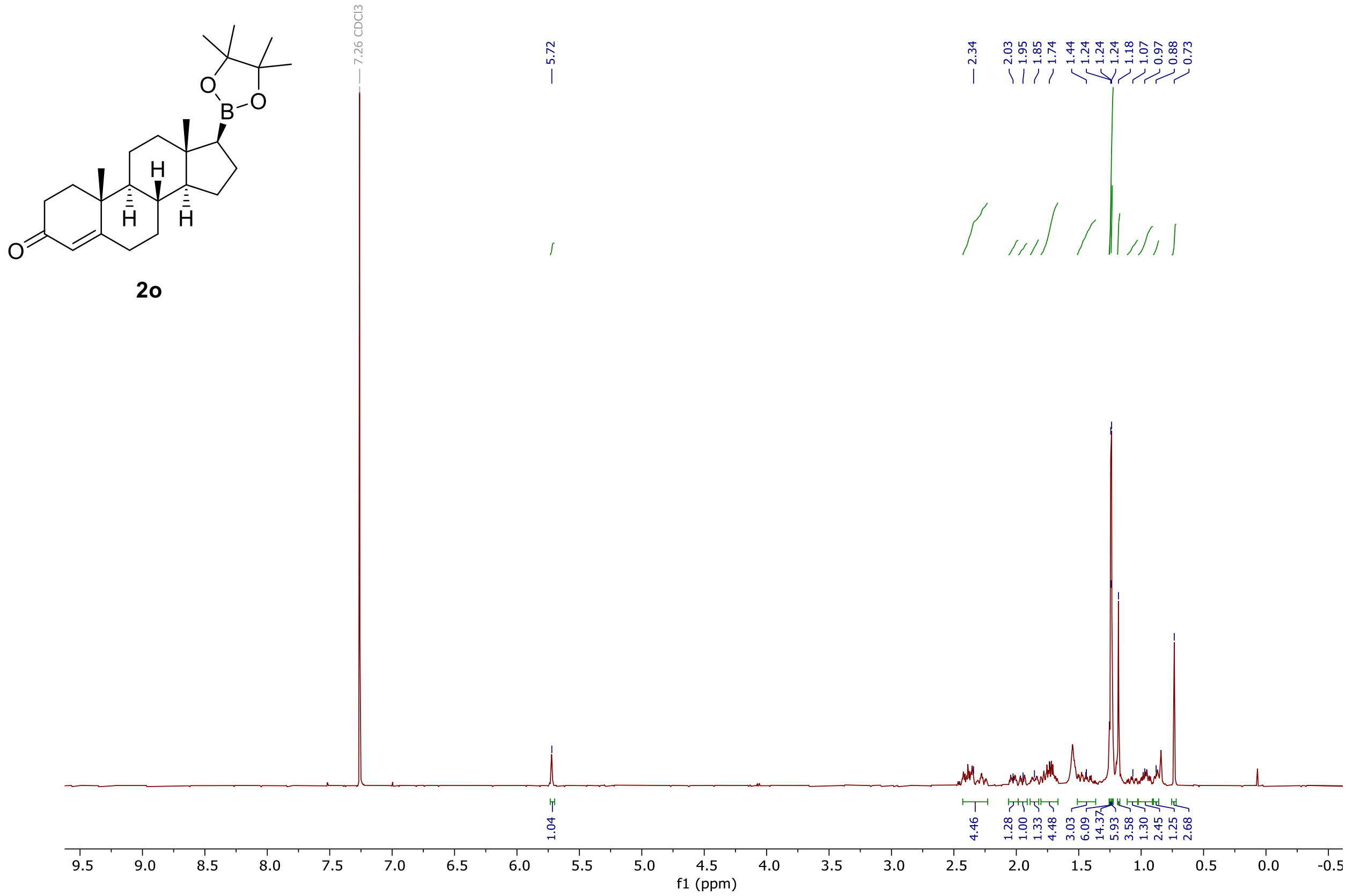

^{13}C NMR (151 MHz, $CDCl_3$) of **2o**

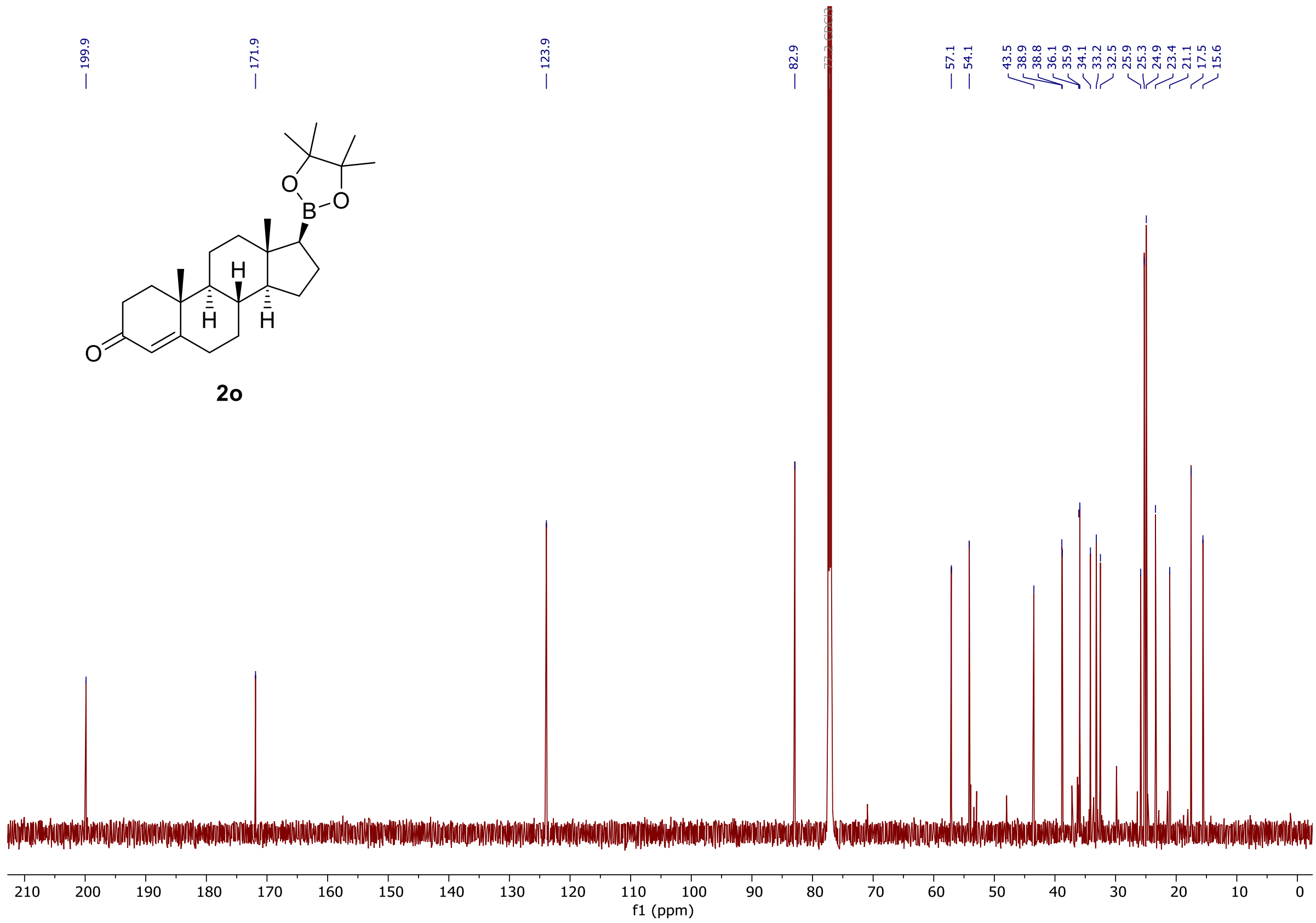

^{1}H NMR (400 MHz, $CDCl_3$) of **2p** (*see procedure*)

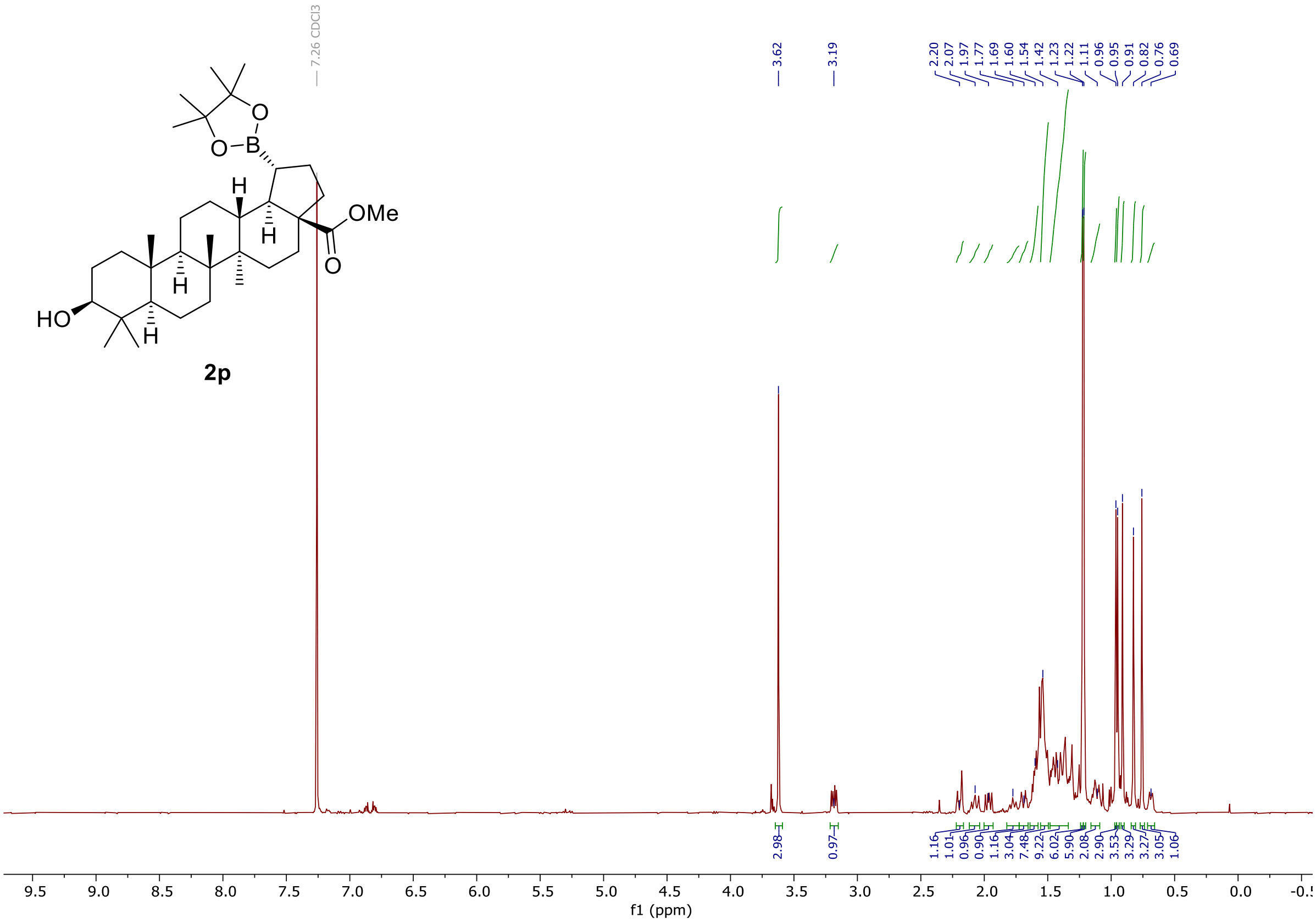

^{13}C NMR (101 MHz, $CDCl_3$) of **2p**

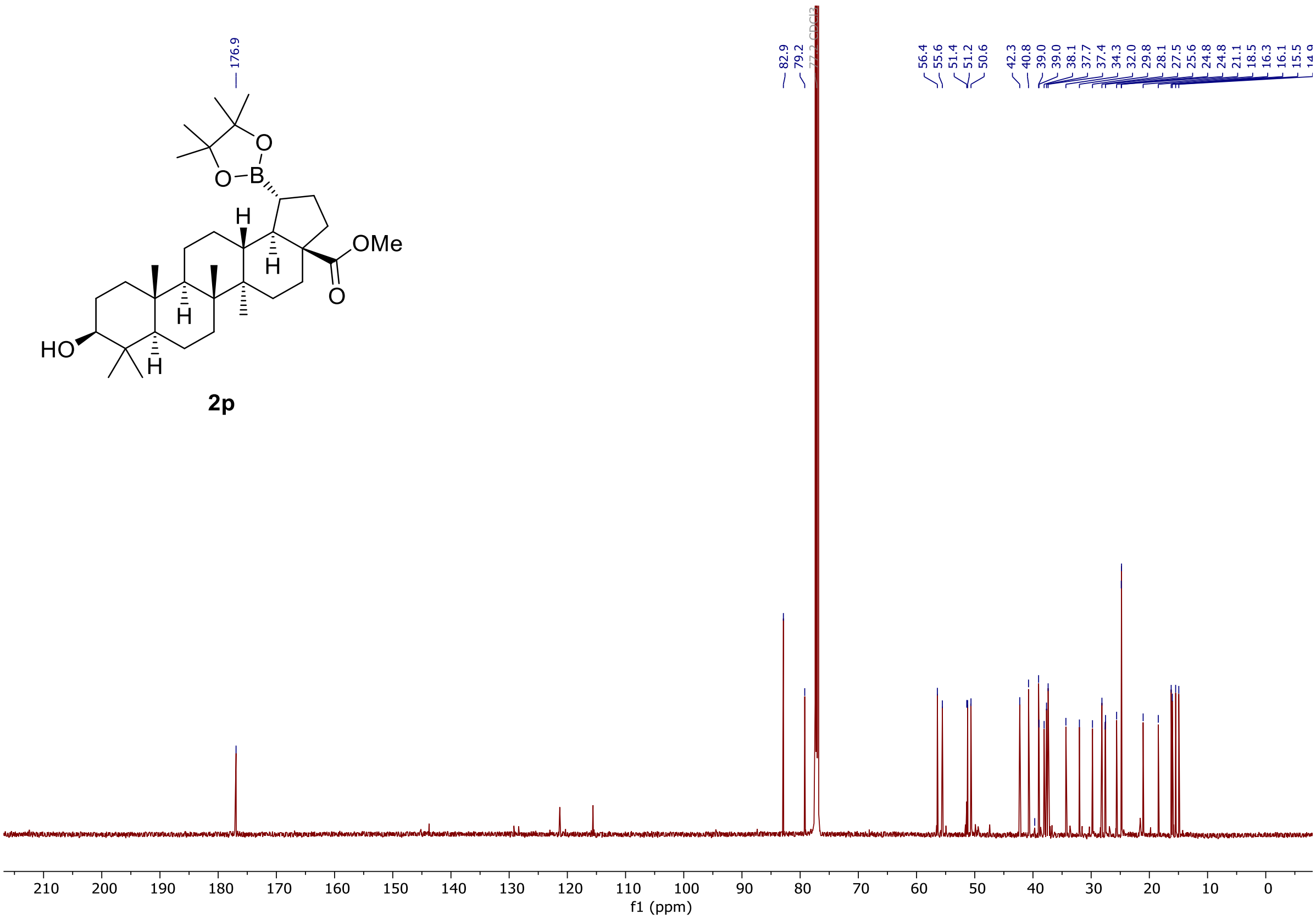

^{1}H NMR (400 MHz, $CDCl_3$) of **2q** (*see procedure*)

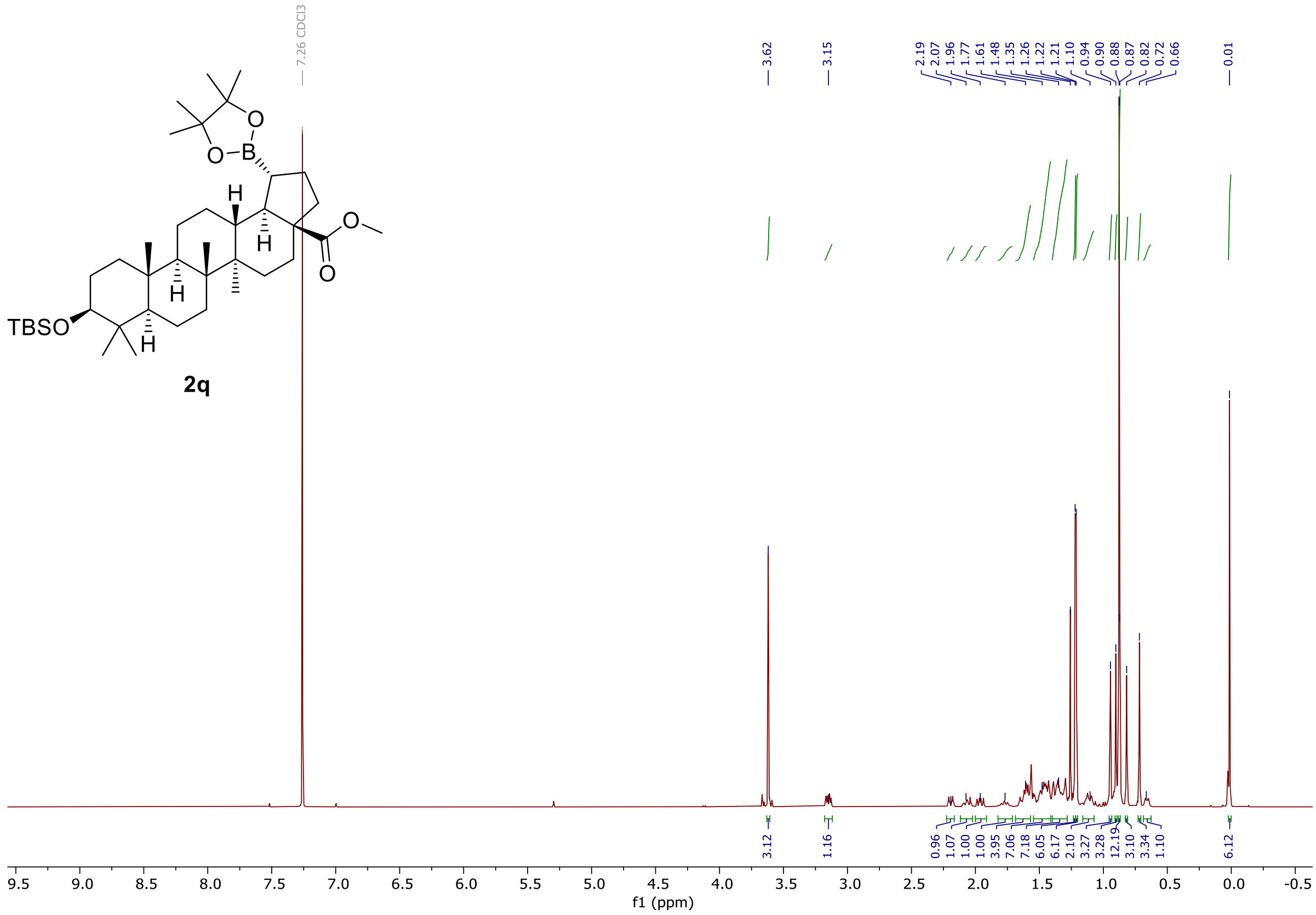

^{13}C NMR (101 MHz, $CDCl_3$) of **2q**

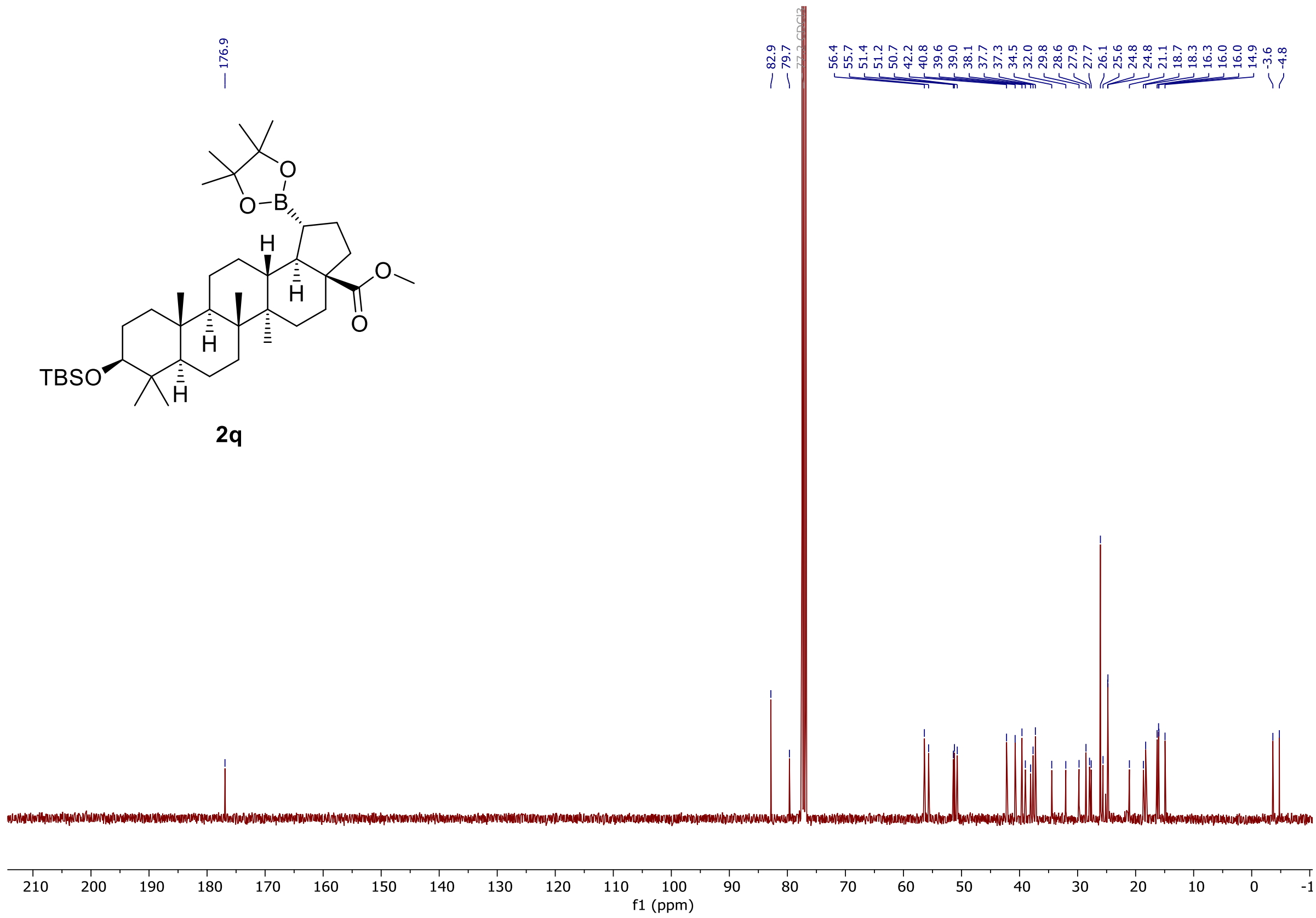

^{1}H NMR (400 MHz, $CDCl_3$) of **2r** (*see procedure*)

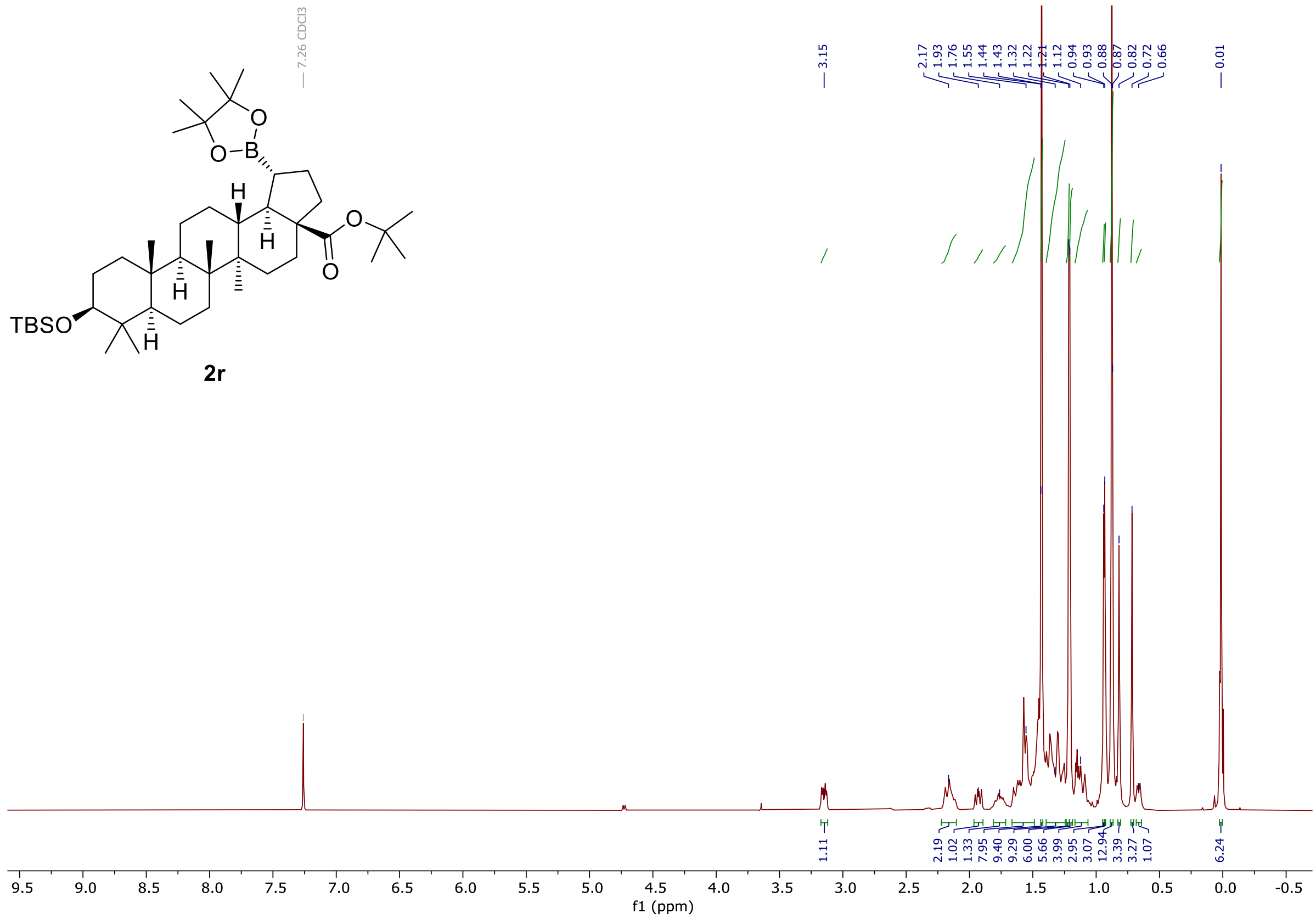

^{13}C NMR (126 MHz, $CDCl_3$) of **2r**

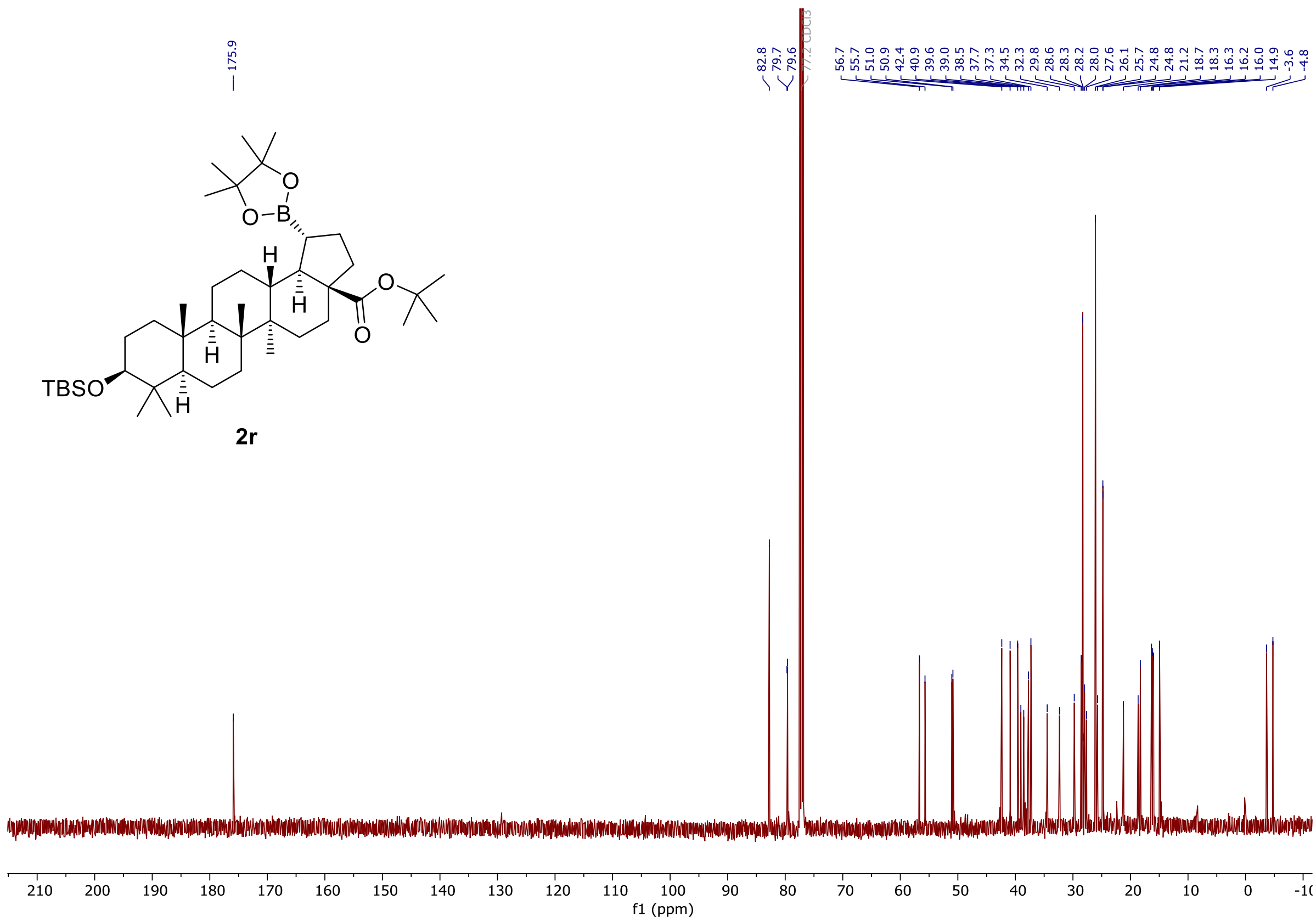

^{1}H NMR (400 MHz, $CDCl_3$) of **4a** (*see procedure*)

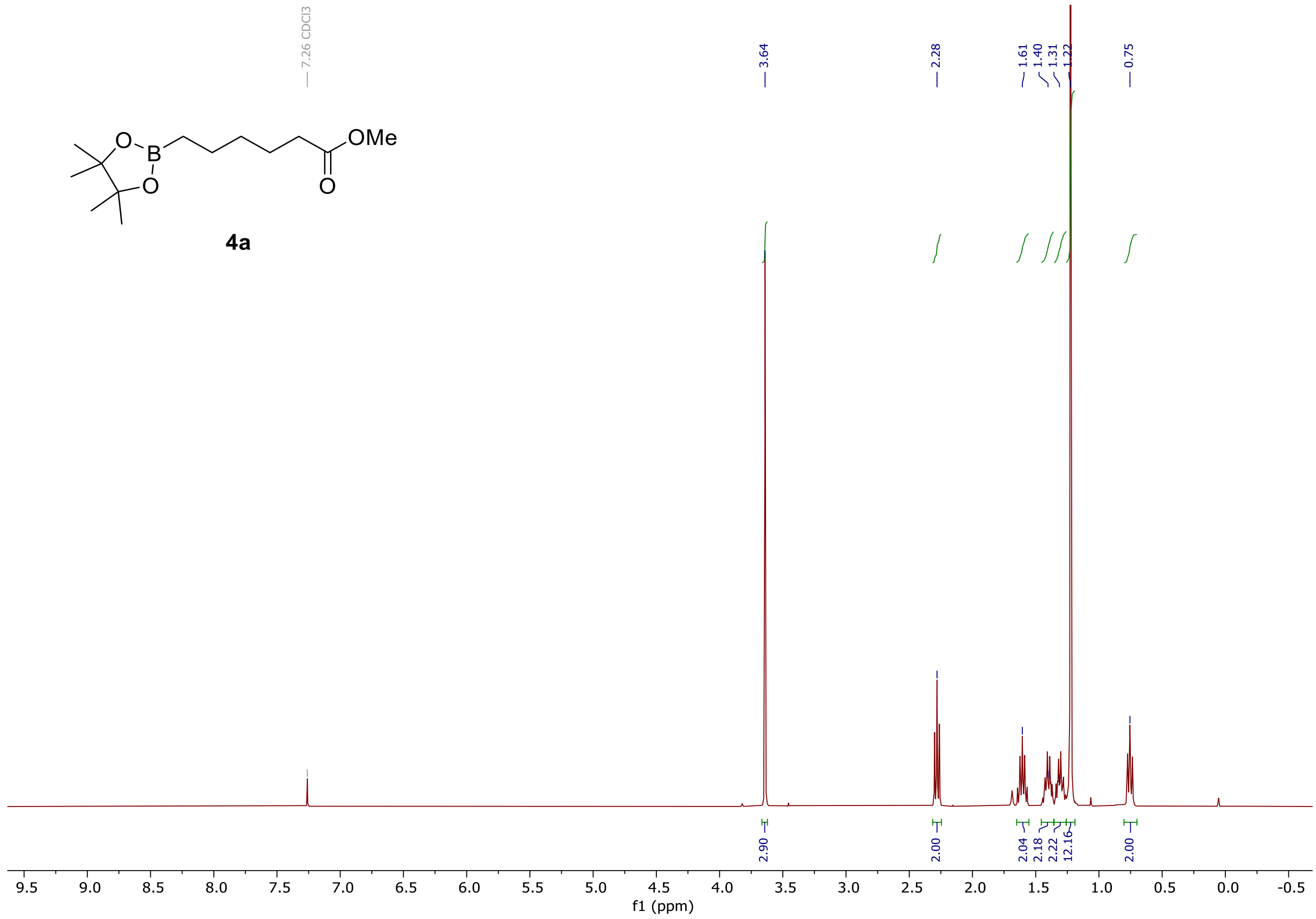

^{13}C NMR (101 MHz, $CDCl_3$) of **4a**

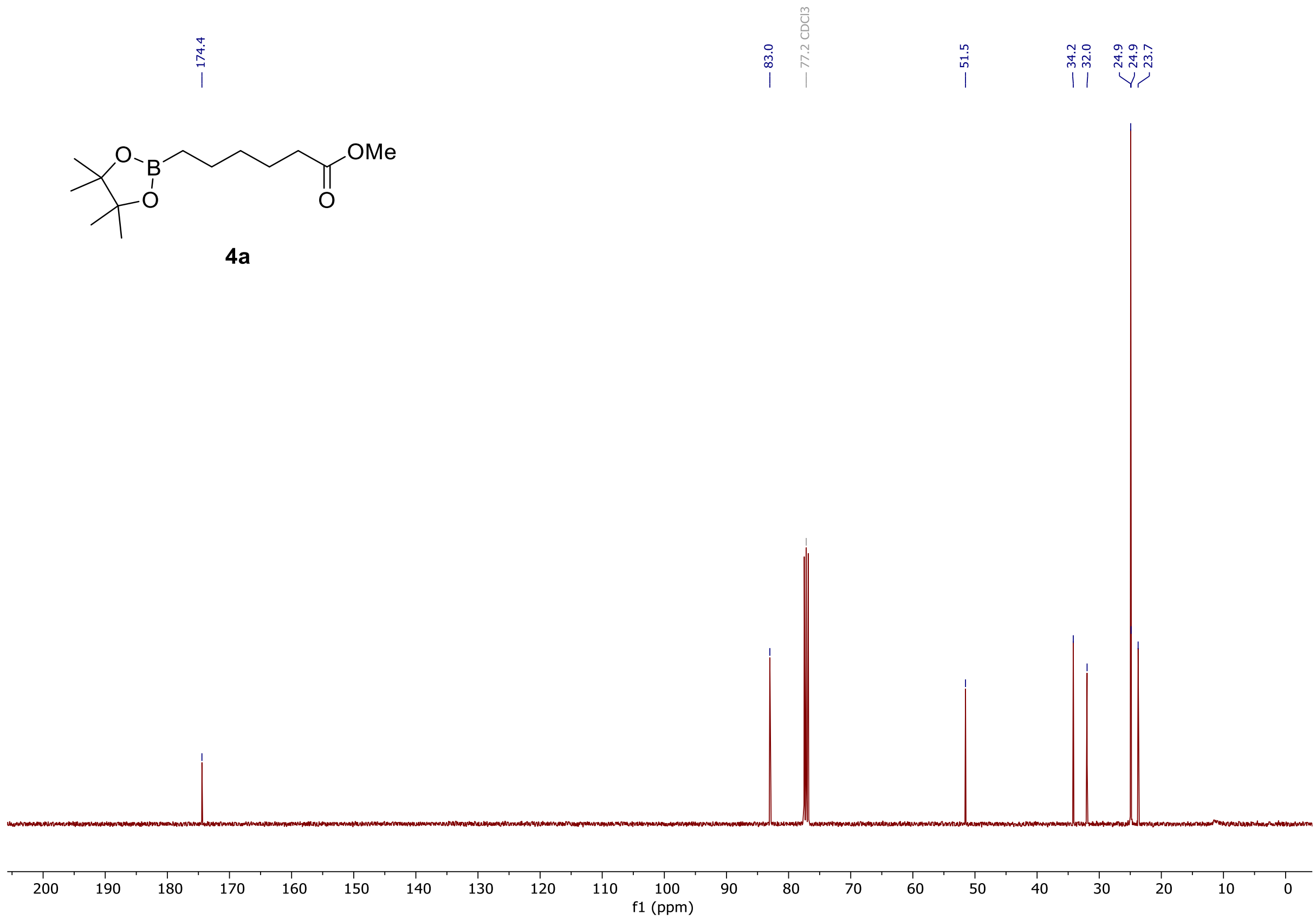

^{1}H NMR (400 MHz, $CDCl_3$) of **4b** (*see procedure*)

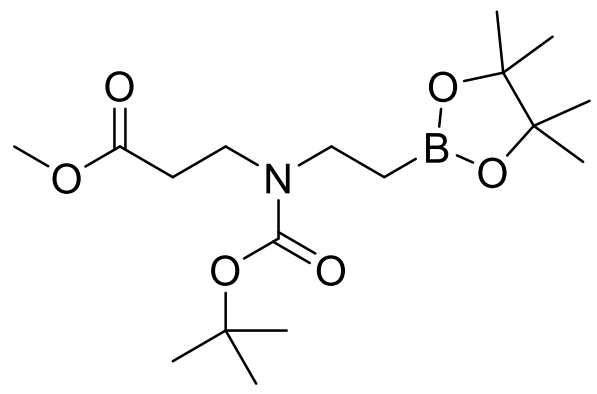

4b

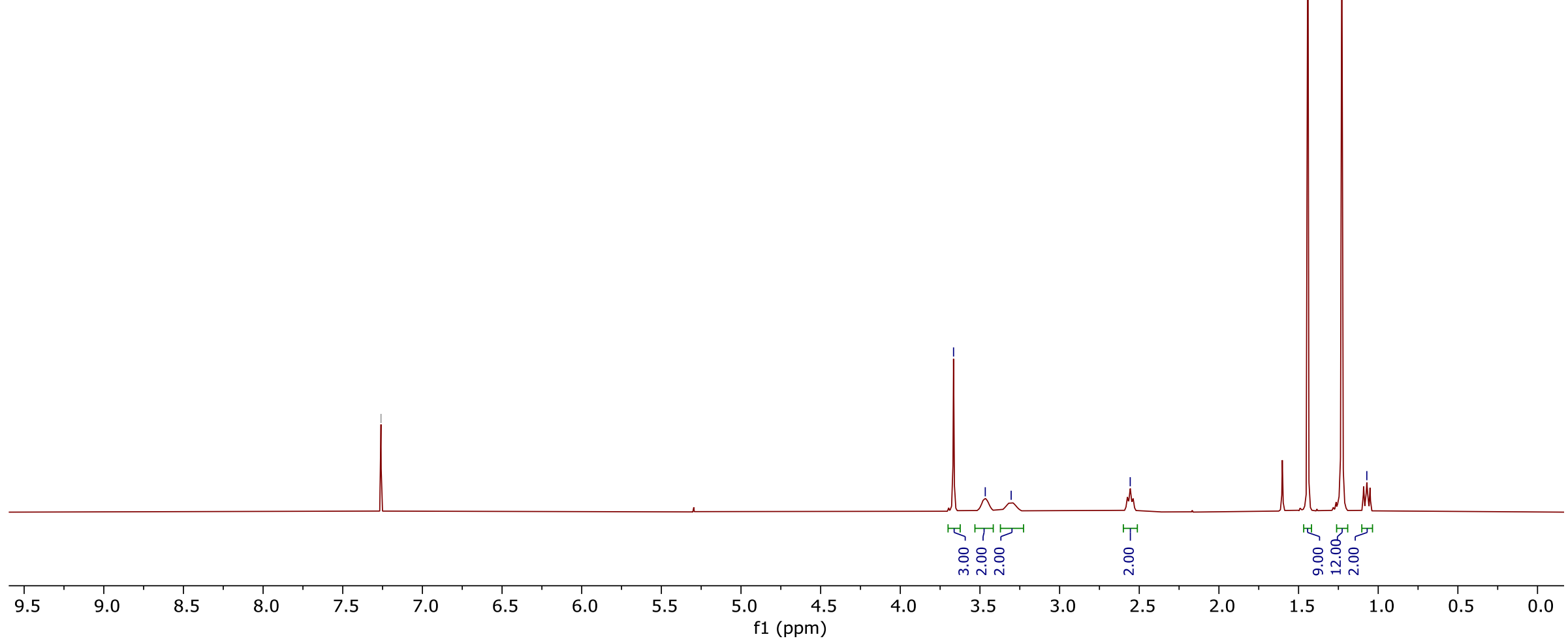

^{13}C NMR (101 MHz, $CDCl_3$) of **4b**

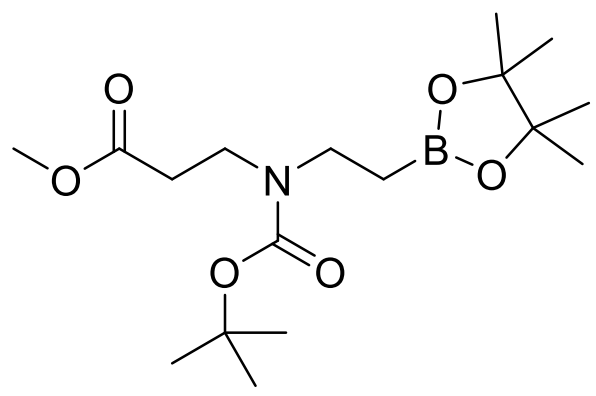

4b

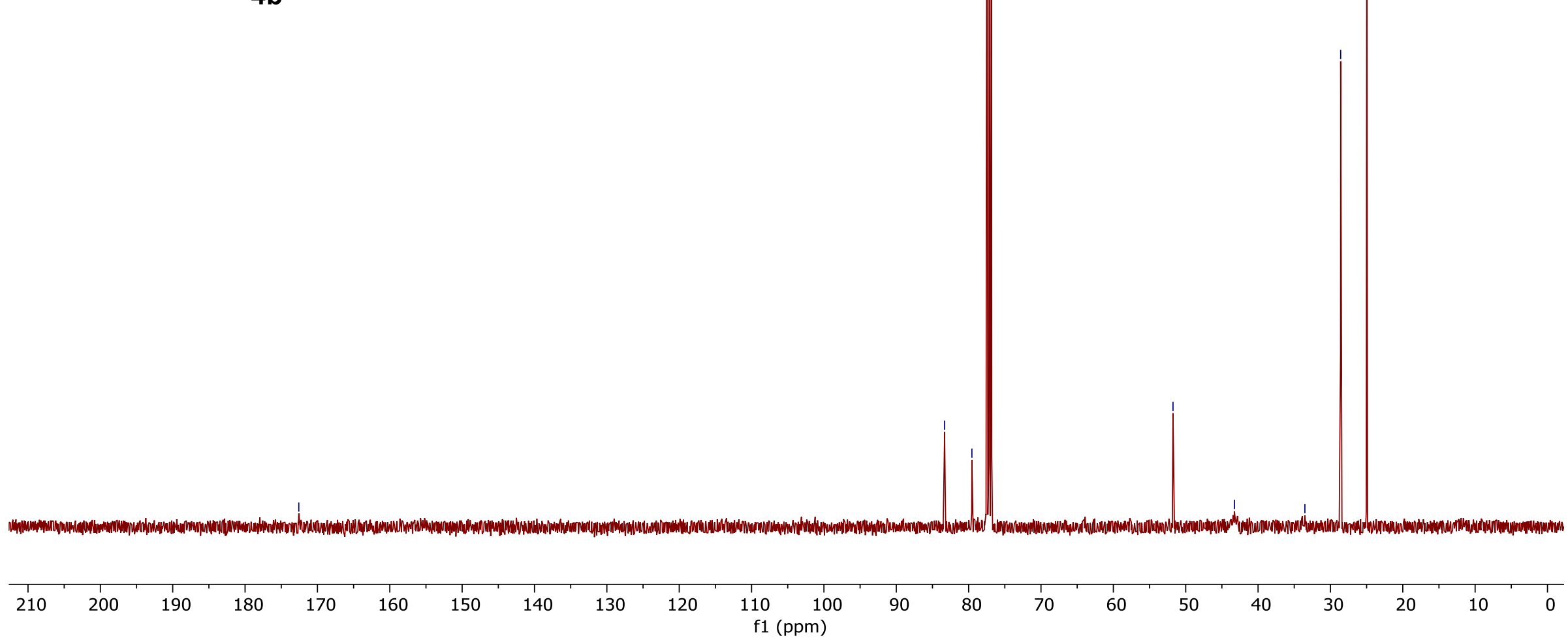

^{1}H NMR (400 MHz, $CDCl_3$) of **4ca** and **4cb** (*see procedure*)

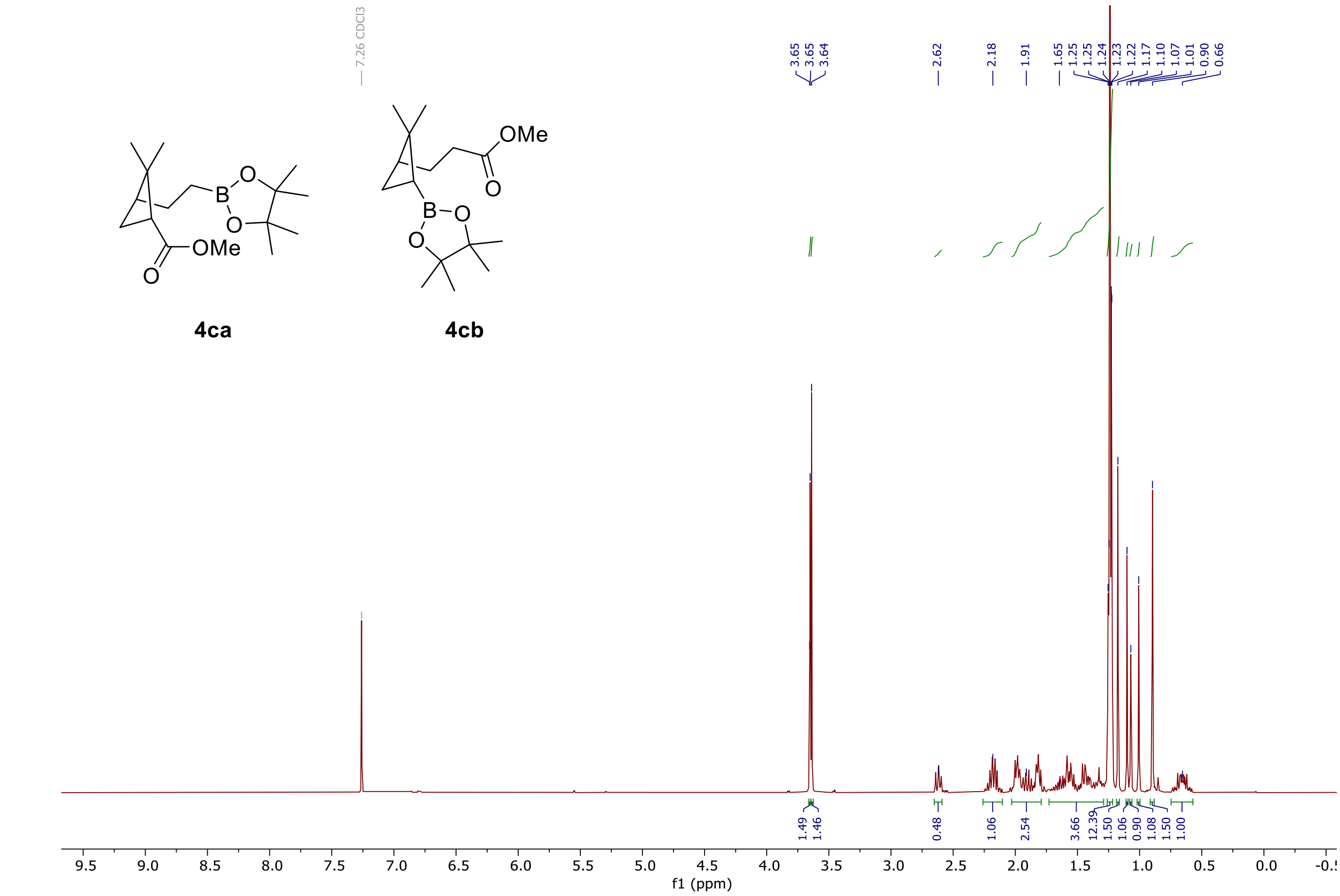

^{13}C NMR (101 MHz, $CDCl_3$) of **4ca** and **4cb**

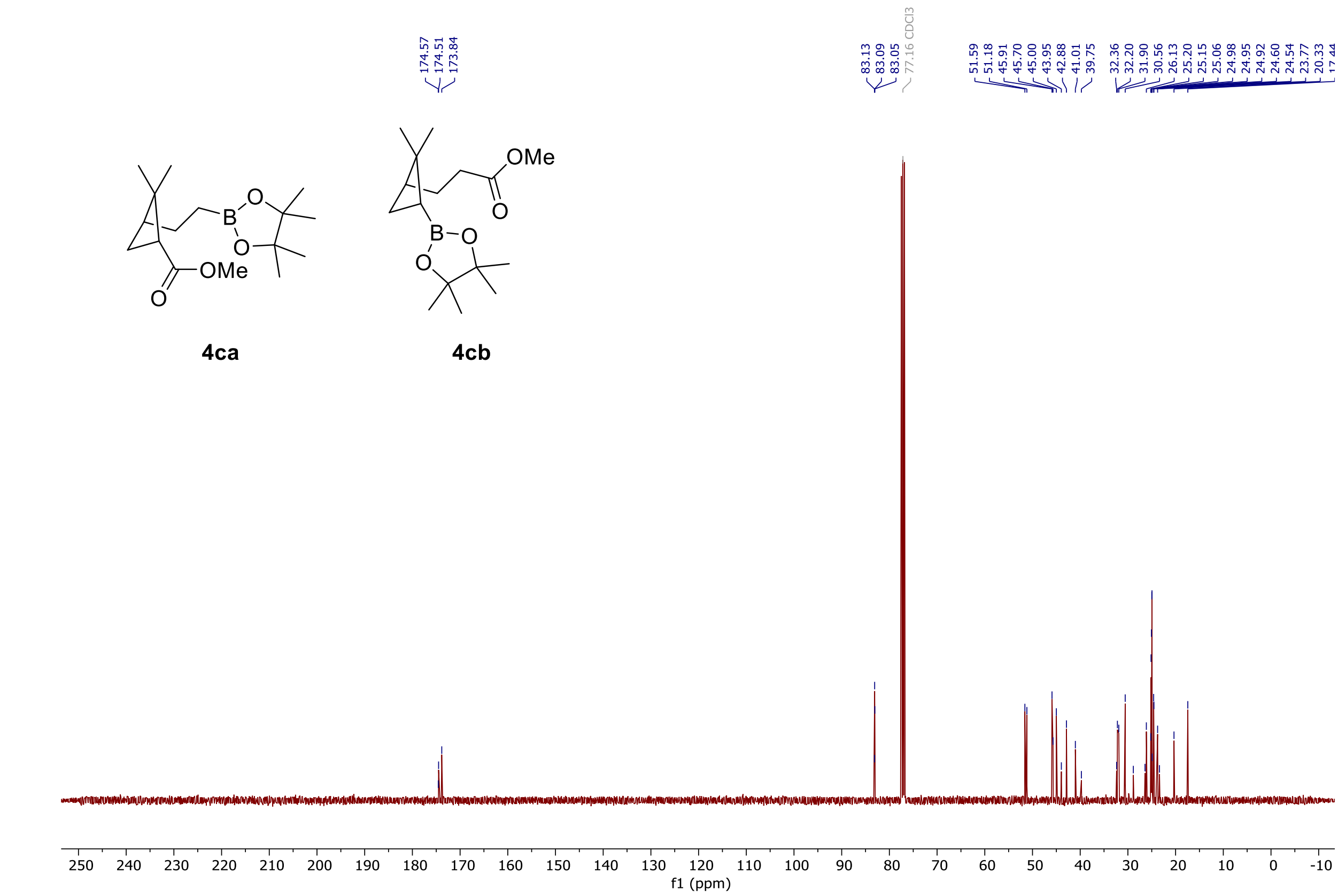

^{1}H NMR (400 MHz, $CDCl_3$) of **4d** (*see procedure*)

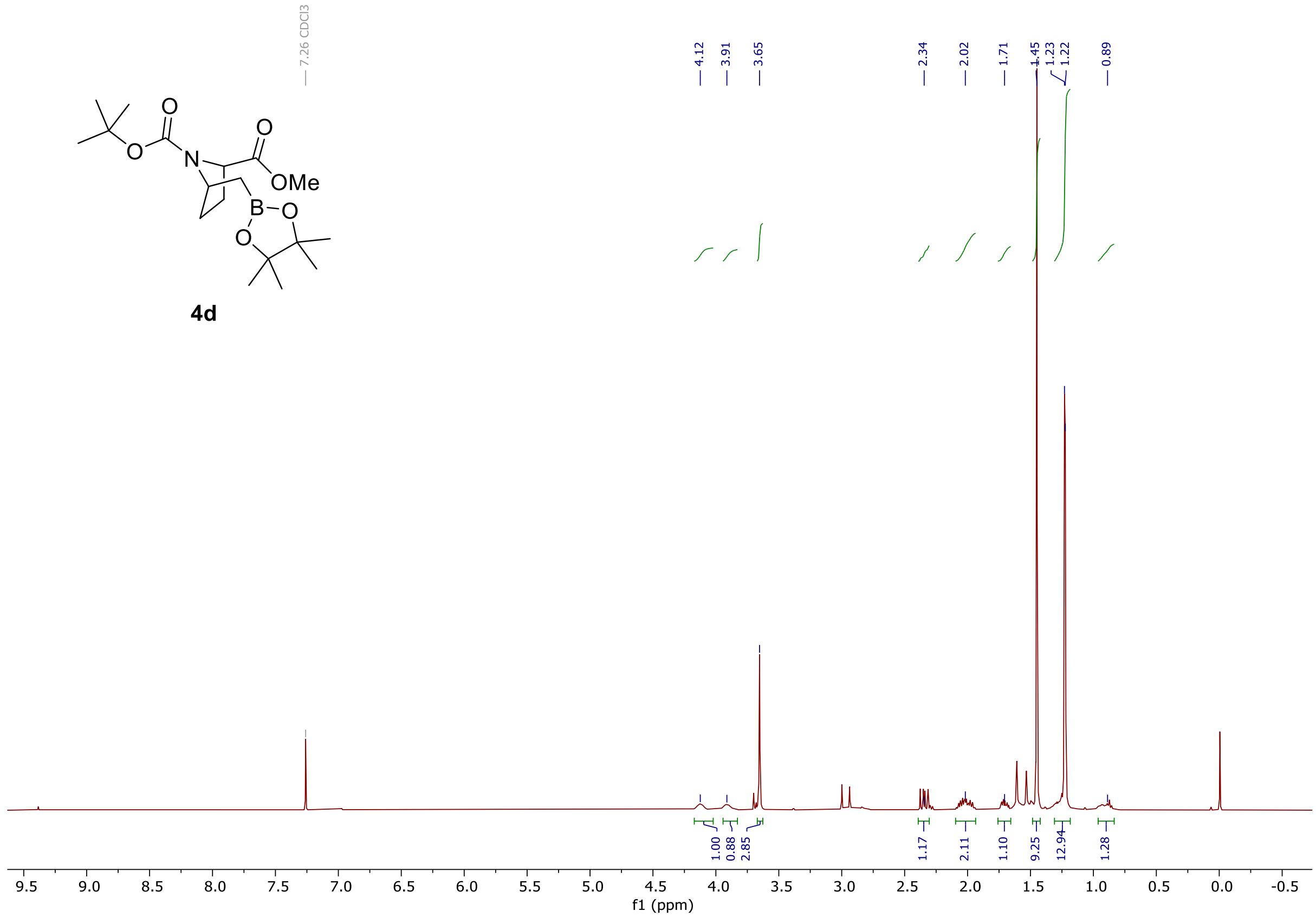

^{13}C NMR (101 MHz, $CDCl_3$) of **4d**

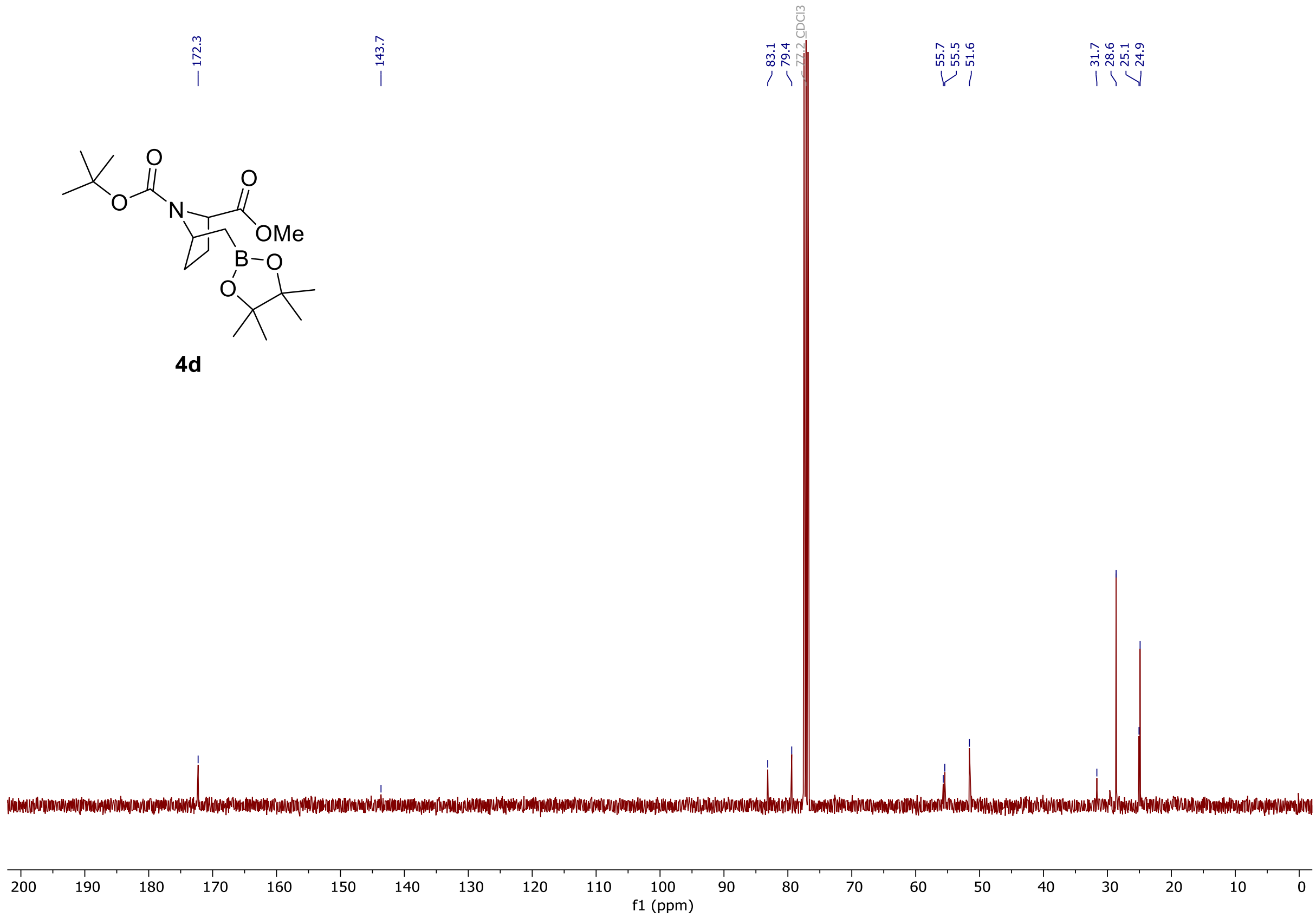

^{1}H NMR (400 MHz, $CDCl_3$) of **4e** (*see procedure*)

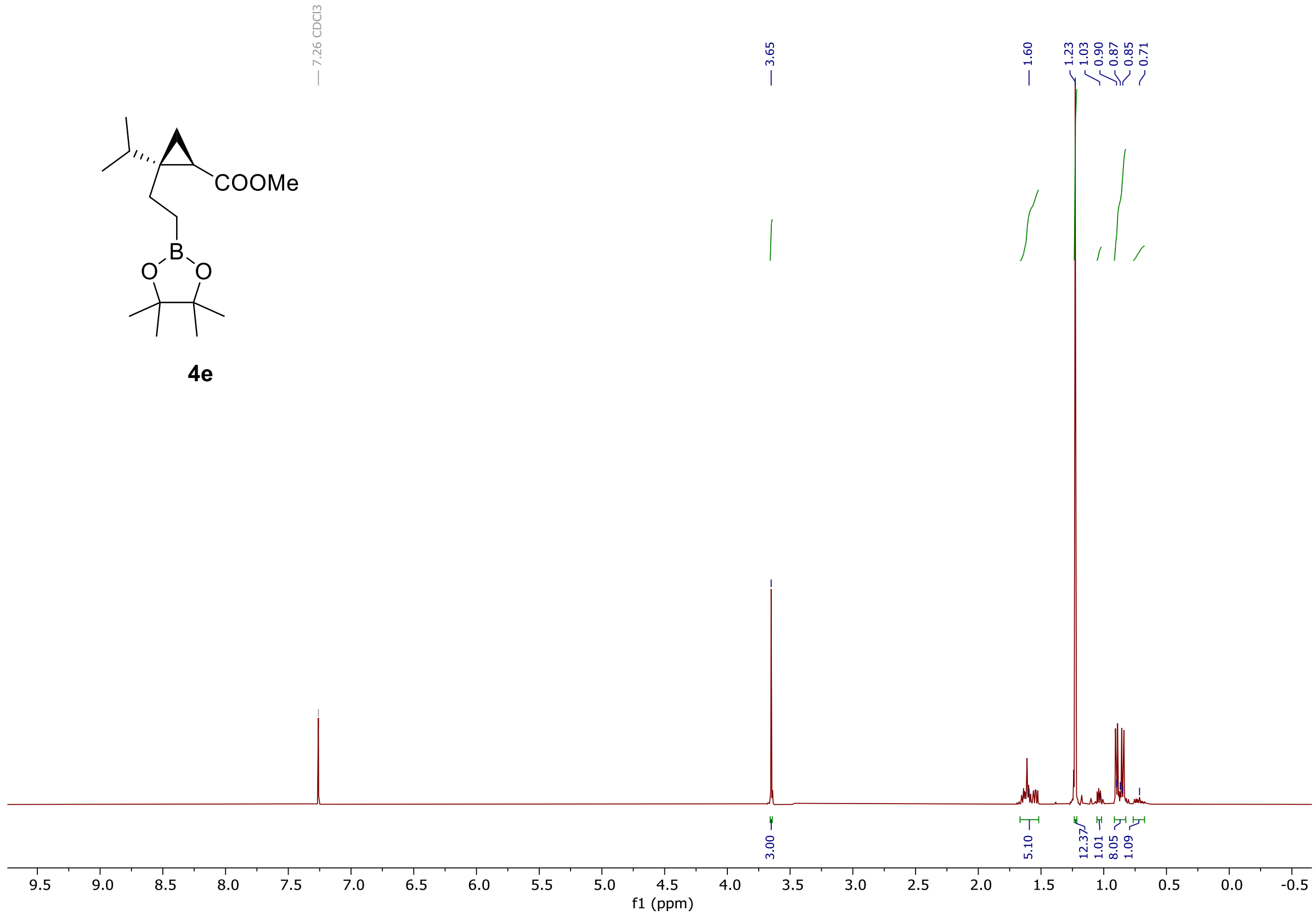

^{13}C NMR (101 MHz, $CDCl_3$) of **4e**

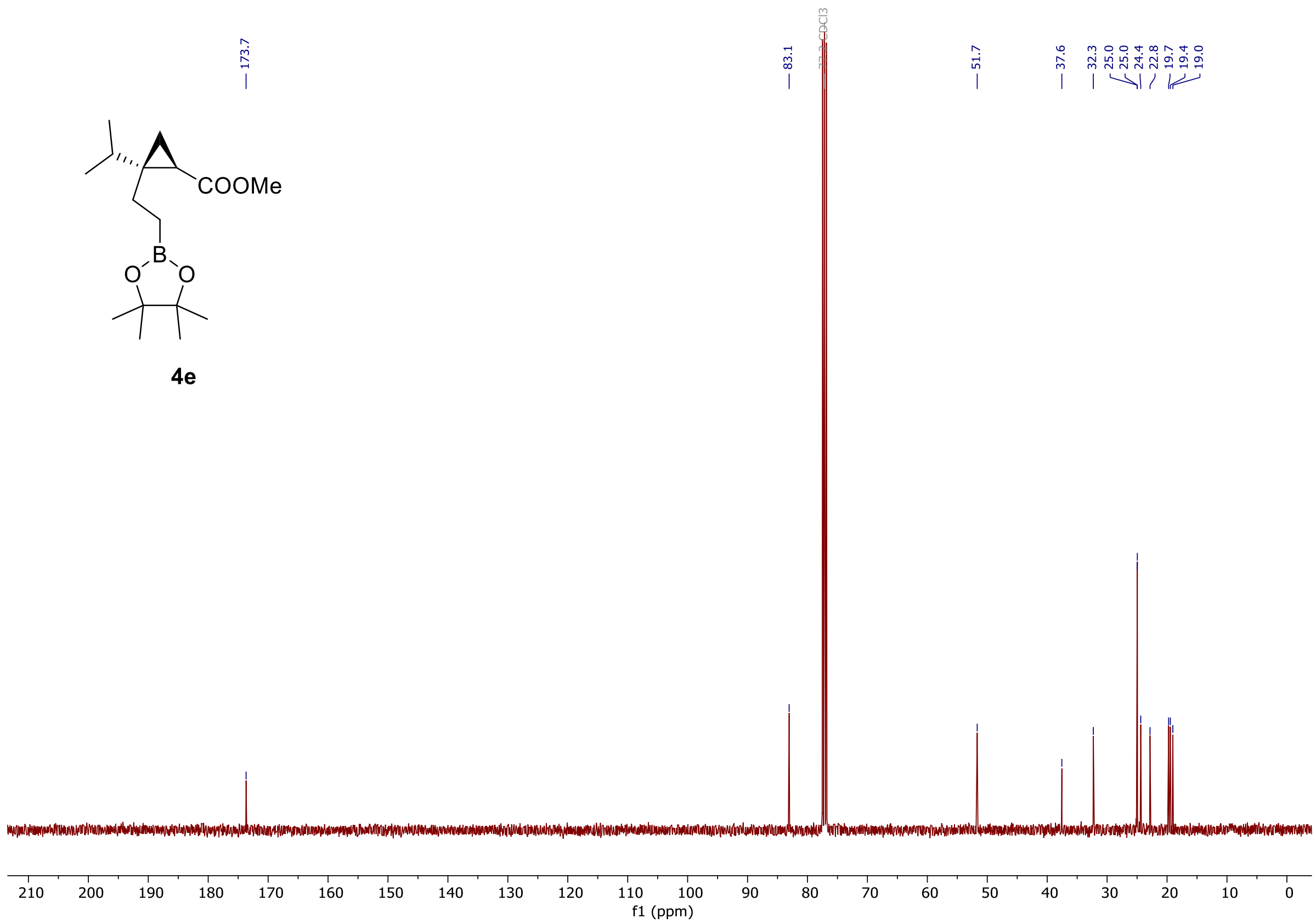

^{1}H NMR (400 MHz, $CDCl_3$) of **5** (*see procedure*)

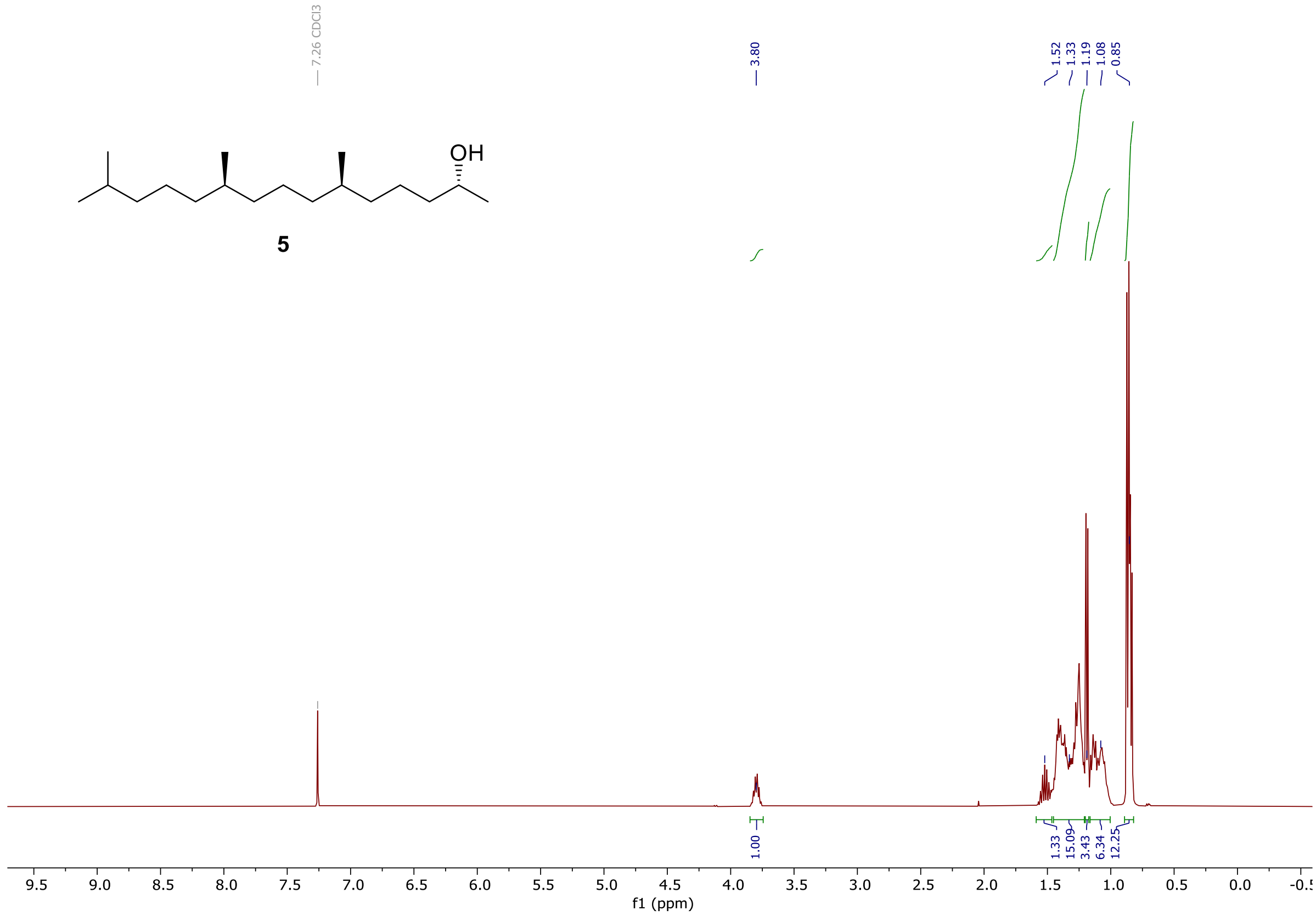

^{13}C NMR (101 MHz, $CDCl_3$) of **5**

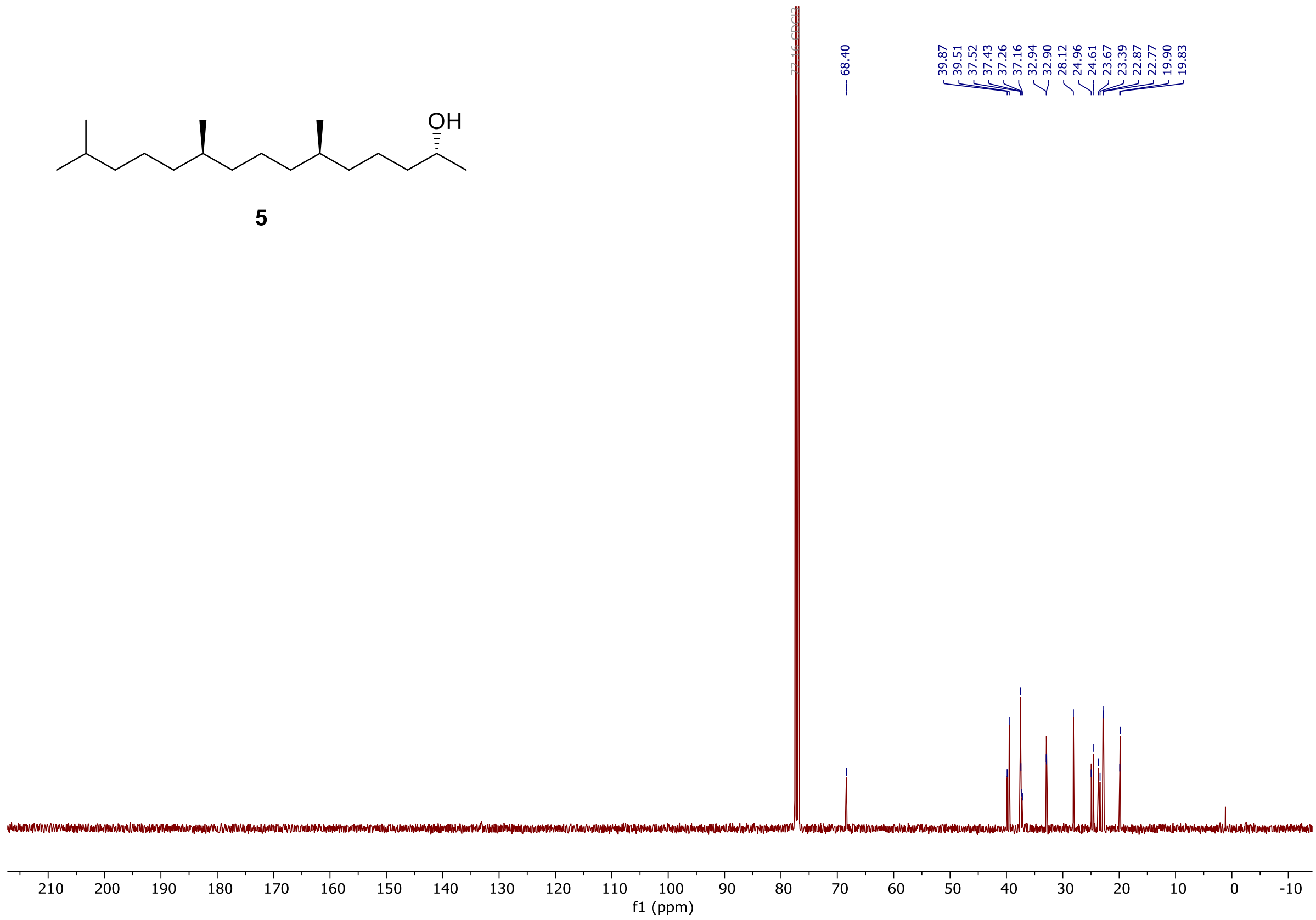

^{1}H NMR (400 MHz, $CDCl_3$) of **6** (*see procedure*)

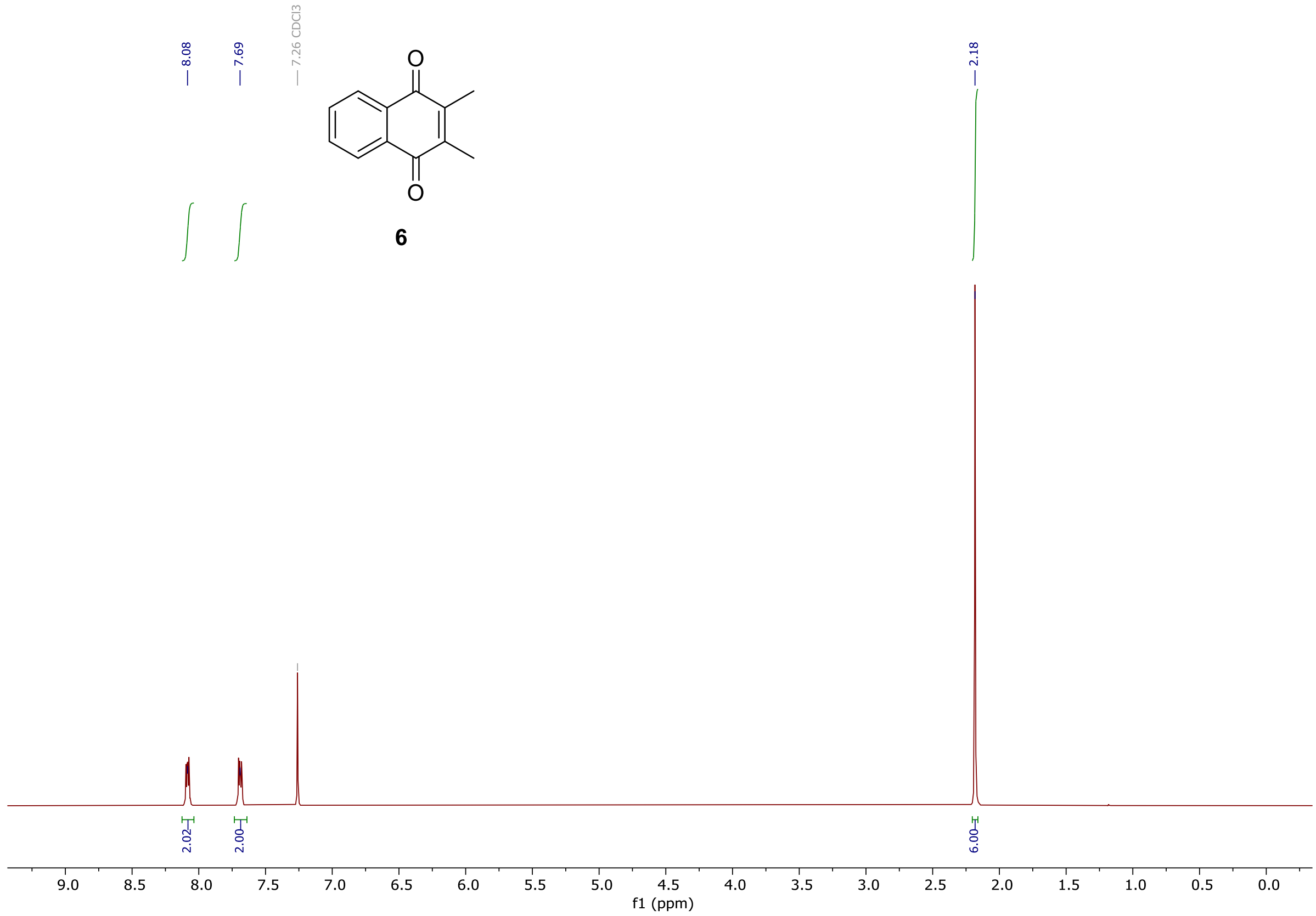

^{13}C NMR (101 MHz, $CDCl_3$) of **6**

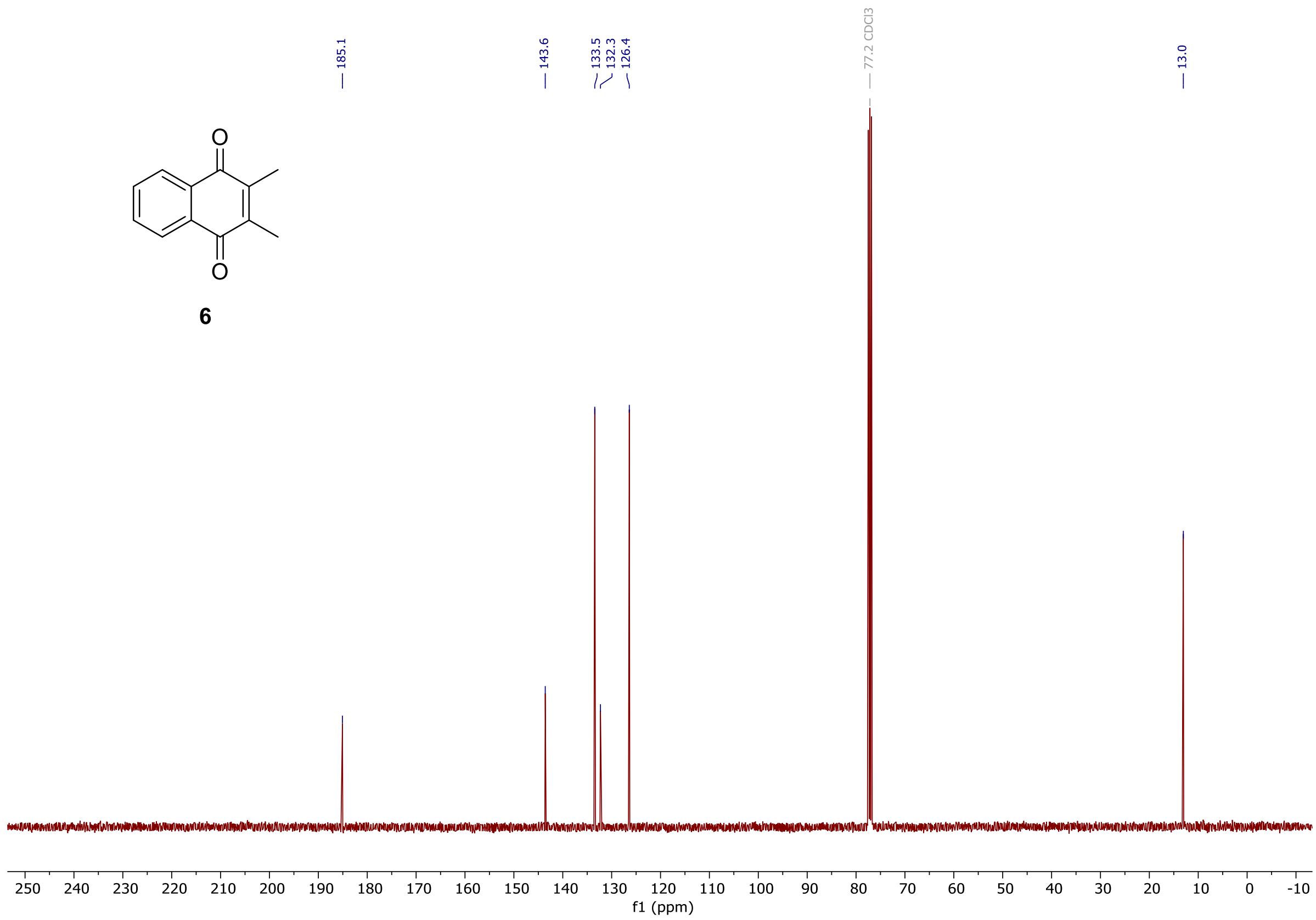

^{1}H NMR (400 MHz, $CDCl_3$) of **7** (*see procedure*)

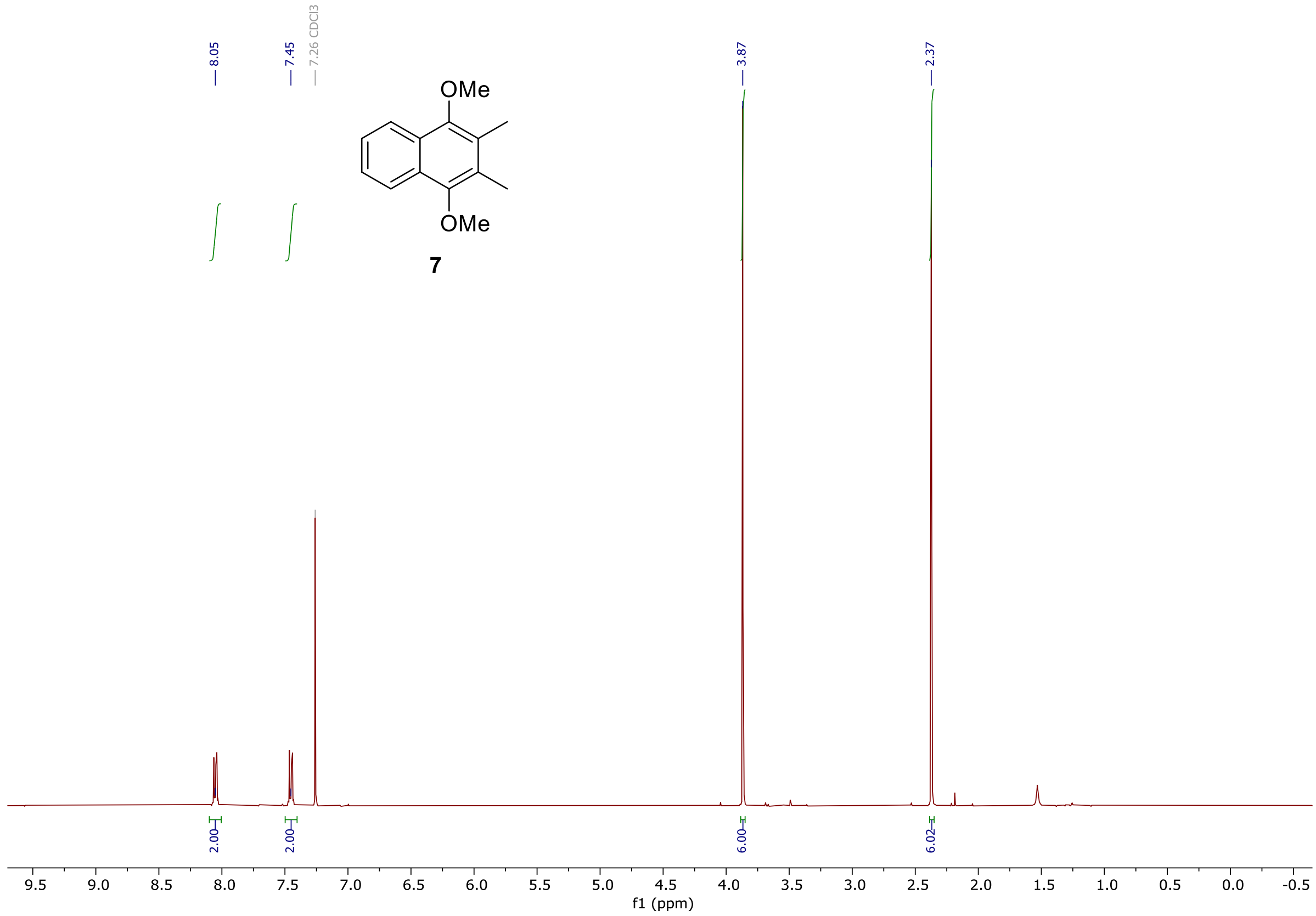

^{13}C NMR (101 MHz, $CDCl_3$) of **7**

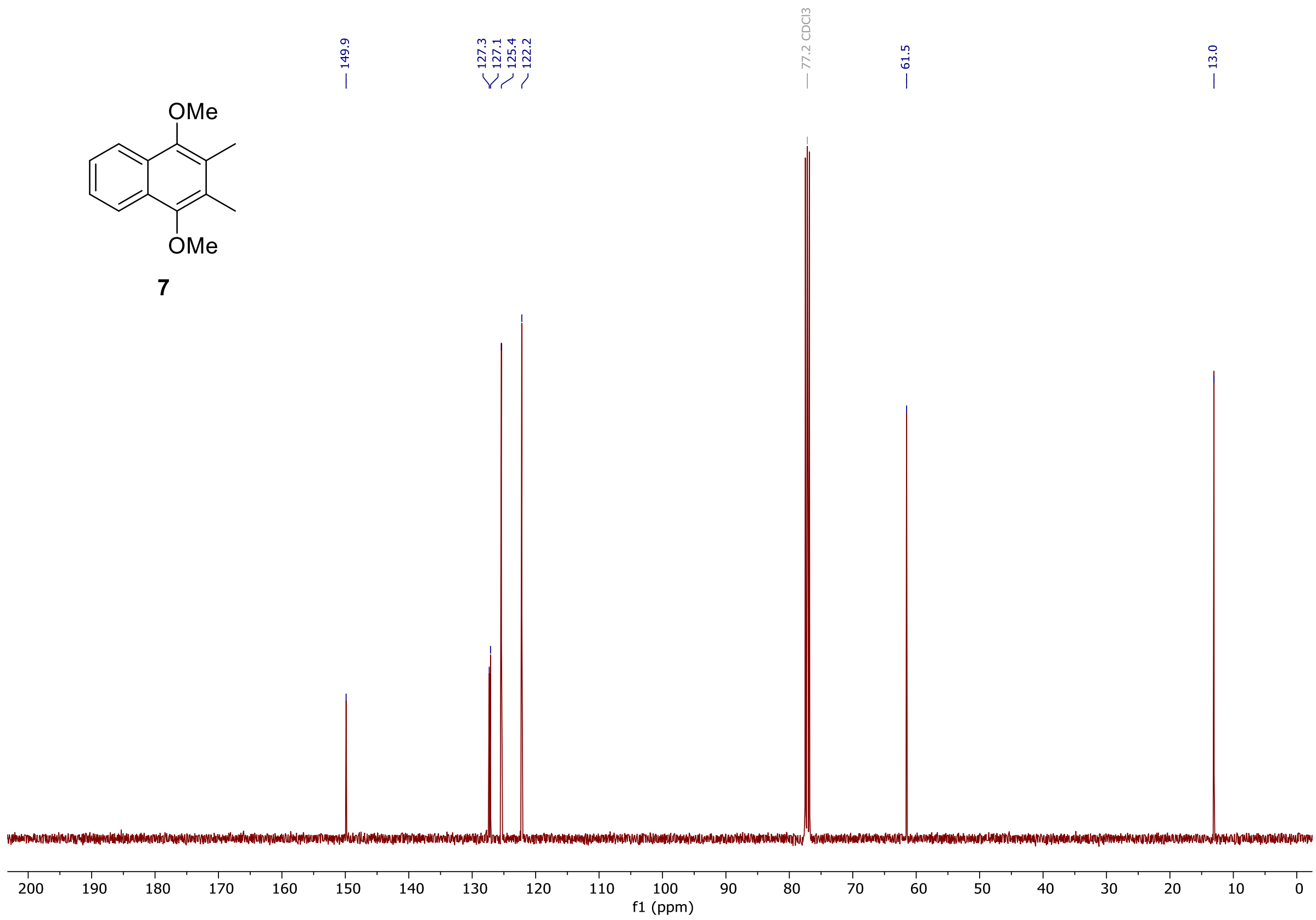

^{1}H NMR (400 MHz, $CDCl_3$) of **8** (*see procedure*)

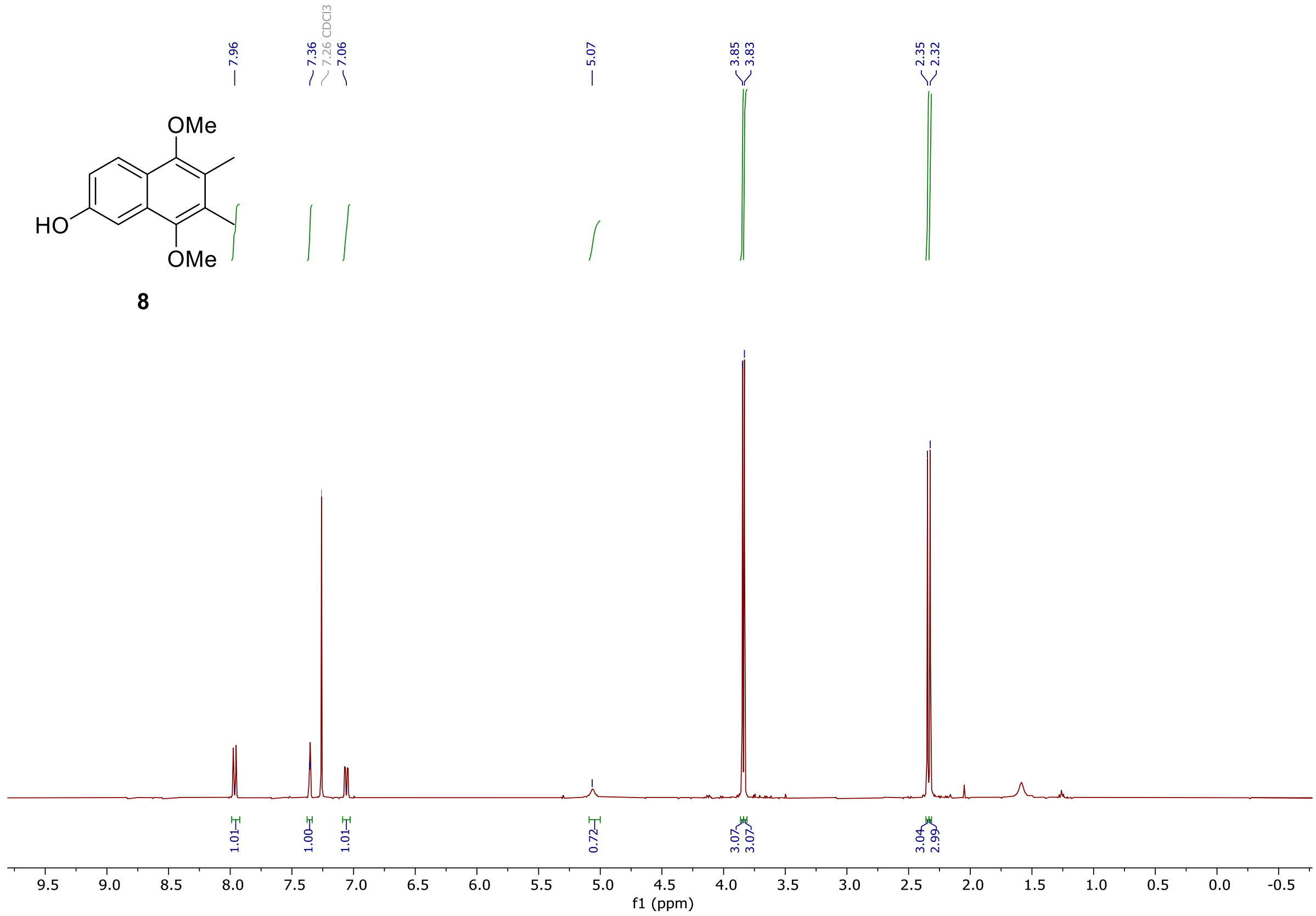

^{13}C NMR (101 MHz, $CDCl_3$) of **8**

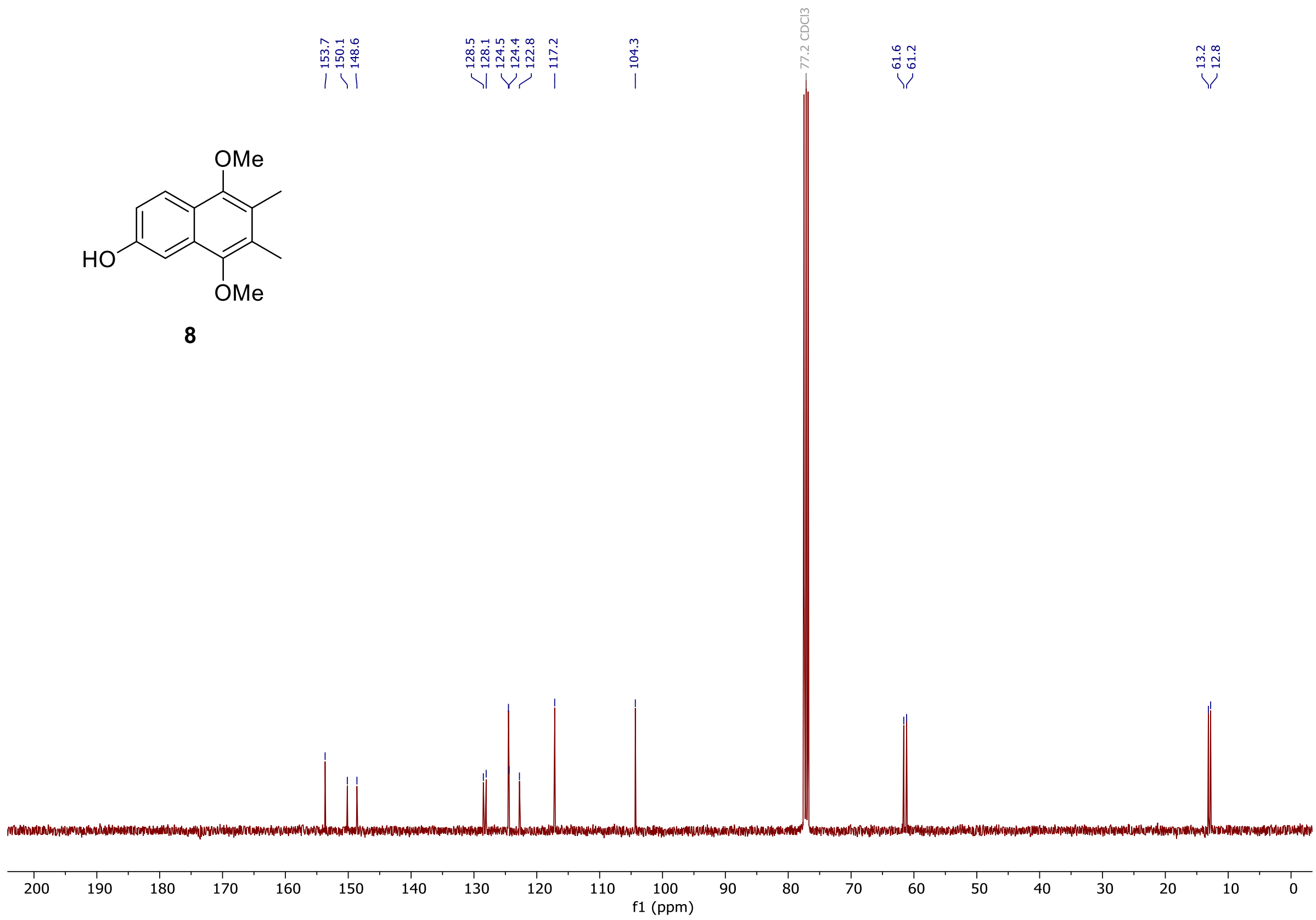

^{1}H NMR (400 MHz, $CDCl_3$) of **9** (*see procedure*)

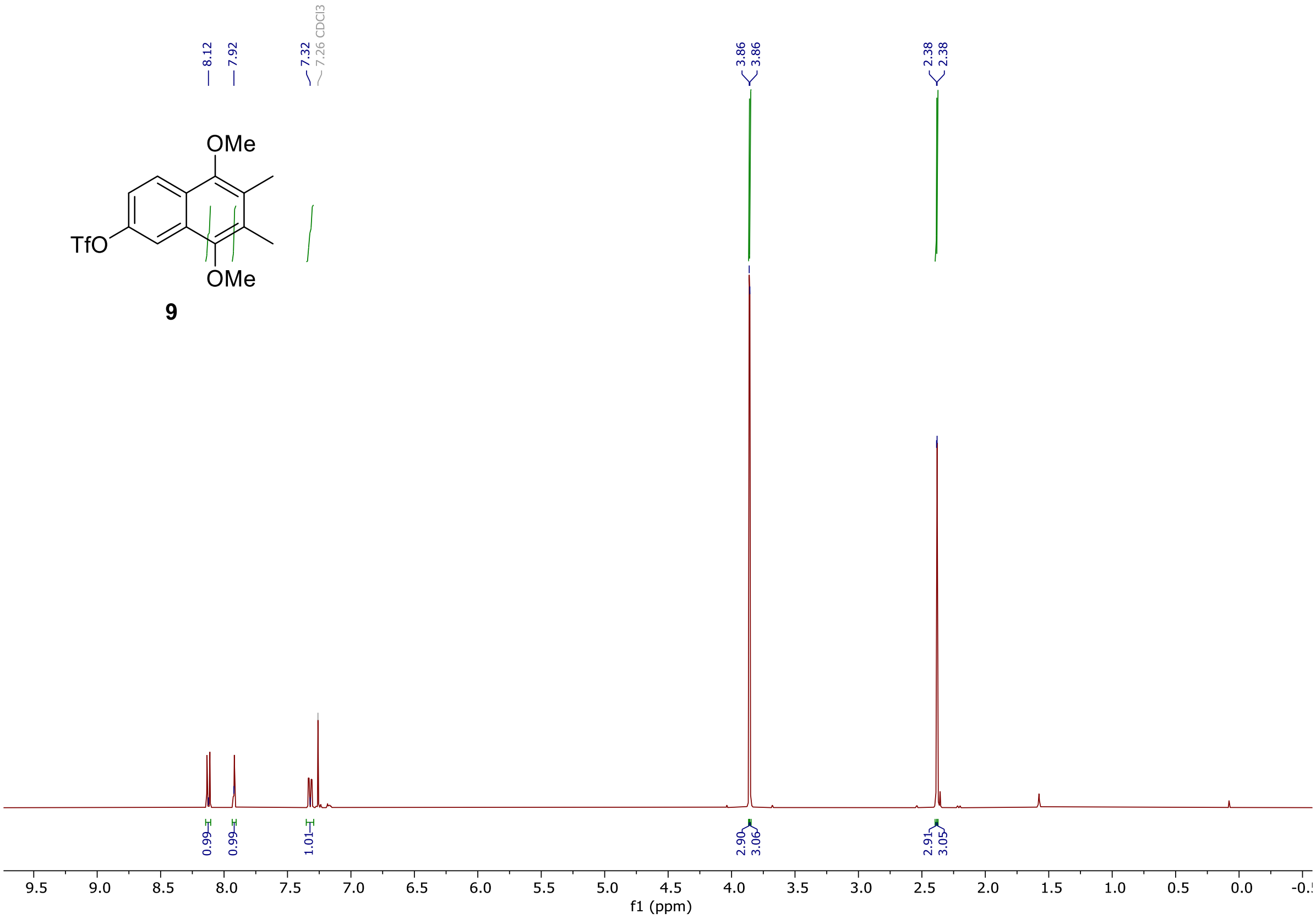

^{13}C NMR (101 MHz, $CDCl_3$) of **9**

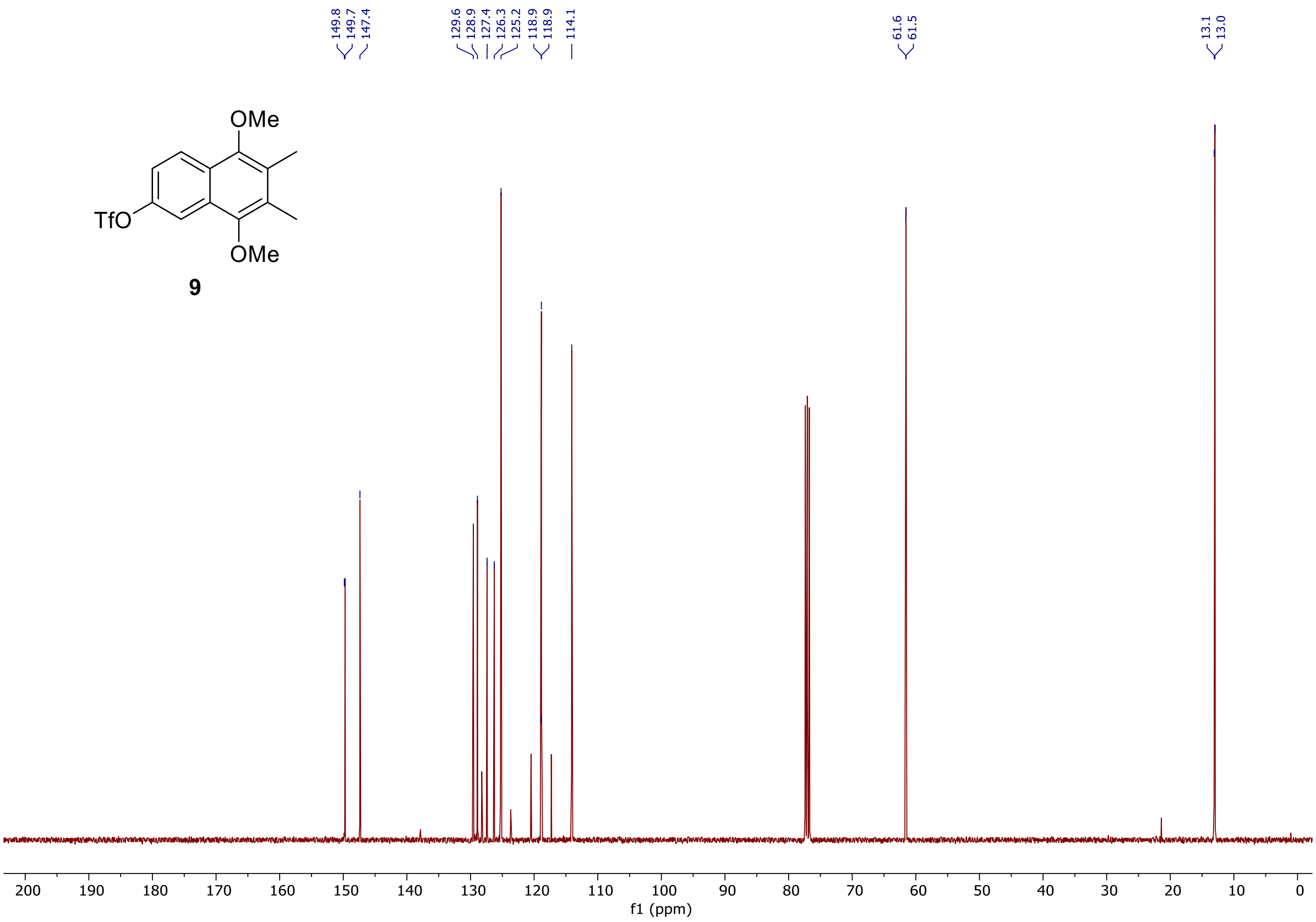

^{19}F NMR (377 MHz, $CDCl_3$) of **9**

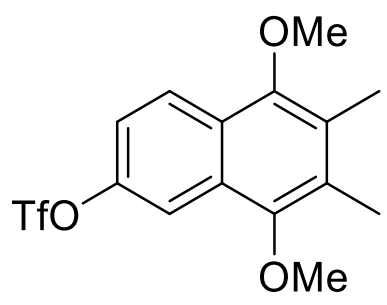

9

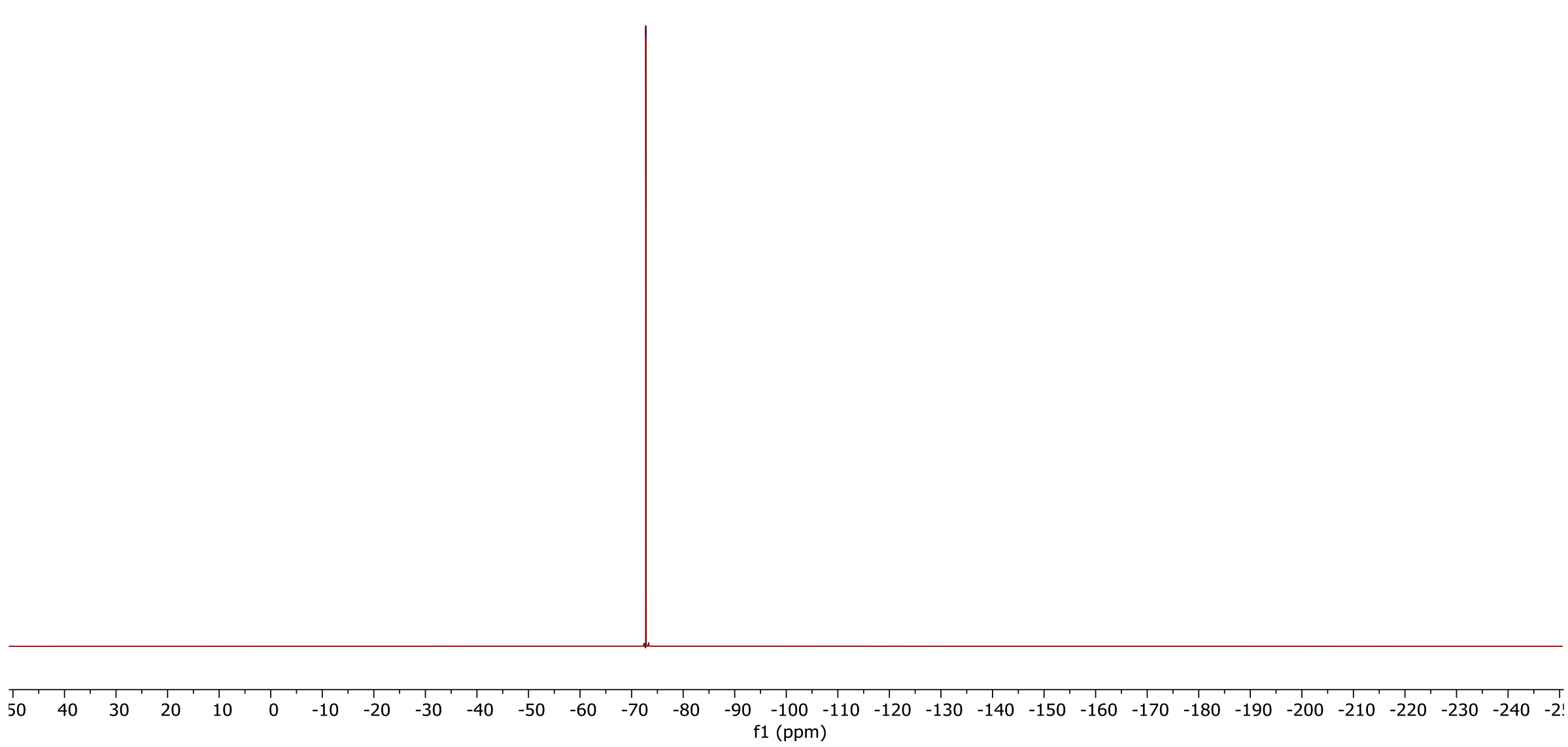

^{1}H NMR (400 MHz, $CDCl_3$) of **10** (*see procedure*)

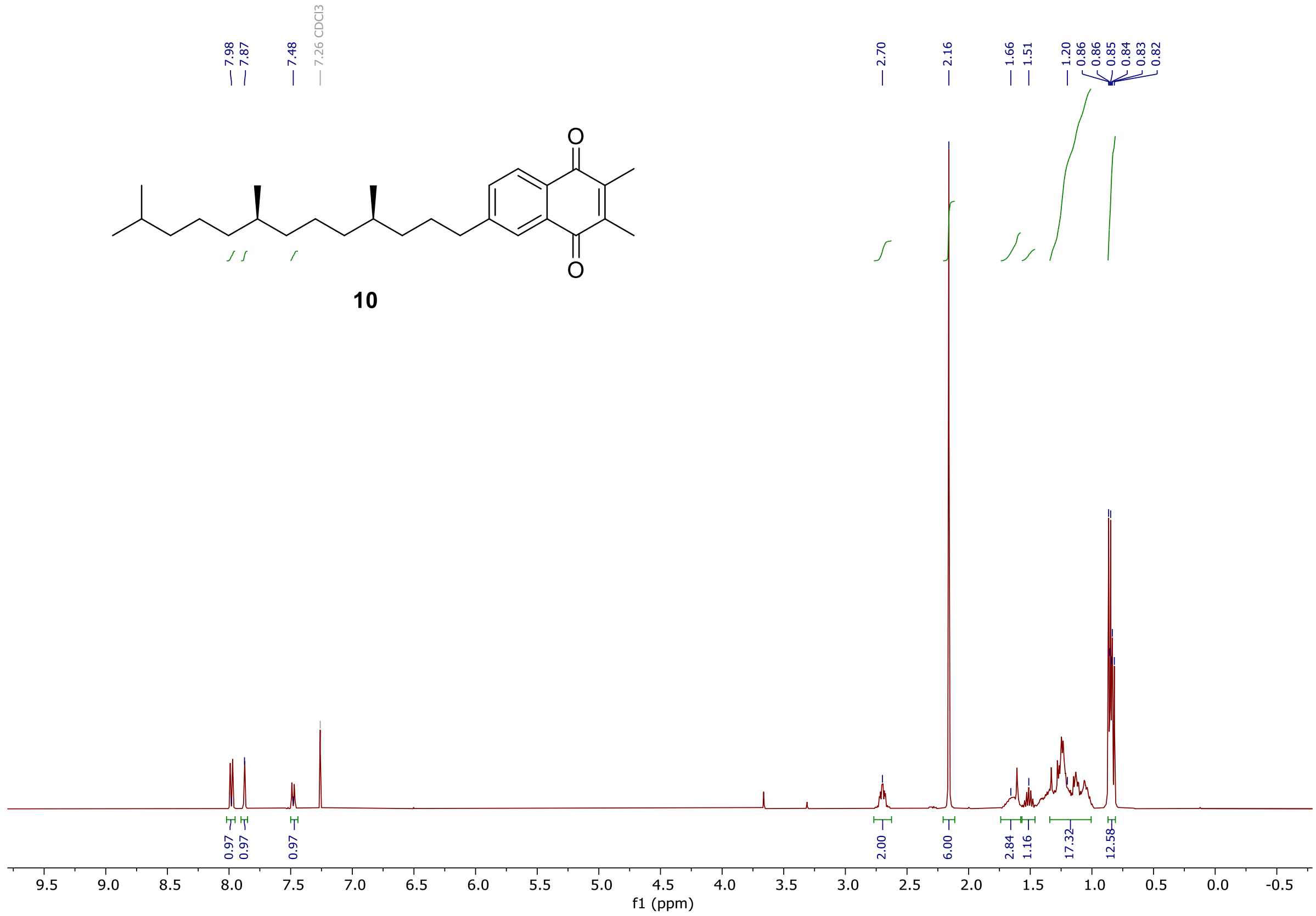

^{13}C NMR (101 MHz, $CDCl_3$) of **10**

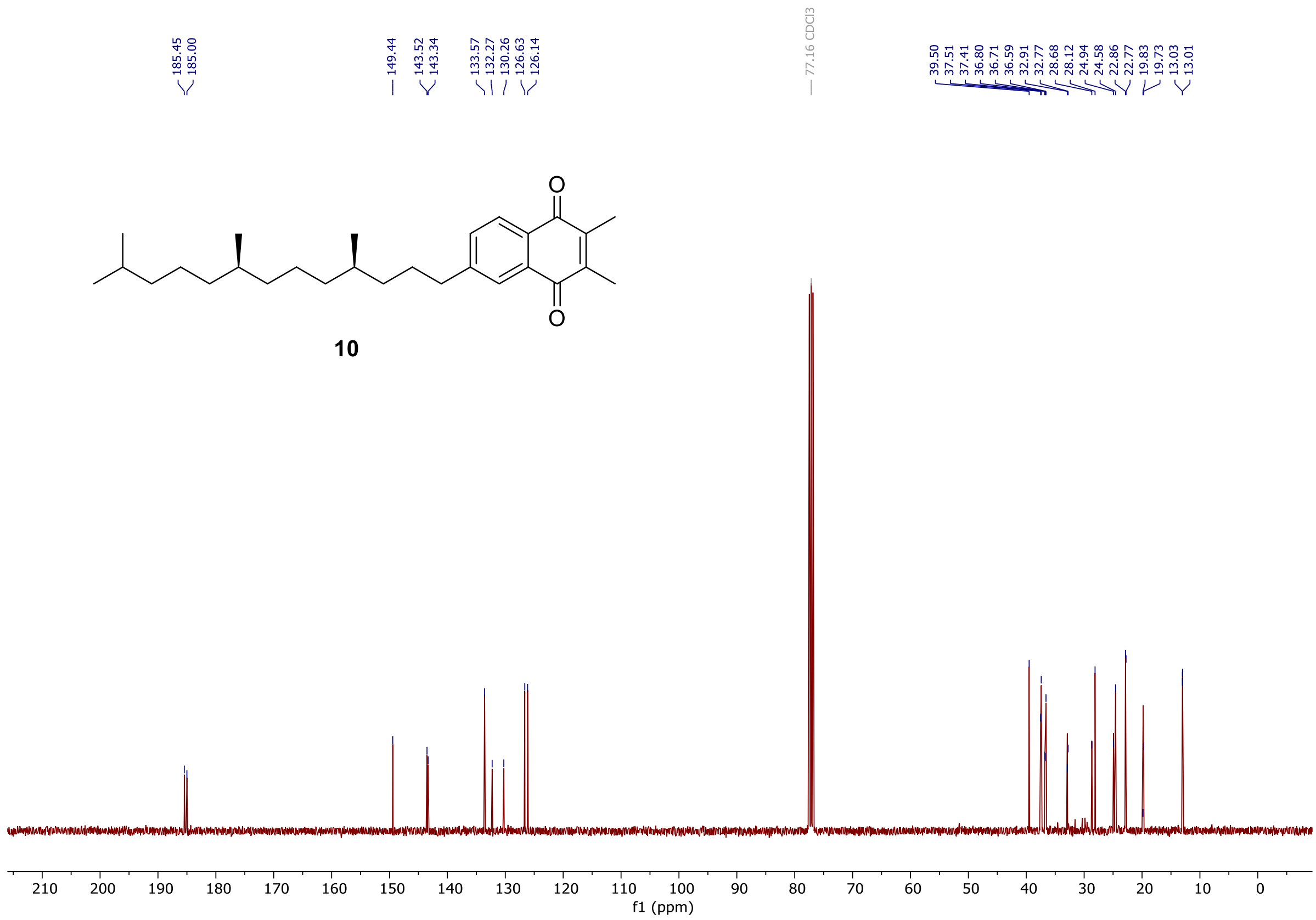

^{1}H NMR (400 MHz, $CDCl_3$) of **11** (*see procedure*)

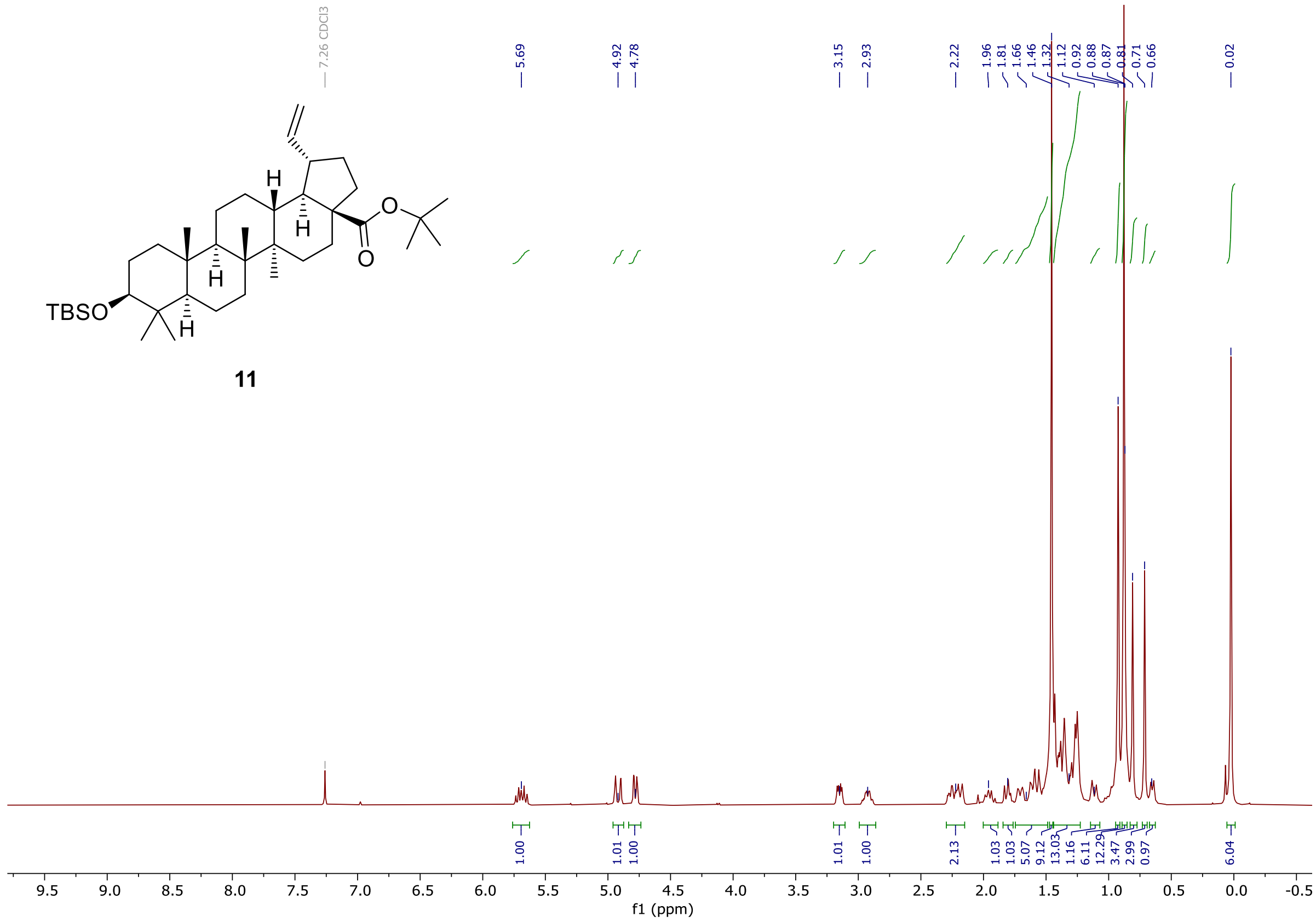

^{13}C NMR (101 MHz, $CDCl_3$) of **11**

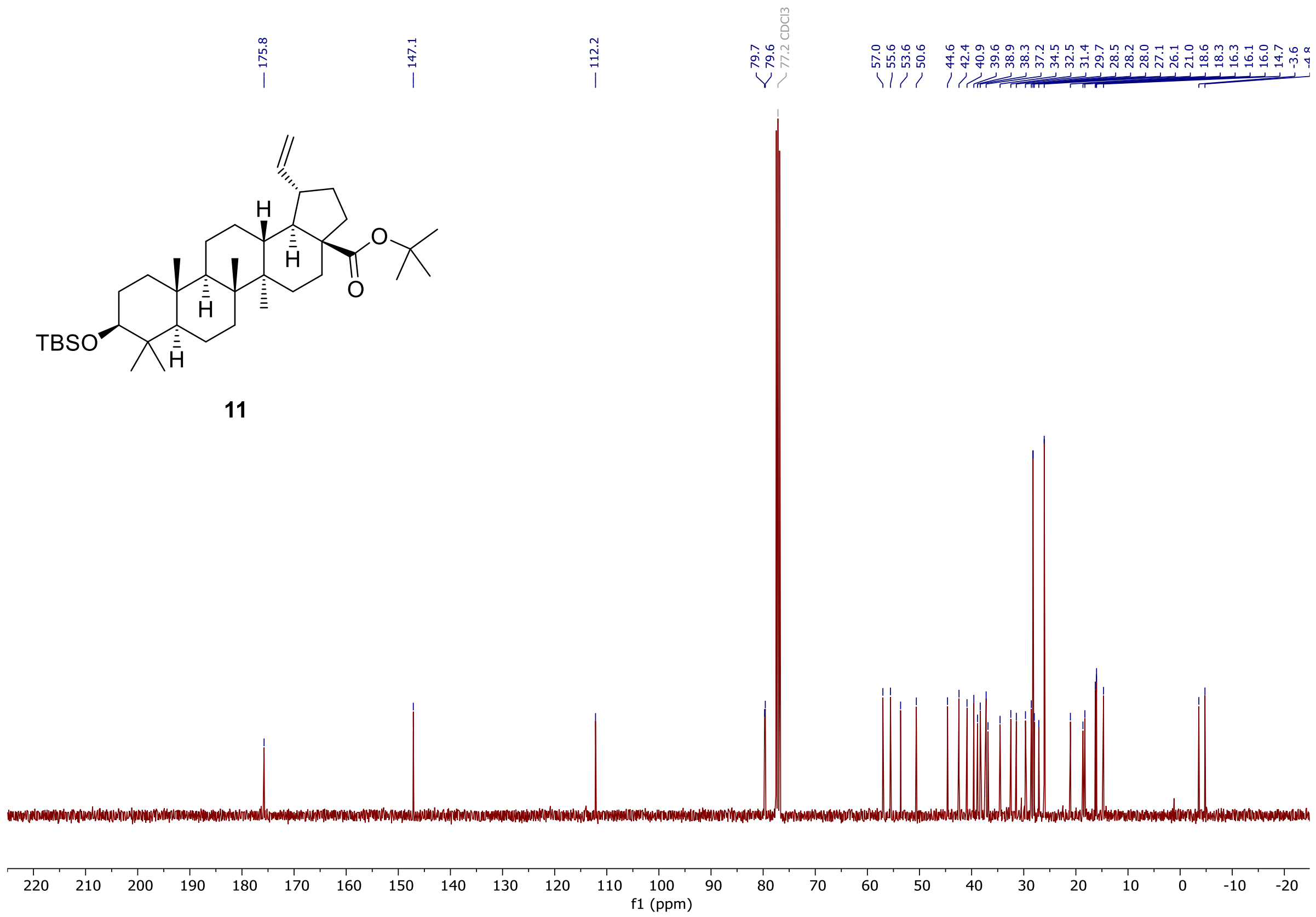

^{1}H NMR (400 MHz, CDCl₃) of **12** (*see procedure*)

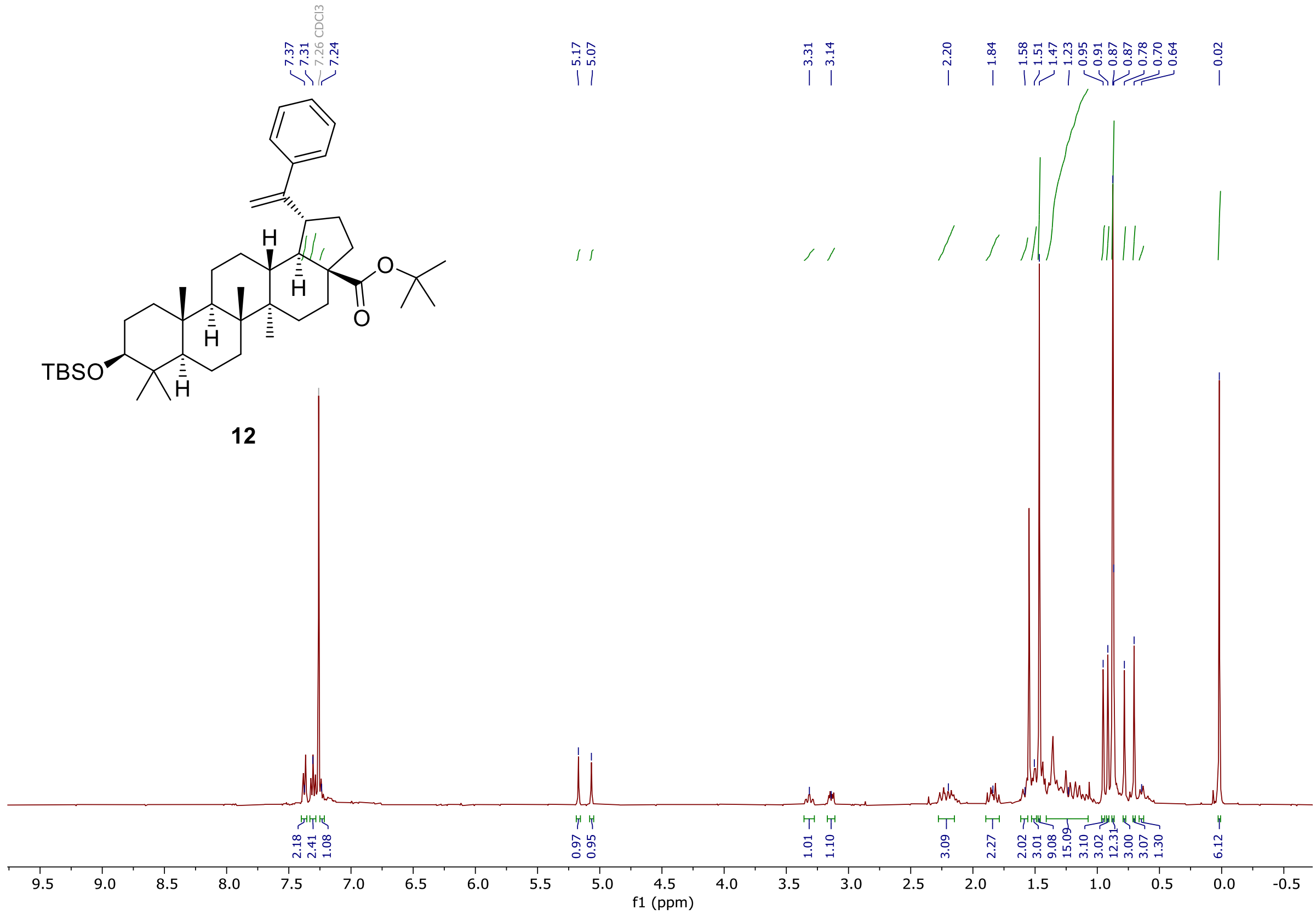

^{13}C NMR (126 MHz, CDCl₃) of **12**

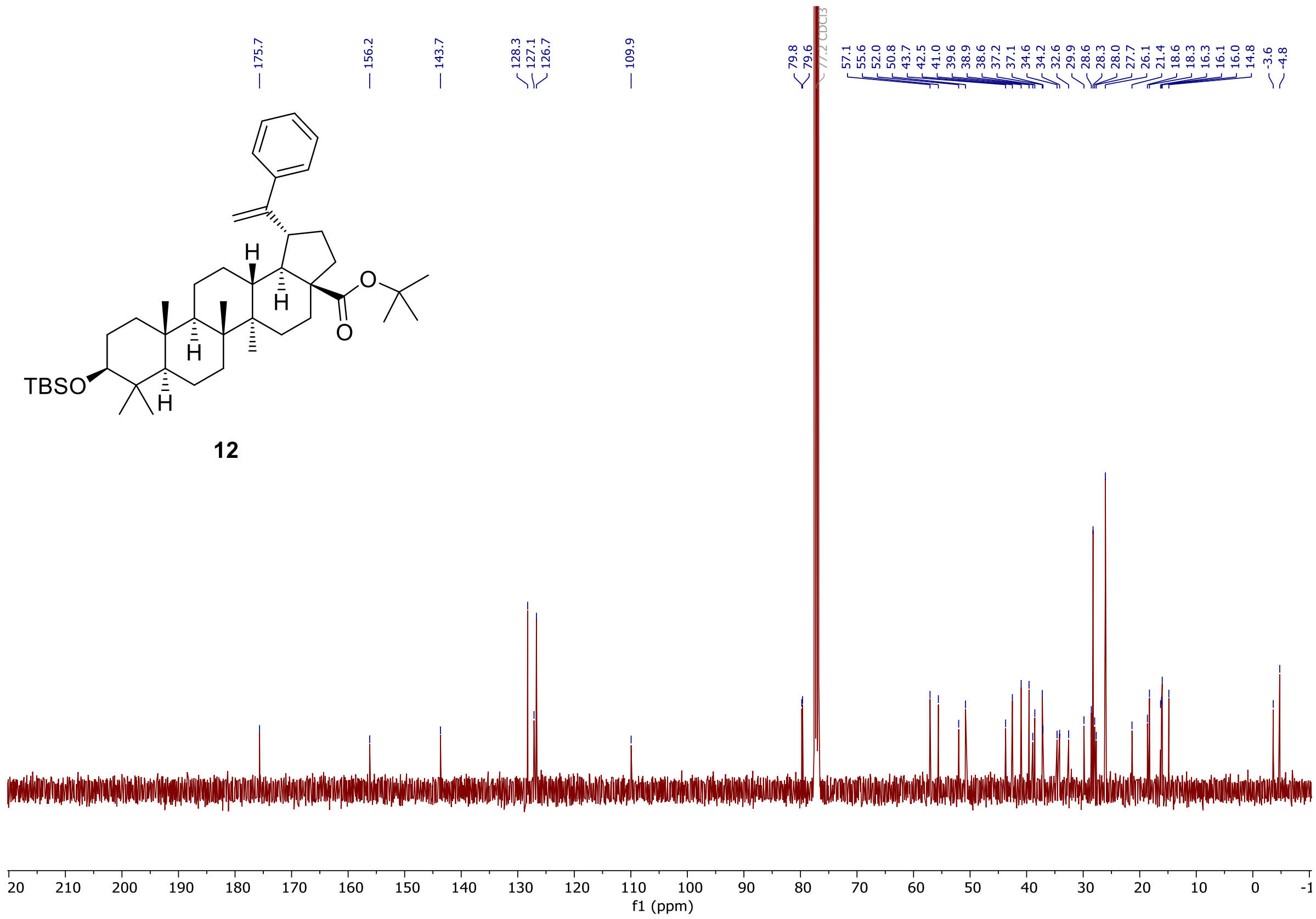

^{1}H NMR (400 MHz, $CDCl_3$) of **13** (*see procedure*)

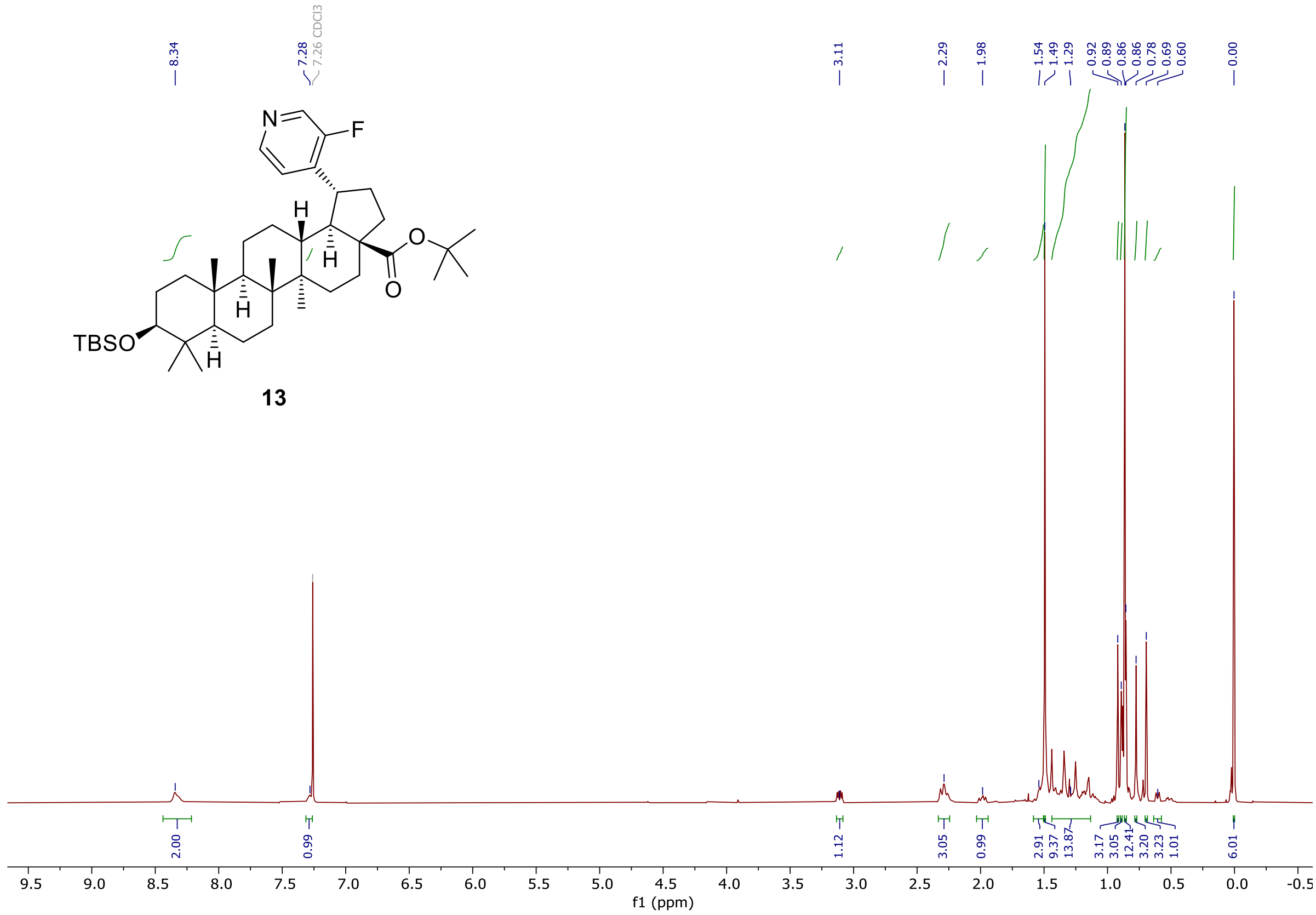

^{13}C NMR (101 MHz, $CDCl_3$) of **13**

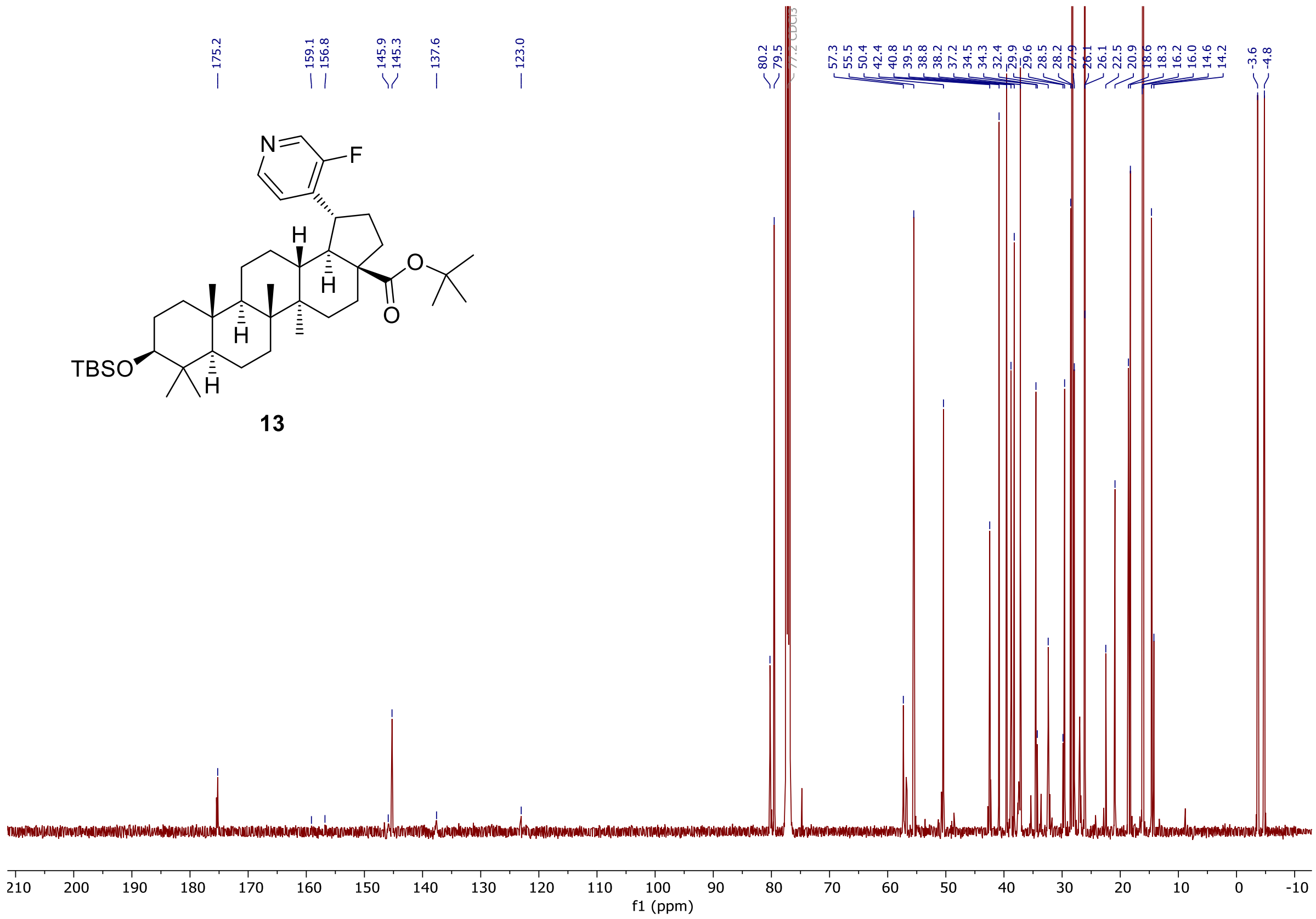

^{19}F NMR (377 MHz, $CDCl_3$) of **13**

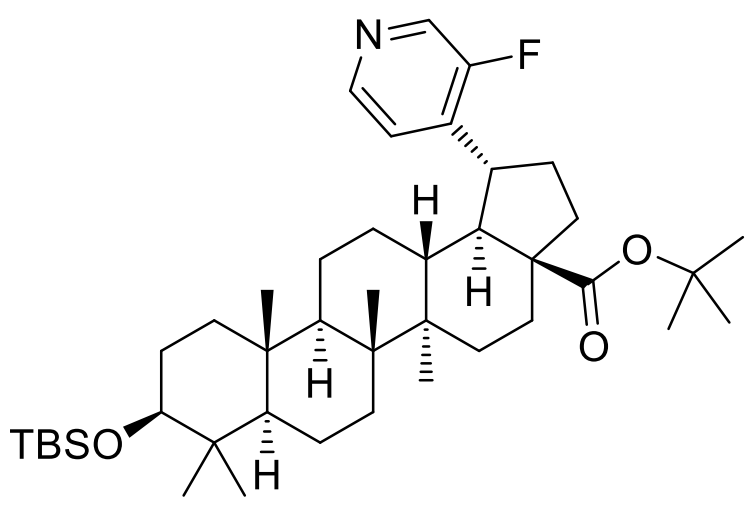

13

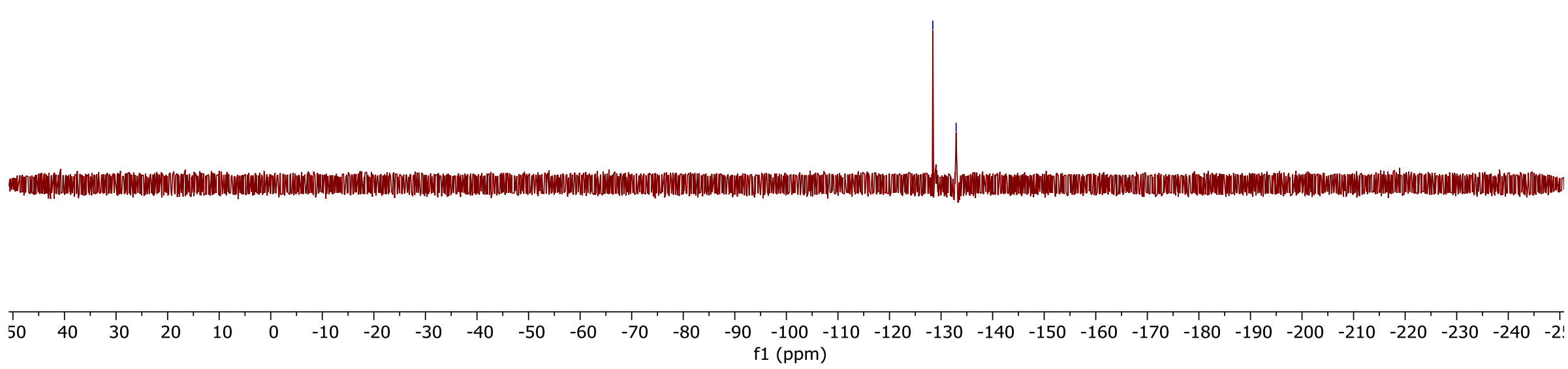

^{1}H NMR (400 MHz, $CDCl_3$) of **14** (*see procedure*)

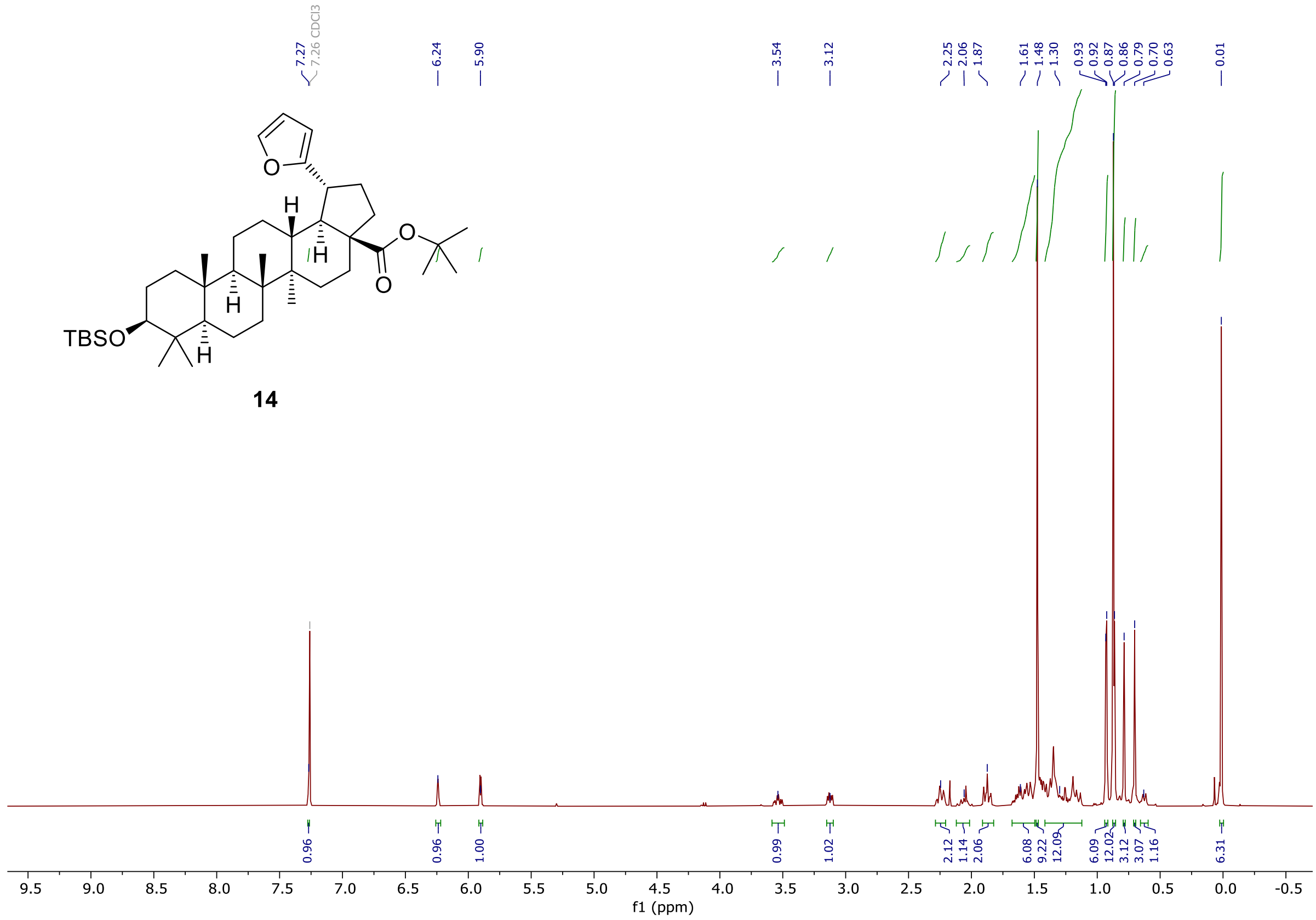

^{13}C NMR (101 MHz, $CDCl_3$) of **14**

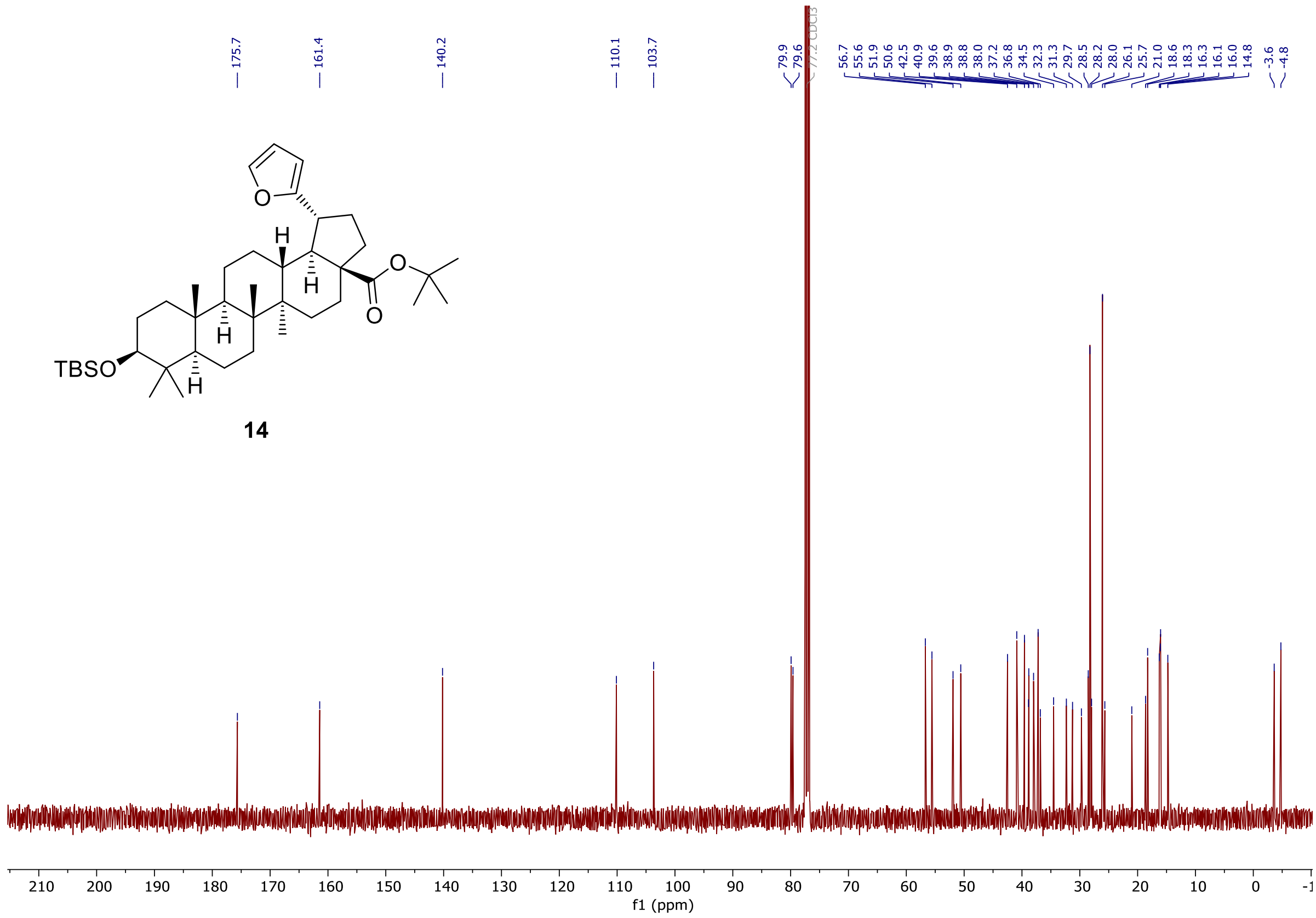

6. REFERENCES

(1) Burchat, A. F.; Chong, J. M.; Nielsen, N. Titration of alkyllithiums with a simple reagent to a blue endpoint. *J. Organomet. Chem.* **1997**, *542*, 281–283.

(2) Hitosugi, S.; Tanimoto, D.; Nakanishi, W.; Isobe, H. A Facile Chromatographic Method for Purification of Pinacol Boronic Esters. *Chem. Lett.* **2012**, *41*, 972–973.

(3) Siu, J. C.; Parry, J. B.; Lin, S. Aminoxyl-Catalyzed Electrochemical Diazidation of Alkenes Mediated by a Metastable Charge-Transfer Complex. *J. Am. Chem. Soc.* **2019**, *141*, 2825–2831.

(4) Reid, W. B.; Watson, D. A. Synthesis of Trisubstituted Alkenyl Boronic Esters from Alkenes Using the Boryl-Heck Reaction. *Org. Lett.* **2018**, *20*, 6832–6835.

(5) Gonnard, L.; Guérinot, A.; Cossy, J. Cobalt-Catalyzed Cross-Coupling of 3- and 4-Iodopiperidines with Grignard Reagents. *Chem. Eur. J.* **2015**, *21*, 12797–12803.

(6) Wollnitzke, P.; Essig, S.; Gölz, J. P.; von Schwarzenberg, K.; Menche, D. Total Synthesis of Ajudazol A by a Modular Oxazole Diversification Strategy. *Org. Lett.* **2020**, *22*, 6344–6348.

(7) Shen, K.; Feng, C.; Liu, Y.; Yi, D.; Lin, P.; Li, H.; Gong, Y.; Wei, S.; Fu, Q.; Zhang, Z. Visible light-enabled synthesis of phosphorylated indolizine and pyridoindole derivatives via HAT-mediated radical cascade cyclization. *Org. Biomol. Chem.* **2023**, *21*, 9341–9345.

(8) Essayan, D. E.; Schubach, M. J.; Smoot, J. M.; Puri, T.; Pronin, S. V. Directed Hydrogen Atom Transfer for Selective Reactions of Polyenols. *J. Am. Chem. Soc.* **2024**, *146*, 18224–18229.

(9) Gonzalez, M. D.; Burton, G. Preparation and carbon-13 NMR study of 3- and 20-methylene analogues of steroid hormones. *Magn. Reson. Chem.* **1988**, *26*, 963–966.

(10) Wolfram, R. K.; Heller, L.; Csuk, R. Targeting mitochondria: Esters of rhodamine B with triterpenoids are mitocanic triggers of apoptosis. *Eur. J. Med. Chem.* **2018**, *152*, 21–30.

(11) Green, S. A.; Vásquez-Céspedes, S.; Shenvi, R. A. Iron–Nickel Dual-Catalysis: A New Engine for Olefin Functionalization and the Formation of Quaternary Centers. *J. Am. Chem. Soc.* **2018**, *140*, 11317–11324.

(12) Govaerts, S.; Mayer-Figge, J. L.; Chotia, M.; Kirsch, S. F.; Gómez-Suárez, A. Synthesis and Nucleophilic Ring-Opening of 1,1-Dicyanocyclopropanes: Accessing β-Aminocarbonyl Derivatives from Olefins. *Org. Lett.* **2025**, *27*, 5549–5554.

(13) Zhang, L.; Zuo, Z.; Leng, X.; Huang, Z. A Cobalt-Catalyzed Alkene Hydroboration with Pinacolborane. *Angew. Chem. Int. Ed.* **2014**, *53*, 2696–2700.

(14) Yi, J.; Liu, J.-H.; Liang, J.; Dai, J.-J.; Yang, C.-T.; Fu, Y.; Liu, L. Alkylboronic Esters from Palladium- and Nickel-Catalyzed Borylation of Primary and Secondary Alkyl Bromides. *Adv. Synth. Catal.* **2012**, *354*, 1685–1691.

(15) Sandfort, F.; Strieth-Kalthoff, F.; Klauck, F. J. R.; James, M. J.; Glorius, F. Deaminative Borylation of Aliphatic Amines Enabled by Visible Light Excitation of an Electron Donor–Acceptor Complex. *Chem. Eur. J.* **2018**, *24*, 17210–17214.

(16) Chen, S.-C.; Zhu, Q.; Chen, H.; Chen, Z.; Luo, T. Pro-aromaticity Enabled Dealkenylative Functionalizations via Photo-Excitation and Oxidation. *Chem. Eur. J.* **2023**, *29*, e202203425.

(17) Guo, P.; Jin, H.; Han, J.; Xu, L.; Li, P.; Zhan, M. Nickel-Catalyzed Negishi Cross-Coupling of Alkyl Halides, Including Unactivated Tertiary Halides, with a Boron-Stabilized Organozinc Reagent. *Org. Lett.* **2023**, *25*, 1268–1273.

(18) Nakamura, Y.; Mori, K. New Syntheses of the Rice Moth and Stink Bug Pheromones by Employing (2*R*, 6*S*)-7-Acetoxy-2,6-dimethyl-1-heptanol as a Building Block. *Biosci. Biotechnol. Biochem.* **2000**, *64*, 1713–1721.

(19) Yang, J.; Dong, Y.; He, S.; Shi, Z.-C.; Wang, Y.; Wang, J.-Y. Bismuth-catalyzed methylation and alkylation of quinone derivatives with tert-butyl peroxybenzoate as an oxidant. *Tetrahedron* **2019**, *75*, 130729.

(20) Mastachi-Loza, S.; Ramírez-Candelero, T. I.; González-Romero, C.; Díaz-Torres, E.; Fuentes-Benítes, A.; Tamariz, J. Reactivity of N-Substituted Exo-oxazolidin-2-one Dienes with Naphthalene Chalcones and Cyclic 1,3-Dicarbonyl Compounds. *Asian J. Chem.* **2018**, *7*, 2120–2125.

(21) de Carvalho, R. L.; Wood, J. M.; Almeida, R. G.; Berry, N. G.; da Silva Júnior, E. N.; Bower, J. F. The Synthesis and Reactivity of Naphthoquinonynes. *Angew. Chem. Int. Ed.* **2024**, *63*, e202400188.

(22) Ferguson, R. N.; Whidby, J. F.; Sanders, E. B.; Levins, R. J.; Katz, T.; DeBardeleben, J. F.; Einolf, W. N. Isolation and identification of 2,3-dimethyl-6-(4,8,12-trimethyltridecyl)-1,4-naphthoquinone from tobacco and smoke. *Tetrahedron Lett.* **1978**, *19*, 2645–2648.

(23) Li, J.; Wang, H.; Qiu, Z.; Huang, C.-Y.; Li, C.-J. Metal-Free Direct Deoxygenative Borylation of Aldehydes and Ketones. *J. Am. Chem. Soc.* **2020**, *142*, 13011–13020.
